# Prognostic Modeling of Lung Adenocarcinoma Based on Hypoxia and Ferroptosis-Related Genes

**DOI:** 10.1155/2022/1022580

**Published:** 2022-09-19

**Authors:** Chang Liu, Yan-Qin Ruan, Lai-Hao Qu, Zhen-Hua Li, Chao Xie, Ya-Qiang Pan, Hao-Fei Li, Ding-Biao Li

**Affiliations:** ^1^Thoracic Surgery, Yan'an Affiliated Hospital of Kunming Medical University, Kunming Medical University, Kunming 650000, Yunnan Province, China; ^2^Key Laboratory of Tumor Immunological Prevention and Treatment of Yunnan Province, Yan'an Affiliated Hospital of Kunming Medical University, Kunming Medical University, Kunming 650000, Yunnan Province, China; ^3^Guizhou Center for Disease Control and Prevention, Guiyang, Guizhou Province 550001, China; ^4^The First Clinical College of Xinxiang Medical University, Xinxiang, Henan Province 453003, China

## Abstract

**Background:**

It is well known that hypoxia and ferroptosis are intimately connected with tumor development. The purpose of this investigation was to identify whether they have a prognostic signature. To this end, genes related to hypoxia and ferroptosis scores were investigated using bioinformatics analysis to stratify the risk of lung adenocarcinoma.

**Methods:**

Hypoxia and ferroptosis scores were estimated using The Cancer Genome Atlas (TCGA) database-derived cohort transcriptome profiles via the single sample gene set enrichment analysis (ssGSEA) algorithm. The candidate genes associated with hypoxia and ferroptosis scores were identified using weighted correlation network analysis (WGCNA) and differential expression analysis. The prognostic genes in this study were discovered using the Cox regression (CR) model in conjunction with the LASSO method, which was then utilized to create a prognostic signature. The efficacy, accuracy, and clinical value of the prognostic model were evaluated using an independent validation cohort, Receiver Operator Characteristic (ROC) curve, and nomogram. The analysis of function and immune cell infiltration was also carried out.

**Results:**

Here, we appraised 152 candidate genes expressed not the same, which were related to hypoxia and ferroptosis for prognostic modeling in The Cancer Genome Atlas Lung Adenocarcinoma (TCGA-LUAD) cohort, and these genes were further validated in the GSE31210 cohort. We found that the 14-gene-based prognostic model, utilizing *MAPK4*, *TNS4*, *WFDC2*, *FSTL3*, *ITGA2*, *KLK11*, *PHLDB2*, *VGLL3*, *SNX30*, *KCNQ3*, *SMAD9*, *ANGPTL4*, *LAMA3*, and *STK32A*, performed well in predicting the prognosis in lung adenocarcinoma. ROC and nomogram analyses showed that risk scores based on prognostic signatures provided desirable predictive accuracy and clinical utility. Moreover, gene set variance analysis showed differential enrichment of 33 hallmark gene sets between different risk groups. Additionally, our results indicated that a higher risk score will lead to more fibroblasts and activated CD4 *T*  cells but fewer myeloid dendritic cells, endothelial cells, eosinophils, immature dendritic cells, and neutrophils.

**Conclusion:**

Our research found a 14-gene signature and established a nomogram that accurately predicted the prognosis in patients with lung adenocarcinoma. Clinical decision-making and therapeutic customization may benefit from these results, which may serve as a valuable reference in the future.

## 1. Introduction

Lung cancer is one of the most frequent malignancies with high mortality and poor prognosis [1, 2]; 80% of lung malignancies diagnosed were NSCLC [3]. LUAD accounts for nearly 40% of NSCLC cases [4, 5], and its incidence is continually increasing [6]. In recent years, several therapeutic advances have been made, including targeted therapies and emerging immunotherapy [7, 8]. Although both methods are effective in a restricted range of lung cancer subtypes, the rate of survival for LUAD is still poor [9]. According to statistics, LUAD has a poor prognosis that only 18% could survive longer than 5 years [10]. As a result, the search for valid biomarkers might lead to the establishment of individualized diagnosis and therapy for LUAD patients [11]. The cancer tissue has many specific characteristics, including accelerated cell cycle, alterations of the genome, increase in cell mobility and invasive growth of the cells, incapable of going through normal apoptosis process, and depletion of normal cell functions. Because of these physiological and pathological characteristics, it is difficult for tumors to be treated.

Recently, it has been studied that ferroptosis is a relatively new type of cell death. This process is often accompanied by significant iron buildup and lipid peroxidation in dying cells [12]. It can be distinguished from apoptosis, necrosis, and autophagy by certain key characteristics. Firstly, it is iron-dependent and is induced by the buildup of harmful lipid reactive oxygen species. In addition, polyunsaturated fatty acids are consumed during the process [12]. With the rapid development of the role of iron ions in cancer, new prospects have emerged for their use in cancer therapy [13]. The expression of the S100 calcium-binding protein A4 (FSP1) in lung cancer cell lines is related to resistance to ferroptosis, suggesting that overexpression of FSP1 may be a method for ferroptosis escape [14]. In addition, MAPK pathway activation is associated with the susceptibility to ferroptosis triggered by cystine deprivation in NSCLC cell lines [15]^.^ Alvarez et al. [16] recently found that inhibiting the iron-sulfur cluster biosynthesis enzyme NFS1 induced ferroptosis in vitro and slowed tumor development in LUAD. Additionally, Liu et al. [17] discovered that brusatol, an inhibitor of NRF2, increased the response rate of cystine deprivation-triggered ferroptosis through the FOCAD-FAK signaling pathway in NSCLC cell lines. What is more surprising is that the merger of brusatol and erastin demonstrated a superior therapeutic effect on NSCLC. The findings in these prior studies suggest that ferroptosis is quite important for lung cancer treatment. Based on the above research, we made the following hypothesis that ferroptosis is connected with the prognosis of LUAD, and thus ferroptosis-related genes may function as prognostic biomarkers.

Hypoxia or oxygen deprivation is a feature of most solid tumors because the growth of a tumor requires a large amount of oxygen. As the rapid tumor growth outstrips the supply of oxygen, an imbalance between decreased oxygen supply and increased oxygen demand was formed. This is a typical feature observed in the tumor microenvironment (TME) that increases the aggressiveness of many tumors and also causes abnormal blood vessel formation due to impaired blood supply, leading to poorer clinical outcomes [18–20]. Many transcription factors are active in tumor cells when the environment is hypoxic, and these transcription factors regulate cell proliferation, motility, and apoptosis via a variety of downstream signaling mechanisms [21]. This leads to an immunosuppressive TME that reduces the effectiveness of immunotherapy [22] and upregulates the expression of PD-L1, further supporting cancer escape [23, 24]. Although several studies have shown that intratumoral hypoxia and HIF1A expression affect overall survival (OS) in LUAD [25–27], hypoxia-based cannot be used to estimate who are at a high risk very early.

According to recent research, HIF1A may influence lipid metabolism and cause lipids to be stored in droplets, which reduces peroxidation-mediated endosomal damage and limits cellular ferroptosis [28]. Additionally, HIF-2*α* has been reported to activate hypoxia-inducible lipid droplet-associated (HILPDA) expression and selectively enrich polyunsaturated lipids, thus promoting cellular ferroptosis [29]. Furthermore, increased ferritin heavy chains under hypoxic conditions can protect HT1080 tumor cells from ferroptosis [30]. These findings suggest a potential relationship between ferroptosis and hypoxia. But more research is needed to further investigate how ferroptosis and hypoxia interact with each other and how they can affect LUAD patients' prognosis.

A variety of models have been created to predict the prognostication in LUAD according to the TME [31], ferroptosis [32], hypoxia [33], and tumor immunology [34]. However, to our knowledge, there is no reported prognostic role of hypoxia and ferroptosis-interrelated features in LUAD. To fill the gap and broaden the diagnostic and therapeutic potential of LUAD, we performed a comprehensive analysis using TCGA and Gene Expression Omnibus (GEO), aiming to endorse the least prognostic genes for LUAD. Finally, a signature on hypoxia- and ferroptosis-interrelated genes was constructed to know the prognostic value in LUAD patients.

## 2. Materials and Methods

### 2.1. Data Source

Transcriptomic data from 593 samples, composed of 59 normal and 534 LUAD, from TCGA database were used in this study. A total of 476 LUAD samples had available survival data. The GSE31210 dataset [35, 36] (https://www.ncbi.nlm.nih.gov/geo/query/acc.cgi?acc=GSE31210), containing transcriptomic data and survival information for 226 LUAD patients, was obtained from the GEO database to validate the established model.

### 2.2. Single Sample Gene Set Enrichment Analysis

The MSigDB (https://www.gsea-msigdb.org/gsea/msigdb/) was performed to acquire the hallmark gene sets of hypoxias, which consisted of 200 genes. The results show that there are 259 genes related to ferroptosis in total, which were gathered from the FerrDb database (https://www.zhounan.org/ferrdb/). The TCGA-LUAD database matched the expression patterns of the aforementioned genes. The ssGSEA method (from the *R* package GSVA) was performed to analyze all samples, and the hypoxia and ferroptosis scores for each sample were then calculated [37].

### 2.3. Coexpression Network Construction

The TCGA-LUAD transcriptome data were selected for the establishment of gene coexpression networks using the *R* package WGCNA [38]. Hypoxia and ferroptosis scores were used as phenotypic characteristics. To assess the correlation of all samples in the TCGA-LUAD database, we performed a cluster analysis to ensure the completeness of the samples. As shown in Supplementary Figure 1(a), TCGA-44-3917-01A-01R-A278-07 was identified as an outlier and therefore was not included in this section of the subsequent analysis. During the network construction phase, the soft thresholding power *β* was obtained above 0.90, with the fit index of the scale-free topology. A dendrogram of all genes was established using the dissimilarity measure to group them together (1-TOM) (Supplementary Figure 1(b)). We set 30 as the minimum module size, and modules with similar gene expressions were clustered and displayed in a tree diagram with color assignments according to the dynamic tree-cutting algorithm. To identify the modules associated with hypoxia and ferroptosis scores, a heatmap of module-feature relationships with correlation coefficients and *P*-values was drawn. Modules that had a strong dependency on both scores were identified as modules of interest, and the genes in these modules of interest were defined as hub genes.

### 2.4. Analysis of Differentially Expressed Genes (DEGs)

Transcriptome data from 53 normal and 539 LUAD samples were used as the foundation for comparison to analyze genes expressed differently. DEGs were analyzed using the *R* package limma, with significance criteria of |log_2_ fold change (FC)| > 1 and *P* < 0.05 as significance thresholds.

### 2.5. Overlap Analysis

Overlap analysis was used to identify common genes between the identified hub genes and DEGs, which were defined as DE-hypoxia and ferroptosis score-related genes for the subsequent analysis.

### 2.6. Functional Enrichment

Using Metascape (https://metascape.org) [39], the researchers were able to confirm the functional enrichment of DE-hypoxia and ferroptosis score-related genes in this investigation. *P* < 0.05 was the significant threshold.

The active signaling was analyzed using gene set variation analysis (GSVA) [37], which could compute sample gene set enrichment using a Kolmogorov–Smirnov-like rank statistical analysis. In the present study, a GSVA assessment was used to establish the *t* score and to allocate 50 hallmark gene signature activity conditions to the groups with high or low risk. At last, we compared the values. The cutoff value was set to |*t*| > 2.

### 2.7. Identification and Establishment of the Gene Signature

TCGA's 476 LUAD cases were randomly separated into two groups by using a 7 : 3 split ratio. One group was used for training and another one for testing. The DE-hypoxia and ferroptosis score-interrelated genes that are related to OS were discovered using the TCGA training dataset. The characteristics related to LUAD prognosis were determined by using univariate Cox regression (UCR) analysis. *P* < 0.05 was considered as significant. After the LASSO-penalized Cox regression (LCR) analysis of the proposed predictive panels, 10-fold cross-validation was used. Risk scores can be generated by using prognostic gene signature. In accordance with the appropriate cutoff of the risk score, patients from the TCGA training and TCGA test sets, as well as GSE31210, were split into two groups. The AUC of the ROC curve and Kaplan–Meier (KM) analyses were applied. External validation was performed using the GSE31210 dataset.

### 2.8. Nomogram Construction and Validation

To identify whether the risk model can be influenced by clinical factors, UCR and MCR analysis together with the survival *R* package were performed. Following those analyses, a nomogram was obtained using MCR coefficients of the risk score and clinical variables in the TCGA cohort, which was then analyzed. It was necessary to create calibration curves to determine whether OS for one, three, or five years were consistent with the actual findings (bootstrap-based 1000 iterations resampling validations). We developed these analyses based on the *R* package rms.

### 2.9. Immune Cells Infiltration (ICI)

The ICI into two groups was determined using the ssGSEA method and the *R* software [40]. The analysis considered only values with a *P* < 0.05. The violin diagrams used to illustrate the changes in ICI between separate categories were drawn with the ggplot2 package.

### 2.10. Patients and Tissue Samples

We performed experimental validation on specimens from five LUAD patients who underwent surgery at Yan'an Affiliated Hospital, Kunming Medical University, to validate 14 hypoxia and ferroptosis score-related signature expression status in LUAD and adjacent normal tissues (ANT). ANTs were used as controls. The institutional and national research committees were followed in the conduct of all procedures, as well as the Helsinki Declaration. The hospital's Ethics Committee gave its approval before any of the operations could be carried out (Permit No. 2017-014-01). All of the patients who took part in the trial gave their informed permission before participation.

### 2.11. RNA Isolation and qRT-PCR

The 20 tissues were dissociated using TRIzol Reagent (Life Technologies); then, total RNA was collected and determined the concentration using NanoDrop 2000FC-3100 (Thermo Fisher Scientific). Prior to performing qRT-PCR, the SureScript-First-strand-cDNA-synthesis kit (GeneCopoeia) was used to reverse transcription reaction. The qRT-PCR reaction was as follows: 4 *μ*L of reverse transcription product, 2 *μ*L of 5 × BlazeTaq qPCR Mix (GeneCopoeia, Guangzhou, China), 0.5 *μ*L primers, and 3 *μ*L of ddH_2_O. A BIO-RAD CFX96 TouchTM PCR detection system (Bio-Rad Laboratories, Inc., USA) was utilized to perform the PCR reaction as follows: 95°C for 30 s, 40 cycles of incubation at 95°C for 10 s, 60°C for 20 s, and 72°C for 30 s. In this study, the primers used were synthesized by Servicebio (Servicebio Co., Ltd., Guangzhou, China) as follows: for KLK11:5′-AGGGCTTGTAGGGGGAGA-3′, 5′-TGGGGAGGCTGTTGTTGA-3′; for MAPK4: 5′-TCAAGATTGGGGATTTCG-3′, 5′-TATGGGCTCATGTAGGGG-3′; for ITGA2: 5′-ATCAGGCGTCTCTCAGTTTC-3′, 5′-GTTTTCTTCTTGGCTTTCAC-3′; for WFDC2: 5′-CAGGCACAGGAGCAGAGAAG-3′, 5′-TCATTGGGCAGAGAGCAGAA-3′; for TNS4: 5′-GGGGCTTTTGTCATAAGGG-3′, 5′-TTTGAAGTGGACCACGGTG-3′; for LAMA3: 5′-GGTTTTGGTCCGTGTTCT-3′, 5′-ACTGCCCCGTCATCTCTT-3′; for SMAD9: 5′-GGAGATGAAGAGGAAAAGTGG-3′, 5′-GAAAGAGTCAGGATAGGTGGC-3′. GAPDH was chosen to be an internal control, and the 2^−ΔΔCt^ method was used to calculate the hub genes' relative expression level [41]. The experiment was repeated in triplicate on independent occasions.

### 2.12. Statistical Analysis

Statistical analysis was performed using *R* 3.4.3 and GraphPad Prism V9. *P*-value <0.05 means significant difference. To evaluate survival, both UCR and MCR analyzes were used. Both hazard ratios (HRs) and 95 percent CIs were reckoned to identify genes that were related to OS. Paired *t*-tests were performed for statistical differences in this study using GraphPad Prism V9.

## 3. Results

### 3.1. Filtering for Hypoxia Score- and Ferroptosis Score-Related Genes in TCGA-LUAD Database

A total of 200 hypoxia-interrelated and 259 ferroptosis-interrelated genes were gained from MSigDB and FerrDB, respectively. The expression conditions of these genes in 593 samples (normal: 59, LUAD: 534) were then matched and utilized as the basis for ssGSEA, which aimed to derive the hypoxia and ferroptosis scores in TCGA database. The ssGSEA outputs for the detailed score results are shown in Supplementary Table 1.

**Figure 1:**
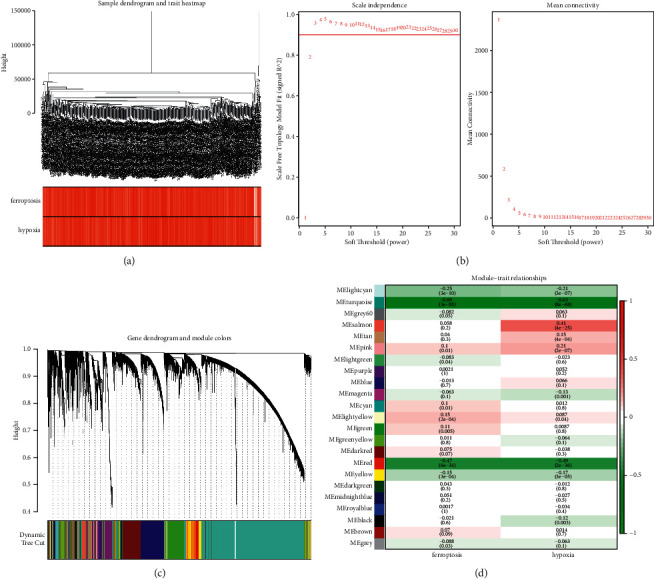
(a) Sample-clustering dendrogram with feature heatmap. (b) Network topology analysis with different soft threshold power. (c) Cluster dendrograms of genes based on topological overlap of dissimilarities, and module colors were assigned. (d) Heatmap showing the relationship between gene modules and phenotypic traits. Each row and column correspond to a module *e*-gene and a trait. The correlation coefficient in each cell represents the same relationship with heatmap in decreasing magnitude from red to green. The number in parentheses in each cell represents the correlation *P*-value.

WGCNA was performed by applying the obtained hypoxia and ferroptosis scores as phenotypic data. After excluding the outlier samples, we constructed a sample-clustering tree (Figure 1(a)). Herein, a scale-free network was built when *β* = 3, which was defined as a soft threshold parameter (Figure 1(b)). Finally, 23 modules were identified according to the dynamic tree-cutting algorithm and were labeled with different colors (Figure 1(c)). The turquoise module was most irrelevant to ferroptosis score (cor = −0.69, *P*=3*e* − 10) and hypoxia score (cor = −0.63, *P*=8*e* − 68), whereas the red module correlated more strongly with both ferroptosis score (cor of −0.47, *P*=6*e* − 34) and hypoxia score (cor = −0.49, *P*=2*e* − 36) (Figure 1(d)). Therefore, these two models were identified as the modules of interest. Collectively, 8314 genes (Supplementary Table 2) and 660 genes (Supplementary Table 3) were identified as hub genes and considered as hypoxia and ferroptosis score-related genes for subsequent analysis.

### 3.2. Identification of LUAD-Related DEGs

**Figure 2:**
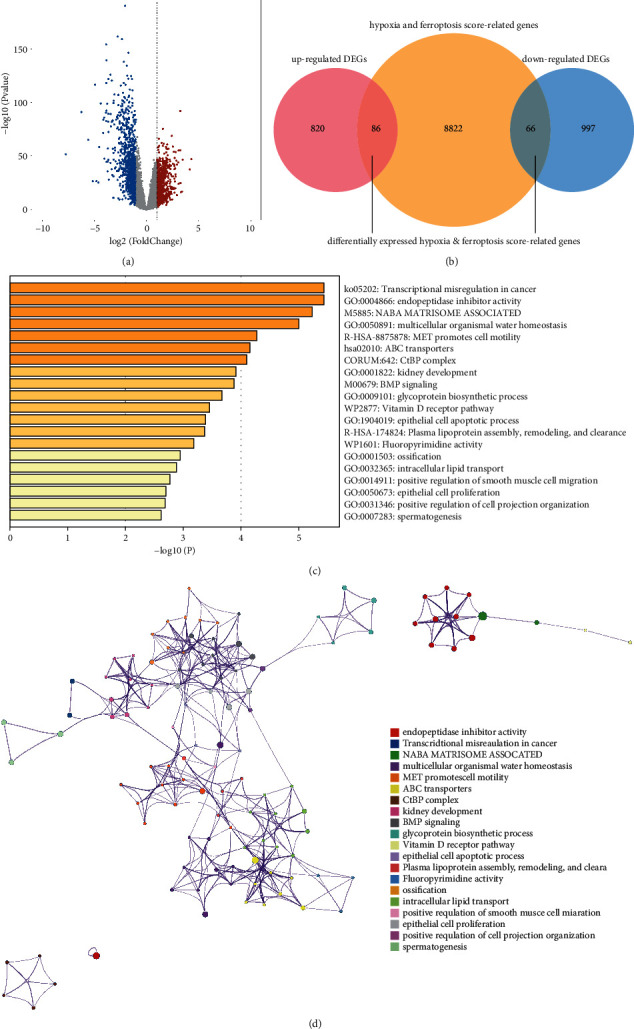
(a) Volcano map of significant DEGs. Red spots: upregulated genes; blue spots: downregulated genes; gray: genes with no change in expression. (b) Venn diagram showing the repetitious genes of DEGs and WGCNA. (c, d) Function analysis of DE-hypoxia and ferroptosis score-related genes using Metascape.

Differential expression analysis was used to acquire transcriptome data from TCGA (59 normal and 534 LUAD samples), which was produced using the *R* program limma. When LUAD samples were compared to normal samples, a total of 1,969 eligible DEGs were obtained, among which 906 were significantly increased in LUAD samples, and 1,063 were significantly decreased (Figure 2(a); Supplementary Table 4).

### 3.3. Analysis of DE-Hypoxia and Ferroptosis Score-Related Genes

Based on the overlap analysis, we identified 152 common genes from the list of 8,974 hypoxia and ferroptosis score-related genes and the list of 1,969 LUAD-related DEGs, which were defined as DE-hypoxia and ferroptosis score-related genes (Figure 2(b)). In LUAD, 86 of these genes were upregulated, while 66 were inversed. The expression patterns of DE-hypoxia and ferroptosis score-related genes in the TCGA-LUAD database are described in Supplementary Table 5.

Functional annotations obtained from Metascape indicated that DE-hypoxia and ferroptosis score-related genes were mainly augmented in “transcriptional misregulation in cancer,” “spermatogenesis,” and “positive regulation of cell projection organization” (Figures 2(c) and 2(d)).

### 3.4. Establishment of the Hypoxia and Ferroptosis Score-Related Signature

**Figure 3:**
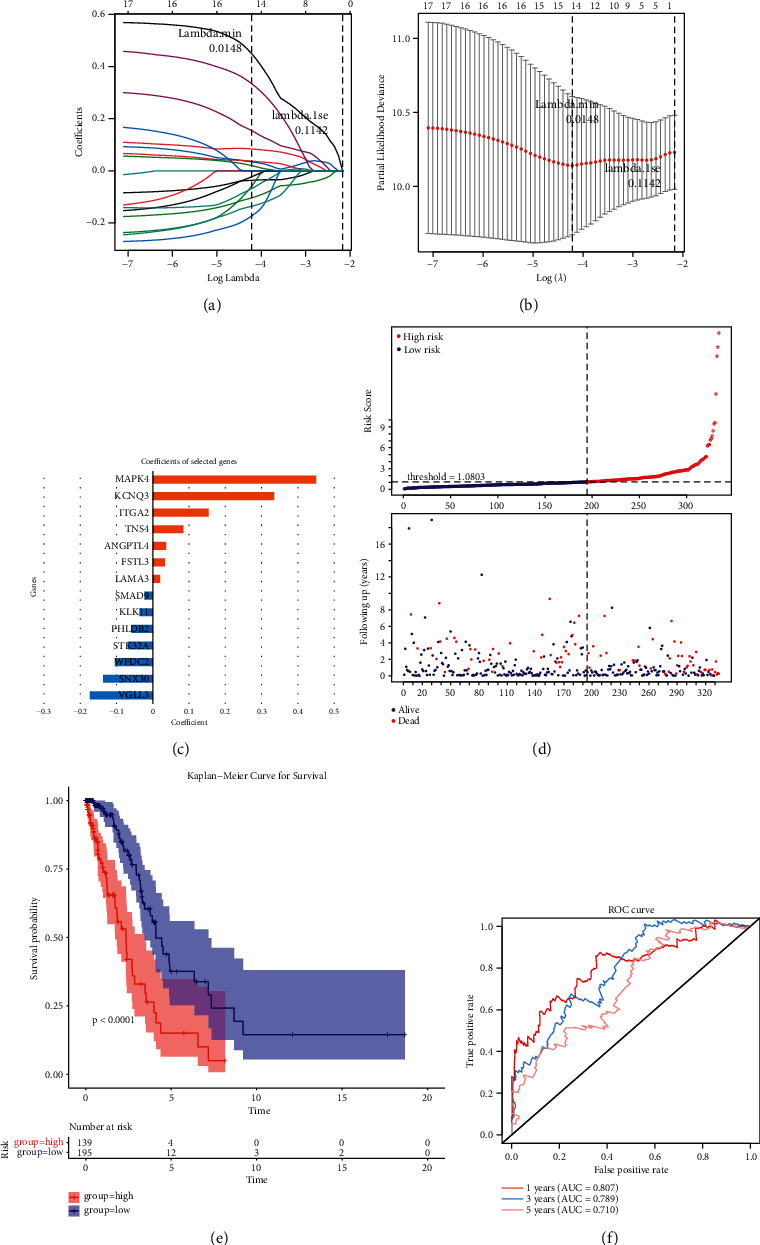
(a–c) The LCR was used to figure out the lowest criteria (a, b) and coefficients (c). (d) Allocations of risk scores (based on the hypoxia and ferroptosis score-related prognostic signature); (e) *K*-*M* survival curves. (f) Hypoxia and ferroptosis score-related signature can be utilized to predict OS in the TCGA training set according to ROC curves.

In the TCGA training set (*n* = 334), the association of the 152 identified DE-hypoxia and ferroptosis score-related genes with survival in LUAD patients was analyzed using UCR. As shown in Table 1, only 17 of the 152 genes met the set significance threshold of *P* < 0.05. The HRs of *SMAD9*, *SNX30*, *STK32A*, *WFDC2*, *KLK11*, and *CTD*.*2589M5*.*4* were all <1, indicating that they were potential protective factors for LUAD. In contrast, *ANGPTL4*, *LAMA3*, *VGLL3*, *ITGA2*, *TNS4*, *KCNQ3*, *PHLDB2*, *FAM83A*.*AS1*, *SLC16A3*, *FSTL3*, and *MAPK4*, all with HR >1, were possible oncogenes. We performed LLR analysis based on 17 variables in the TCGA training set (Figures 3(a) and 3(b)) to obtain the best genes for constructing the prognostic signature. Ultimately, the hypoxia and ferroptosis score-related signature involved 14 genes: *MAPK4*, *TNS4*, *WFDC2*, *FSTL3*, *ITGA2*, *KLK11*, *PHLDB2*, *VGLL3*, *SNX30*, *KCNQ3*, *SMAD9*, *ANGPTL4*, *LAMA3*, and *STK32A*. We estimated the risk score of each individual in TCGA set based on the coefficient of each gene (Figure 3(c); Supplementary Table 6).

**Figure 4:**
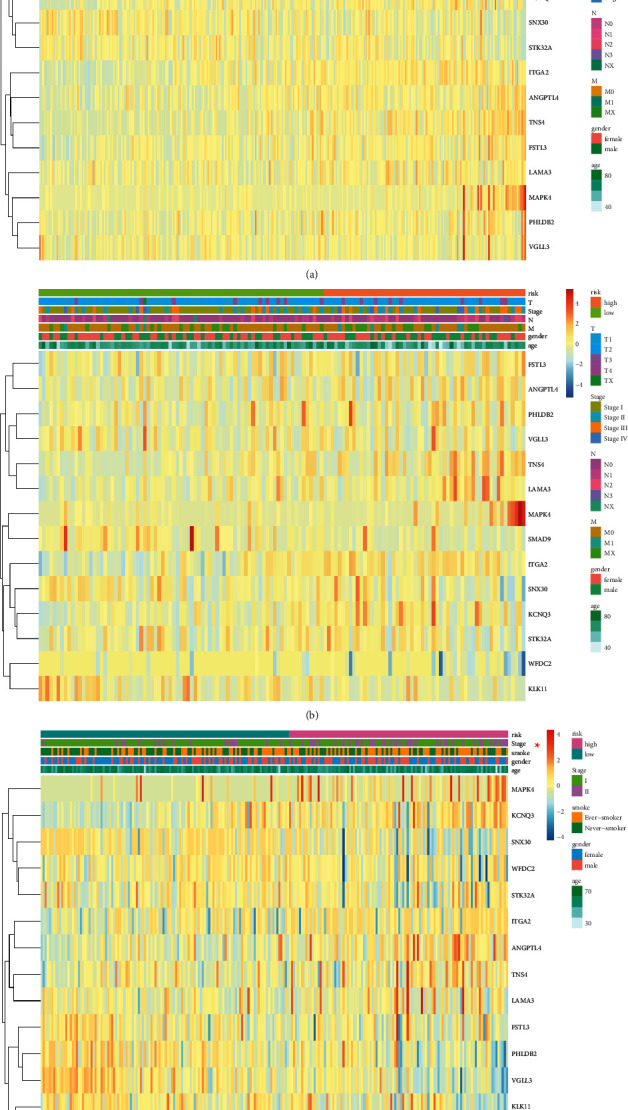
Heatmap of the relationship between the expression of 14 genes associated with hypoxia and ferroptosis scores and clinicopathological features in the (a) TCGA training, (b) TCGA test, and (c) GSE31210 dataset.

The patients with LUAD in the TCGA training set were separated into two groups with the cutoff value at 1.0803 (Supplementary Table 7). The allocation of risk scores is shown in Figure 3(d). Association analyses revealed a significant correlation (*P* < 0.05) between the *T* stage and various risk groups in the TCGA training set (Table 2). A significant association between a high-risk score and a poor outcome (*P* < 0.0001; Figure 3(e)) was shown in the Kaplan–Meier survival curves. ROC curves indicated that hypoxia and ferroptosis score-related signature could be used to predict OS in the TCGA training group (Figure 3(f)). Additionally, the heatmap indicated that the expression levels of *KCNQ3*, *ITGA2*, *ANGPTL4*, *TNS4*, *FSTL3*, *LAMA3*, *MAPK4*, *PHLDB2*, and *VGLL3* were upregulated with enhancing risk score, but the expression levels of *KLK11*, *SMAD9*, *WFDC2*, *SNX30*, and *STK32A* were reduced. Additionally, in individuals with LUAD, *T* stages are also relevant to these genes expression (Figure 4).

### 3.5. Validation Prognostic Signature with 14 Genes

**Figure 5:**
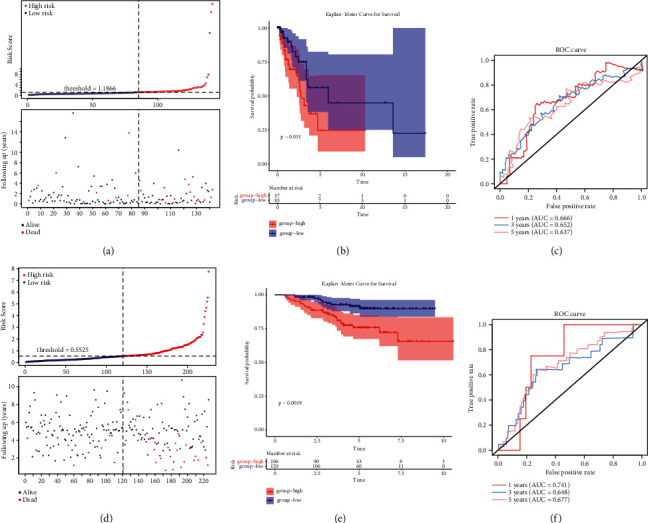
(a, d) Allocations of risk scores. (b), (e) The *K*-*M* survival curves showed that a high-risk score was related to less OS. Hypoxia and ferroptosis score-related signature can be utilized to predict OS in the (c) TCGA test and (d) GSE31210 dataset according to ROC curves.

We used the same algorithm to compute the risk scores for the patients in the TCGA test cohort (*n* = 142; Supplementary Table 8) and the GSE31210 dataset (*n* = 226; Supplementary Table 9). According to cutoff values determined for each dataset, patients were separated into two risk groups. The results corroborated those from the TCGA training set. Figures 5(a) and 5(d) indicated that mortality status was more concentrated in the domain of high-risk scores. In both validation datasets, Figures 5(b) and 5(e) showed that high-risk patients had a considerably poorer outcome. In both datasets, the 14-gene prognostic signature performed well. The risk scores of AUCs for 1-, 3-, and 5-year OS predictions were 0.666, 0.652, and 0.637 in the TCGA test set, respectively (Figure 5(c)), while the AUCs of the 14-gene signature were 0.741, 0.648, and 0.677 for the three kinds of OS predictions, respectively, using the GSE31210 dataset (Figure 5(f)). The distribution of LUAD patients with different groups according to each clinical feature in the TCGA test set is shown in Table 3. Association studies revealed a significant (*P* < 0.01) correlation between the clinical stage and different risk groups in the GSE31210 dataset (Table 4).

### 3.6. Correlation Analysis of Risk Score with Clinical Characteristics of LUAD

**Figure 6:**
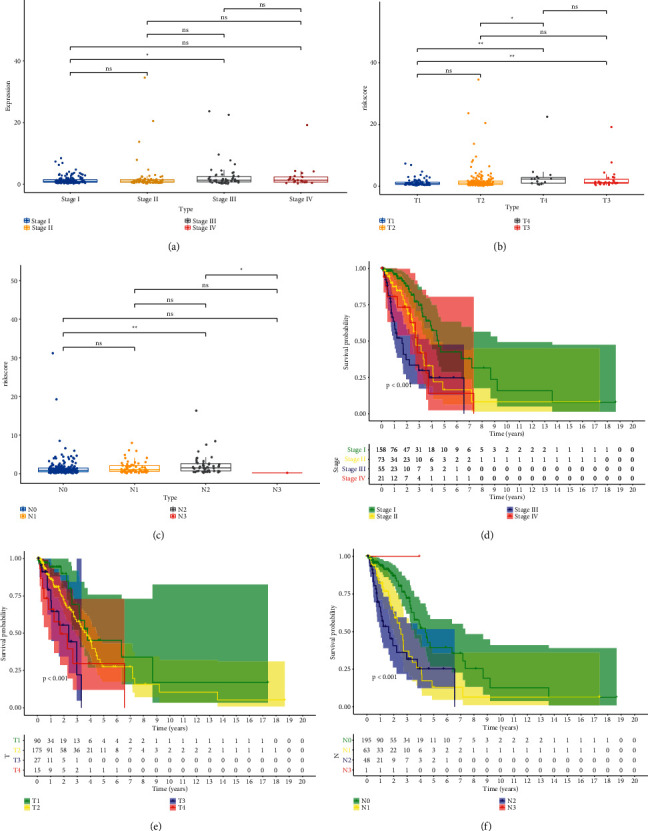
Wilcoxon analysis of the differing risk score distributions among various (a) stages, (b) *T*  stages, and (c) *N* stages in the TCGA-LUAD cohort. The *K*-*M* survival curves of patients with different (d) stages, (e) *T* stages, and (f) *N* stages. ^*∗*^*P* < 0.05, ^*∗∗*^*P* < 0.01, and ^*∗∗∗*^*P* < 0.001.

We observed the allocation of patient risk scores according to different clinical characteristics. Interestingly, the distribution of patient risk scores was highly related to the stages of the patients. Risk scores in patients in stage III were increased compared to those in stage I (*P* < 0.05; Figure 6(a)). In terms of the *T* stage (Figure 6(b)), patients with LUAD in *T*4 had the highest risk scores, which have a significant difference in *T*1 and *T*2, but comparable to *T*3. Patients with LUAD in the *T*3 stage had slightly higher risk scores than those in the *T*1 stage (*P* < 0.01); in the *N* stage (Figure 6(c)), patients in the *N*2 stage had higher risk scores than those in the *N*0 stage (*P* < 0.01). Although the risk score in stage *N*3 was lower than in stage *N*2 (*P* < 0.05), the sample size in stage *N*3 was too small to be considered valid. Subsequently, the impact of clinical characteristics on the OS in LUAD patients was investigated using KM survival analysis. Specifically, in the stratified analysis of stage (Figure 6(d)), patients with a lower stage are more likely to have a better prognosis, which showed the same trend with distribution of risk score levels. In the stratified analysis of the *T* stage (Figure 6(e)), the *T*1 stage had a better OS, whereas *T*3 and *T*4 stages exhibited a poor prognosis. The worst prognosis in LUAD patients in the *T*4 stage was consistent with the previous result that patients with *T*4 stage had the highest risk score. In terms of the *N* stage (Figure 6(f)), the *N*3 stage contained only one LUAD sample, and therefore, its impact on patient prognosis was ignored. Patients with the *N*0 stage had the longest survival time compared with those with the *N*2 stage who had the shortest survival time. The allocation of risk scores and stratified prognosis according to other clinical characteristics, including age, sex, and *M* stage, are detailed in Supplementary Figure 2.

### 3.7. Subgroup Analysis of the Prognostic Signature

**Figure 7:**
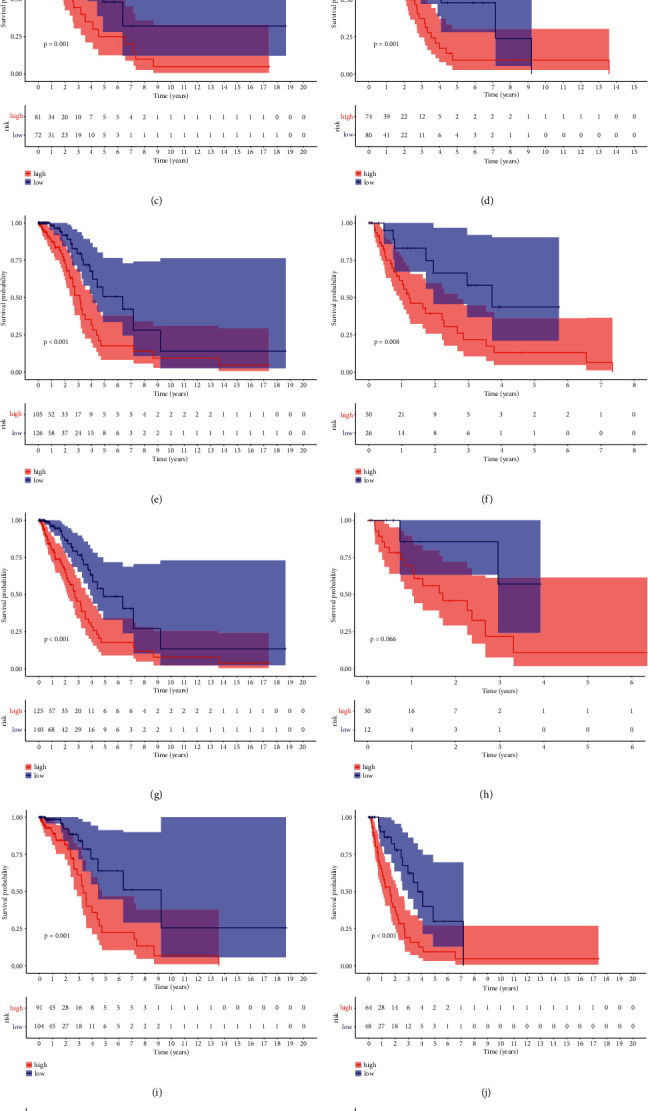
*K*-*M* survival analysis of the fourteen-gene risk score level in subgroups: (a) younger than 60 years old and older than 60 years old, (b) male and female, (c) stages I-II and stages III-IV, (d) T1 2 stage and T3-4 stage, (e) *N*0 stage and *N*+ stage, and (f) *M*0 stage and *M*1 stage.

After establishing a correlation between hypoxia and ferroptosis score-related gene signatures and the aforementioned clinicopathological traits, we aimed to measure whether our model's prognostic efficacy can be utilized for clinical factors. Five patients were separated according to the indicated subgroups, and then data stratification was executed according to age, sex, pathological tumor stage, pathological *T* stage, pathological *N* stage, and pathological *M* stage. The hypoxia and ferroptosis score-related gene signature was able to differentiate between prognoses in all subgroups except for *T*3-*T*4 and *M*1 features, implying a clinically and statistically significant prognostic value (Figure 7).

### 3.8. Independent Prognostic Role of Risk Scores

**Figure 8:**
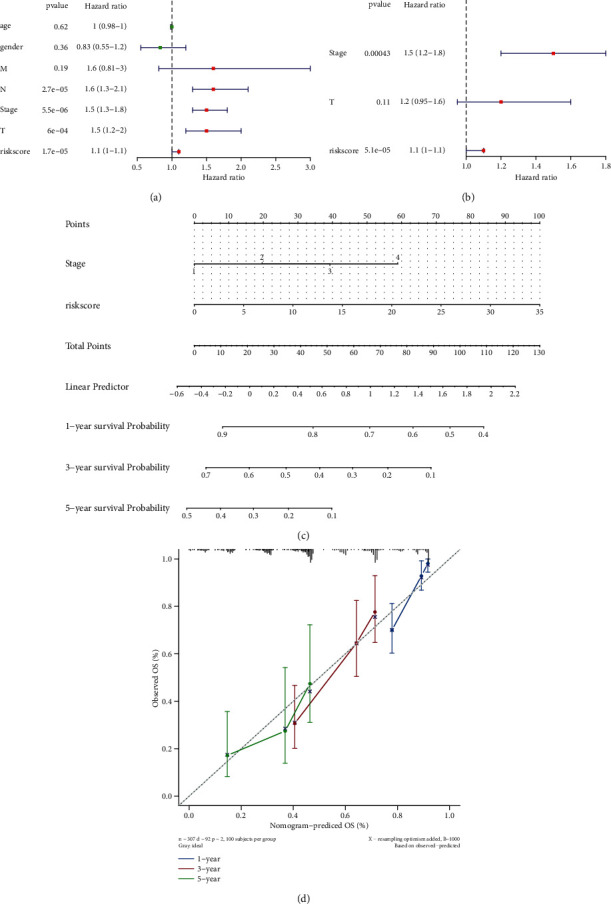
(a) Forrest plot of UCR analysis in LUAD. (b) Forrest plot of MCR analysis in LUAD. (c) A prognostic nomogram predicting OS of LUAD. (d) Calibration plots of the nomogram for predicting the OS in the TCGA-LUAD dataset.

We investigated whether the risk score could be the only prognostic factor in LUAD patients using UCR and MCR. Based on the data from in TCGA set, UCR analyses showed that the risk score, stage, *T* stage, and *N* stage were significantly related to LUAD prognosis (Figure 8(a)). Subsequently, the above-mentioned variables (*P* < 0.05) were subjected to MCR analysis. The results identified hypoxia and ferroptosis score-related gene signature (risk score) and stage as two independent prognostic factors predicting prognosis in LUAD patients (Figure 8(b)).

LUAD patients' OS were predicted using a compound nomogram incorporating the risk score and stage. This approach was developed to provide a more accurate prediction tool for clinical practice (Figure 8(c)). It was evident from the calibration plots that the prognostic nomogram model accurately predicted patient survival with only a slight divergence from the actual outcomes (Figure 8(d)).

### 3.9. Differences in Hallmark Gene Sets between Two Group Patients

**Figure 9:**
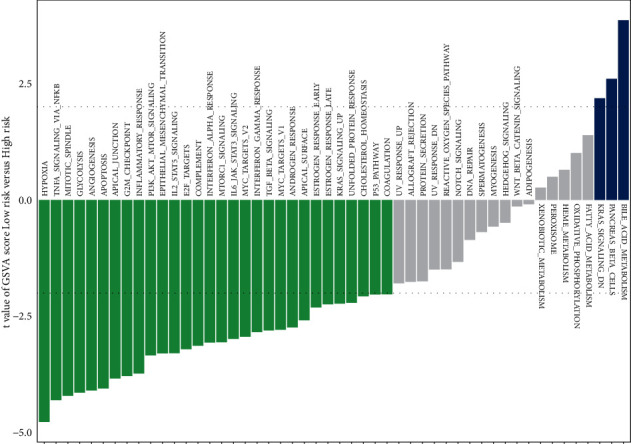
Gene set variation analysis. Differences in hallmark gene set activities scored by GSVA between two groups. *T* values are figured out using a linear model and the |*t*| > 2 as a cutoff value.

According to the results of the analysis of signature gene sets, signaling pathways converging in numerous biological processes were found to vary in two groups. Notably, hypoxia, TNF*α* signaling via NF-*κ*B, mitotic spindle, and glycolysis were decreased in the low-risk group. On the other hand, the other group was preferentially associated with bile acid metabolism, pancreatic beta cells, and KRAS signaling (Figure 9 and Supplementary Table 10).

### 3.10. TME Infiltration Pattern of LUAD Based on Risk Score

**Figure 10:**
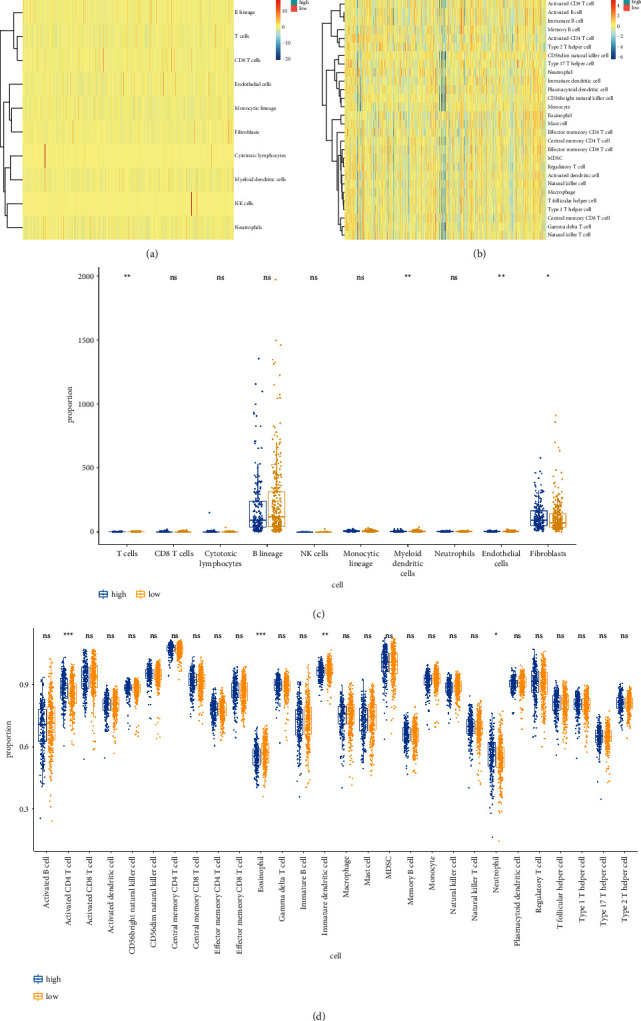
(a, b) Heatmap illustrating the distributions of immune cell subsets, fibroblasts, and endothelial cells assessed via MCP-counter (a) and ssGSEA (b) algorithms in the TCGA-LUAD cohort. (c, d) Wilcoxon analysis of the differing TME subtype distributions between two groups in the TCGA-LUAD cohort. ^*∗*^*P* < 0.05, ^*∗∗*^*P* < 0.01, and ^*∗∗∗*^*P* < 0.001.

The ssGSEA algorithms were used on the data to investigate how risk scores affect TME components. As the results of heatmaps and Wilcoxon tests performed on TCGA-LUAD datasets, the infiltration of several TME contents, such as eosinophils and immature dendritic cells, was increased in the less-risk group, whereas the ICI of activated CD4 *T* cells and others was more in the other group, as depicted in Figure 10.

### 3.11. Validation of Seven Selected Prognostic Genes Based on qRT-PCR

**Figure 11:**
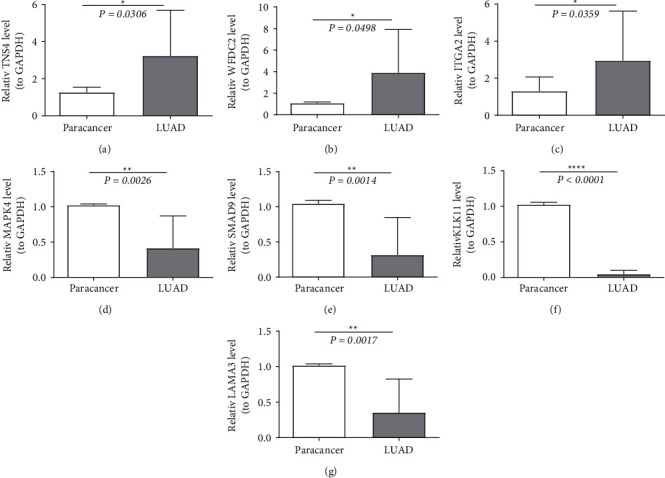
The high expression of TNS4 (a), WFDC2 (b), and ITGA2 (c) and the low expression of MAPK4 (d), SMAD9 (e), KLK11 (f), and LAMA3 (g) in LUAD tissues were confirmed compared to the paracancerous tissues.

According to the expression profiles of the identified DEGs (Supplementary Table 5), *TNS4*, *WFDC2*, and *ITGA2* were revealed to be all highly expressed, while *MAPK4*, *SMAD9*, *KLK11*, and *LAMA3* were all downregulated in LUAD samples from the TCGA dataset. As shown in Figure 11, the high expression of *TNS4*, *WFDC2*, and *ITGA2* and the low expression of *MAPK4*, *SMAD9*, *KLK11*, and *LAMA3* in LUAD tissues (*n* = 10) were confirmed compared with the expression levels in the ANTs (*n* = 10).

## 4. Discussion

As well known, lung cancer is one of the general forms of malignancy globally. Nearly 80% of lung cancer patients have NSCLC, and nearly 50% have LUAD [42]. LUAD is a malignant tumor that affects the lungs and has a poor prognosis [43]. Although there have been breakthroughs in the treatment of patients with LUAD, the OS rate in these individuals remains low.

Ferroptosis is a particular kind of programmed cell death [17]. Ferroptosis-related research on lung cancer has mostly focused on the identification of related biomarkers that could induce ferroptosis [16, 44–46]. Hypoxia is also related to high proliferation rates in tumor cells [47]. Tumor hypoxia has a broad range of consequences, affecting a variety of biological systems, including metabolic changes, angiogenesis, and metastasis [48–50]. Numerous hypoxia-associated genes are associated with lung adenocarcinoma [51, 52]. However, no high-throughput research has been conducted to date to explore the possible prognostic value of them in LUAD.

Here, the ferroptosis and hypoxia *Z*-scores of each sample were estimated as clinical features based on the expression of ferroptosis and hypoxia-related genes identified in each sample, respectively. We obtained 23 modules, and the turquoise module showed no relationship with ferroptosis scores (cor = −0.69, *P*=3*e* − 10) and hypoxia scores (cor = −0.63, *P*=8*e* − 68), while the red module correlated more strongly with both scoring phenotypes, with ferroptosis score and hypoxia score. We then identified 152 common genes from the list of 8,974 hypoxia and ferroptosis score-related genes and 1,969 LUAD-related DEGs, which were defined as DE-hypoxia and ferroptosis score-related genes, respectively.

Functional annotations obtained from Metascape indicated that DE-hypoxia and ferroptosis score-related genes were mainly enriched in “transcriptional misregulation in cancer,” “endopeptidase inhibitor activity,” and “positive regulation of cell projection organization.” Overexpression of oncogenic transcription factors has been proven in recent research to change cells' core autoregulatory circuitry, which has long been recognized to induce tumorigenesis due to mutations in transcription factor genes [53]. Therefore, it is possible to intervene in this pathway to prevent the development of LUAD.

Of the 152 DE-hypoxia and ferroptosis score-related genes, 7.3% (17/152) were associated with prognosis in univariate Cox analysis. In addition, univariate Cox analysis identified six genes as protective markers and 11 genes as risk factors for patients with LUAD. Fourteen genes were identified using LASSO Cox regression (*MAPK4*, *TNS4*, *WFDC2*, *FSTL3*, *ITGA2*, *KLK11*, *PHLDB2*, *VGLL3*, *SNX30*, *KCNQ3*, *SMAD9*, *ANGPTL4*, *LAMA3*, and *STK32A*) to construct prognostic-related gene signatures and develop prognostic models to classify LUAD patients into two groups with various risks. Herein, we suggested that lower-risk patients seem to live longer. Additionally, we built a nomogram using MCR analysis and proved its predictive ability using ROC curves, calibration plots, and decision curves.

MAPK4 overexpression promotes LUAD progression [54]. Tensin 4 (*TNS4*) is involved in MET-induced cell motility and is connected to the GPCR signaling pathway. According to one study, increased *TNS4* expression leads to poor treatment outcomes in gastric cancer patients [55]. *WFDC2* is upregulated in lung cancer [56–58] and has thus recognized the clinical application of *WFDC2* as a serum tumor marker in the early diagnosis and efficacy monitoring of lung cancer [59]. In addition, in a study of individuals with LUAD, Song et al. [34] reported that *WFDC2* was substantially related to the TNM stage of LUAD and prognosis of patients. Recent studies have reported substantial overexpression of *FSTL3* in a subset of cancers [60–62]. Additionally, in patients with NSCLC and thyroid carcinoma, *FSLT3* expression is substantially linked to lymph node metastasis and poor prognosis [60, 61]. *ITGA2* overexpression is essential for tumor development, metastasis, and motility, and this molecule triggers the overexpression of the STAT3 signaling pathway, thus promoting tumor progression [63]. KLK11 protein is expressed more in NSCLC serum, although *KLK11* mRNA levels are lower in cancerous lung tissues than in ANTs [64]. Leakage of these secreted proteins into the systemic circulation due to disruption of lung structure during angiogenesis or development may be the reason for this discrepancy between low mRNA levels and elevated serum protein levels in lung cancer [65]. It has been well studied that PHLDB2 is linked to a variety of malignancies [66, 67]. PHLDB2's primary role is to control migration through interacting with the transcription factors CLASPS, prickle 1, and liprin 1 [68, 69]. According to Ge et al., patients with lower PHLDB2 expression have a better prognosis [70]. VGLL3 is a unique Ets1 interacting partner that inhibits adipocyte differentiation and controls trigeminal nerve development [71]. VGLL3 acts as a coactivator of mammalian toxicity equivalency factors and is implicated in many kinds of cancers, including breast, colon, and lung cancers [72, 73]. Methylation, phosphorylation [74], and dephosphorylation of SMAD9 may function in the progression of lung cancer [75]. Tumor cell-derived human angiopoietin-like protein 4 (ANGPTL4) has been shown to disrupt vascular endothelial cell connections, enhance pulmonary capillary permeability, and facilitate tumor cell protrusion through the vascular endothelium, which is involved in lung cancer [76]. Through the synergistic action of AP-1 binding sites [77], the epithelial enhancer mediates the production of laminin subunit alpha 3 (LAMA3), which is associated with tumor progression. Xu et al. [78] reported that it was discovered that the inhibition of *LINC00628* decreased LUAD cell proliferation and drug resistance by lowering the methylation of the *LAMA3* promoter. STK32A is important in cellular balance and transcription factor phosphorylation, together with cell cycle regulation, and its overexpression leads to enhanced NSCLC cell progression, as well as enhanced NF-*κ*B p65 phosphorylation and inhibition of apoptosis [79]. *SNX30* encodes sorted nexin-30 protein, a member of the sorted nexin, which a large class of proteins localized in the cytoplasm with membrane-bound potential via a phospholipid-binding domain [80]. *KCNQ3* encodes a protein that regulates neuronal excitability, and *GCSH* encodes a mitochondrial protein that forms the glycine cleavage system [81]. However, there is a lack of research on the mechanisms of action of these two genes in cancer.

Following this assessment, KM survival studies demonstrated that the 14 prognosis-associated genes may have a contribution to the initiation and development of LUAD in certain individuals. It came as a surprise to observe that risk scores for the 14-gene prognostic profile were shown to be strongly correlated with the OS in LUAD patients in two cohorts split by the TCGA and one GEO validation cohort. We discovered that modulation of the prognostic gene profile was linked with the LUAD survival models (*T*, *N*, *M*, stage, sex, and age) in our study. Furthermore, the nomogram of independent risk factors, which included risk score models, had a good predictive value and might assist clinicians in making optimum treatment choices to enhance the OS rates of patients with LUAD in the future. These results suggest that hypoxia- and ferroptosis-related genes were indispensable in the construction of prognostic models for LUAD development and that they may have the potential to act as OS biomarkers.

Our findings suggested that the signaling pathways that converge in various biological processes differ between two groups, and the hypoxia, TNF*α*, signaling via NF-*κ*B, mitotic spindle, and glycolysis were significantly downregulated in the less-risk group. Additionally, 14 prognosis-related genes in LUAD, including one hypoxia-related gene, *ANGPTL4*, were significantly expressed in the tumor tissues. This finding reflects the dependence of LUAD on hypoxia and the heterogeneity of hypoxia responses in the low- and high-risk groups. Hypoxia heterogeneity indicates its involvement in promoting a phenotypic variety of cancer cells in the TME, which promotes metastasis and therapeutic resistance. Li et al. [82] demonstrated that suppressing NLRP2 boosted cell proliferation through NF-*κ*B signaling activation, thus resulting in an EMT phenotype in LUAD cells. Therefore, the regulatory pathways involved in NF-*κ*B also function in the progression of LUAD. The evidence implies that LUAD pathogenesis is a complicated biological process involving multiple genes. Apart from that, dysregulation of multiple genes may contribute to the progression of LUAD by a variety of distinct processes. The differences in GSVA signatures and prognostic genes between the two groups have the potential to be explored in a more in-depth study. These discoveries may, in general, open new avenues of investigation of additional molecular mechanisms of LUAD for academics and physicians.

Significant differences in immune infiltrating cell types between two groups were shown in this study. Interestingly, the enrichment fraction of activated CD4 *T* cells and neutrophils was enhanced in the high-risk group, whereas the enrichment fraction of eosinophil and immature dendritic cells was found in the low-risk group. Immune cells, neutrophils that infiltrate tumor tissue, called TANs, also play a role in antitumor immunity. TANs stimulate *T*  cell responses in lung cancer rather than have an immunosuppressive effect [83]. In LUAD, overexpression of bridging granule genes is associated with a significant enhancement in infiltration of activated CD4 and CD8 *T* cells [84]. We hypothesize that the inflammatory response induced by immune cells may function in accelerating tumor cell mutations, which in turn may affect patient prognosis. The specific mechanisms by which the tumor immune microenvironment affects prognosis remain to be explored.

Here, a prognostic model of LUAD with general applicability was successfully developed and validated based on hypoxia and ferroptosis. In addition, we performed experiments to validate the 14 molecules in the model. Of these, seven molecules were validated by qRT-PCR to be significantly different between tumor and paracancerous tissues. However, our study has some limitations. Due to the lack of studies on hypoxia and ferroptosis in tumors, the information provided by MSigDB and FerrDB websites may be inaccurate, as the references were manually obtained from previous studies. More studies will do to validate the roles of these fundamental prognostic genes' hypoxia- and ferroptosis regulation roles in LUAD [3]. Both cohorts (TCGA-LUAD and 1 GEO cohort) were used to construct predictive signature. This hypoxia- and ferroptosis-predictive signal may be more reliable if examined in our research center's prospective clinical trial cohort.

## 5. Conclusion

Hypoxia and ferroptosis are two major mechanisms associated with lung adenocarcinoma development. In this research, the candidate genes associated with hypoxia and ferroptosis scores were identified; as a result, we have found a 14-gene signature and developed a predictive nomogram that could accurately predict OS in individuals with LUAD. These results may be useful in facilitating the making of medical decisions and personalizing therapeutic interventions.

## Figures and Tables

**Table 1:** UCR analysis of the 152 identified DE-hypoxia and ferroptosis score-related genes explores 17 genes associated with LUAD patient survival.

ID	*z*	HR	HR. 95L	HR. 95H	*P*-value
MAPK4	4.21113029756985	1.46745295679297	1.22755540646437	1.75423298130611	2.54*E *−* *05
TNS4	4.02119788604733	1.29938376610491	1.14367045280752	1.47629779843681	5.79*E *−* *05
WFDC2	−3.79860720374219	0.811653740106402	0.728800938850636	0.903925555951741	0.000145511488661
FSTL3	3.68304906615447	1.40517139515741	1.17250302799022	1.68400985126077	0.000230460779083
FAM83A.AS1	3.19219681770566	1.38043585405413	1.13252613465791	1.68261295597717	0.00141195086371
ITGA2	3.1297146270081	1.26268315773328	1.0910870928145	1.46126626125743	0.001749761967554
KLK11	−2.89081539413551	0.817085437051864	0.712500352475736	0.937022149003037	0.003842437575419
SLC16A3	2.69059352882516	1.38307857948644	1.09206101692107	1.75164787259545	0.007132503883482
PHLDB2	2.67561048467087	1.36825660397289	1.08747876280116	1.72152891472853	0.007459328240577
VGLL3	2.48631904817044	1.24621454130366	1.04770025500438	1.48234256462046	0.012907219145633
SNX30	−2.41280356238469	0.718205289102291	0.548879152164541	0.939767588658339	0.015830348860021
KCNQ3	2.23280002150132	1.34420966442955	1.03680712106375	1.74275386929437	0.025562134874828
SMAD9	−2.20299987655253	0.660110772189965	0.456177215306126	0.955212616809054	0.02759475734267
ANGPTL4	2.17133207596654	1.15838922218355	1.01441557907091	1.32279670951033	0.029906079314469
LAMA3	2.05948665187877	1.18322521780815	1.00816336324203	1.38868557130958	0.039447642418345
CTD.2589M5.4	−1.98163055774902	0.831910477060721	0.693468992502609	0.997989887544674	0.047520604494006
STK32A	−1.97258211072512	0.789000052327525	0.623465515365524	0.998485188403512	0.048543192818321

**Table 2:** Association analysis shows that clinical characteristics correlate results with different risk groups in the TCGA training set.

Expression				
	Total (*N* = 309)	High	Low	*P*-value
		(*N* = 129)	(*N* = 180)	
Gender				
Female	165 (53.4%)	66 (51.2%)	99 (55.0%)	0.582
Male	144 (46.6%)	63 (48.8%)	81 (45.0%)	

Age (years)				
≥60	229 (74.1%)	95 (73.6%)	134 (74.4%)	0.979
<60	80 (25.9%)	34 (26.4%)	46 (25.6%)	

Pathologic stage				
Stage I	168 (54.4%)	62 (48.1%)	106 (58.9%)	0.0815
Stage II	74 (23.9%)	30 (23.3%)	44 (24.4%)	
Stage III	50 (16.2%)	28 (21.7%)	22 (12.2%)	
Stage IV	17 (5.5%)	9 (7.0%)	8 (4.4%)	

*T* stage				
*T*1	102 (33.0%)	31 (24.0%)	71 (39.4%)	0.0128
*T*2	166 (53.7%)	76 (58.9%)	90 (50.0%)	
*T*3	29 (9.4%)	13 (10.1%)	16 (8.9%)	
*T*4	10 (3.2%)	8 (6.2%)	2 (1.1%)	
*T*X	2 (0.6%)	1 (0.8%)	1 (0.6%)	

*M* stage				
*M*0	198 (64.1%)	82 (63.6%)	116 (64.4%)	0.47
*M*1	16 (5.2%)	9 (7.0%)	7 (3.9%)	
*M*X	95 (30.7%)	38 (29.5%)	57 (31.7%)	

*N* stage				
*N*0	202 (65.4%)	75 (58.1%)	127 (70.6%)	0.16
*N*1	54 (17.5%)	26 (20.2%)	28 (15.6%)	
*N*2	46 (14.9%)	25 (19.4%)	21 (11.7%)	
*N*3	1 (0.3%)	0 (0%)	1 (0.6%)	
*N*X	6 (1.9%)	3 (2.3%)	3 (1.7%)	

**Table 3:** Association analysis shows that clinical characteristics correlate results with different risk groups in the TCGA test set.

Expression				
	Total (*N* = 135)	High	Low	*P*-value
		(*N* = 56)	(*N* = 79)	

Gender				
Female	73 (54.1%)	28 (50.0%)	45 (57.0%)	0.532
Male	62 (45.9%)	28 (50.0%)	34 (43.0%)	

Age (years)				
≥60	97 (71.9%)	35 (62.5%)	62 (78.5%)	0.0658
<60	38 (28.1%)	21 (37.5%)	17 (21.5%)	

Pathologic stage				
Stage I	73 (54.1%)	25 (44.6%)	48 (60.8%)	0.159
Stage II	31 (23.0%)	13 (23.2%)	18 (22.8%)	
Stage III	23 (17.0%)	13 (23.2%)	10 (12.7%)	
Stage IV	8 (5.9%)	5 (8.9%)	3 (3.8%)	

*T* stage				
*T*1	48 (35.6%)	16 (28.6%)	32 (40.5%)	0.506
*T*2	68 (50.4%)	31 (55.4%)	37 (46.8%)	
*T*3	13 (9.6%)	7 (12.5%)	6 (7.6%)	
*T*4	5 (3.7%)	2 (3.6%)	3 (3.8%)	
*T*X	1 (0.7%)	0 (0%)	1 (1.3%)	

*M* stage				
*M*0	91 (67.4%)	35 (62.5%)	56 (70.9%)	0.381
*M*1	8 (5.9%)	5 (8.9%)	3 (3.8%)	
*M*X	36 (26.7%)	16 (28.6%)	20 (25.3%)	

*N* stage				
*N*0	86 (63.7%)	31 (55.4%)	55 (69.6%)	0.094
*N*1	27 (20.0%)	13 (23.2%)	14 (17.7%)	
*N*2	18 (13.3%)	11 (19.6%)	7 (8.9%)	
*N*3	1 (0.7%)	1 (1.8%)	0 (0%)	
*N*X	3 (2.2%)	0 (0%)	3 (3.8%)	

**Table 4:** Association analysis shows that clinical characteristics correlate results with different risk groups in the GSE31210 dataset.

Expression				
	Total	High	Low	*P*-value
	(*N* = 226)	(*N* = 106)	(*N* = 120)	

Gender				
Female	121 (53.5%)	53 (50.0%)	68 (56.7%)	0.385
Male	105 (46.5%)	53 (50.0%)	52 (43.3%)	

Age (years)				
≥60	130 (57.5%)	58 (54.7%)	72 (60.0%)	0.505
<60	96 (42.5%)	48 (45.3%)	48 (40.0%)	

Pathologic stage				
I	168 (74.3%)	65 (61.3%)	103 (85.8%)	<0.001
II	58 (25.7%)	41 (38.7%)	17 (14.2%)	

Smoke				
Ever-smoker	111 (49.1%)	58 (54.7%)	53 (44.2%)	0.147
Never-smoker	115 (50.9%)	48 (45.3%)	67 (55.8%)	

## Data Availability

The datasets used and/or analyzed during the current study are available from the corresponding author upon reasonable request.

## Abbreviations

LUAD: Lung adenocarcinoma
TCGA: The Cancer Genome Atlas
ssGSEA: Single sample gene set enrichment analysis
WGCNA: Weighted gene coexpression network analysis
LASSO: Least absolute shrinkage and selection operator
ROC: Receiver operating characteristic
MAPK4: Mitogen-activated protein kinase 4
TNS4:Tensin 4WFDC2: Tensin 4WFDC2:WAP four-disulfide core domain 2
FSTL3: Follistatin-like 3
ITGA2: Integrin subunit alpha 2
KLK11: Kallikrein-related peptidase 11
PHLDB2: Pleckstrin homology like domain family *B* member 2
VGLL3: Vestigial like family member 3
SNX30: Sorting nexin family member 30
KCNQ3: Potassium voltage-gated channel subfamily *Q* member 3
SMAD9: SMAD family member 9
ANGPTL4: Angiopoietin-like 4
LAMA3: Laminin subunit alpha 3
STK32A: Serine/threonine kinase 32A
GSVA: Gene set variance analysis
NSCLC: Non-small-cell lung cancer
FSP1: S100 calcium-binding protein A4
MAPK: Mitogen-activated kinase-like protein
NRF2: Nuclear factor erythroid 2-like 2
TME: Tumor microenvironment
HIF1A:Hypoxia-inducible factor 1-*α*HILPDA: Hypoxia-inducible factor 1-*α*HILPDA:Hypoxia-inducible lipid droplet-associated
DEGs: Differentially expressed genes
GSVA: Gene set variation analysis
K-M: Kaplan–Meier
AUC: Area under the curve
OS: Overall survival
HR: Hazard ratio
CI: Confidence interval
TANs: Tumor-associated neutrophils.

## Supplementary Materials

Supplementary Materials Supplementary Figure 1. Hypoxia and ferroptosis scores were used as phenotypic characteristics. To assess the correlation of all samples in the TCGA-LUAD database, we performed a cluster analysis to ensure the completeness of the samples. Supplementary Figure 2. The allocation of risk scores and stratified prognosis according to other clinical characteristics, including age, sex, and *M* stage, are detailed. Supplementary Table 1. The ssGSEA outputs for the detailed score results. Supplementary Table 2. 8314 genes. Supplementary Table 3. 660 genes. Supplementary Table 4. Significant increase in LUAD samples, and 1,063 were significantly decreased. Supplementary Table 5. The expression profiles of the identified DEGs. Supplementary Table 6. The risk score of each individual in the TCGA set. Supplementary Table 7. The patients with LUAD in the TCGA training set were separated into two groups with the cutoff value at 1.0803. Supplementary Table 8. We used the same algorithm to compute risk scores for the patients in the TCGA test cohort (*n* = 142). Supplementary Table 9. The GSE31210 dataset. Supplementary Table 10. The other group was preferentially associated with bile acid metabolism, pancreatic beta cells, and KRAS signaling.

## References

[1] DeSantis C. E., Miller K. D., Goding Sauer A., Jemal A., Siegel R. L. (2019). Cancer statistics for African Americans, 2019. *CA: A Cancer Journal for Clinicians*.

[2] Li Q., Ma W., Chen S. (2020). High integrin *α*3 expression is associated with poor prognosis in patients with non-small cell lung cancer. *Translational Lung Cancer Research*.

[3] Herbst R. S., Morgensztern D., Boshoff C. (2018). The biology and management of non-small cell lung cancer. *Nature*.

[4] Bender E. (2014). Epidemiology: the dominant malignancy. *Nature*.

[5] Shukla S., Evans J. R., Malik R. (2017). Development of a RNA-seq based prognostic signature in lung adenocarcinoma. *Journal of the National Cancer Institute*.

[6] Siegel R. L., Miller K. D., Jemal A. (2020). Cancer statistics, 2020. *CA: A Cancer Journal for Clinicians*.

[7] Li X. T., Yang J. J., Wu Y. L., Hou J. (2018). Toward innovative combinational immunotherapy: a systems biology perspective. *Cancer Treatment Reviews*.

[8] Low J. L., Walsh R. J., Ang Y., Chan G., Soo R. A. (2019). The evolving immuno-oncology landscape in advanced lung cancer: first-line treatment of non-small cell lung cancer. *Ther Adv Med Oncol*.

[9] Wang B., Jing T., Jin W. (2020). KIAA1522 potentiates TNF*α*-NF*κ*B signaling to antagonize platinum-based chemotherapy in lung adenocarcinoma. *Journal of Experimental & Clinical Cancer Research*.

[10] Imielinski M., Berger A. H., Hammerman P. S. (2012). Mapping the hallmarks of lung adenocarcinoma with massively parallel sequencing. *Cell*.

[11] Santarpia M., Aguilar A., Chaib I. (2020). Non-small-cell lung cancer signaling pathways, metabolism, and PD-1/PD-L1 antibodies. *Cancers*.

[12] Li J., Cao F., Yin H. L. (2020). Ferroptosis: past, present and future. *Cell Death & Disease*.

[13] Mou Y., Wang J., Wu J. (2019). Ferroptosis, a new form of cell death: opportunities and challenges in cancer. *Journal of Hematology & Oncology*.

[14] Doll S., Freitas F. P., Shah R. (2019). FSP1 is a glutathione-independent ferroptosis suppressor. *Nature*.

[15] Poursaitidis I., Wang X., Crighton T. (2017). Oncogene-Selective sensitivity to Synchronous cell death following modulation of the Amino acid Nutrient cystine. *Cell Reports*.

[16] Alvarez S. W., Sviderskiy V. O., Terzi E. M. (2017). NFS1 undergoes positive selection in lung tumours and protects cells from ferroptosis. *Nature*.

[17] Liu P., Wu D., Duan J. (2020). NRF2 regulates the sensitivity of human NSCLC cells to cystine deprivation-induced ferroptosis via FOCAD-FAK signaling pathway. *Redox Biology*.

[18] Kung-Chun Chiu D., Pui-Wah Tse A., Law C. T. (2019). Hypoxia regulates the mitochondrial activity of hepatocellular carcinoma cells through HIF/HEY1/PINK1 pathway. *Cell Death & Disease*.

[19] Walsh J. C., Lebedev A., Aten E., Madsen K., Marciano L., Kolb H. C. (2014). The clinical importance of assessing tumor hypoxia: relationship of tumor hypoxia to prognosis and therapeutic opportunities. *Antioxidants and Redox Signaling*.

[20] Muz B., de la Puente P., Azab F., Azab A. K. (2015). The role of hypoxia in cancer progression, angiogenesis, metastasis, and resistance to therapy. *Hypoxia*.

[21] Semenza G. L. (2012). Hypoxia-inducible factors: mediators of cancer progression and targets for cancer therapy. *Trends in Pharmacological Sciences*.

[22] Fukumura D., Kloepper J., Amoozgar Z., Duda D. G., Jain R. K. (2018). Enhancing cancer immunotherapy using antiangiogenics: opportunities and challenges. *Nature Reviews Clinical Oncology*.

[23] Koh J., Jang J. Y., Keam B. (2016). EML4-ALK enhances programmed cell death-ligand 1 expression in pulmonary adenocarcinoma via hypoxia-inducible factor (HIF)-1*α* and STAT3. *OncoImmunology*.

[24] Ruf M., Moch H., Schraml P. (2016). PD-L1 expression is regulated by hypoxia inducible factor in clear cell renal cell carcinoma. *International Journal of Cancer*.

[25] Terry S., Buart S., Tan T. Z. (2017). Acquisition of tumor cell phenotypic diversity along the EMT spectrum under hypoxic pressure: consequences on susceptibility to cell-mediated cytotoxicity. *OncoImmunology*.

[26] Zhao M., Zhang Y., Zhang H. (2015). Hypoxia-induced cell stemness leads to drug resistance and poor prognosis in lung adenocarcinoma. *Lung Cancer*.

[27] Putra A. C., Tanimoto K., Arifin M., Hiyama K. (2011). Hypoxia-inducible factor-1*α* polymorphisms are associated with genetic aberrations in lung cancer. *Respirology*.

[28] Yang M., Chen P., Liu J. (2019). Clockophagy is a novel selective autophagy process favoring ferroptosis. *Science Advances*.

[29] Zou Y., Palte M. J., Deik A. A. (2019). A GPX4-dependent cancer cell state underlies the clear-cell morphology and confers sensitivity to ferroptosis. *Nature Communications*.

[30] Fuhrmann D. C., Mondorf A., Beifuß J., Jung M., Brune B. (2020). Hypoxia inhibits ferritinophagy, increases mitochondrial ferritin, and protects from ferroptosis. *Redox Biology*.

[31] Ma C., Luo H., Cao J. (2020). Identification of a novel tumor microenvironment-associated eight-gene signature for prognosis prediction in lung adenocarcinoma. *Frontiers in Molecular Biosciences*.

[32] Zhang A., Yang J., Ma C., Li F., Luo H. (2021). Development and validation of a Robust ferroptosis-related prognostic signature in lung adenocarcinoma. *Frontiers in Cell and Developmental Biology*.

[33] Dai Z., Liu T., Liu G. (2021). Identification of clinical and tumor microenvironment characteristics of hypoxia-related risk signature in lung adenocarcinoma. *Frontiers in Molecular Biosciences*.

[34] Song C., Guo Z., Yu D. (2020). A prognostic nomogram Combining immune-related gene signature and clinical factors predicts survival in patients with lung adenocarcinoma. *Front Oncol*.

[35] Okayama H., Kohno T., Ishii Y. (2012). Identification of genes upregulated in ALK-positive and EGFR/KRAS/ALK-negative lung adenocarcinomas. *Cancer Research*.

[36] Yamauchi M., Yamaguchi R., Nakata A. (2012). Epidermal growth factor receptor tyrosine kinase defines critical prognostic genes of stage I lung adenocarcinoma. *PLoS One*.

[37] Hanzelmann S., Castelo R., Guinney J. (2013). GSVA: gene set variation analysis for microarray and RNA-seq data. *BMC Bioinformatics*.

[38] Wang J. D., Zhou H. S., Tu X. X. (2019). Prediction of competing endogenous RNA coexpression network as prognostic markers in AML. *Aging (Albany NY)*.

[39] Zhou Y., Zhou B., Pache L. (2019). Metascape provides a biologist-oriented resource for the analysis of systems-level datasets. *Nature Communications*.

[40] Subramanian A., Tamayo P., Mootha V. K. (2005). Gene set enrichment analysis: a knowledge-based approach for interpreting genome-wide expression profiles. *Proc Natl Acad Sci U S A*.

[41] Livak K. J., Schmittgen T. D. (2001). Analysis of relative gene expression data using Real-time Quantitative PCR and the 2−ΔΔCT method. *Methods*.

[42] Dong H. X., Wang R., Jin X. Y., Zeng J., Pan J. (2018). LncRNA DGCR5 promotes lung adenocarcinoma (LUAD) progression via inhibiting hsa-mir-22-3p. *Journal of Cellular Physiology*.

[43] Devarakonda S., Morgensztern D., Govindan R. (2015). Genomic alterations in lung adenocarcinoma. *The Lancet Oncology*.

[44] Chen Y. J., Roumeliotis T. I., Chang Y. H. (2020). Proteogenomics of non-smoking lung cancer in East Asia Delineates molecular signatures of pathogenesis and progression. *Cell*.

[45] Kwon O. S., Kwon E. J., Kong H. J. (2020). Systematic identification of a nuclear receptor-enriched predictive signature for erastin-induced ferroptosis. *Redox Biology*.

[46] Lou J. S., Zhao L. P., Huang Z. H. (2021). Ginkgetin derived from Ginkgo biloba leaves enhances the therapeutic effect of cisplatin via ferroptosis-mediated disruption of the Nrf2/HO-1 axis in EGFR wild-type non-small-cell lung cancer. *Phytomedicine*.

[47] Brahimi-Horn M. C., Chiche J., Pouyssegur J. (2007). Hypoxia and cancer. *Journal of Molecular Medicine (Berlin)*.

[48] Eales K. L., Hollinshead K. E. R., Tennant D. A. (2016). Hypoxia and metabolic adaptation of cancer cells. *Oncogenesis*.

[49] Gilkes D. M., Semenza G. L., Wirtz D. (2014). Hypoxia and the extracellular matrix: drivers of tumour metastasis. *Nature Reviews Cancer*.

[50] Krock B. L., Skuli N., Simon M. C. (2011). Hypoxia-induced angiogenesis: good and evil. *Genes & Cancer*.

[51] Moreno Leon L., Gautier M., Allan R. (2019). The nuclear hypoxia-regulated NLUCAT1 long non-coding RNA contributes to an aggressive phenotype in lung adenocarcinoma through regulation of oxidative stress. *Oncogene*.

[52] Cao X., Fang X., Malik W. S. (2020). TRB3 interacts with ERK and JNK and contributes to the proliferation, apoptosis, and migration of lung adenocarcinoma cells. *Journal of Cellular Physiology*.

[53] Lee T. I., Young R. A. (2013). Transcriptional regulation and its misregulation in disease. *Cell*.

[54] Wang W., Shen T., Dong B. (2019). MAPK4 overexpression promotes tumor progression via noncanonical activation of AKT/mTOR signaling. *Journal of Clinical Investigation*.

[55] Sakashita K., Mimori K., Tanaka F. (2008). Prognostic relevance of Tensin4 expression in human gastric cancer. *Annals of Surgical Oncology*.

[56] Wojcik E., Tarapacz J., Rychlik U. (2016). Human epididymis protein 4 (HE4) in patients with small-cell lung cancer. *Clin Lab*.

[57] Choi S. I., Jang M. A., Jeon B. R., Shin H. B., Lee Y. K., Lee Y. W. (2017). Clinical usefulness of human epididymis protein 4 in lung cancer. *Ann Lab Med*.

[58] Huang W., Wu S., Lin Z., Chen P., Wu G. (2017). Evaluation of HE4 in the diagnosis and follow up of non-small cell lung cancers. *Clin Lab*.

[59] Zeng Q., Liu M., Zhou N., Liu L., Song X. (2016). Serum human epididymis protein 4 (HE4) may be a better tumor marker in early lung cancer. *Clinica Chimica Acta*.

[60] Gao L., Chen X., Wang Y., Zhang J. (2020). Up-regulation of FSTL3, regulated by lncRNA DSCAM-AS1/miR-122-5p Axis, promotes proliferation and migration of non-small cell lung cancer cells. *OncoTargets and Therapy*.

[61] Panagiotou G., Ghaly W., Upadhyay J., Pazaitou-Panayiotou K., Mantzoros C. S. (2021). Serum follistatin is increased in thyroid cancer and is associated with Adverse tumor characteristics in humans. *The Journal of Clinical Endocrinology & Metabolism*.

[62] Du J., Yan X., Mi S. (2020). Identification of prognostic model and biomarkers for cancer Stem cell characteristics in glioblastoma by network analysis of Multi-Omics data and stemness Indices. *Frontiers in Cell and Developmental Biology*.

[63] Ren D., Zhao J., Sun Y. (2019). Overexpressed ITGA2 promotes malignant tumor aggression by up-regulating PD-L1 expression through the activation of the STAT3 signaling pathway. *Journal of Experimental & Clinical Cancer Research*.

[64] Sasaki H., Kawano O., Endo K. (2006). Decreased kallikrein 11 messenger RNA expression in lung cancer. *Clinical Lung Cancer*.

[65] Planque C., Li L., Zheng Y. (2008). A multiparametric serum kallikrein panel for diagnosis of non-small cell lung carcinoma. *Clinical Cancer Research*.

[66] Chen G., Zhou T., Li Y., Yu Z., Sun L. (2017). p53 target miR-29c-3p suppresses colon cancer cell invasion and migration through inhibition of PHLDB2. *Biochemical and Biophysical Research Communications*.

[67] Stehbens S. J., Paszek M., Pemble H., Ettinger A., Gierke S., Wittmann T. (2014). CLASPs link focal-adhesion-associated microtubule capture to localized exocytosis and adhesion site turnover. *Nature Cell Biology*.

[68] Lim B. C., Matsumoto S., Yamamoto H. (2016). *Journal of Cell Science*.

[69] Astro V., Chiaretti S., Magistrati E., Fivaz M., de Curtis I. (2014). *Journal of Cell Science*.

[70] Ge D., Shao Y., Wang M., Tao H., Mu M., Tao X. (2021). RNA-seq-Based Screening in coal Dust-treated cells identified PHLDB2 as a novel lung cancer-related molecular marker. *BioMed Research International*.

[71] Simon E., Theze N., Fedou S., Thiebaud P., Faucheux C. (2017). Vestigial-like 3 is a novel Ets1 interacting partner and regulates trigeminal nerve formation and cranial neural crest migration. *Biol Open*.

[72] Gambaro K., Quinn M. C., Wojnarowicz P. M. (2013). VGLL3 expression is associated with a tumor suppressor phenotype in epithelial ovarian cancer. *Molecular Oncology*.

[73] Helias-Rodzewicz Z., Perot G., Chibon F. (2010). YAP1 and VGLL3, encoding two cofactors of TEAD transcription factors, are amplified and overexpressed in a subset of soft tissue sarcomas. *Genes, Chromosomes and Cancer*.

[74] Dexheimer V., Gabler J., Bomans K., Sims T., Omlor G., Richter W. (2016). Differential expression of TGF-beta superfamily members and role of Smad1/5/9-signalling in chondral versus endochondral chondrocyte differentiation. *Scientific Reports*.

[75] Gao L., Tian Q., Wu T. (2021). Reduction of miR-744 delivered by NSCLC cell-derived extracellular vesicles upregulates SUV39H1 to promote NSCLC progression via activation of the Smad9/BMP9 axis. *Journal of Translational Medicine*.

[76] Zhu X., Guo X., Wu S., Wei L. (2016). ANGPTL4 correlates with NSCLC progression and regulates epithelial-Mesenchymal Transition via ERK pathway. *Lung*.

[77] Virolle T., Coraux C., Ferrigno O., Cailleteau L., Ortonne J. P., Pognonec P. (2002). Binding of USF to a non-canonical E-box following stress results in a cell-specific derepression of the lama3 gene. *Nucleic Acids Research*.

[78] Xu S. F., Zheng Y., Zhang L. (2019). Long non-coding RNA LINC00628 interacts epigenetically with the LAMA3 promoter and contributes to lung adenocarcinoma. *Molecular Therapy - Nucleic Acids*.

[79] Ma F., Xie Y., Lei Y., Kuang Z., Liu X. (2020). The microRNA-130a-5p/RUNX2/STK32A network modulates tumor invasive and metastatic potential in non-small cell lung cancer. *BMC Cancer*.

[80] Haberg K., Lundmark R., Carlsson S. R. (2008). SNX18 is an SNX9 paralog that acts as a membrane tubulator in AP-1-positive endosomal trafficking. *Journal of Cell Science*.

[81] Sands T. T., Miceli F., Lesca G. (2019). Autism and developmental disability caused by KCNQ3 gain-of-function variants. *Annals of Neurology*.

[82] Li T., Li X., Mao R. (2022). NLRP2 inhibits cell proliferation and migration by regulating EMT in lung adenocarcinoma cells. *Cell Biology International*.

[83] Eruslanov E. B., Bhojnagarwala P. S., Quatromoni J. G. (2014). Tumor-associated neutrophils stimulate T cell responses in early-stage human lung cancer. *Journal of Clinical Investigation*.

[84] Chae Y. K., Choi W. M., Bae W. H. (2018). Overexpression of adhesion molecules and barrier molecules is associated with differential infiltration of immune cells in non-small cell lung cancer. *Scientific Reports*.

