# Supplementary material for: Prognostic Modeling of Lung Adenocarcinoma Based on Hypoxia and Ferroptosis-Related Genes
Source: J Oncol. 2022 Sep 19;2022:1022580. doi: 10.1155/2022/1022580 (PMC9553523; doi:10.1155/2022/1022580)
Supplement: Supplementary Materials — Supplementary Figure 1. Hypoxia and ferroptosis scores were used as phenotypic characteristics. To assess the correlation of all samples in the TCGA-LUAD database, we performed a cluster analysis to ensure the completeness of the samples. Supplementary Figure 2. The allocation of risk scores and stratified prognosis according to other clinical characteristics, including age, sex, and M stage, are detailed. Supplementary Table 1. The ssGSEA outputs for the detailed score results. Supplementary Table 2. 8314 genes. Supplementary Table 3. 660 genes. Supplementary Table 4. Significant increase in LUAD samples, and 1,063 were significantly decreased. Supplementary Table 5. The expression profiles of the identified DEGs. Supplementary Table 6. The risk score of each individual in the TCGA set. Supplementary Table 7. The patients with LUAD in the TCGA training set were separated into two groups with the cutoff value at 1.0803. Supplementary Table 8. We used the same algorithm to compute risk scores for the patients in the TCGA test cohort (n = 142). Supplementary Table 9. The GSE31210 dataset. Supplementary Table 10. The other group was preferentially associated with bile acid metabolism, pancreatic beta cells, and KRAS signaling. [file 1022580.f1.doc]

Supplementary Figure 1


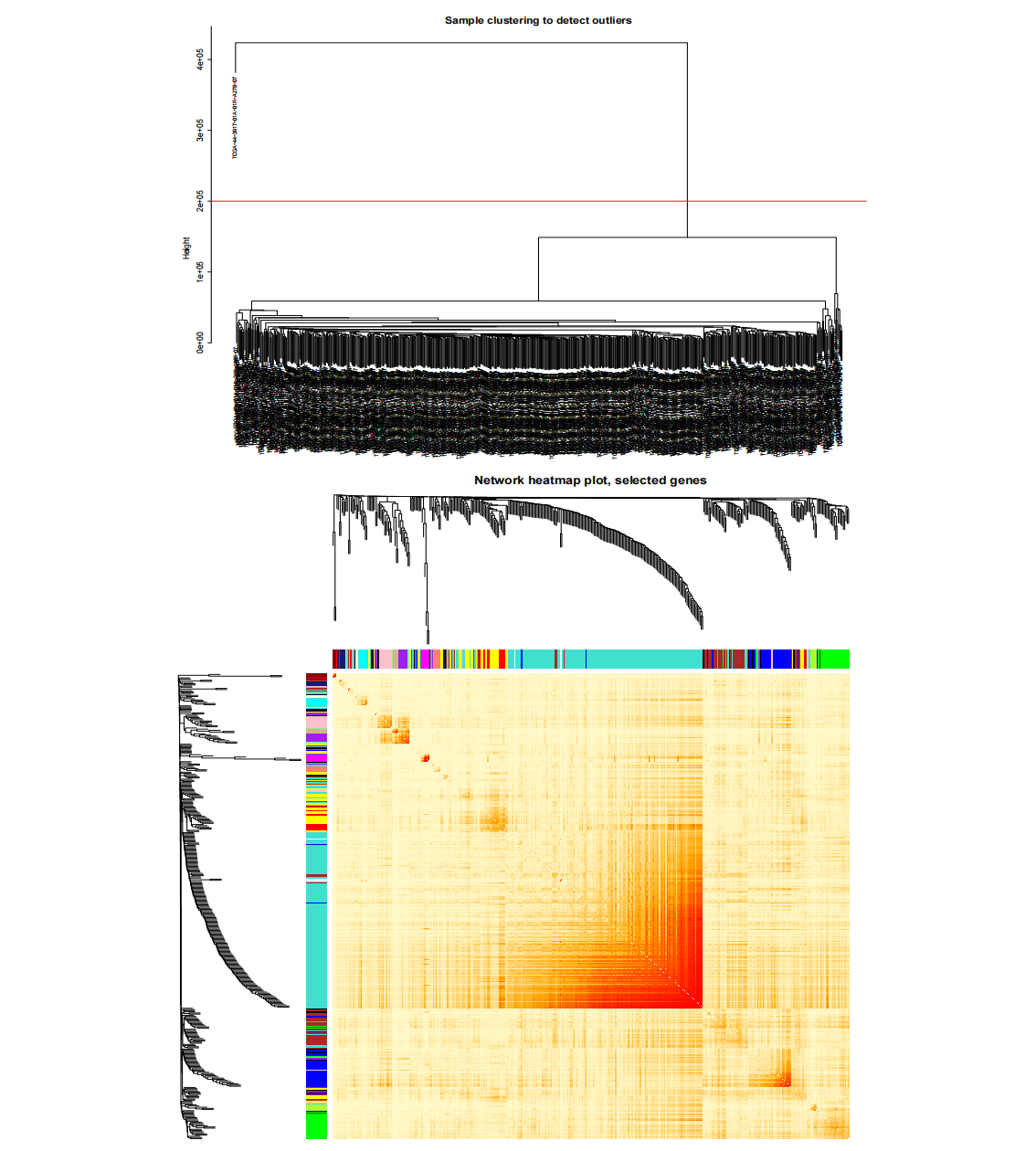


Supplement Figure2


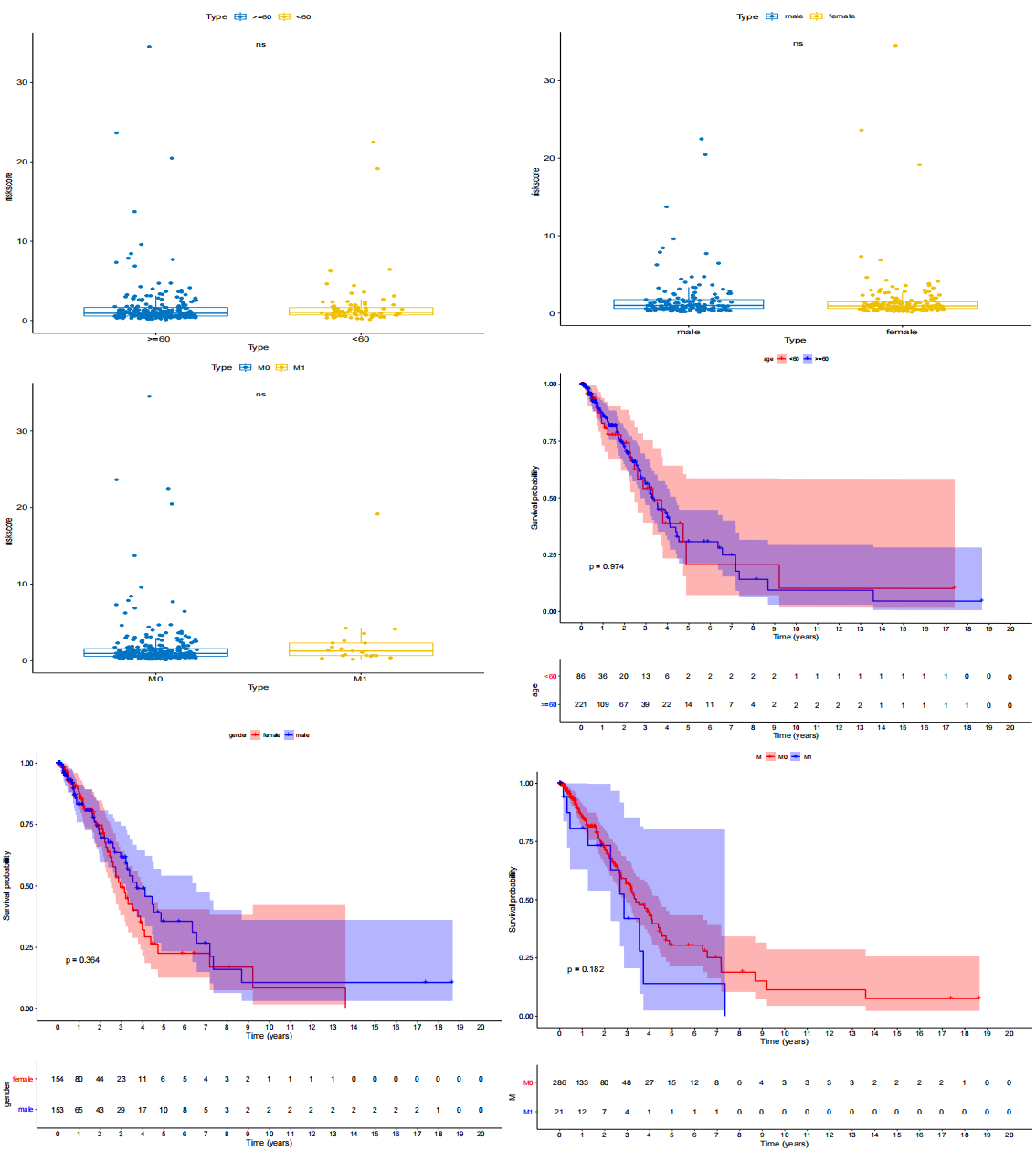


Supplementary Table 1

| id | ferroptosis | hypoxia |
| --- | --- | --- |
| TCGA-91-6831-11A-02R-1858-07 | 3.269811367 | 3.355531709 |
| TCGA-55-6979-11A-01R-1949-07 | 3.264937952 | 3.297788888 |
| TCGA-91-6849-11A-01R-1949-07 | 3.266846623 | 3.33609408 |
| TCGA-50-5936-11A-01R-1628-07 | 3.301424205 | 3.342766341 |
| TCGA-44-2668-11A-01R-1758-07 | 3.301012897 | 3.381673682 |
| TCGA-49-6745-11A-01R-1858-07 | 3.266761385 | 3.282997033 |
| TCGA-44-6146-11A-01R-1858-07 | 3.24682304 | 3.348965954 |
| TCGA-44-3396-11A-01R-1758-07 | 3.321446563 | 3.401668599 |
| TCGA-55-6980-11A-01R-1949-07 | 3.232893917 | 3.282980664 |
| TCGA-55-6982-11A-01R-1949-07 | 3.237256798 | 3.254813946 |
| TCGA-73-4676-11A-01R-1755-07 | 3.308005642 | 3.404973145 |
| TCGA-44-6778-11A-01R-1858-07 | 3.253829946 | 3.331231907 |
| TCGA-91-6847-11A-01R-1949-07 | 3.256772674 | 3.227568368 |
| TCGA-50-6595-11A-01R-1858-07 | 3.251979606 | 3.307859122 |
| TCGA-44-2655-11A-01R-1758-07 | 3.29801079 | 3.344968504 |
| TCGA-44-5645-11A-01R-1628-07 | 3.239547269 | 3.26888616 |
| TCGA-49-4512-11A-01R-1858-07 | 3.258893243 | 3.278431787 |
| TCGA-44-6148-11A-01R-1858-07 | 3.247790055 | 3.287752745 |
| TCGA-50-5930-11A-01R-1755-07 | 3.248775867 | 3.344898943 |
| TCGA-55-6969-11A-01R-1949-07 | 3.222544168 | 3.253330742 |
| TCGA-91-6836-11A-01R-1858-07 | 3.266847323 | 3.30051975 |
| TCGA-44-2657-11A-01R-1758-07 | 3.2904121 | 3.275234256 |
| TCGA-55-6986-11A-01R-1949-07 | 3.237284683 | 3.292072358 |
| TCGA-55-6985-11A-01R-1949-07 | 3.321562013 | 3.295975643 |
| TCGA-49-4490-11A-01R-1858-07 | 3.271562983 | 3.388142969 |
| TCGA-49-6743-11A-01R-1858-07 | 3.267510505 | 3.3597335 |
| TCGA-44-6145-11A-01R-1858-07 | 3.293872069 | 3.365661621 |
| TCGA-44-6144-11A-01R-1755-07 | 3.272976514 | 3.303947109 |
| TCGA-50-5939-11A-01R-1628-07 | 3.273753791 | 3.359185861 |
| TCGA-50-5935-11A-01R-1858-07 | 3.280960099 | 3.3455115 |
| TCGA-50-5931-11A-01R-1858-07 | 3.253376312 | 3.340383889 |
| TCGA-49-6761-11A-01R-1949-07 | 3.277142445 | 3.321533534 |
| TCGA-38-4627-11A-01R-1758-07 | 3.298789231 | 3.377277467 |
| TCGA-55-6971-11A-01R-1949-07 | 3.217675569 | 3.307681817 |
| TCGA-55-6975-11A-01R-1949-07 | 3.290006005 | 3.319348303 |
| TCGA-50-5932-11A-01R-1755-07 | 3.257176087 | 3.383355222 |
| TCGA-38-4626-11A-01R-1758-07 | 3.326119066 | 3.301285145 |
| TCGA-49-6744-11A-01R-1858-07 | 3.261223468 | 3.326992598 |
| TCGA-55-6983-11A-01R-1949-07 | 3.305984919 | 3.257571869 |
| TCGA-38-4625-11A-01R-1758-07 | 3.305001718 | 3.353784706 |
| TCGA-44-6147-11A-01R-1858-07 | 3.295878111 | 3.3985194 |
| TCGA-44-2665-11A-01R-1758-07 | 3.294302022 | 3.394388592 |
| TCGA-91-6829-11A-01R-1858-07 | 3.235433814 | 3.179279373 |
| TCGA-55-6978-11A-01R-1949-07 | 3.251222232 | 3.302993144 |
| TCGA-55-6981-11A-01R-1949-07 | 3.228765777 | 3.308724822 |
| TCGA-44-2661-11A-01R-1758-07 | 3.258460351 | 3.343219906 |
| TCGA-44-6776-11A-01R-1858-07 | 3.324272696 | 3.322196629 |
| TCGA-44-2662-11A-01R-1758-07 | 3.279250264 | 3.39230524 |
| TCGA-44-6777-11A-01R-1858-07 | 3.274261041 | 3.302659089 |
| TCGA-55-6970-11A-01R-1949-07 | 3.269910847 | 3.300075947 |
| TCGA-38-4632-11A-01R-1755-07 | 3.343573849 | 3.317802933 |
| TCGA-44-3398-11B-01R-1758-07 | 3.302917684 | 3.384781469 |
| TCGA-55-6968-11A-01R-1949-07 | 3.237456474 | 3.252596454 |
| TCGA-55-6984-11A-01R-1949-07 | 3.237496514 | 3.262872328 |
| TCGA-91-6835-11A-01R-1858-07 | 3.244789255 | 3.30125166 |
| TCGA-55-6972-11A-01R-1949-07 | 3.24775296 | 3.298471098 |
| TCGA-91-6828-11A-01R-1858-07 | 3.328324246 | 3.344179699 |
| TCGA-50-5933-11A-01R-1755-07 | 3.325614909 | 3.365070325 |
| TCGA-49-6742-11A-01R-1858-07 | 3.298774712 | 3.317483478 |
| TCGA-91-A4BD-01A-11R-A24H-07 | 3.224191924 | 3.205197412 |
| TCGA-78-7149-01A-11R-2039-07 | 3.272262009 | 3.256825951 |
| TCGA-78-7535-01A-11R-2066-07 | 3.113962498 | 3.2412592 |
| TCGA-49-6742-01A-11R-1858-07 | 3.3166413 | 3.342508819 |
| TCGA-05-4249-01A-01R-1107-07 | 3.224909416 | 3.217909742 |
| TCGA-64-1679-01A-21R-2066-07 | 3.242794208 | 3.464928787 |
| TCGA-69-7760-01A-11R-2170-07 | 3.220467239 | 3.267992793 |
| TCGA-05-4405-01A-21R-1858-07 | 3.221976808 | 3.274140408 |
| TCGA-55-6987-01A-11R-1949-07 | 3.315466291 | 3.362594122 |
| TCGA-78-8655-01A-11R-2403-07 | 3.173701174 | 3.184367158 |
| TCGA-MP-A4TF-01A-11R-A262-07 | 3.169520874 | 3.346180605 |
| TCGA-55-8203-01A-11R-2241-07 | 3.210176052 | 3.252595477 |
| TCGA-91-6848-01A-11R-1949-07 | 3.306625362 | 3.34255653 |
| TCGA-86-8073-01A-11R-2241-07 | 3.349625471 | 3.255341893 |
| TCGA-64-5775-01A-01R-1628-07 | 3.232166369 | 3.336863421 |
| TCGA-44-7659-01A-11R-2066-07 | 3.238415644 | 3.144651005 |
| TCGA-55-A4DG-01A-11R-A24H-07 | 3.0948816 | 3.165860924 |
| TCGA-55-6985-01A-11R-1949-07 | 3.318690339 | 3.361674205 |
| TCGA-86-8054-01A-11R-2241-07 | 3.320862035 | 3.382763572 |
| TCGA-38-4629-01A-02R-1206-07 | 3.294324328 | 3.446655774 |
| TCGA-78-7147-01A-11R-2039-07 | 3.227526765 | 3.205818484 |
| TCGA-55-8092-01A-11R-2241-07 | 3.188371949 | 3.212201363 |
| TCGA-75-6205-01A-11R-1755-07 | 3.362261258 | 3.296611588 |
| TCGA-86-A456-01A-11R-A24H-07 | 3.273462694 | 3.269983109 |
| TCGA-05-5429-01A-01R-1628-07 | 3.274942736 | 3.376857228 |
| TCGA-55-A48X-01A-11R-A24H-07 | 3.211053403 | 3.223414809 |
| TCGA-86-8673-01A-11R-2403-07 | 3.212991407 | 3.342382465 |
| TCGA-86-8674-01A-21R-2403-07 | 3.318442453 | 3.253976301 |
| TCGA-49-6744-01A-11R-1858-07 | 3.268148744 | 3.365530217 |
| TCGA-44-5643-01A-01R-1628-07 | 3.396041212 | 3.267821631 |
| TCGA-05-4382-01A-01R-1206-07 | 3.278075728 | 3.431304561 |
| TCGA-49-AARQ-01A-11R-A41B-07 | 3.226903976 | 3.135531208 |
| TCGA-55-8094-01A-11R-2241-07 | 3.226854653 | 3.292385242 |
| TCGA-MP-A4TC-01A-11R-A24X-07 | 3.241881651 | 3.407930295 |
| TCGA-49-AAR0-01A-21R-A39D-07 | 3.313517651 | 3.278834892 |
| TCGA-05-4427-01A-21R-1858-07 | 3.21667688 | 3.290101838 |
| TCGA-75-6214-01A-41R-1949-07 | 3.299423936 | 3.298797696 |
| TCGA-69-7763-01A-11R-2170-07 | 3.255688448 | 3.354488789 |
| TCGA-86-8076-01A-31R-2241-07 | 3.3172725 | 3.333046756 |
| TCGA-44-4112-01A-01R-1107-07 | 3.253335664 | 3.413091517 |
| TCGA-69-7765-01A-11R-2170-07 | 3.210352052 | 3.404238256 |
| TCGA-55-7281-01A-11R-2039-07 | 3.216455522 | 3.34322926 |
| TCGA-50-6597-01A-11R-1858-07 | 3.184981155 | 3.212005418 |
| TCGA-73-4677-01A-01R-1206-07 | 3.309876665 | 3.275796072 |
| TCGA-05-4434-01A-01R-1206-07 | 3.410786099 | 3.437708247 |
| TCGA-MP-A4SW-01A-21R-A24X-07 | 3.26262531 | 3.270737277 |
| TCGA-55-6972-01A-11R-1949-07 | 3.110869948 | 2.925450121 |
| TCGA-78-7633-01A-11R-2066-07 | 3.255057099 | 3.246036589 |
| TCGA-44-2659-01A-01R-0946-07 | 3.182280708 | 3.205034834 |
| TCGA-55-6983-01A-11R-1949-07 | 3.344254112 | 3.307005831 |
| TCGA-97-A4M0-01A-11R-A24X-07 | 3.142999813 | 3.183019457 |
| TCGA-50-5068-01A-01R-1628-07 | 3.238309339 | 3.261062279 |
| TCGA-86-A4P8-01A-11R-A24X-07 | 3.231309694 | 3.248957251 |
| TCGA-86-A4D0-01A-11R-A24H-07 | 3.351717892 | 3.254003971 |
| TCGA-55-8085-01A-11R-2241-07 | 3.300490231 | 3.294568333 |
| TCGA-50-5935-01A-11R-1755-07 | 3.222924623 | 3.327197865 |
| TCGA-53-7813-01A-11R-2170-07 | 3.172158594 | 3.216784012 |
| TCGA-73-4662-01A-01R-1206-07 | 3.190937506 | 3.227941877 |
| TCGA-62-8399-01A-21R-2326-07 | 3.165922949 | 3.307959904 |
| TCGA-44-7671-01A-11R-2066-07 | 3.377733948 | 3.338122971 |
| TCGA-97-7552-01A-11R-2039-07 | 3.312364402 | 3.282838634 |
| TCGA-44-6145-01A-11R-1755-07 | 3.371604491 | 3.381976389 |
| TCGA-44-7670-01A-11R-2066-07 | 3.421411119 | 3.340417229 |
| TCGA-49-AARO-01A-12R-A41B-07 | 3.226611821 | 3.354395593 |
| TCGA-44-2657-01A-01R-1107-07 | 3.266283323 | 3.290134156 |
| TCGA-55-8204-01A-11R-2241-07 | 3.358046962 | 3.33415332 |
| TCGA-64-1676-01A-01R-0946-07 | 3.171821379 | 3.275433316 |
| TCGA-91-8496-01A-11R-2403-07 | 3.24660051 | 3.241841123 |
| TCGA-35-3615-01A-01R-0946-07 | 3.261677447 | 3.114304954 |
| TCGA-55-8096-01A-11R-2241-07 | 3.208587405 | 3.375781127 |
| TCGA-67-6215-01A-11R-1755-07 | 3.207735449 | 3.20195268 |
| TCGA-53-A4EZ-01A-12R-A24X-07 | 3.274638834 | 3.182629377 |
| TCGA-MP-A4SY-01A-21R-A24X-07 | 3.288581447 | 3.363342721 |
| TCGA-73-4676-01A-01R-1755-07 | 3.361431501 | 3.380614454 |
| TCGA-86-8075-01A-11R-2241-07 | 3.29916935 | 3.444254655 |
| TCGA-78-8660-01A-11R-2403-07 | 3.337379023 | 3.26735674 |
| TCGA-91-6840-01A-11R-1949-07 | 3.189632047 | 3.191021063 |
| TCGA-44-2665-01A-01R-0946-07 | 3.25069865 | 3.384523778 |
| TCGA-44-6775-01A-11R-1858-07 | 3.300212977 | 3.343320071 |
| TCGA-75-7025-01A-12R-1949-07 | 3.173376553 | 3.225661598 |
| TCGA-44-A479-01A-31R-A24H-07 | 3.273545491 | 3.266547652 |
| TCGA-44-3917-01B-02R-A277-07 | 2.95157504 | 2.614468683 |
| TCGA-50-6590-01A-12R-1858-07 | 3.255172298 | 3.352536857 |
| TCGA-69-8255-01A-11R-2287-07 | 3.322953029 | 3.194606645 |
| TCGA-73-7498-01A-12R-2187-07 | 3.215015046 | 3.168186399 |
| TCGA-78-7148-01A-11R-2039-07 | 3.369754576 | 3.370210168 |
| TCGA-95-A4VN-01A-11R-A262-07 | 3.28191569 | 3.338618395 |
| TCGA-78-7152-01A-11R-2039-07 | 3.333556 | 3.256891675 |
| TCGA-MP-A4TD-01A-32R-A262-07 | 3.331112379 | 3.354982639 |
| TCGA-55-7914-01A-11R-2170-07 | 3.185050199 | 3.217220553 |
| TCGA-44-A47A-01A-21R-A24H-07 | 3.272342736 | 3.245144494 |
| TCGA-97-A4M3-01A-11R-A24X-07 | 3.315615979 | 3.33652192 |
| TCGA-67-3773-01A-01R-0946-07 | 3.202499254 | 3.232046977 |
| TCGA-50-6593-01A-11R-1755-07 | 3.256612633 | 3.318526822 |
| TCGA-73-4659-01A-01R-1206-07 | 3.394006488 | 3.404642326 |
| TCGA-38-4630-01A-01R-1206-07 | 3.126512051 | 3.265192962 |
| TCGA-78-7540-01A-11R-2066-07 | 3.282211748 | 3.296250099 |
| TCGA-J2-A4AD-01A-11R-A24H-07 | 3.284642585 | 3.271492588 |
| TCGA-44-6778-01A-11R-1858-07 | 3.300480212 | 3.34877979 |
| TCGA-38-A44F-01A-11R-A24H-07 | 3.286667859 | 3.348927613 |
| TCGA-50-6592-01A-11R-1755-07 | 3.435062949 | 3.399894897 |
| TCGA-44-6775-01A-11R-A278-07 | 3.234535721 | 3.287834935 |
| TCGA-95-8494-01A-11R-2326-07 | 3.214832213 | 3.354429662 |
| TCGA-44-2656-01A-02R-0946-07 | 3.268215688 | 3.288073139 |
| TCGA-50-5055-01A-01R-1628-07 | 3.256839785 | 3.342967967 |
| TCGA-75-5147-01A-01R-1628-07 | 3.291681458 | 3.323812858 |
| TCGA-86-8278-01A-11R-2287-07 | 3.260316797 | 3.309377915 |
| TCGA-05-4250-01A-01R-1107-07 | 3.309865797 | 3.421170393 |
| TCGA-4B-A93V-01A-11R-A39D-07 | 3.279812113 | 3.333875015 |
| TCGA-86-7713-01A-11R-2066-07 | 3.209049046 | 3.267945992 |
| TCGA-62-A46V-01A-11R-A24H-07 | 3.2182537 | 3.187675031 |
| TCGA-55-A48Y-01A-11R-A24H-07 | 3.338722134 | 3.319398518 |
| TCGA-71-6725-01A-11R-1858-07 | 3.131954826 | 3.076430882 |
| TCGA-55-6642-01A-11R-1858-07 | 3.284777763 | 3.442744174 |
| TCGA-62-A472-01A-11R-A24H-07 | 3.20079437 | 3.246612734 |
| TCGA-44-6146-01A-11R-1755-07 | 3.276033508 | 3.242380774 |
| TCGA-MP-A4SV-01A-11R-A24X-07 | 3.219250118 | 3.273505138 |
| TCGA-49-AARN-01A-21R-A41B-07 | 3.280411716 | 3.324269134 |
| TCGA-50-5072-01A-21R-1858-07 | 3.386463749 | 3.412097125 |
| TCGA-55-5899-01A-11R-1628-07 | 3.320764469 | 3.322140143 |
| TCGA-97-A4LX-01A-11R-A24X-07 | 3.210711333 | 3.287155355 |
| TCGA-L9-A444-01A-21R-A24H-07 | 3.119068144 | 3.241216512 |
| TCGA-55-8615-01A-11R-2403-07 | 3.276977361 | 3.303329205 |
| TCGA-50-7109-01A-11R-2039-07 | 3.244222405 | 3.248133894 |
| TCGA-91-7771-01A-11R-2170-07 | 3.2948991 | 3.366896032 |
| TCGA-97-A4M1-01A-11R-A24X-07 | 3.144489136 | 3.145664147 |
| TCGA-44-5644-01A-21R-2039-07 | 3.275180316 | 3.338043254 |
| TCGA-78-7536-01A-11R-2066-07 | 3.268062901 | 3.240287298 |
| TCGA-MP-A4TI-01A-21R-A24X-07 | 3.224676399 | 3.365198034 |
| TCGA-05-4403-01A-01R-1206-07 | 3.344949697 | 3.38791363 |
| TCGA-44-2666-01B-02R-A277-07 | 2.8931073 | 2.832582393 |
| TCGA-99-7458-01A-11R-2039-07 | 3.238862593 | 3.311881131 |
| TCGA-64-5778-01A-01R-1628-07 | 3.146464458 | 3.213179141 |
| TCGA-78-7145-01A-11R-2039-07 | 3.239671746 | 3.37741846 |
| TCGA-NJ-A4YP-01A-11R-A262-07 | 3.204865491 | 3.297602422 |
| TCGA-55-7283-01A-11R-2039-07 | 3.211808229 | 3.180487005 |
| TCGA-55-8208-01A-11R-2241-07 | 3.372522933 | 3.338111185 |
| TCGA-55-8302-01A-11R-2326-07 | 3.296598829 | 3.334552426 |
| TCGA-05-4384-01A-01R-1755-07 | 3.317724221 | 3.284737979 |
| TCGA-55-8511-01A-11R-2403-07 | 3.264626369 | 3.269370966 |
| TCGA-97-7941-01A-11R-2187-07 | 3.327174907 | 3.344624407 |
| TCGA-62-8397-01A-11R-2326-07 | 3.189107534 | 3.264593952 |
| TCGA-91-6830-01A-11R-1949-07 | 3.362556855 | 3.425807617 |
| TCGA-44-3398-01A-01R-1107-07 | 3.328146998 | 3.468862362 |
| TCGA-55-7913-01B-11R-2241-07 | 3.225134155 | 3.244574363 |
| TCGA-44-2656-01A-02R-A278-07 | 3.150051609 | 3.131059447 |
| TCGA-55-1592-01A-01R-0946-07 | 3.29458002 | 3.283054713 |
| TCGA-78-7537-01A-11R-2066-07 | 3.205483706 | 3.220864192 |
| TCGA-78-7166-01A-12R-2066-07 | 3.29532508 | 3.369870613 |
| TCGA-55-7995-01A-11R-2187-07 | 3.443543796 | 3.338086073 |
| TCGA-62-8395-01A-11R-2326-07 | 3.315659907 | 3.314246543 |
| TCGA-55-7727-01A-11R-2170-07 | 3.211515195 | 3.207919237 |
| TCGA-49-4494-01A-01R-1206-07 | 3.321119505 | 3.344481578 |
| TCGA-49-4501-01A-01R-1206-07 | 3.248438318 | 3.326899318 |
| TCGA-91-6847-01A-11R-1949-07 | 3.159682471 | 3.202492619 |
| TCGA-75-5126-01A-01R-1755-07 | 3.34984318 | 3.387276574 |
| TCGA-86-A4JF-01A-11R-A24X-07 | 3.199388108 | 3.332297983 |
| TCGA-49-AAR2-01A-11R-A39D-07 | 3.120919228 | 3.114790887 |
| TCGA-75-6211-01A-11R-1755-07 | 3.282430871 | 3.284771958 |
| TCGA-73-4675-01A-01R-1206-07 | 3.303856584 | 3.317788681 |
| TCGA-44-2668-01A-01R-0946-07 | 3.283196699 | 3.438630963 |
| TCGA-38-4628-01A-01R-1206-07 | 3.236879865 | 3.186144714 |
| TCGA-O1-A52J-01A-11R-A262-07 | 3.130860673 | 3.247922594 |
| TCGA-05-4396-01A-21R-1858-07 | 3.276084175 | 3.265614071 |
| TCGA-44-7660-01A-11R-2066-07 | 3.354069196 | 3.253085379 |
| TCGA-86-8279-01A-11R-2287-07 | 3.161879909 | 3.295728385 |
| TCGA-97-7937-01A-11R-2170-07 | 3.139448281 | 3.171420257 |
| TCGA-05-5420-01A-01R-1628-07 | 3.331103085 | 3.302350318 |
| TCGA-78-7542-01A-21R-2066-07 | 3.255113546 | 3.313949369 |
| TCGA-67-3771-01A-01R-0946-07 | 3.384352247 | 3.391399004 |
| TCGA-38-7271-01A-11R-2039-07 | 3.271850803 | 3.340508682 |
| TCGA-78-7163-01A-12R-2066-07 | 3.25293031 | 3.266473895 |
| TCGA-73-4658-01A-01R-1755-07 | 3.282974537 | 3.430672893 |
| TCGA-75-6207-01A-11R-1755-07 | 3.232203416 | 3.256151179 |
| TCGA-78-7160-01A-11R-2039-07 | 3.37569736 | 3.374590811 |
| TCGA-49-6743-01A-11R-1858-07 | 3.373216391 | 3.362180424 |
| TCGA-05-4425-01A-01R-1755-07 | 3.321953629 | 3.341417271 |
| TCGA-78-7143-01A-11R-2039-07 | 3.239125292 | 3.219808096 |
| TCGA-55-8087-01A-11R-2241-07 | 3.112071306 | 3.073256202 |
| TCGA-55-6986-01A-11R-1949-07 | 3.302245422 | 3.315035648 |
| TCGA-78-7220-01A-11R-2039-07 | 3.377906868 | 3.322749922 |
| TCGA-L9-A8F4-01A-11R-A39D-07 | 3.201767463 | 3.259701551 |
| TCGA-35-5375-01A-01R-1628-07 | 3.336900747 | 3.306462821 |
| TCGA-50-5939-01A-11R-1628-07 | 3.399380571 | 3.470562377 |
| TCGA-55-8510-01A-11R-2403-07 | 3.305412588 | 3.306439552 |
| TCGA-38-6178-01A-11R-1755-07 | 3.26172667 | 3.352368713 |
| TCGA-MN-A4N4-01A-12R-A24X-07 | 3.241593771 | 3.380222037 |
| TCGA-44-7662-01A-11R-2066-07 | 3.215893283 | 3.343279805 |
| TCGA-MP-A4T4-01A-11R-A262-07 | 3.265168064 | 3.299138745 |
| TCGA-95-A4VP-01A-21R-A262-07 | 3.250573118 | 3.36953708 |
| TCGA-55-8206-01A-11R-2241-07 | 3.23886788 | 3.18902202 |
| TCGA-86-8585-01A-11R-2403-07 | 3.168950842 | 3.352995068 |
| TCGA-95-7562-01A-11R-2241-07 | 3.279799963 | 3.283988031 |
| TCGA-05-4426-01A-01R-1206-07 | 3.22179186 | 3.389225144 |
| TCGA-55-8508-01A-11R-2403-07 | 3.23618751 | 3.396383478 |
| TCGA-99-8025-01A-11R-2241-07 | 3.24020416 | 3.268185436 |
| TCGA-44-7672-01A-11R-2066-07 | 3.275714227 | 3.378448165 |
| TCGA-NJ-A4YG-01A-22R-A262-07 | 3.264728692 | 3.22715978 |
| TCGA-44-8117-01A-11R-2241-07 | 3.105281369 | 3.257400004 |
| TCGA-62-A46O-01A-11R-A24H-07 | 3.300373818 | 3.232019204 |
| TCGA-93-A4JO-01A-21R-A24X-07 | 3.194490731 | 3.225927598 |
| TCGA-44-2666-01A-01R-0946-07 | 3.292525814 | 3.251863192 |
| TCGA-67-3774-01A-01R-0946-07 | 3.334605733 | 3.367187049 |
| TCGA-86-8358-01A-11R-2326-07 | 3.32706624 | 3.337569452 |
| TCGA-62-8394-01A-11R-2326-07 | 3.284598933 | 3.344299116 |
| TCGA-MP-A4T6-01A-32R-A262-07 | 3.030874267 | 3.038734409 |
| TCGA-55-8514-01A-11R-2403-07 | 3.132718436 | 3.200361103 |
| TCGA-44-6147-01A-11R-A278-07 | 3.142924286 | 3.189642424 |
| TCGA-64-1681-01A-11R-2066-07 | 3.307007 | 3.335631981 |
| TCGA-50-5944-01A-11R-1755-07 | 3.278825862 | 3.234798568 |
| TCGA-44-A47B-01A-11R-A24H-07 | 3.186654878 | 3.275147599 |
| TCGA-L4-A4E6-01A-11R-A24H-07 | 3.213255199 | 3.282244259 |
| TCGA-49-AARR-01A-11R-A41B-07 | 3.238120698 | 3.314378862 |
| TCGA-49-4507-01A-01R-1206-07 | 3.295455967 | 3.298823308 |
| TCGA-75-7027-01A-11R-1949-07 | 3.345314257 | 3.355947894 |
| TCGA-38-4625-01A-01R-1206-07 | 3.257901303 | 3.244765864 |
| TCGA-44-8120-01A-11R-2241-07 | 3.17211418 | 3.290148845 |
| TCGA-44-6776-01A-11R-1858-07 | 3.30698278 | 3.211303972 |
| TCGA-62-8402-01A-11R-2326-07 | 3.306187836 | 3.193238544 |
| TCGA-75-5125-01A-01R-1755-07 | 3.250636996 | 3.31005268 |
| TCGA-64-1678-01A-01R-0946-07 | 3.240513318 | 3.294246195 |
| TCGA-95-7567-01A-11R-2066-07 | 3.184563102 | 3.237866783 |
| TCGA-99-AA5R-01A-11R-A39D-07 | 3.176801002 | 3.322953566 |
| TCGA-97-8174-01A-11R-2287-07 | 3.232508064 | 3.259006236 |
| TCGA-55-8089-01A-11R-2241-07 | 3.291609287 | 3.27338826 |
| TCGA-69-A59K-01A-11R-A262-07 | 3.295602827 | 3.276407679 |
| TCGA-55-6978-01A-11R-1949-07 | 3.298014744 | 3.382213902 |
| TCGA-78-7153-01A-11R-2039-07 | 3.240770386 | 3.246715107 |
| TCGA-05-5715-01A-01R-1628-07 | 3.270004177 | 3.269502675 |
| TCGA-69-8254-01A-11R-2287-07 | 3.34224486 | 3.309695631 |
| TCGA-J2-A4AE-01A-21R-A24H-07 | 3.276200833 | 3.250745888 |
| TCGA-78-7150-01A-21R-2039-07 | 3.358301305 | 3.438245893 |
| TCGA-93-A4JN-01A-11R-A24X-07 | 3.206535279 | 3.270964637 |
| TCGA-J2-8194-01A-11R-2241-07 | 3.33589915 | 3.366628433 |
| TCGA-55-6712-01A-11R-1858-07 | 3.261479124 | 3.347139987 |
| TCGA-44-2668-01A-01R-A278-07 | 3.203892936 | 3.383785173 |
| TCGA-55-7815-01A-11R-2170-07 | 3.294657037 | 3.316298984 |
| TCGA-50-5049-01A-01R-1628-07 | 3.274667807 | 3.354603721 |
| TCGA-49-4510-01A-01R-1206-07 | 3.237919554 | 3.328470205 |
| TCGA-73-4666-01A-01R-1206-07 | 3.354071024 | 3.279471883 |
| TCGA-MP-A5C7-01A-11R-A262-07 | 3.194240363 | 3.103634308 |
| TCGA-55-7994-01A-11R-2187-07 | 3.233030864 | 3.201220535 |
| TCGA-95-7039-01A-11R-1949-07 | 3.202774267 | 3.315441001 |
| TCGA-78-7156-01A-11R-2039-07 | 3.17836182 | 3.113695078 |
| TCGA-55-8299-01A-11R-2287-07 | 3.267391365 | 3.372953499 |
| TCGA-44-7661-01A-11R-2066-07 | 3.264150912 | 3.468379471 |
| TCGA-05-5428-01A-01R-1628-07 | 3.277908143 | 3.292956389 |
| TCGA-55-7724-01A-11R-2170-07 | 3.326126146 | 3.366447031 |
| TCGA-97-7938-01A-11R-2170-07 | 3.208461334 | 3.268211391 |
| TCGA-75-6206-01A-11R-1755-07 | 3.285760605 | 3.282287111 |
| TCGA-05-4418-01A-01R-1206-07 | 3.346496462 | 3.398082822 |
| TCGA-44-2661-01A-01R-1107-07 | 3.245567515 | 3.236347898 |
| TCGA-80-5608-01A-31R-1949-07 | 3.312341019 | 3.371911106 |
| TCGA-55-6984-01A-11R-1949-07 | 3.337098165 | 3.356934899 |
| TCGA-75-7031-01A-11R-1949-07 | 3.312295815 | 3.272053028 |
| TCGA-99-8032-01A-11R-2241-07 | 3.305000151 | 3.427670108 |
| TCGA-55-8512-01A-11R-2403-07 | 3.313802506 | 3.250238193 |
| TCGA-97-A4M2-01A-12R-A24X-07 | 3.246933824 | 3.184688878 |
| TCGA-50-6673-01A-11R-1949-07 | 3.317262375 | 3.376505597 |
| TCGA-67-3772-01A-01R-0946-07 | 3.253343267 | 3.335412993 |
| TCGA-86-7954-01A-11R-2187-07 | 3.326044157 | 3.205661707 |
| TCGA-44-6775-01C-02R-A277-07 | 2.860263525 | 2.8323088 |
| TCGA-L9-A743-01A-43R-A39D-07 | 3.291753125 | 3.384171844 |
| TCGA-05-4433-01A-22R-1858-07 | 3.347845253 | 3.28253854 |
| TCGA-91-A4BC-01A-11R-A24H-07 | 3.195333217 | 3.282535442 |
| TCGA-91-6829-01A-21R-1858-07 | 3.221866865 | 3.315457804 |
| TCGA-44-6779-01A-11R-1858-07 | 3.302490846 | 3.316602276 |
| TCGA-05-4432-01A-01R-1206-07 | 3.346201978 | 3.427966418 |
| TCGA-05-4398-01A-01R-1206-07 | 3.23448842 | 3.407494476 |
| TCGA-L4-A4E5-01A-11R-A24X-07 | 3.151677781 | 3.182116263 |
| TCGA-53-7624-01A-11R-2066-07 | 3.298612155 | 3.371587224 |
| TCGA-55-6968-01A-11R-1949-07 | 3.332819095 | 3.291560136 |
| TCGA-93-A4JP-01A-11R-A24X-07 | 3.271536649 | 3.293449283 |
| TCGA-44-2655-01A-01R-0946-07 | 3.319576651 | 3.228505838 |
| TCGA-55-1594-01A-01R-0946-07 | 3.300742318 | 3.268656209 |
| TCGA-50-5941-01A-11R-1755-07 | 3.279182303 | 3.301193855 |
| TCGA-49-6767-01A-11R-1858-07 | 3.182488184 | 3.248420114 |
| TCGA-75-5122-01A-01R-1755-07 | 3.323199056 | 3.301962222 |
| TCGA-78-7539-01A-11R-2066-07 | 3.146470703 | 3.189015356 |
| TCGA-55-7726-01A-11R-2170-07 | 3.271702599 | 3.367127263 |
| TCGA-78-7162-01A-21R-2066-07 | 3.343516224 | 3.304253928 |
| TCGA-55-6979-01A-11R-1949-07 | 3.260119831 | 3.301532067 |
| TCGA-86-8669-01A-11R-2403-07 | 3.291274712 | 3.342768423 |
| TCGA-44-3918-01A-01R-1107-07 | 3.247472388 | 3.334469679 |
| TCGA-75-6212-01A-11R-1755-07 | 3.302209829 | 3.382708031 |
| TCGA-44-5645-01B-04R-A277-07 | 2.665522608 | 2.532480966 |
| TCGA-44-3396-01A-01R-1206-07 | 3.267764685 | 3.360506085 |
| TCGA-50-8457-01A-11R-2326-07 | 3.210756603 | 3.285158404 |
| TCGA-49-AARE-01A-11R-A41B-07 | 3.318018559 | 3.253627753 |
| TCGA-49-4487-01A-21R-1858-07 | 3.257910274 | 3.395289544 |
| TCGA-44-6774-01A-21R-1858-07 | 3.288712037 | 3.405257582 |
| TCGA-L9-A5IP-01A-21R-A39D-07 | 3.325056583 | 3.397424408 |
| TCGA-69-8253-01A-11R-2287-07 | 3.296538822 | 3.35918744 |
| TCGA-L9-A7SV-01A-11R-A39D-07 | 3.113835857 | 3.063284249 |
| TCGA-97-8172-01A-11R-2287-07 | 3.172445352 | 3.193817539 |
| TCGA-86-A4P7-01A-11R-A24X-07 | 3.219464384 | 3.277011343 |
| TCGA-55-7574-01A-11R-2039-07 | 3.216266307 | 3.3817127 |
| TCGA-NJ-A4YI-01A-11R-A262-07 | 3.187779978 | 3.16677097 |
| TCGA-44-2668-01B-02R-A277-07 | 2.950915726 | 3.135800813 |
| TCGA-55-8620-01A-11R-2403-07 | 3.287381362 | 3.206262838 |
| TCGA-55-7728-01A-11R-2187-07 | 3.206696306 | 3.252503825 |
| TCGA-50-5066-02A-11R-2090-07 | 3.419121046 | 3.360808039 |
| TCGA-97-7554-01A-11R-2039-07 | 3.173031778 | 3.324179236 |
| TCGA-78-7167-01A-11R-2066-07 | 3.215356595 | 3.125182476 |
| TCGA-50-6595-01A-12R-1858-07 | 3.302087981 | 3.439162302 |
| TCGA-64-1677-01A-01R-0946-07 | 3.19748205 | 3.268461119 |
| TCGA-97-A4M6-01A-11R-A24X-07 | 3.28342419 | 3.291881255 |
| TCGA-97-8547-01A-11R-2403-07 | 3.268065524 | 3.320321328 |
| TCGA-62-A470-01A-11R-A24H-07 | 3.32618604 | 3.279998977 |
| TCGA-97-8171-01A-11R-2287-07 | 3.057107265 | 3.034703276 |
| TCGA-44-6148-01A-11R-1755-07 | 3.221174132 | 3.284088984 |
| TCGA-MP-A4T7-01A-11R-A24X-07 | 3.334011905 | 3.428813301 |
| TCGA-49-4514-01A-21R-1858-07 | 3.337024187 | 3.251103178 |
| TCGA-50-5946-01A-11R-1755-07 | 3.052874895 | 3.197816954 |
| TCGA-55-8621-01A-11R-2403-07 | 3.316219563 | 3.359760026 |
| TCGA-55-8614-01A-11R-2403-07 | 3.269704607 | 3.412332576 |
| TCGA-71-8520-01A-11R-2403-07 | 3.185659765 | 3.335258142 |
| TCGA-69-7978-01A-11R-2187-07 | 3.338274735 | 3.417342759 |
| TCGA-L9-A443-01A-12R-A24H-07 | 3.203004154 | 3.295184425 |
| TCGA-55-7573-01A-11R-2039-07 | 3.248446389 | 3.225127164 |
| TCGA-78-7154-01A-11R-2039-07 | 3.436368449 | 3.315354579 |
| TCGA-44-3918-01B-02R-A277-07 | 2.811824959 | 2.884673523 |
| TCGA-S2-AA1A-01A-12R-A39D-07 | 3.251034392 | 3.272911498 |
| TCGA-05-5423-01A-01R-1628-07 | 3.211100717 | 3.224320011 |
| TCGA-50-6591-01A-11R-1755-07 | 3.072340257 | 3.314530193 |
| TCGA-MP-A4TE-01A-22R-A466-07 | 3.304091809 | 3.290092058 |
| TCGA-55-6543-01A-11R-1755-07 | 3.30774441 | 3.338726455 |
| TCGA-49-AAR3-01A-11R-A41B-07 | 3.308153117 | 3.245925774 |
| TCGA-38-4632-01A-01R-1755-07 | 3.242425004 | 3.303388836 |
| TCGA-62-A46S-01A-11R-A24H-07 | 3.21665624 | 3.298284464 |
| TCGA-99-8033-01A-11R-2241-07 | 3.28935835 | 3.3716202 |
| TCGA-55-6980-01A-11R-1949-07 | 3.268206115 | 3.438273723 |
| TCGA-05-4244-01A-01R-1107-07 | 3.229841067 | 3.270319698 |
| TCGA-91-8497-01A-11R-2403-07 | 3.260560914 | 3.292815753 |
| TCGA-64-1680-01A-02R-0946-07 | 3.232402099 | 3.187055789 |
| TCGA-86-7953-01A-11R-2187-07 | 3.332492617 | 3.383769006 |
| TCGA-55-8616-01A-11R-2403-07 | 3.169096391 | 3.257766408 |
| TCGA-86-8055-01A-11R-2241-07 | 3.315796798 | 3.473717027 |
| TCGA-73-4668-01A-01R-1206-07 | 3.256730879 | 3.387050126 |
| TCGA-55-6982-01A-11R-1949-07 | 3.322696439 | 3.366194961 |
| TCGA-67-6217-01A-11R-1755-07 | 3.18744371 | 3.205690019 |
| TCGA-44-6146-01A-11R-A278-07 | 3.210996249 | 3.153546646 |
| TCGA-49-4512-01A-21R-1858-07 | 3.182562782 | 3.248639439 |
| TCGA-64-5774-01A-01R-1628-07 | 3.249358847 | 3.406167081 |
| TCGA-97-8177-01A-11R-2287-07 | 3.289226429 | 3.391021081 |
| TCGA-78-8640-01A-11R-2403-07 | 3.325163723 | 3.246990243 |
| TCGA-95-7947-01A-11R-2187-07 | 3.264062494 | 3.202668132 |
| TCGA-44-6777-01A-11R-1858-07 | 3.286682754 | 3.483534209 |
| TCGA-55-7816-01A-11R-2170-07 | 3.225723871 | 3.263567767 |
| TCGA-49-AAR4-01A-12R-A41B-07 | 3.198716316 | 3.27927394 |
| TCGA-55-7907-01A-11R-2170-07 | 3.222007946 | 3.260983968 |
| TCGA-86-8359-01A-11R-2326-07 | 3.289686198 | 3.378553099 |
| TCGA-50-5051-01A-21R-1858-07 | 3.301539258 | 3.336600231 |
| TCGA-05-4402-01A-01R-1206-07 | 3.269805324 | 3.352773413 |
| TCGA-50-8459-01A-11R-2326-07 | 3.2951842 | 3.44656362 |
| TCGA-NJ-A7XG-01A-12R-A39D-07 | 3.183387818 | 3.162979352 |
| TCGA-49-4488-01A-01R-1755-07 | 3.291883243 | 3.294074311 |
| TCGA-64-5815-01A-01R-1628-07 | 3.318244695 | 3.532480966 |
| TCGA-86-8056-01A-11R-2241-07 | 3.281851723 | 3.220632104 |
| TCGA-78-7146-01A-11R-2039-07 | 3.327154047 | 3.369682136 |
| TCGA-91-8499-01A-11R-2403-07 | 3.258418728 | 3.263420507 |
| TCGA-55-6970-01A-11R-1949-07 | 3.339039043 | 3.381663944 |
| TCGA-05-4420-01A-01R-1206-07 | 3.250857574 | 3.235418696 |
| TCGA-86-7701-01A-11R-2170-07 | 3.286914529 | 3.369164414 |
| TCGA-NJ-A55R-01A-11R-A262-07 | 3.24443126 | 3.314141002 |
| TCGA-05-4397-01A-01R-1206-07 | 3.24695882 | 3.321911324 |
| TCGA-53-7626-01A-12R-2066-07 | 3.230713908 | 3.323832443 |
| TCGA-95-7944-01A-11R-2187-07 | 3.147373549 | 3.254841652 |
| TCGA-44-2656-01B-06R-A277-07 | 2.875748497 | 2.853074046 |
| TCGA-73-A9RS-01A-11R-A41B-07 | 3.272576877 | 3.182683959 |
| TCGA-44-7667-01A-31R-2066-07 | 3.237556683 | 3.181815771 |
| TCGA-NJ-A55O-01A-11R-A262-07 | 3.229448536 | 3.312539649 |
| TCGA-55-8301-01A-11R-2287-07 | 3.237943503 | 3.318789066 |
| TCGA-05-4415-01A-22R-1858-07 | 3.241685152 | 3.341163725 |
| TCGA-91-6835-01A-11R-1858-07 | 3.265133165 | 3.26811051 |
| TCGA-55-7725-01A-11R-2170-07 | 3.143841258 | 3.15986794 |
| TCGA-55-8506-01A-11R-2403-07 | 3.206264268 | 3.279937508 |
| TCGA-50-5931-01A-11R-1755-07 | 3.274917953 | 3.395710284 |
| TCGA-44-5645-01A-01R-1628-07 | 3.190206063 | 3.175803702 |
| TCGA-55-A494-01A-11R-A24X-07 | 3.096803789 | 3.125374454 |
| TCGA-50-5930-01A-11R-1755-07 | 3.34509794 | 3.423785391 |
| TCGA-38-4627-01A-01R-1206-07 | 3.402868251 | 3.455368325 |
| TCGA-67-4679-01B-01R-1755-07 | 3.243093244 | 3.204090577 |
| TCGA-55-7903-01A-11R-2170-07 | 3.177298025 | 3.24874057 |
| TCGA-55-8619-01A-11R-2403-07 | 3.284059483 | 3.353571225 |
| TCGA-78-7159-01A-11R-2039-07 | 3.291134848 | 3.338114181 |
| TCGA-55-6971-01A-11R-1949-07 | 3.315543281 | 3.397626558 |
| TCGA-50-5933-01A-11R-1755-07 | 3.302052404 | 3.430456563 |
| TCGA-95-A4VK-01A-11R-A262-07 | 3.21071247 | 3.248035404 |
| TCGA-64-5779-01A-01R-1628-07 | 3.173267429 | 3.359886467 |
| TCGA-05-4410-01A-21R-1858-07 | 3.225360424 | 3.239713041 |
| TCGA-44-A47G-01A-21R-A24H-07 | 3.328527281 | 3.393791675 |
| TCGA-97-8179-01A-11R-2287-07 | 3.293468281 | 3.176846445 |
| TCGA-78-7155-01A-11R-2039-07 | 3.140525614 | 3.129669195 |
| TCGA-49-4505-01A-01R-1206-07 | 3.257880378 | 3.354838805 |
| TCGA-97-8552-01A-11R-2403-07 | 3.244903451 | 3.229437036 |
| TCGA-MP-A4TJ-01A-51R-A262-07 | 3.295436662 | 3.345431839 |
| TCGA-73-4670-01A-01R-1206-07 | 3.33774755 | 3.454065875 |
| TCGA-55-A493-01A-11R-A24H-07 | 3.216815652 | 3.403456832 |
| TCGA-69-7973-01A-11R-2187-07 | 3.312529685 | 3.345661517 |
| TCGA-50-5932-01A-11R-1755-07 | 3.204051179 | 3.193748161 |
| TCGA-05-4430-01A-02R-1206-07 | 3.299343963 | 3.334491289 |
| TCGA-49-4486-01A-01R-1206-07 | 3.214321052 | 3.182893009 |
| TCGA-78-8662-01A-11R-2403-07 | 3.23790588 | 3.174121829 |
| TCGA-95-8039-01A-11R-2241-07 | 3.24702867 | 3.236909772 |
| TCGA-MP-A4TA-01A-21R-A24X-07 | 3.30969705 | 3.223736985 |
| TCGA-55-7284-01B-11R-2241-07 | 3.309686178 | 3.346730667 |
| TCGA-69-7764-01A-11R-2170-07 | 3.147141257 | 3.182864989 |
| TCGA-44-A4SU-01A-11R-A24X-07 | 3.176102098 | 3.273952966 |
| TCGA-49-4506-01A-01R-1206-07 | 3.284643366 | 3.325211748 |
| TCGA-78-7161-01A-11R-2039-07 | 3.232428998 | 3.272187777 |
| TCGA-49-AAQV-01A-11R-A39D-07 | 3.251674841 | 3.32044719 |
| TCGA-62-A46P-01A-11R-A24H-07 | 3.184905951 | 3.254007675 |
| TCGA-49-6761-01A-31R-1949-07 | 3.237994709 | 3.374350051 |
| TCGA-44-3919-01A-02R-1107-07 | 3.245267169 | 3.320523316 |
| TCGA-69-7980-01A-11R-2187-07 | 3.229274555 | 3.271113883 |
| TCGA-93-7348-01A-21R-2039-07 | 3.264051623 | 3.351854034 |
| TCGA-75-7030-01A-11R-1949-07 | 3.3247075 | 3.477002112 |
| TCGA-86-8074-01A-11R-2241-07 | 3.294614825 | 3.375198742 |
| TCGA-49-6745-01A-11R-1858-07 | 3.31640646 | 3.380394852 |
| TCGA-38-4631-01A-01R-1755-07 | 3.335956723 | 3.274663071 |
| TCGA-38-4626-01A-01R-1206-07 | 3.334826331 | 3.393986386 |
| TCGA-44-6147-01B-06R-A277-07 | 2.73434044 | 2.889856289 |
| TCGA-55-7227-01A-11R-2039-07 | 3.22842983 | 3.287258358 |
| TCGA-55-A57B-01A-12R-A39D-07 | 3.24859531 | 3.270333519 |
| TCGA-69-7979-01A-11R-2187-07 | 3.303469444 | 3.256213228 |
| TCGA-44-A4SS-01A-11R-A24X-07 | 3.347354299 | 3.323930059 |
| TCGA-05-4424-01A-22R-1858-07 | 3.331530902 | 3.376634247 |
| TCGA-MN-A4N5-01A-11R-A24X-07 | 3.170684395 | 3.272758557 |
| TCGA-99-8028-01A-11R-2241-07 | 3.271038812 | 3.426749548 |
| TCGA-75-5146-01A-01R-1628-07 | 3.252258551 | 3.252773922 |
| TCGA-55-1596-01A-01R-0946-07 | 3.29677088 | 3.196303638 |
| TCGA-55-7910-01A-11R-2170-07 | 3.261757381 | 3.33911917 |
| TCGA-80-5611-01A-01R-1628-07 | 3.329030219 | 3.296087328 |
| TCGA-91-6828-01A-11R-1858-07 | 3.329363792 | 3.328059019 |
| TCGA-69-7761-01A-11R-2170-07 | 3.253760596 | 3.306555262 |
| TCGA-91-6849-01A-11R-1949-07 | 3.347566232 | 3.316811195 |
| TCGA-MP-A4TH-01A-31R-A262-07 | 3.146505591 | 3.182412487 |
| TCGA-44-7669-01A-21R-2066-07 | 3.350199545 | 3.335938149 |
| TCGA-44-2662-01A-01R-A278-07 | 3.2233554 | 3.38424931 |
| TCGA-73-7499-01A-11R-2187-07 | 3.250997151 | 3.205644919 |
| TCGA-55-8505-01A-11R-2403-07 | 3.420883337 | 3.466019956 |
| TCGA-86-8671-01A-11R-2403-07 | 3.249725919 | 3.295161575 |
| TCGA-95-7948-01A-11R-2187-07 | 3.108612845 | 3.131072025 |
| TCGA-95-7043-01A-11R-1949-07 | 3.336238942 | 3.146859725 |
| TCGA-50-5044-01A-21R-1858-07 | 3.285067271 | 3.358480449 |
| TCGA-J2-A4AG-01A-11R-A24H-07 | 3.207066415 | 3.346172866 |
| TCGA-86-7955-01A-11R-2187-07 | 3.192649904 | 3.191160477 |
| TCGA-78-7158-01A-11R-2039-07 | 3.142299107 | 3.0612993 |
| TCGA-05-5425-01A-02R-1628-07 | 3.313421719 | 3.350824882 |
| TCGA-91-6831-01A-11R-1858-07 | 3.267597445 | 3.357700368 |
| TCGA-97-8175-01A-11R-2287-07 | 3.260290946 | 3.401930348 |
| TCGA-86-8672-01A-21R-2403-07 | 3.332471145 | 3.31668544 |
| TCGA-44-5645-01A-01R-A278-07 | 3.064760343 | 3.01089425 |
| TCGA-50-6594-01A-11R-1755-07 | 3.248144761 | 3.252028436 |
| TCGA-86-7714-01A-12R-2170-07 | 3.240621219 | 3.195302415 |
| TCGA-86-6562-01A-11R-1755-07 | 3.257593978 | 3.384648272 |
| TCGA-55-8090-01A-11R-2241-07 | 3.337690852 | 3.370656704 |
| TCGA-55-7911-01A-11R-2170-07 | 3.185611722 | 3.141125881 |
| TCGA-55-8507-01A-11R-2403-07 | 3.228336133 | 3.314647552 |
| TCGA-93-8067-01A-11R-2287-07 | 3.406524254 | 3.361499101 |
| TCGA-44-3918-01A-01R-A278-07 | 3.078129937 | 3.121758389 |
| TCGA-97-7553-01A-21R-2039-07 | 3.31714052 | 3.290732997 |
| TCGA-55-8205-01A-11R-2241-07 | 3.243504684 | 3.272370058 |
| TCGA-05-4390-01A-02R-1755-07 | 3.334459712 | 3.376225541 |
| TCGA-86-8668-01A-11R-2403-07 | 3.236724292 | 3.273935766 |
| TCGA-62-8398-01A-11R-2326-07 | 3.376685964 | 3.403310586 |
| TCGA-55-8513-01A-11R-2403-07 | 3.328133047 | 3.282268911 |
| TCGA-83-5908-01A-21R-2287-07 | 3.298921822 | 3.291631946 |
| TCGA-05-4422-01A-01R-1206-07 | 3.264318228 | 3.174640923 |
| TCGA-44-2662-01A-01R-0946-07 | 3.280168153 | 3.464054935 |
| TCGA-50-5045-01A-01R-1628-07 | 3.256922211 | 3.381777116 |
| TCGA-44-6146-01B-04R-A277-07 | 2.826221624 | 2.832087915 |
| TCGA-44-4112-01B-06R-A277-07 | 2.847823924 | 2.985884718 |
| TCGA-91-6836-01A-21R-1858-07 | 3.175731513 | 3.347221862 |
| TCGA-55-6975-01A-11R-1949-07 | 3.299607653 | 3.347699418 |
| TCGA-55-A491-01A-11R-A24H-07 | 3.31251557 | 3.356641522 |
| TCGA-93-7347-01A-11R-2187-07 | 3.305291931 | 3.27205752 |
| TCGA-MP-A4T9-01A-11R-A24X-07 | 3.295825006 | 3.438112451 |
| TCGA-49-AAR9-01A-21R-A41B-07 | 3.25373799 | 3.3162991 |
| TCGA-75-6203-01A-11R-1755-07 | 3.277235281 | 3.335969452 |
| TCGA-55-8091-01A-11R-2241-07 | 3.373081352 | 3.398911654 |
| TCGA-97-7547-01A-11R-2039-07 | 3.245725786 | 3.252141791 |
| TCGA-05-4417-01A-22R-1858-07 | 3.353359171 | 3.397574002 |
| TCGA-62-A46R-01A-11R-A24H-07 | 3.160395987 | 3.148184473 |
| TCGA-49-4490-01A-21R-1858-07 | 3.167540618 | 3.341548196 |
| TCGA-05-4395-01A-01R-1206-07 | 3.264410803 | 3.337762443 |
| TCGA-62-A46Y-01A-11R-A24H-07 | 3.249397857 | 3.273668586 |
| TCGA-97-A4M7-01A-11R-A24X-07 | 3.266148682 | 3.293612186 |
| TCGA-35-4123-01A-01R-1107-07 | 3.184042673 | 3.366330581 |
| TCGA-55-7576-01A-11R-2066-07 | 3.210211858 | 3.253705949 |
| TCGA-64-5781-01A-01R-1628-07 | 3.298050135 | 3.363204393 |
| TCGA-L9-A50W-01A-12R-A39D-07 | 3.24063052 | 3.253758732 |
| TCGA-97-A4M5-01A-11R-A24X-07 | 3.330249889 | 3.294592126 |
| TCGA-35-4122-01A-01R-1107-07 | 3.299160978 | 3.356284213 |
| TCGA-55-A48Z-01A-12R-A24X-07 | 3.227998413 | 3.355572294 |
| TCGA-86-6851-01A-11R-1949-07 | 3.213573536 | 3.263553271 |
| TCGA-NJ-A4YF-01A-12R-A262-07 | 3.168904919 | 3.34157982 |
| TCGA-05-4389-01A-01R-1206-07 | 3.421451374 | 3.245533536 |
| TCGA-NJ-A4YQ-01A-11R-A262-07 | 3.229584274 | 3.221269261 |
| TCGA-55-6981-01A-11R-1949-07 | 3.285841406 | 3.288172953 |
| TCGA-97-7546-01A-11R-2039-07 | 3.203595474 | 3.257142731 |
| TCGA-MN-A4N1-01A-11R-A24X-07 | 3.199962635 | 3.234010318 |
| TCGA-62-A471-01A-12R-A24H-07 | 3.33933982 | 3.357091605 |
| TCGA-55-7570-01A-11R-2039-07 | 3.289179077 | 3.149602123 |
| TCGA-44-3917-01A-01R-A278-07 | 3.19754507 | 3.010599428 |
| TCGA-55-A4DF-01A-11R-A24H-07 | 3.379032919 | 3.35435063 |
| TCGA-50-5946-02A-11R-2090-07 | 3.205756738 | 3.354496246 |
| TCGA-78-8648-01A-11R-2403-07 | 3.294162714 | 3.463510914 |
| TCGA-67-3770-01A-01R-0946-07 | 3.292089475 | 3.272755263 |
| TCGA-50-5936-01A-11R-1628-07 | 3.337563685 | 3.407726879 |
| TCGA-67-6216-01A-11R-1755-07 | 3.242045416 | 3.293539498 |
| TCGA-69-7974-01A-11R-2187-07 | 3.303232762 | 3.354316397 |
| TCGA-86-8281-01A-11R-2287-07 | 3.287515487 | 3.200032438 |
| TCGA-44-2662-01B-02R-A277-07 | 2.872198539 | 3.069273957 |
| TCGA-93-A4JQ-01A-11R-A24X-07 | 3.226219488 | 3.31681166 |
| TCGA-55-8207-01A-11R-2241-07 | 3.225838442 | 3.363186995 |
| TCGA-97-8176-01A-11R-2403-07 | 3.367234209 | 3.287501209 |
| TCGA-86-8280-01A-11R-2287-07 | 3.27811609 | 3.313929174 |
| TCGA-MP-A4T8-01A-11R-A24X-07 | 3.224693179 | 3.346805529 |
| TCGA-55-A492-01A-11R-A24H-07 | 3.215228689 | 3.139959616 |
| TCGA-J2-8192-01A-11R-2241-07 | 3.255860787 | 3.405206921 |
| TCGA-50-8460-01A-11R-2326-07 | 3.245103501 | 3.244841941 |
| TCGA-80-5607-01A-31R-1949-07 | 3.333421286 | 3.373426994 |
| TCGA-NJ-A55A-01A-11R-A262-07 | 3.209482794 | 3.206261134 |
| TCGA-55-8097-01A-11R-2241-07 | 3.149003491 | 3.099490255 |
| TCGA-44-6147-01A-11R-1755-07 | 3.255487246 | 3.350105189 |
| TCGA-55-A490-01A-11R-A466-07 | 3.118889875 | 3.354506784 |
| TCGA-86-7711-01A-11R-2066-07 | 3.335004054 | 3.315758648 |
| TCGA-MP-A4TK-01A-11R-A24X-07 | 3.215964586 | 3.466376869 |
| TCGA-50-5066-01A-01R-1628-07 | 3.351640125 | 3.396857547 |
| TCGA-44-2666-01A-01R-A278-07 | 3.215584341 | 3.120722583 |
| TCGA-50-5942-01A-21R-1755-07 | 3.268934324 | 3.223013084 |
| TCGA-69-8453-01A-12R-2326-07 | 3.355483109 | 3.377048402 |
| TCGA-44-8119-01A-11R-2241-07 | 3.279977782 | 3.497848032 |

Supplementary Table 2

| probes | moduleColor |
| --- | --- |
| A2M-AS1 | turquoise |
| A4GALT | turquoise |
| AAK1 | turquoise |
| AAMDC | turquoise |
| AAMP | turquoise |
| AAR2 | turquoise |
| AASDH | turquoise |
| AASDHPPT | turquoise |
| AASS | turquoise |
| ABALON | turquoise |
| ABAT | turquoise |
| ABCA1 | turquoise |
| ABCA11P | turquoise |
| ABCA12 | turquoise |
| ABCA13 | turquoise |
| ABCA5 | turquoise |
| ABCA6 | turquoise |
| ABCA9 | turquoise |
| ABCB10 | turquoise |
| ABCB7 | turquoise |
| ABCC4 | turquoise |
| ABCC9 | turquoise |
| ABCD1 | turquoise |
| ABCD3 | turquoise |
| ABHD11-AS1 | turquoise |
| ABHD12 | turquoise |
| ABHD13 | turquoise |
| ABHD14A | turquoise |
| ABHD14B | turquoise |
| ABHD15 | turquoise |
| ABHD17B | turquoise |
| ABHD3 | turquoise |
| ABHD4 | turquoise |
| ABHD8 | turquoise |
| ABI1 | turquoise |
| ABI2 | turquoise |
| ABL2 | turquoise |
| ABLIM1 | turquoise |
| ABO | turquoise |
| AC000078.5 | turquoise |
| AC000123.2 | turquoise |
| AC000123.3 | turquoise |
| AC000123.4 | turquoise |
| AC002044.4 | turquoise |
| AC002064.4 | turquoise |
| AC002075.4 | turquoise |
| AC002128.5 | turquoise |
| AC002480.3 | turquoise |
| AC002550.6 | turquoise |
| AC003092.1 | turquoise |
| AC003973.3 | turquoise |
| AC004067.5 | turquoise |
| AC004069.2 | turquoise |
| AC004846.1 | turquoise |
| AC004893.11 | turquoise |
| AC004951.5 | turquoise |
| AC004967.7 | turquoise |
| AC004980.7 | turquoise |
| AC005042.1 | turquoise |
| AC005052.1 | turquoise |
| AC005071.3 | turquoise |
| AC005071.4 | turquoise |
| AC005082.12 | turquoise |
| AC005104.3 | turquoise |
| AC005154.6 | turquoise |
| AC005154.7 | turquoise |
| AC005154.8 | turquoise |
| AC005253.2 | turquoise |
| AC005255.3 | turquoise |
| AC005517.3 | turquoise |
| AC005519.4 | turquoise |
| AC005540.3 | turquoise |
| AC005682.5 | turquoise |
| AC005682.6 | turquoise |
| AC005702.1 | turquoise |
| AC005785.5 | turquoise |
| AC006042.6 | turquoise |
| AC006077.3 | turquoise |
| AC006378.2 | turquoise |
| AC006539.1 | turquoise |
| AC006547.13 | turquoise |
| AC006978.6 | turquoise |
| AC007000.11 | turquoise |
| AC007009.2 | turquoise |
| AC007038.7 | turquoise |
| AC007191.4 | turquoise |
| AC007228.9 | turquoise |
| AC007246.3 | turquoise |
| AC007283.4 | turquoise |
| AC007319.1 | turquoise |
| AC007387.2 | turquoise |
| AC007528.1 | turquoise |
| AC007528.2 | turquoise |
| AC007566.10 | turquoise |
| AC007773.1 | turquoise |
| AC007879.7 | turquoise |
| AC007919.18 | turquoise |
| AC008063.2 | turquoise |
| AC008746.12 | turquoise |
| AC008746.5 | turquoise |
| AC008982.2 | turquoise |
| AC009120.3 | turquoise |
| AC009120.5 | turquoise |
| AC009120.6 | turquoise |
| AC009237.11 | turquoise |
| AC009237.8 | turquoise |
| AC009245.3 | turquoise |
| AC009299.3 | turquoise |
| AC009303.2 | turquoise |
| AC009403.2 | turquoise |
| AC009948.5 | turquoise |
| AC010095.5 | turquoise |
| AC010226.4 | turquoise |
| AC010504.2 | turquoise |
| AC010733.5 | turquoise |
| AC010761.13 | turquoise |
| AC010761.8 | turquoise |
| AC010883.5 | turquoise |
| AC010970.2 | turquoise |
| AC010999.1 | turquoise |
| AC011290.5 | turquoise |
| AC011513.4 | turquoise |
| AC012358.4 | turquoise |
| AC012363.2 | turquoise |
| AC013275.2 | turquoise |
| AC015849.19 | turquoise |
| AC016656.1 | turquoise |
| AC016831.7 | turquoise |
| AC017060.1 | turquoise |
| AC017074.2 | turquoise |
| AC017104.3 | turquoise |
| AC018738.2 | turquoise |
| AC018816.3 | turquoise |
| AC018867.1 | turquoise |
| AC018902.1 | turquoise |
| AC019097.7 | turquoise |
| AC019117.1 | turquoise |
| AC019117.2 | turquoise |
| AC020571.3 | turquoise |
| AC020594.5 | turquoise |
| AC021087.1 | turquoise |
| AC022173.2 | turquoise |
| AC024060.1 | turquoise |
| AC024361.1 | turquoise |
| AC024560.3 | turquoise |
| AC024937.6 | turquoise |
| AC025171.1 | turquoise |
| AC025918.1 | turquoise |
| AC026202.3 | turquoise |
| AC026471.6 | turquoise |
| AC034220.3 | turquoise |
| AC044907.1 | turquoise |
| AC046143.3 | turquoise |
| AC055866.1 | turquoise |
| AC058791.1 | turquoise |
| AC063932.1 | turquoise |
| AC068580.6 | turquoise |
| AC068641.1 | turquoise |
| AC069063.1 | turquoise |
| AC069257.6 | turquoise |
| AC069282.6 | turquoise |
| AC069286.1 | turquoise |
| AC073046.25 | turquoise |
| AC073063.10 | turquoise |
| AC073130.2 | turquoise |
| AC073415.2 | turquoise |
| AC073635.5 | turquoise |
| AC074289.1 | turquoise |
| AC079781.5 | turquoise |
| AC079907.1 | turquoise |
| AC079949.1 | turquoise |
| AC083843.1 | turquoise |
| AC083843.4 | turquoise |
| AC083900.1 | turquoise |
| AC083949.1 | turquoise |
| AC084117.3 | turquoise |
| AC084357.1 | turquoise |
| AC087350.1 | turquoise |
| AC087793.1 | turquoise |
| AC087884.1 | turquoise |
| AC090154.1 | turquoise |
| AC090181.1 | turquoise |
| AC090587.5 | turquoise |
| AC091180.1 | turquoise |
| AC091492.2 | turquoise |
| AC092574.2 | turquoise |
| AC093162.5 | turquoise |
| AC093375.1 | turquoise |
| AC093495.4 | turquoise |
| AC093627.10 | turquoise |
| AC093642.5 | turquoise |
| AC093673.5 | turquoise |
| AC093724.2 | turquoise |
| AC093788.1 | turquoise |
| AC093818.1 | turquoise |
| AC096582.7 | turquoise |
| AC098592.7 | turquoise |
| AC098614.2 | turquoise |
| AC100821.2 | turquoise |
| AC100830.5 | turquoise |
| AC104534.2 | turquoise |
| AC104650.2 | turquoise |
| AC104841.1 | turquoise |
| AC104986.1 | turquoise |
| AC105009.1 | turquoise |
| AC107072.2 | turquoise |
| AC107983.4 | turquoise |
| AC108456.1 | turquoise |
| AC108463.2 | turquoise |
| AC109333.10 | turquoise |
| AC110602.1 | turquoise |
| AC112229.1 | turquoise |
| AC113189.5 | turquoise |
| AC114494.1 | turquoise |
| AC116366.6 | turquoise |
| AC117507.1 | turquoise |
| AC118344.1 | turquoise |
| AC122718.1 | turquoise |
| AC124074.1 | turquoise |
| AC124914.3 | turquoise |
| AC125232.1 | turquoise |
| AC131160.2 | turquoise |
| AC131263.1 | turquoise |
| AC133644.2 | turquoise |
| AC137932.4 | turquoise |
| AC137932.5 | turquoise |
| AC139100.4 | turquoise |
| AC139530.1 | turquoise |
| AC139887.4 | turquoise |
| AC141586.5 | turquoise |
| AC144450.1 | turquoise |
| AC144530.1 | turquoise |
| AC234582.1 | turquoise |
| AC242988.1 | turquoise |
| AC253572.1 | turquoise |
| AC253576.2 | turquoise |
| ACAA2 | turquoise |
| ACACA | turquoise |
| ACADM | turquoise |
| ACADS | turquoise |
| ACADSB | turquoise |
| ACAP2 | turquoise |
| ACAP2-IT1 | turquoise |
| ACAT1 | turquoise |
| ACBD3 | turquoise |
| ACBD5 | turquoise |
| ACD | turquoise |
| ACER3 | turquoise |
| ACKR2 | turquoise |
| ACKR3 | turquoise |
| ACMSD | turquoise |
| ACO1 | turquoise |
| ACO2 | turquoise |
| ACOT1 | turquoise |
| ACOT11 | turquoise |
| ACOT2 | turquoise |
| ACOT4 | turquoise |
| ACOX1 | turquoise |
| ACP2 | turquoise |
| ACP6 | turquoise |
| ACPP | turquoise |
| ACRC | turquoise |
| ACSL1 | turquoise |
| ACSL3 | turquoise |
| ACTA2-AS1 | turquoise |
| ACTB | turquoise |
| ACTBP12 | turquoise |
| ACTG1 | turquoise |
| ACTN4 | turquoise |
| ACTR1A | turquoise |
| ACTR1B | turquoise |
| ACTR2 | turquoise |
| ACTR3C | turquoise |
| ACTR8 | turquoise |
| ACTRT3 | turquoise |
| ACVR1 | turquoise |
| ACVR2A | turquoise |
| ACVR2B | turquoise |
| ACVR2B-AS1 | turquoise |
| ACYP1 | turquoise |
| ACYP2 | turquoise |
| AD000092.3 | turquoise |
| AD000684.2 | turquoise |
| ADAL | turquoise |
| ADAM10 | turquoise |
| ADAM15 | turquoise |
| ADAM17 | turquoise |
| ADAM28 | turquoise |
| ADAM9 | turquoise |
| ADAMTS3 | turquoise |
| ADAMTS5 | turquoise |
| ADAMTS9 | turquoise |
| ADAMTSL4-AS1 | turquoise |
| ADAR | turquoise |
| ADAT1 | turquoise |
| ADAT2 | turquoise |
| ADCK2 | turquoise |
| ADCY10P1 | turquoise |
| ADD3 | turquoise |
| ADI1 | turquoise |
| ADIPOR1 | turquoise |
| ADIPOR2 | turquoise |
| ADNP | turquoise |
| ADNP-AS1 | turquoise |
| ADNP2 | turquoise |
| ADO | turquoise |
| ADORA1 | turquoise |
| ADORA2B | turquoise |
| ADPGK | turquoise |
| ADPRHL2 | turquoise |
| ADRBK2 | turquoise |
| ADRM1 | turquoise |
| AEBP2 | turquoise |
| AES | turquoise |
| AF011889.2 | turquoise |
| AF129075.5 | turquoise |
| AF131215.2 | turquoise |
| AF131215.9 | turquoise |
| AF178030.2 | turquoise |
| AF186192.1 | turquoise |
| AF186192.5 | turquoise |
| AFAP1 | turquoise |
| AFAP1L2 | turquoise |
| AFF1 | turquoise |
| AFF4 | turquoise |
| AFTPH | turquoise |
| AGAP1 | turquoise |
| AGAP1-IT1 | turquoise |
| AGAP2-AS1 | turquoise |
| AGAP4 | turquoise |
| AGBL5-IT1 | turquoise |
| AGFG1 | turquoise |
| AGFG2 | turquoise |
| AGGF1 | turquoise |
| AGK | turquoise |
| AGL | turquoise |
| AGO1 | turquoise |
| AGO2 | turquoise |
| AGO3 | turquoise |
| AGO4 | turquoise |
| AGPAT1 | turquoise |
| AGPAT2 | turquoise |
| AGPS | turquoise |
| AGTPBP1 | turquoise |
| AGTRAP | turquoise |
| AHCTF1 | turquoise |
| AHCYL1 | turquoise |
| AHI1 | turquoise |
| AHR | turquoise |
| AHSA1 | turquoise |
| AIDA | turquoise |
| AIF1L | turquoise |
| AIM1 | turquoise |
| AIM1L | turquoise |
| AIP | turquoise |
| AJUBA | turquoise |
| AK3 | turquoise |
| AK3P5 | turquoise |
| AK9 | turquoise |
| AKAP10 | turquoise |
| AKAP11 | turquoise |
| AKAP13 | turquoise |
| AKAP5 | turquoise |
| AKAP6 | turquoise |
| AKAP7 | turquoise |
| AKAP9 | turquoise |
| AKIP1 | turquoise |
| AKR1A1 | turquoise |
| AKR1B1 | turquoise |
| AKR1E2 | turquoise |
| AKR7A2 | turquoise |
| AKT1 | turquoise |
| AKT1S1 | turquoise |
| AKT3 | turquoise |
| AKTIP | turquoise |
| AL022326.1 | turquoise |
| AL031587.1 | turquoise |
| AL031594.1 | turquoise |
| AL049872.1 | turquoise |
| AL109761.5 | turquoise |
| AL121584.1 | turquoise |
| AL132988.1 | turquoise |
| AL133243.1 | turquoise |
| AL136303.1 | turquoise |
| AL138706.2 | turquoise |
| AL138751.1 | turquoise |
| AL138898.1 | turquoise |
| AL138994.1 | turquoise |
| AL139333.1 | turquoise |
| AL160011.1 | turquoise |
| AL357515.1 | turquoise |
| AL359753.1 | turquoise |
| AL360001.1 | turquoise |
| AL360091.1 | turquoise |
| AL360176.1 | turquoise |
| AL390877.1 | turquoise |
| AL391994.1 | turquoise |
| AL513327.1 | turquoise |
| AL513548.1 | turquoise |
| AL589863.1 | turquoise |
| AL590431.1 | turquoise |
| AL590762.11 | turquoise |
| AL662800.1 | turquoise |
| AL713999.1 | turquoise |
| ALAD | turquoise |
| ALAS1 | turquoise |
| ALCAM | turquoise |
| ALDH16A1 | turquoise |
| ALDH1A2 | turquoise |
| ALDH1A3 | turquoise |
| ALDH1L2 | turquoise |
| ALDH3B1 | turquoise |
| ALDH4A1 | turquoise |
| ALDH5A1 | turquoise |
| ALDH6A1 | turquoise |
| ALDH9A1 | turquoise |
| ALDOC | turquoise |
| ALG1 | turquoise |
| ALG10 | turquoise |
| ALG10B | turquoise |
| ALG11 | turquoise |
| ALG13 | turquoise |
| ALG13-AS1 | turquoise |
| ALG1L6P | turquoise |
| ALG2 | turquoise |
| ALG6 | turquoise |
| ALG9 | turquoise |
| ALKBH1 | turquoise |
| ALKBH3 | turquoise |
| ALKBH4 | turquoise |
| ALKBH5 | turquoise |
| ALKBH8 | turquoise |
| ALMS1 | turquoise |
| ALMS1-IT1 | turquoise |
| ALOX12 | turquoise |
| ALOX12-AS1 | turquoise |
| ALOX12P2 | turquoise |
| ALPK1 | turquoise |
| ALPK3 | turquoise |
| ALPPL2 | turquoise |
| ALS2 | turquoise |
| AMBRA1 | turquoise |
| AMD1 | turquoise |
| AMER1 | turquoise |
| AMIGO2 | turquoise |
| AMMECR1 | turquoise |
| AMMECR1L | turquoise |
| AMN1 | turquoise |
| AMOT | turquoise |
| AMPD3 | turquoise |
| AMY2B | turquoise |
| ANAPC1 | turquoise |
| ANAPC10 | turquoise |
| ANAPC15 | turquoise |
| ANAPC4 | turquoise |
| ANG | turquoise |
| ANGEL1 | turquoise |
| ANGEL2 | turquoise |
| ANGPT2 | turquoise |
| ANGPTL1 | turquoise |
| ANGPTL4 | turquoise |
| ANK2 | turquoise |
| ANK3 | turquoise |
| ANKAR | turquoise |
| ANKDD1A | turquoise |
| ANKDD1B | turquoise |
| ANKFY1 | turquoise |
| ANKH | turquoise |
| ANKHD1 | turquoise |
| ANKIB1 | turquoise |
| ANKLE2 | turquoise |
| ANKMY2 | turquoise |
| ANKRA2 | turquoise |
| ANKRD10 | turquoise |
| ANKRD10-IT1 | turquoise |
| ANKRD11 | turquoise |
| ANKRD12 | turquoise |
| ANKRD13A | turquoise |
| ANKRD13C | turquoise |
| ANKRD17 | turquoise |
| ANKRD18EP | turquoise |
| ANKRD26 | turquoise |
| ANKRD28 | turquoise |
| ANKRD32 | turquoise |
| ANKRD34B | turquoise |
| ANKRD36 | turquoise |
| ANKRD36BP2 | turquoise |
| ANKRD36C | turquoise |
| ANKRD40 | turquoise |
| ANKRD44 | turquoise |
| ANKRD44-IT1 | turquoise |
| ANKRD46 | turquoise |
| ANKRD49 | turquoise |
| ANKRD50 | turquoise |
| ANKRD6 | turquoise |
| ANKRD61 | turquoise |
| ANKRD9 | turquoise |
| ANO10 | turquoise |
| ANO5 | turquoise |
| ANO7 | turquoise |
| ANP32B | turquoise |
| ANTXR2 | turquoise |
| ANXA11 | turquoise |
| ANXA2P1 | turquoise |
| ANXA2R | turquoise |
| AP000240.9 | turquoise |
| AP000254.8 | turquoise |
| AP000351.3 | turquoise |
| AP000442.1 | turquoise |
| AP000487.6 | turquoise |
| AP000580.1 | turquoise |
| AP000648.5 | turquoise |
| AP000692.9 | turquoise |
| AP000704.5 | turquoise |
| AP000807.1 | turquoise |
| AP000807.2 | turquoise |
| AP000866.1 | turquoise |
| AP000908.1 | turquoise |
| AP000936.1 | turquoise |
| AP000944.1 | turquoise |
| AP001052.1 | turquoise |
| AP001107.1 | turquoise |
| AP001205.1 | turquoise |
| AP001258.4 | turquoise |
| AP001271.3 | turquoise |
| AP001429.1 | turquoise |
| AP001432.14 | turquoise |
| AP001434.2 | turquoise |
| AP001469.7 | turquoise |
| AP001469.9 | turquoise |
| AP001625.6 | turquoise |
| AP001626.1 | turquoise |
| AP001628.6 | turquoise |
| AP002495.1 | turquoise |
| AP006621.6 | turquoise |
| AP1AR | turquoise |
| AP1B1 | turquoise |
| AP1G1 | turquoise |
| AP1M1 | turquoise |
| AP1M2 | turquoise |
| AP1S1 | turquoise |
| AP1S2 | turquoise |
| AP1S3 | turquoise |
| AP2A1 | turquoise |
| AP2M1 | turquoise |
| AP2S1 | turquoise |
| AP3B1 | turquoise |
| AP3M1 | turquoise |
| AP3M2 | turquoise |
| AP3S1 | turquoise |
| AP3S2 | turquoise |
| AP4E1 | turquoise |
| AP4M1 | turquoise |
| AP4S1 | turquoise |
| AP5B1 | turquoise |
| AP5M1 | turquoise |
| APAF1 | turquoise |
| APBA1 | turquoise |
| APBB2 | turquoise |
| APC | turquoise |
| APEH | turquoise |
| APEX2 | turquoise |
| APH1A | turquoise |
| APH1B | turquoise |
| API5 | turquoise |
| APMAP | turquoise |
| APOBEC2 | turquoise |
| APOD | turquoise |
| APOL1 | turquoise |
| APOL6 | turquoise |
| APOOL | turquoise |
| APP | turquoise |
| APPBP2 | turquoise |
| APPL1 | turquoise |
| APPL2 | turquoise |
| APTR | turquoise |
| AQP11 | turquoise |
| AQR | turquoise |
| ARAF | turquoise |
| ARAP2 | turquoise |
| AREG | turquoise |
| AREL1 | turquoise |
| ARF1 | turquoise |
| ARF3 | turquoise |
| ARF6 | turquoise |
| ARFGAP2 | turquoise |
| ARFGEF1 | turquoise |
| ARFGEF2 | turquoise |
| ARFIP1 | turquoise |
| ARHGAP1 | turquoise |
| ARHGAP10 | turquoise |
| ARHGAP11B | turquoise |
| ARHGAP12 | turquoise |
| ARHGAP15 | turquoise |
| ARHGAP17 | turquoise |
| ARHGAP19 | turquoise |
| ARHGAP20 | turquoise |
| ARHGAP21 | turquoise |
| ARHGAP26 | turquoise |
| ARHGAP28 | turquoise |
| ARHGAP29 | turquoise |
| ARHGAP31-AS1 | turquoise |
| ARHGAP32 | turquoise |
| ARHGAP35 | turquoise |
| ARHGAP42 | turquoise |
| ARHGAP5 | turquoise |
| ARHGAP5-AS1 | turquoise |
| ARHGAP8 | turquoise |
| ARHGDIA | turquoise |
| ARHGEF12 | turquoise |
| ARHGEF26-AS1 | turquoise |
| ARHGEF28 | turquoise |
| ARHGEF34P | turquoise |
| ARHGEF38 | turquoise |
| ARHGEF38-IT1 | turquoise |
| ARHGEF7 | turquoise |
| ARHGEF9 | turquoise |
| ARID1A | turquoise |
| ARID1B | turquoise |
| ARID2 | turquoise |
| ARID4A | turquoise |
| ARID4B | turquoise |
| ARID5B | turquoise |
| ARIH1 | turquoise |
| ARIH2 | turquoise |
| ARL10 | turquoise |
| ARL13B | turquoise |
| ARL14EP | turquoise |
| ARL15 | turquoise |
| ARL2 | turquoise |
| ARL4A | turquoise |
| ARL4D | turquoise |
| ARL5A | turquoise |
| ARL5B | turquoise |
| ARL6 | turquoise |
| ARL8A | turquoise |
| ARL8B | turquoise |
| ARMC6 | turquoise |
| ARMC7 | turquoise |
| ARMC8 | turquoise |
| ARMCX2 | turquoise |
| ARMCX3 | turquoise |
| ARMCX4 | turquoise |
| ARMCX5 | turquoise |
| ARMCX6 | turquoise |
| ARNT | turquoise |
| ARNT2 | turquoise |
| ARNTL | turquoise |
| ARPC1B | turquoise |
| ARPC3P1 | turquoise |
| ARPC4 | turquoise |
| ARPC5L | turquoise |
| ARPIN | turquoise |
| ARPP19 | turquoise |
| ARRDC3 | turquoise |
| ARSA | turquoise |
| ARSB | turquoise |
| ARSG | turquoise |
| ARSJ | turquoise |
| ARSK | turquoise |
| ARX | turquoise |
| ASAH2B | turquoise |
| ASAP1 | turquoise |
| ASB1 | turquoise |
| ASB13 | turquoise |
| ASB7 | turquoise |
| ASCC2 | turquoise |
| ASCC3 | turquoise |
| ASCL5 | turquoise |
| ASH1L | turquoise |
| ASH1L-AS1 | turquoise |
| ASL | turquoise |
| ASLP1 | turquoise |
| ASMTL | turquoise |
| ASPH | turquoise |
| ASS1 | turquoise |
| ASTE1 | turquoise |
| ASXL1 | turquoise |
| ASXL2 | turquoise |
| ATAD1 | turquoise |
| ATAD2B | turquoise |
| ATAD5 | turquoise |
| ATE1 | turquoise |
| ATF2 | turquoise |
| ATF4 | turquoise |
| ATF6 | turquoise |
| ATF6B | turquoise |
| ATF7 | turquoise |
| ATF7IP | turquoise |
| ATF7IP2 | turquoise |
| ATG10 | turquoise |
| ATG101 | turquoise |
| ATG12 | turquoise |
| ATG13 | turquoise |
| ATG14 | turquoise |
| ATG2B | turquoise |
| ATG4C | turquoise |
| ATG4D | turquoise |
| ATG7 | turquoise |
| ATG9A | turquoise |
| ATL2 | turquoise |
| ATL3 | turquoise |
| ATM | turquoise |
| ATMIN | turquoise |
| ATN1 | turquoise |
| ATP10B | turquoise |
| ATP10D | turquoise |
| ATP11A | turquoise |
| ATP11B | turquoise |
| ATP11C | turquoise |
| ATP12A | turquoise |
| ATP13A2 | turquoise |
| ATP13A3 | turquoise |
| ATP13A4 | turquoise |
| ATP13A4-AS1 | turquoise |
| ATP1A1 | turquoise |
| ATP1A1-AS1 | turquoise |
| ATP1B3-AS1 | turquoise |
| ATP2B1 | turquoise |
| ATP2B4 | turquoise |
| ATP2C1 | turquoise |
| ATP5A1 | turquoise |
| ATP5HP4 | turquoise |
| ATP5S | turquoise |
| ATP5SL | turquoise |
| ATP6AP1 | turquoise |
| ATP6AP1L | turquoise |
| ATP6V0A2 | turquoise |
| ATP6V0C | turquoise |
| ATP6V0D1 | turquoise |
| ATP6V0E1 | turquoise |
| ATP6V0E1P1 | turquoise |
| ATP6V0E2 | turquoise |
| ATP6V1A | turquoise |
| ATP6V1C1 | turquoise |
| ATP6V1E1 | turquoise |
| ATP7A | turquoise |
| ATP7B | turquoise |
| ATP8A1 | turquoise |
| ATP8A2 | turquoise |
| ATP8B1 | turquoise |
| ATP8B2 | turquoise |
| ATP8B4 | turquoise |
| ATP9A | turquoise |
| ATP9B | turquoise |
| ATPIF1 | turquoise |
| ATR | turquoise |
| ATRN | turquoise |
| ATRX | turquoise |
| ATXN1 | turquoise |
| ATXN2 | turquoise |
| ATXN3 | turquoise |
| ATXN7 | turquoise |
| ATXN7L1 | turquoise |
| AUH | turquoise |
| AUTS2 | turquoise |
| AVL9 | turquoise |
| AVPI1 | turquoise |
| AZGP1 | turquoise |
| AZI2 | turquoise |
| AZIN1 | turquoise |
| B3GALNT2 | turquoise |
| B3GALT2 | turquoise |
| B3GALT4 | turquoise |
| B3GALTL | turquoise |
| B3GAT3 | turquoise |
| B3GNT5 | turquoise |
| B3GNT7 | turquoise |
| B3GNT9 | turquoise |
| B4GALT6 | turquoise |
| B4GALT7 | turquoise |
| BAALC | turquoise |
| BAALC-AS1 | turquoise |
| BAAT | turquoise |
| BACH1 | turquoise |
| BACH2 | turquoise |
| BAD | turquoise |
| BAG1 | turquoise |
| BAG3 | turquoise |
| BAG4 | turquoise |
| BAG5 | turquoise |
| BAG6 | turquoise |
| BAHD1 | turquoise |
| BAK1 | turquoise |
| BANF1P3 | turquoise |
| BANP | turquoise |
| BAP1 | turquoise |
| BARD1 | turquoise |
| BATF | turquoise |
| BAX | turquoise |
| BAZ1A | turquoise |
| BAZ1B | turquoise |
| BAZ2A | turquoise |
| BAZ2B | turquoise |
| BBIP1 | turquoise |
| BBS10 | turquoise |
| BBS2 | turquoise |
| BBS7 | turquoise |
| BBS9 | turquoise |
| BBX | turquoise |
| BCAM | turquoise |
| BCAP29 | turquoise |
| BCAP31 | turquoise |
| BCAR1 | turquoise |
| BCAS3 | turquoise |
| BCAT1 | turquoise |
| BCAT2 | turquoise |
| BCKDHA | turquoise |
| BCKDHB | turquoise |
| BCKDK | turquoise |
| BCL10 | turquoise |
| BCL11A | turquoise |
| BCL11B | turquoise |
| BCL2 | turquoise |
| BCL2L1 | turquoise |
| BCL2L11 | turquoise |
| BCL3 | turquoise |
| BCL6 | turquoise |
| BCL7B | turquoise |
| BCL9L | turquoise |
| BCLAF1 | turquoise |
| BCOR | turquoise |
| BDNF-AS | turquoise |
| BDP1 | turquoise |
| BEND3 | turquoise |
| BEND7 | turquoise |
| BET1 | turquoise |
| BET1L | turquoise |
| BEX2 | turquoise |
| BFAR | turquoise |
| BHLHB9 | turquoise |
| BHLHE40 | turquoise |
| BICC1 | turquoise |
| BICD1 | turquoise |
| BIN1 | turquoise |
| BIRC2 | turquoise |
| BIRC3 | turquoise |
| BIRC6 | turquoise |
| BIVM | turquoise |
| BLACAT1 | turquoise |
| BLOC1S3 | turquoise |
| BLOC1S5 | turquoise |
| BLOC1S6 | turquoise |
| BLVRA | turquoise |
| BLVRB | turquoise |
| BLZF1 | turquoise |
| BMI1 | turquoise |
| BMP2K | turquoise |
| BMP3 | turquoise |
| BMP4 | turquoise |
| BMP5 | turquoise |
| BMP8A | turquoise |
| BMPR1A | turquoise |
| BMPR1B | turquoise |
| BMPR2 | turquoise |
| BMS1P1 | turquoise |
| BNC2 | turquoise |
| BNIP2 | turquoise |
| BNIP3L | turquoise |
| BNIP3P11 | turquoise |
| BNIP3P17 | turquoise |
| BNIP3P40 | turquoise |
| BOD1L1 | turquoise |
| BOK | turquoise |
| BPTF | turquoise |
| BRAF | turquoise |
| BRAP | turquoise |
| BRCA2 | turquoise |
| BRCC3 | turquoise |
| BRD3 | turquoise |
| BRD7 | turquoise |
| BRDT | turquoise |
| BRE | turquoise |
| BRI3 | turquoise |
| BRINP1 | turquoise |
| BRK1 | turquoise |
| BRMS1L | turquoise |
| BROX | turquoise |
| BRWD1 | turquoise |
| BRWD3 | turquoise |
| BSCL2 | turquoise |
| BSG | turquoise |
| BST2 | turquoise |
| BTAF1 | turquoise |
| BTBD1 | turquoise |
| BTBD10 | turquoise |
| BTBD19 | turquoise |
| BTBD2 | turquoise |
| BTBD3 | turquoise |
| BTBD6 | turquoise |
| BTBD7 | turquoise |
| BTBD9 | turquoise |
| BTBD9-AS1 | turquoise |
| BTC | turquoise |
| BTLA | turquoise |
| BTN2A3P | turquoise |
| BTRC | turquoise |
| BVES | turquoise |
| BX322557.10 | turquoise |
| BX470102.3 | turquoise |
| C10orf11 | turquoise |
| C10orf12 | turquoise |
| C10orf55 | turquoise |
| C10orf76 | turquoise |
| C11orf30 | turquoise |
| C11orf54 | turquoise |
| C11orf57 | turquoise |
| C11orf68 | turquoise |
| C11orf80 | turquoise |
| C11orf95 | turquoise |
| C12orf10 | turquoise |
| C12orf4 | turquoise |
| C12orf66 | turquoise |
| C12orf76 | turquoise |
| C14orf1 | turquoise |
| C14orf159 | turquoise |
| C14orf28 | turquoise |
| C14orf37 | turquoise |
| C15orf39 | turquoise |
| C15orf40 | turquoise |
| C15orf41 | turquoise |
| C15orf57 | turquoise |
| C15orf62 | turquoise |
| C16orf52 | turquoise |
| C16orf58 | turquoise |
| C16orf62 | turquoise |
| C16orf70 | turquoise |
| C16orf72 | turquoise |
| C16orf87 | turquoise |
| C17orf100 | turquoise |
| C17orf51 | turquoise |
| C17orf59 | turquoise |
| C17orf67 | turquoise |
| C17orf85 | turquoise |
| C18orf25 | turquoise |
| C18orf54 | turquoise |
| C18orf8 | turquoise |
| C19orf12 | turquoise |
| C19orf25 | turquoise |
| C19orf33 | turquoise |
| C19orf43 | turquoise |
| C19orf47 | turquoise |
| C19orf52 | turquoise |
| C19orf54 | turquoise |
| C19orf60 | turquoise |
| C19orf73 | turquoise |
| C1GALT1 | turquoise |
| C1orf132 | turquoise |
| C1orf186 | turquoise |
| C1orf210 | turquoise |
| C1orf216 | turquoise |
| C1orf27 | turquoise |
| C1orf52 | turquoise |
| C1orf95 | turquoise |
| C1QL3 | turquoise |
| C1RL | turquoise |
| C1RL-AS1 | turquoise |
| C2 | turquoise |
| C20orf197 | turquoise |
| C20orf27 | turquoise |
| C21orf91 | turquoise |
| C2CD2 | turquoise |
| C2CD3 | turquoise |
| C2CD5 | turquoise |
| C2orf27A | turquoise |
| C2orf42 | turquoise |
| C2orf43 | turquoise |
| C2orf49 | turquoise |
| C2orf69 | turquoise |
| C2orf88 | turquoise |
| C3orf17 | turquoise |
| C3orf38 | turquoise |
| C3orf52 | turquoise |
| C3orf58 | turquoise |
| C3orf70 | turquoise |
| C4BPB | turquoise |
| C4orf19 | turquoise |
| C4orf29 | turquoise |
| C4orf33 | turquoise |
| C4orf36 | turquoise |
| C5 | turquoise |
| C5orf15 | turquoise |
| C5orf24 | turquoise |
| C5orf28 | turquoise |
| C5orf42 | turquoise |
| C5orf46 | turquoise |
| C5orf51 | turquoise |
| C5orf56 | turquoise |
| C5orf63 | turquoise |
| C6orf1 | turquoise |
| C6orf106 | turquoise |
| C6orf120 | turquoise |
| C6orf141 | turquoise |
| C6orf223 | turquoise |
| C6orf226 | turquoise |
| C6orf47 | turquoise |
| C6orf62 | turquoise |
| C7orf26 | turquoise |
| C7orf31 | turquoise |
| C7orf55-LUC7L2 | turquoise |
| C7orf60 | turquoise |
| C8A | turquoise |
| C8orf37 | turquoise |
| C8orf44 | turquoise |
| C8orf58 | turquoise |
| C8orf88 | turquoise |
| C9orf156 | turquoise |
| C9orf16 | turquoise |
| C9orf3 | turquoise |
| C9orf41 | turquoise |
| C9orf64 | turquoise |
| C9orf69 | turquoise |
| C9orf72 | turquoise |
| C9orf78 | turquoise |
| C9orf84 | turquoise |
| C9orf85 | turquoise |
| C9orf89 | turquoise |
| C9orf91 | turquoise |
| CA11 | turquoise |
| CA13 | turquoise |
| CA5B | turquoise |
| CA5BP1 | turquoise |
| CAAP1 | turquoise |
| CAB39 | turquoise |
| CACFD1 | turquoise |
| CACNA1C | turquoise |
| CACNA1D | turquoise |
| CACNB2 | turquoise |
| CACNG4 | turquoise |
| CACUL1 | turquoise |
| CADPS2 | turquoise |
| CALM2P2 | turquoise |
| CALM3 | turquoise |
| CALR | turquoise |
| CAMK2D | turquoise |
| CAMKMT | turquoise |
| CAMSAP1 | turquoise |
| CAMSAP2 | turquoise |
| CAMTA1 | turquoise |
| CAND1 | turquoise |
| CAPN1 | turquoise |
| CAPN3 | turquoise |
| CAPN7 | turquoise |
| CAPNS1 | turquoise |
| CAPRIN1 | turquoise |
| CAPRIN2 | turquoise |
| CAPS2 | turquoise |
| CAPZB | turquoise |
| CARD10 | turquoise |
| CARD8 | turquoise |
| CARF | turquoise |
| CARHSP1 | turquoise |
| CASC15 | turquoise |
| CASC19 | turquoise |
| CASC4 | turquoise |
| CASD1 | turquoise |
| CASK | turquoise |
| CASP10 | turquoise |
| CASP2 | turquoise |
| CASP8 | turquoise |
| CASP8AP2 | turquoise |
| CASP9 | turquoise |
| CAST | turquoise |
| CATSPER1 | turquoise |
| CATSPER2P1 | turquoise |
| CATSPERB | turquoise |
| CBFB | turquoise |
| CBL | turquoise |
| CBLB | turquoise |
| CBLL1 | turquoise |
| CBR3-AS1 | turquoise |
| CBR4 | turquoise |
| CBWD1 | turquoise |
| CBWD2 | turquoise |
| CBX3P2 | turquoise |
| CBX5 | turquoise |
| CBY1 | turquoise |
| CCAR1 | turquoise |
| CCAR2 | turquoise |
| CCBL2 | turquoise |
| CCDC101 | turquoise |
| CCDC102A | turquoise |
| CCDC102B | turquoise |
| CCDC106 | turquoise |
| CCDC107 | turquoise |
| CCDC112 | turquoise |
| CCDC115 | turquoise |
| CCDC117 | turquoise |
| CCDC12 | turquoise |
| CCDC121 | turquoise |
| CCDC122 | turquoise |
| CCDC124 | turquoise |
| CCDC125 | turquoise |
| CCDC126 | turquoise |
| CCDC129 | turquoise |
| CCDC132 | turquoise |
| CCDC14 | turquoise |
| CCDC147-AS1 | turquoise |
| CCDC15 | turquoise |
| CCDC174 | turquoise |
| CCDC18 | turquoise |
| CCDC186 | turquoise |
| CCDC22 | turquoise |
| CCDC25 | turquoise |
| CCDC39 | turquoise |
| CCDC47 | turquoise |
| CCDC50 | turquoise |
| CCDC51 | turquoise |
| CCDC53 | turquoise |
| CCDC6 | turquoise |
| CCDC66 | turquoise |
| CCDC68 | turquoise |
| CCDC71 | turquoise |
| CCDC71L | turquoise |
| CCDC82 | turquoise |
| CCDC85B | turquoise |
| CCDC85C | turquoise |
| CCDC88A | turquoise |
| CCDC9 | turquoise |
| CCDC91 | turquoise |
| CCDC93 | turquoise |
| CCDC94 | turquoise |
| CCDC97 | turquoise |
| CCL17 | turquoise |
| CCL21 | turquoise |
| CCL22 | turquoise |
| CCL26 | turquoise |
| CCL28 | turquoise |
| CCNC | turquoise |
| CCND2 | turquoise |
| CCND3 | turquoise |
| CCNG2 | turquoise |
| CCNH | turquoise |
| CCNI | turquoise |
| CCNJ | turquoise |
| CCNK | turquoise |
| CCNL1 | turquoise |
| CCNT1 | turquoise |
| CCNT2 | turquoise |
| CCNT2-AS1 | turquoise |
| CCNY | turquoise |
| CCNYL1 | turquoise |
| CCP110 | turquoise |
| CCPG1 | turquoise |
| CCRN4L | turquoise |
| CCSAP | turquoise |
| CCSER1 | turquoise |
| CCSER2 | turquoise |
| CCT6B | turquoise |
| CCT6P1 | turquoise |
| CCT6P3 | turquoise |
| CCZ1 | turquoise |
| CD109 | turquoise |
| CD151 | turquoise |
| CD164 | turquoise |
| CD200R1 | turquoise |
| CD276 | turquoise |
| CD28 | turquoise |
| CD2AP | turquoise |
| CD2BP2 | turquoise |
| CD320 | turquoise |
| CD46 | turquoise |
| CD47 | turquoise |
| CD63 | turquoise |
| CD80 | turquoise |
| CD84 | turquoise |
| CD9 | turquoise |
| CD99 | turquoise |
| CD99L2 | turquoise |
| CDA | turquoise |
| CDADC1 | turquoise |
| CDC14A | turquoise |
| CDC14B | turquoise |
| CDC16 | turquoise |
| CDC23 | turquoise |
| CDC26 | turquoise |
| CDC27 | turquoise |
| CDC34 | turquoise |
| CDC37 | turquoise |
| CDC37L1 | turquoise |
| CDC40 | turquoise |
| CDC42BPA | turquoise |
| CDC42EP1 | turquoise |
| CDC42EP3 | turquoise |
| CDC42EP4 | turquoise |
| CDC42EP5 | turquoise |
| CDC42SE1 | turquoise |
| CDC42SE2 | turquoise |
| CDC73 | turquoise |
| CDCA4P1 | turquoise |
| CDCA7L | turquoise |
| CDH26 | turquoise |
| CDH6 | turquoise |
| CDIP1 | turquoise |
| CDIPT | turquoise |
| CDK12 | turquoise |
| CDK13 | turquoise |
| CDK14 | turquoise |
| CDK17 | turquoise |
| CDK19 | turquoise |
| CDK2AP2 | turquoise |
| CDK4 | turquoise |
| CDK5RAP2 | turquoise |
| CDK6 | turquoise |
| CDKL2 | turquoise |
| CDKL5 | turquoise |
| CDKN1B | turquoise |
| CDKN2AIP | turquoise |
| CDNF | turquoise |
| CDPF1 | turquoise |
| CDR2 | turquoise |
| CDR2L | turquoise |
| CDS2 | turquoise |
| CDV3 | turquoise |
| CDYL | turquoise |
| CDYL2 | turquoise |
| CEBPA | turquoise |
| CEBPB | turquoise |
| CELF1 | turquoise |
| CELSR3-AS1 | turquoise |
| CENPB | turquoise |
| CENPC | turquoise |
| CENPJ | turquoise |
| CEP104 | turquoise |
| CEP112 | turquoise |
| CEP120 | turquoise |
| CEP128 | turquoise |
| CEP135 | turquoise |
| CEP152 | turquoise |
| CEP162 | turquoise |
| CEP170 | turquoise |
| CEP192 | turquoise |
| CEP290 | turquoise |
| CEP295 | turquoise |
| CEP350 | turquoise |
| CEP41 | turquoise |
| CEP44 | turquoise |
| CEP57 | turquoise |
| CEP57L1 | turquoise |
| CEP63 | turquoise |
| CEP68 | turquoise |
| CEP70 | turquoise |
| CEP72 | turquoise |
| CEP78 | turquoise |
| CEP83 | turquoise |
| CEP85L | turquoise |
| CEP95 | turquoise |
| CEP97 | turquoise |
| CEPT1 | turquoise |
| CERK | turquoise |
| CERKL | turquoise |
| CERS5 | turquoise |
| CERS6 | turquoise |
| CES1 | turquoise |
| CFAP20 | turquoise |
| CFAP44 | turquoise |
| CFAP69 | turquoise |
| CFAP97 | turquoise |
| CFH | turquoise |
| CFL1 | turquoise |
| CFLAR | turquoise |
| CFLAR-AS1 | turquoise |
| CFTR | turquoise |
| CGGBP1 | turquoise |
| CH17-13I23.3 | turquoise |
| CH17-140K24.6 | turquoise |
| CH17-189H20.1 | turquoise |
| CH17-262A2.1 | turquoise |
| CH17-340M24.3 | turquoise |
| CH17-373J23.1 | turquoise |
| CH17-472G23.2 | turquoise |
| CH17-472G23.4 | turquoise |
| CH507-513H4.5 | turquoise |
| CHADL | turquoise |
| CHAMP1 | turquoise |
| CHCHD4 | turquoise |
| CHCHD6 | turquoise |
| CHD1 | turquoise |
| CHD2 | turquoise |
| CHD6 | turquoise |
| CHD7 | turquoise |
| CHD8 | turquoise |
| CHD9 | turquoise |
| CHFR | turquoise |
| CHI3L1 | turquoise |
| CHIC1 | turquoise |
| CHIC2 | turquoise |
| CHID1 | turquoise |
| CHL1 | turquoise |
| CHM | turquoise |
| CHML | turquoise |
| CHMP1A | turquoise |
| CHMP2A | turquoise |
| CHMP2B | turquoise |
| CHMP4B | turquoise |
| CHMP6 | turquoise |
| CHMP7 | turquoise |
| CHN2 | turquoise |
| CHORDC1 | turquoise |
| CHPF | turquoise |
| CHST10 | turquoise |
| CHST14 | turquoise |
| CHST3 | turquoise |
| CHSY1 | turquoise |
| CHSY3 | turquoise |
| CHTF8 | turquoise |
| CHUK | turquoise |
| CHURC1 | turquoise |
| CIB1 | turquoise |
| CIPC | turquoise |
| CIR1 | turquoise |
| CIRBP-AS1 | turquoise |
| CISD3 | turquoise |
| CITED4 | turquoise |
| CITF22-92A6.1 | turquoise |
| CIZ1 | turquoise |
| CKB | turquoise |
| CKMT2 | turquoise |
| CKMT2-AS1 | turquoise |
| CLASP1 | turquoise |
| CLASP2 | turquoise |
| CLCC1 | turquoise |
| CLCN3 | turquoise |
| CLCN5 | turquoise |
| CLDN10-AS1 | turquoise |
| CLDN12 | turquoise |
| CLEC16A | turquoise |
| CLEC2D | turquoise |
| CLEC5A | turquoise |
| CLHC1 | turquoise |
| CLIC1 | turquoise |
| CLINT1 | turquoise |
| CLIP1 | turquoise |
| CLIP4 | turquoise |
| CLK1 | turquoise |
| CLK4 | turquoise |
| CLMN | turquoise |
| CLN3 | turquoise |
| CLN6 | turquoise |
| CLN8 | turquoise |
| CLOCK | turquoise |
| CLP1 | turquoise |
| CLPP | turquoise |
| CLPTM1 | turquoise |
| CLPX | turquoise |
| CLSTN1 | turquoise |
| CLTA | turquoise |
| CLTB | turquoise |
| CLTC | turquoise |
| CLTCL1 | turquoise |
| CLU | turquoise |
| CLUH | turquoise |
| CMAHP | turquoise |
| CMPK1 | turquoise |
| CMPK2 | turquoise |
| CMTM1 | turquoise |
| CMTM4 | turquoise |
| CMTM6 | turquoise |
| CMTM8 | turquoise |
| CMTR2 | turquoise |
| CNDP2 | turquoise |
| CNEP1R1 | turquoise |
| CNGA1 | turquoise |
| CNKSR3 | turquoise |
| CNN2 | turquoise |
| CNNM2 | turquoise |
| CNNM4 | turquoise |
| CNOT1 | turquoise |
| CNOT10 | turquoise |
| CNOT2 | turquoise |
| CNOT4 | turquoise |
| CNOT6 | turquoise |
| CNOT6L | turquoise |
| CNOT7 | turquoise |
| CNOT8 | turquoise |
| CNPPD1 | turquoise |
| CNPY3 | turquoise |
| CNST | turquoise |
| CNTD1 | turquoise |
| CNTLN | turquoise |
| CNTN1 | turquoise |
| CNTN3 | turquoise |
| CNTRL | turquoise |
| COA1 | turquoise |
| COBLL1 | turquoise |
| COG3 | turquoise |
| COG5 | turquoise |
| COG6 | turquoise |
| COL14A1 | turquoise |
| COL21A1 | turquoise |
| COL28A1 | turquoise |
| COL4A3 | turquoise |
| COL4A3BP | turquoise |
| COL4A5 | turquoise |
| COL6A5 | turquoise |
| COLCA1 | turquoise |
| COLGALT1 | turquoise |
| COMMD9 | turquoise |
| COMT | turquoise |
| COMTD1 | turquoise |
| COPRS | turquoise |
| COPS2 | turquoise |
| COPS6 | turquoise |
| COPS7A | turquoise |
| COQ9 | turquoise |
| CORO1B | turquoise |
| COX10-AS1 | turquoise |
| COX11 | turquoise |
| COX15 | turquoise |
| COX18 | turquoise |
| COX20 | turquoise |
| COX20P1 | turquoise |
| COX5BP6 | turquoise |
| COX6CP1 | turquoise |
| COX7CP1 | turquoise |
| CPD | turquoise |
| CPEB2 | turquoise |
| CPEB3 | turquoise |
| CPEB4 | turquoise |
| CPNE2 | turquoise |
| CPNE3 | turquoise |
| CPNE4 | turquoise |
| CPNE8 | turquoise |
| CPSF2 | turquoise |
| CPSF6 | turquoise |
| CPT2 | turquoise |
| CPTP | turquoise |
| CPXM2 | turquoise |
| CRACR2A | turquoise |
| CRAT | turquoise |
| CRB3 | turquoise |
| CRBN | turquoise |
| CREB1 | turquoise |
| CREB3 | turquoise |
| CREB3L1 | turquoise |
| CREB3L2 | turquoise |
| CREB5 | turquoise |
| CREBBP | turquoise |
| CREBRF | turquoise |
| CREBZF | turquoise |
| CRELD1 | turquoise |
| CRELD2 | turquoise |
| CREM | turquoise |
| CRHR1-IT1 | turquoise |
| CRIP1 | turquoise |
| CRIP2 | turquoise |
| CRIPAK | turquoise |
| CRK | turquoise |
| CRKL | turquoise |
| CRLF3 | turquoise |
| CRLS1 | turquoise |
| CRNDE | turquoise |
| CRNKL1 | turquoise |
| CROCCP3 | turquoise |
| CROT | turquoise |
| CRTC3 | turquoise |
| CRY1 | turquoise |
| CRYBB2P1 | turquoise |
| CRYBG3 | turquoise |
| CRYL1 | turquoise |
| CRYZL1 | turquoise |
| CSDE1 | turquoise |
| CSGALNACT1 | turquoise |
| CSGALNACT2 | turquoise |
| CSNK1A1 | turquoise |
| CSNK1G1 | turquoise |
| CSNK1G3 | turquoise |
| CSNK2A2 | turquoise |
| CSPG5 | turquoise |
| CSPP1 | turquoise |
| CSRNP2 | turquoise |
| CSRP2BP | turquoise |
| CST2 | turquoise |
| CST3 | turquoise |
| CST4 | turquoise |
| CST6 | turquoise |
| CSTB | turquoise |
| CSTF2T | turquoise |
| CTA-217C2.2 | turquoise |
| CTA-221G9.12 | turquoise |
| CTA-253N17.1 | turquoise |
| CTA-276F8.1 | turquoise |
| CTA-276O3.4 | turquoise |
| CTA-292E10.6 | turquoise |
| CTA-292E10.9 | turquoise |
| CTA-293F17.1 | turquoise |
| CTA-363E19.2 | turquoise |
| CTA-363E6.2 | turquoise |
| CTA-363E6.6 | turquoise |
| CTA-38K21.5 | turquoise |
| CTA-989H11.1 | turquoise |
| CTAGE5 | turquoise |
| CTB-102L5.4 | turquoise |
| CTB-113P19.5 | turquoise |
| CTB-119C2.1 | turquoise |
| CTB-131K11.1 | turquoise |
| CTB-13F3.1 | turquoise |
| CTB-152G17.6 | turquoise |
| CTB-175E5.7 | turquoise |
| CTB-179K24.3 | turquoise |
| CTB-25B13.12 | turquoise |
| CTB-31N19.3 | turquoise |
| CTB-36H16.2 | turquoise |
| CTB-36O1.7 | turquoise |
| CTB-50E14.5 | turquoise |
| CTB-55O6.12 | turquoise |
| CTB-58E17.3 | turquoise |
| CTB-61M7.2 | turquoise |
| CTB-89H12.4 | turquoise |
| CTB-92J24.2 | turquoise |
| CTBS | turquoise |
| CTC-205M6.1 | turquoise |
| CTC-205M6.5 | turquoise |
| CTC-215O4.4 | turquoise |
| CTC-231O11.1 | turquoise |
| CTC-260E6.4 | turquoise |
| CTC-277H1.7 | turquoise |
| CTC-301O7.4 | turquoise |
| CTC-325H20.4 | turquoise |
| CTC-339F2.2 | turquoise |
| CTC-343N3.1 | turquoise |
| CTC-359D24.3 | turquoise |
| CTC-366B18.4 | turquoise |
| CTC-398G3.2 | turquoise |
| CTC-425O23.5 | turquoise |
| CTC-428H11.2 | turquoise |
| CTC-429L19.3 | turquoise |
| CTC-429P9.1 | turquoise |
| CTC-429P9.2 | turquoise |
| CTC-429P9.5 | turquoise |
| CTC-431G16.2 | turquoise |
| CTC-444N24.11 | turquoise |
| CTC-444N24.6 | turquoise |
| CTC-444N24.7 | turquoise |
| CTC-444N24.8 | turquoise |
| CTC-448F2.6 | turquoise |
| CTC-459F4.3 | turquoise |
| CTC-459F4.9 | turquoise |
| CTC-471F3.5 | turquoise |
| CTC-471J1.11 | turquoise |
| CTC-471J1.2 | turquoise |
| CTC-487M23.5 | turquoise |
| CTC-487M23.7 | turquoise |
| CTC-490E21.10 | turquoise |
| CTC-490E21.11 | turquoise |
| CTC-497E21.3 | turquoise |
| CTC-510F12.7 | turquoise |
| CTC-523E23.11 | turquoise |
| CTC-523E23.3 | turquoise |
| CTC-526N19.1 | turquoise |
| CTC-529I10.1 | turquoise |
| CTC-529I10.2 | turquoise |
| CTC-534A2.2 | turquoise |
| CTC-542B22.2 | turquoise |
| CTC-550B14.7 | turquoise |
| CTC-559E9.1 | turquoise |
| CTC-559E9.5 | turquoise |
| CTC-559E9.6 | turquoise |
| CTC-559E9.8 | turquoise |
| CTC-573N18.1 | turquoise |
| CTCF | turquoise |
| CTD-2002H8.2 | turquoise |
| CTD-2006C1.12 | turquoise |
| CTD-2006H14.2 | turquoise |
| CTD-2010I16.1 | turquoise |
| CTD-2012J19.2 | turquoise |
| CTD-2012J19.3 | turquoise |
| CTD-2015H6.3 | turquoise |
| CTD-2017C7.2 | turquoise |
| CTD-2017D11.1 | turquoise |
| CTD-2017D11.2 | turquoise |
| CTD-2020K17.4 | turquoise |
| CTD-2023M8.1 | turquoise |
| CTD-2024I7.1 | turquoise |
| CTD-2026D20.3 | turquoise |
| CTD-2027I19.3 | turquoise |
| CTD-2033D15.2 | turquoise |
| CTD-2033D15.3 | turquoise |
| CTD-2035E11.3 | turquoise |
| CTD-2035E11.4 | turquoise |
| CTD-2035E11.5 | turquoise |
| CTD-2047H16.2 | turquoise |
| CTD-2047H16.4 | turquoise |
| CTD-2104P17.2 | turquoise |
| CTD-2152M20.2 | turquoise |
| CTD-2196E14.6 | turquoise |
| CTD-2196E14.8 | turquoise |
| CTD-2201E18.3 | turquoise |
| CTD-2201E18.5 | turquoise |
| CTD-2235C13.1 | turquoise |
| CTD-2245F17.3 | turquoise |
| CTD-2248H3.1 | turquoise |
| CTD-2260A17.1 | turquoise |
| CTD-2267D19.3 | turquoise |
| CTD-2270P14.1 | turquoise |
| CTD-2280E9.1 | turquoise |
| CTD-2286N8.2 | turquoise |
| CTD-2291D10.4 | turquoise |
| CTD-2292M14.1 | turquoise |
| CTD-2313J17.5 | turquoise |
| CTD-2331H12.5 | turquoise |
| CTD-2336O2.1 | turquoise |
| CTD-2349P21.10 | turquoise |
| CTD-2349P21.9 | turquoise |
| CTD-2366F13.2 | turquoise |
| CTD-2368P22.1 | turquoise |
| CTD-2371O3.3 | turquoise |
| CTD-2373N4.3 | turquoise |
| CTD-2376I4.1 | turquoise |
| CTD-2376I4.2 | turquoise |
| CTD-2396E7.11 | turquoise |
| CTD-2521M24.9 | turquoise |
| CTD-2528L19.6 | turquoise |
| CTD-2530N21.4 | turquoise |
| CTD-2537I9.18 | turquoise |
| CTD-2547G23.4 | turquoise |
| CTD-2547H18.1 | turquoise |
| CTD-2547L24.3 | turquoise |
| CTD-2547L24.4 | turquoise |
| CTD-2553L13.9 | turquoise |
| CTD-2554C21.2 | turquoise |
| CTD-2554C21.3 | turquoise |
| CTD-2555O16.2 | turquoise |
| CTD-2555O16.4 | turquoise |
| CTD-2561J22.2 | turquoise |
| CTD-2561J22.5 | turquoise |
| CTD-2566J3.1 | turquoise |
| CTD-2574D22.3 | turquoise |
| CTD-2574D22.4 | turquoise |
| CTD-2583A14.8 | turquoise |
| CTD-2589M5.4 | turquoise |
| CTD-2600H12.2 | turquoise |
| CTD-2619J13.16 | turquoise |
| CTD-2619J13.17 | turquoise |
| CTD-2621I17.3 | turquoise |
| CTD-2623N2.3 | turquoise |
| CTD-2626G11.2 | turquoise |
| CTD-2647L4.4 | turquoise |
| CTD-2649C14.2 | turquoise |
| CTD-3014M21.1 | turquoise |
| CTD-3018O17.5 | turquoise |
| CTD-3025N20.3 | turquoise |
| CTD-3032J10.4 | turquoise |
| CTD-3092A11.2 | turquoise |
| CTD-3099C6.9 | turquoise |
| CTD-3131K8.2 | turquoise |
| CTD-3131K8.3 | turquoise |
| CTD-3138B18.5 | turquoise |
| CTD-3138B18.6 | turquoise |
| CTD-3157E16.1 | turquoise |
| CTD-3157E16.2 | turquoise |
| CTD-3185P2.1 | turquoise |
| CTD-3199J23.6 | turquoise |
| CTD-3214K23.1 | turquoise |
| CTD-3220F14.2 | turquoise |
| CTDNEP1 | turquoise |
| CTDSP1 | turquoise |
| CTDSPL2 | turquoise |
| CTF1 | turquoise |
| CTNNB1 | turquoise |
| CTNNBIP1 | turquoise |
| CTNND1 | turquoise |
| CTNND2 | turquoise |
| CTPS2 | turquoise |
| CTR9 | turquoise |
| CTSA | turquoise |
| CTSD | turquoise |
| CTSF | turquoise |
| CTSL | turquoise |
| CTTNBP2NL | turquoise |
| CUEDC2 | turquoise |
| CUL3 | turquoise |
| CUL4B | turquoise |
| CUL5 | turquoise |
| CUTA | turquoise |
| CUX1 | turquoise |
| CWC22 | turquoise |
| CWF19L2 | turquoise |
| CXADR | turquoise |
| CXCL17 | turquoise |
| CXorf23 | turquoise |
| CXorf38 | turquoise |
| CXorf40A | turquoise |
| CXXC4 | turquoise |
| CXXC5 | turquoise |
| CYB561 | turquoise |
| CYB561D2 | turquoise |
| CYB5D2 | turquoise |
| CYB5R1 | turquoise |
| CYB5R3 | turquoise |
| CYB5R4 | turquoise |
| CYB5RL | turquoise |
| CYBA | turquoise |
| CYCSP10 | turquoise |
| CYCSP34 | turquoise |
| CYLD | turquoise |
| CYP17A1 | turquoise |
| CYP1B1 | turquoise |
| CYP20A1 | turquoise |
| CYP2A6 | turquoise |
| CYP2R1 | turquoise |
| CYP4F29P | turquoise |
| CYP4V2 | turquoise |
| CYP4Z2P | turquoise |
| CYP51A1 | turquoise |
| CYP7B1 | turquoise |
| CYSLTR1 | turquoise |
| CYTH3 | turquoise |
| DAAM1 | turquoise |
| DAG1 | turquoise |
| DAK | turquoise |
| DALRD3 | turquoise |
| DAPK3 | turquoise |
| DAPP1 | turquoise |
| DAXX | turquoise |
| DAZAP2 | turquoise |
| DBI | turquoise |
| DBNDD2 | turquoise |
| DBT | turquoise |
| DCAF10 | turquoise |
| DCAF11 | turquoise |
| DCAF16 | turquoise |
| DCAF17 | turquoise |
| DCAF5 | turquoise |
| DCAF6 | turquoise |
| DCAF7 | turquoise |
| DCBLD2 | turquoise |
| DCLK1 | turquoise |
| DCLK2 | turquoise |
| DCLRE1C | turquoise |
| DCP1A | turquoise |
| DCP1B | turquoise |
| DCP2 | turquoise |
| DCTD | turquoise |
| DCTN1 | turquoise |
| DCTN2 | turquoise |
| DCTN4 | turquoise |
| DCTN5 | turquoise |
| DCUN1D1 | turquoise |
| DCUN1D3 | turquoise |
| DCUN1D4 | turquoise |
| DCXR | turquoise |
| DDA1 | turquoise |
| DDAH1 | turquoise |
| DDAH2 | turquoise |
| DDHD1 | turquoise |
| DDHD2 | turquoise |
| DDI2 | turquoise |
| DDIT4 | turquoise |
| DDOST | turquoise |
| DDRGK1 | turquoise |
| DDT | turquoise |
| DDX17 | turquoise |
| DDX19A | turquoise |
| DDX20 | turquoise |
| DDX26B | turquoise |
| DDX28 | turquoise |
| DDX31 | turquoise |
| DDX3X | turquoise |
| DDX3Y | turquoise |
| DDX41 | turquoise |
| DDX42 | turquoise |
| DDX43 | turquoise |
| DDX46 | turquoise |
| DDX47 | turquoise |
| DDX49 | turquoise |
| DDX5 | turquoise |
| DDX52 | turquoise |
| DDX58 | turquoise |
| DDX59 | turquoise |
| DDX6 | turquoise |
| DDX60 | turquoise |
| DDX60L | turquoise |
| DEDD2 | turquoise |
| DEFB4A | turquoise |
| DENND1B | turquoise |
| DENND2C | turquoise |
| DENND4A | turquoise |
| DENND4C | turquoise |
| DENND5A | turquoise |
| DENND5B | turquoise |
| DENND6A | turquoise |
| DEPDC5 | turquoise |
| DEPDC7 | turquoise |
| DESI2 | turquoise |
| DET1 | turquoise |
| DEXI | turquoise |
| DFNA5 | turquoise |
| DFNB59 | turquoise |
| DGAT1 | turquoise |
| DGCR11 | turquoise |
| DGCR14 | turquoise |
| DGCR2 | turquoise |
| DGCR6 | turquoise |
| DGCR6L | turquoise |
| DGCR9 | turquoise |
| DGKE | turquoise |
| DGKG | turquoise |
| DGKH | turquoise |
| DGUOK-AS1 | turquoise |
| DHCR7 | turquoise |
| DHFRL1 | turquoise |
| DHRS3 | turquoise |
| DHRS4 | turquoise |
| DHRS4L2 | turquoise |
| DHRS7 | turquoise |
| DHRS7B | turquoise |
| DHX15 | turquoise |
| DHX29 | turquoise |
| DHX35 | turquoise |
| DHX36 | turquoise |
| DHX40 | turquoise |
| DIAPH2 | turquoise |
| DICER1 | turquoise |
| DIDO1 | turquoise |
| DIEXF | turquoise |
| DIMT1 | turquoise |
| DIP2A | turquoise |
| DIP2B | turquoise |
| DIP2C | turquoise |
| DIRC2 | turquoise |
| DIS3 | turquoise |
| DIS3L | turquoise |
| DIS3L2 | turquoise |
| DISC1 | turquoise |
| DKFZP434I0714 | turquoise |
| DLEU1 | turquoise |
| DLEU2 | turquoise |
| DLG1 | turquoise |
| DLGAP1-AS2 | turquoise |
| DLK1 | turquoise |
| DLST | turquoise |
| DMD | turquoise |
| DMGDH | turquoise |
| DMKN | turquoise |
| DMRTA1 | turquoise |
| DMTF1 | turquoise |
| DMTN | turquoise |
| DMXL1 | turquoise |
| DMXL2 | turquoise |
| DNAH11 | turquoise |
| DNAH14 | turquoise |
| DNAH17 | turquoise |
| DNAH5 | turquoise |
| DNAJB1 | turquoise |
| DNAJB12 | turquoise |
| DNAJB14 | turquoise |
| DNAJB2 | turquoise |
| DNAJB6 | turquoise |
| DNAJC10 | turquoise |
| DNAJC13 | turquoise |
| DNAJC16 | turquoise |
| DNAJC19P5 | turquoise |
| DNAJC2 | turquoise |
| DNAJC21 | turquoise |
| DNAJC24 | turquoise |
| DNAJC25 | turquoise |
| DNAJC27 | turquoise |
| DNAJC28 | turquoise |
| DNAJC3 | turquoise |
| DNAJC3-AS1 | turquoise |
| DNAJC4 | turquoise |
| DNAJC6 | turquoise |
| DNAL4 | turquoise |
| DNASE1L1 | turquoise |
| DNASE2 | turquoise |
| DNHD1 | turquoise |
| DNM1L | turquoise |
| DNM3 | turquoise |
| DNM3OS | turquoise |
| DNMBP | turquoise |
| DNMBP-AS1 | turquoise |
| DNPEP | turquoise |
| DOC2B | turquoise |
| DOCK1 | turquoise |
| DOCK10 | turquoise |
| DOCK11 | turquoise |
| DOCK3 | turquoise |
| DOCK4 | turquoise |
| DOCK5 | turquoise |
| DOCK7 | turquoise |
| DOCK9 | turquoise |
| DOHH | turquoise |
| DOK1 | turquoise |
| DOK5 | turquoise |
| DOK6 | turquoise |
| DOLK | turquoise |
| DOPEY1 | turquoise |
| DOPEY2 | turquoise |
| DPAGT1 | turquoise |
| DPH6 | turquoise |
| DPM2 | turquoise |
| DPP4 | turquoise |
| DPP7 | turquoise |
| DPP8 | turquoise |
| DPY19L1 | turquoise |
| DPY19L1P1 | turquoise |
| DPY19L1P2 | turquoise |
| DPY19L3 | turquoise |
| DPY19L4 | turquoise |
| DPYD | turquoise |
| DPYD-AS1 | turquoise |
| DR1 | turquoise |
| DRGX | turquoise |
| DSC2 | turquoise |
| DSCR3 | turquoise |
| DSE | turquoise |
| DSEL | turquoise |
| DST | turquoise |
| DSTN | turquoise |
| DSTNP1 | turquoise |
| DSTYK | turquoise |
| DTD2 | turquoise |
| DTNA | turquoise |
| DTNB | turquoise |
| DTWD1 | turquoise |
| DTWD2 | turquoise |
| DTX2P1 | turquoise |
| DTX3L | turquoise |
| DUS3L | turquoise |
| DUS4L | turquoise |
| DUSP10 | turquoise |
| DUSP16 | turquoise |
| DUSP28 | turquoise |
| DUSP6 | turquoise |
| DUSP7 | turquoise |
| DUTP6 | turquoise |
| DYM | turquoise |
| DYNC1H1 | turquoise |
| DYNC1LI2 | turquoise |
| DYNC2H1 | turquoise |
| DYNLRB1 | turquoise |
| DYNLT3 | turquoise |
| DYRK1A | turquoise |
| DYRK1B | turquoise |
| DYRK2 | turquoise |
| DYRK3 | turquoise |
| DYRK4 | turquoise |
| E2F4 | turquoise |
| E2F5 | turquoise |
| EAF1 | turquoise |
| EBF1 | turquoise |
| EBLN2 | turquoise |
| EBLN3 | turquoise |
| EBP | turquoise |
| ECD | turquoise |
| ECE1 | turquoise |
| ECH1 | turquoise |
| ECHDC1 | turquoise |
| ECHDC3 | turquoise |
| ECHS1 | turquoise |
| ECI2 | turquoise |
| ECM1 | turquoise |
| ECSIT | turquoise |
| EDA2R | turquoise |
| EDEM1 | turquoise |
| EDEM2 | turquoise |
| EDEM3 | turquoise |
| EDF1 | turquoise |
| EDN2 | turquoise |
| EDNRA | turquoise |
| EDRF1 | turquoise |
| EEA1 | turquoise |
| EED | turquoise |
| EEF1A1P4 | turquoise |
| EEF1D | turquoise |
| EEF2 | turquoise |
| EEF2K | turquoise |
| EEFSEC | turquoise |
| EFCAB11 | turquoise |
| EFCAB13 | turquoise |
| EFCAB14 | turquoise |
| EFCAB7 | turquoise |
| EFHD2 | turquoise |
| EFNA5 | turquoise |
| EFR3A | turquoise |
| EFR3B | turquoise |
| EFTUD1 | turquoise |
| EGLN1 | turquoise |
| EHBP1 | turquoise |
| EHF | turquoise |
| EHMT1 | turquoise |
| EID2 | turquoise |
| EID3 | turquoise |
| EIF1 | turquoise |
| EIF1AX | turquoise |
| EIF1AY | turquoise |
| EIF1B | turquoise |
| EIF2A | turquoise |
| EIF2AK2 | turquoise |
| EIF2AK3 | turquoise |
| EIF2AK4 | turquoise |
| EIF3A | turquoise |
| EIF3C | turquoise |
| EIF3D | turquoise |
| EIF3EP1 | turquoise |
| EIF3F | turquoise |
| EIF3FP3 | turquoise |
| EIF3G | turquoise |
| EIF3J | turquoise |
| EIF4A1 | turquoise |
| EIF4A2 | turquoise |
| EIF4BP3 | turquoise |
| EIF4BP7 | turquoise |
| EIF4E | turquoise |
| EIF4E3 | turquoise |
| EIF4EBP2 | turquoise |
| EIF4EBP3 | turquoise |
| EIF4ENIF1 | turquoise |
| EIF4EP1 | turquoise |
| EIF4G2 | turquoise |
| EIF4G3 | turquoise |
| EIF4H | turquoise |
| EIF5 | turquoise |
| EIF5A2 | turquoise |
| EIF6 | turquoise |
| ELAC1 | turquoise |
| ELAC2 | turquoise |
| ELF1 | turquoise |
| ELF2 | turquoise |
| ELF5 | turquoise |
| ELK3 | turquoise |
| ELK4 | turquoise |
| ELL2 | turquoise |
| ELMO3 | turquoise |
| ELMOD2 | turquoise |
| ELMSAN1 | turquoise |
| ELOF1 | turquoise |
| ELOVL1 | turquoise |
| ELOVL5 | turquoise |
| ELOVL6 | turquoise |
| ELOVL7 | turquoise |
| ELP2 | turquoise |
| EMB | turquoise |
| EMBP1 | turquoise |
| EMC10 | turquoise |
| EMC2 | turquoise |
| EMC3-AS1 | turquoise |
| EMC6 | turquoise |
| EMD | turquoise |
| EML2 | turquoise |
| EML4 | turquoise |
| EMR2 | turquoise |
| ENAH | turquoise |
| ENC1 | turquoise |
| ENDOD1 | turquoise |
| ENDOG | turquoise |
| ENO1-IT1 | turquoise |
| ENOSF1 | turquoise |
| ENPP4 | turquoise |
| ENPP5 | turquoise |
| ENTPD1 | turquoise |
| ENTPD4 | turquoise |
| ENTPD5 | turquoise |
| ENTPD7 | turquoise |
| EOGT | turquoise |
| EP300 | turquoise |
| EP300-AS1 | turquoise |
| EP400 | turquoise |
| EP400NL | turquoise |
| EPB41 | turquoise |
| EPB41L3 | turquoise |
| EPB41L4A | turquoise |
| EPB41L4B | turquoise |
| EPB41L5 | turquoise |
| EPC1 | turquoise |
| EPC2 | turquoise |
| EPG5 | turquoise |
| EPHA2 | turquoise |
| EPHA3 | turquoise |
| EPHA4 | turquoise |
| EPHB4 | turquoise |
| EPHX1 | turquoise |
| EPM2AIP1 | turquoise |
| EPN1 | turquoise |
| EPPK1 | turquoise |
| EPS15 | turquoise |
| EPS8 | turquoise |
| ERAP1 | turquoise |
| ERAP2 | turquoise |
| ERBB2IP | turquoise |
| ERC1 | turquoise |
| ERCC1 | turquoise |
| ERCC4 | turquoise |
| ERCC6 | turquoise |
| ERCC6L2 | turquoise |
| ERCC8 | turquoise |
| EREG | turquoise |
| ERF | turquoise |
| ERGIC1 | turquoise |
| ERGIC2 | turquoise |
| ERGIC3 | turquoise |
| ERI1 | turquoise |
| ERI2 | turquoise |
| ERICH1 | turquoise |
| ERMARD | turquoise |
| ERMP1 | turquoise |
| ERN1 | turquoise |
| ERP29 | turquoise |
| ERRFI1 | turquoise |
| ERV3-1 | turquoise |
| ERVK3-1 | turquoise |
| ESCO1 | turquoise |
| ESF1 | turquoise |
| ESR1 | turquoise |
| ESRRA | turquoise |
| ESYT2 | turquoise |
| ESYT3 | turquoise |
| ETAA1 | turquoise |
| ETFB | turquoise |
| ETFDH | turquoise |
| ETHE1 | turquoise |
| ETNK1 | turquoise |
| ETV1 | turquoise |
| ETV3 | turquoise |
| ETV6 | turquoise |
| EVA1B | turquoise |
| EVC2 | turquoise |
| EVI5 | turquoise |
| EVI5L | turquoise |
| EXD2 | turquoise |
| EXOC1 | turquoise |
| EXOC2 | turquoise |
| EXOC4 | turquoise |
| EXOC5 | turquoise |
| EXOC6 | turquoise |
| EXOC6B | turquoise |
| EXOC8 | turquoise |
| EXOG | turquoise |
| EXOSC3 | turquoise |
| EXOSC3P1 | turquoise |
| EXOSC6 | turquoise |
| EXPH5 | turquoise |
| EXT1 | turquoise |
| EXTL2 | turquoise |
| EYA3 | turquoise |
| EZR | turquoise |
| F3 | turquoise |
| F8A1 | turquoise |
| FABP3 | turquoise |
| FADD | turquoise |
| FADS3 | turquoise |
| FAF2 | turquoise |
| FAH | turquoise |
| FAHD2B | turquoise |
| FAIM | turquoise |
| FAM102A | turquoise |
| FAM102B | turquoise |
| FAM104A | turquoise |
| FAM107B | turquoise |
| FAM109A | turquoise |
| FAM109B | turquoise |
| FAM110B | turquoise |
| FAM110C | turquoise |
| FAM111A | turquoise |
| FAM114A2 | turquoise |
| FAM117B | turquoise |
| FAM118A | turquoise |
| FAM120A | turquoise |
| FAM120AOS | turquoise |
| FAM120B | turquoise |
| FAM120C | turquoise |
| FAM122A | turquoise |
| FAM122B | turquoise |
| FAM122C | turquoise |
| FAM126A | turquoise |
| FAM126B | turquoise |
| FAM127A | turquoise |
| FAM127B | turquoise |
| FAM127C | turquoise |
| FAM129A | turquoise |
| FAM129B | turquoise |
| FAM132B | turquoise |
| FAM133B | turquoise |
| FAM134A | turquoise |
| FAM135A | turquoise |
| FAM13A | turquoise |
| FAM13A-AS1 | turquoise |
| FAM13B | turquoise |
| FAM149A | turquoise |
| FAM149B1 | turquoise |
| FAM160A1 | turquoise |
| FAM160B1 | turquoise |
| FAM161A | turquoise |
| FAM168A | turquoise |
| FAM168B | turquoise |
| FAM171B | turquoise |
| FAM172A | turquoise |
| FAM173B | turquoise |
| FAM175A | turquoise |
| FAM175B | turquoise |
| FAM178A | turquoise |
| FAM179B | turquoise |
| FAM184A | turquoise |
| FAM185A | turquoise |
| FAM185BP | turquoise |
| FAM188A | turquoise |
| FAM193A | turquoise |
| FAM195B | turquoise |
| FAM196A | turquoise |
| FAM198A | turquoise |
| FAM198B | turquoise |
| FAM199X | turquoise |
| FAM200B | turquoise |
| FAM206A | turquoise |
| FAM208A | turquoise |
| FAM208B | turquoise |
| FAM20B | turquoise |
| FAM213B | turquoise |
| FAM214A | turquoise |
| FAM214B | turquoise |
| FAM216A | turquoise |
| FAM217B | turquoise |
| FAM21A | turquoise |
| FAM21C | turquoise |
| FAM221A | turquoise |
| FAM227A | turquoise |
| FAM227B | turquoise |
| FAM228B | turquoise |
| FAM32A | turquoise |
| FAM35A | turquoise |
| FAM3A | turquoise |
| FAM3C | turquoise |
| FAM46A | turquoise |
| FAM47E-STBD1 | turquoise |
| FAM50A | turquoise |
| FAM50B | turquoise |
| FAM53C | turquoise |
| FAM63B | turquoise |
| FAM65B | turquoise |
| FAM65C | turquoise |
| FAM69A | turquoise |
| FAM71E1 | turquoise |
| FAM73A | turquoise |
| FAM76A | turquoise |
| FAM76B | turquoise |
| FAM81A | turquoise |
| FAM83A-AS1 | turquoise |
| FAM83B | turquoise |
| FAM84B | turquoise |
| FAM86DP | turquoise |
| FAM86HP | turquoise |
| FAM89A | turquoise |
| FAM89B | turquoise |
| FAM8A1 | turquoise |
| FAM91A1 | turquoise |
| FAM92A1 | turquoise |
| FAM96AP2 | turquoise |
| FAM98B | turquoise |
| FAM98C | turquoise |
| FAN1 | turquoise |
| FANCC | turquoise |
| FANCL | turquoise |
| FANCM | turquoise |
| FAR1 | turquoise |
| FAR2 | turquoise |
| FARP1 | turquoise |
| FARP2 | turquoise |
| FARSA | turquoise |
| FAS | turquoise |
| FASN | turquoise |
| FASTKD1 | turquoise |
| FASTKD3 | turquoise |
| FASTKD5 | turquoise |
| FAT1 | turquoise |
| FAT4 | turquoise |
| FAXC | turquoise |
| FBLIM1 | turquoise |
| FBXL13 | turquoise |
| FBXL14 | turquoise |
| FBXL15 | turquoise |
| FBXL17 | turquoise |
| FBXL2 | turquoise |
| FBXL20 | turquoise |
| FBXL3 | turquoise |
| FBXL4 | turquoise |
| FBXL5 | turquoise |
| FBXO11 | turquoise |
| FBXO18 | turquoise |
| FBXO2 | turquoise |
| FBXO21 | turquoise |
| FBXO22 | turquoise |
| FBXO25 | turquoise |
| FBXO28 | turquoise |
| FBXO3 | turquoise |
| FBXO30 | turquoise |
| FBXO32 | turquoise |
| FBXO33 | turquoise |
| FBXO34 | turquoise |
| FBXO38 | turquoise |
| FBXO42 | turquoise |
| FBXO48 | turquoise |
| FBXO6 | turquoise |
| FBXO9 | turquoise |
| FBXW11 | turquoise |
| FBXW2 | turquoise |
| FBXW5 | turquoise |
| FBXW7 | turquoise |
| FBXW8 | turquoise |
| FCF1 | turquoise |
| FCF1P2 | turquoise |
| FCGR2C | turquoise |
| FCHO2 | turquoise |
| FCHSD2 | turquoise |
| FDFT1 | turquoise |
| FDPS | turquoise |
| FDXACB1 | turquoise |
| FDXR | turquoise |
| FEM1B | turquoise |
| FEM1C | turquoise |
| FER | turquoise |
| FES | turquoise |
| FEZ2 | turquoise |
| FGD4 | turquoise |
| FGD5-AS1 | turquoise |
| FGD6 | turquoise |
| FGF1 | turquoise |
| FGF7 | turquoise |
| FGF9 | turquoise |
| FGFBP1 | turquoise |
| FGFR1OP | turquoise |
| FGFR1OP2 | turquoise |
| FGL2 | turquoise |
| FHIT | turquoise |
| FHL3 | turquoise |
| FIBCD1 | turquoise |
| FIBP | turquoise |
| FICD | turquoise |
| FIGN | turquoise |
| FILIP1L | turquoise |
| FITM2 | turquoise |
| FKBP14 | turquoise |
| FKBP1A | turquoise |
| FKBP2 | turquoise |
| FKBP5 | turquoise |
| FKBP8 | turquoise |
| FKBP9 | turquoise |
| FKSG48 | turquoise |
| FKTN | turquoise |
| FLII | turquoise |
| FLJ20021 | turquoise |
| FLNB-AS1 | turquoise |
| FLNC | turquoise |
| FLOT1 | turquoise |
| FLOT2 | turquoise |
| FLT1 | turquoise |
| FLVCR1 | turquoise |
| FMN1 | turquoise |
| FMNL2 | turquoise |
| FMO4 | turquoise |
| FMR1 | turquoise |
| FMR1-IT1 | turquoise |
| FNBP1L | turquoise |
| FNBP4 | turquoise |
| FNDC3A | turquoise |
| FNDC3B | turquoise |
| FNIP1 | turquoise |
| FNIP2 | turquoise |
| FNTA | turquoise |
| FOCAD | turquoise |
| FOSL1 | turquoise |
| FOXC1 | turquoise |
| FOXD2 | turquoise |
| FOXJ3 | turquoise |
| FOXK1 | turquoise |
| FOXN2 | turquoise |
| FOXN3 | turquoise |
| FOXO1 | turquoise |
| FOXO3 | turquoise |
| FOXP1 | turquoise |
| FOXP2 | turquoise |
| FOXP4 | turquoise |
| FOXP4-AS1 | turquoise |
| FOXS1 | turquoise |
| FP236383.10 | turquoise |
| FP671120.4 | turquoise |
| FP671120.7 | turquoise |
| FPGS | turquoise |
| FPGT | turquoise |
| FRA10AC1 | turquoise |
| FREM2 | turquoise |
| FRG1 | turquoise |
| FRG1B | turquoise |
| FRK | turquoise |
| FRMD5 | turquoise |
| FRRS1 | turquoise |
| FRYL | turquoise |
| FSD1L | turquoise |
| FSTL3 | turquoise |
| FTCDNL1 | turquoise |
| FTH1 | turquoise |
| FTH1P4 | turquoise |
| FTL | turquoise |
| FTO-IT1 | turquoise |
| FTX | turquoise |
| FTX_1 | turquoise |
| FUBP1 | turquoise |
| FUBP3 | turquoise |
| FUOM | turquoise |
| FUT10 | turquoise |
| FUT11 | turquoise |
| FUT4 | turquoise |
| FUT8 | turquoise |
| FXR2 | turquoise |
| FXYD5 | turquoise |
| FYB | turquoise |
| FYN | turquoise |
| FZD2 | turquoise |
| FZD3 | turquoise |
| FZD5 | turquoise |
| FZD6 | turquoise |
| G2E3 | turquoise |
| G3BP1 | turquoise |
| G3BP2 | turquoise |
| G6PC3 | turquoise |
| GAA | turquoise |
| GAB1 | turquoise |
| GABARAP | turquoise |
| GABPA | turquoise |
| GABPB1 | turquoise |
| GABPB1-AS1 | turquoise |
| GABPB2 | turquoise |
| GABRD | turquoise |
| GABRE | turquoise |
| GADD45A | turquoise |
| GALE | turquoise |
| GALK2 | turquoise |
| GALM | turquoise |
| GALNT1 | turquoise |
| GALNT11 | turquoise |
| GALNT13 | turquoise |
| GALNT2 | turquoise |
| GALNT7 | turquoise |
| GAMT | turquoise |
| GAN | turquoise |
| GANC | turquoise |
| GAPVD1 | turquoise |
| GAREM | turquoise |
| GAS2L1 | turquoise |
| GAS2L3 | turquoise |
| GAS5-AS1 | turquoise |
| GAS6-AS1 | turquoise |
| GAS8-AS1 | turquoise |
| GATA2-AS1 | turquoise |
| GATAD1 | turquoise |
| GATAD2B | turquoise |
| GATS | turquoise |
| GATSL2 | turquoise |
| GBA | turquoise |
| GBE1 | turquoise |
| GCA | turquoise |
| GCAT | turquoise |
| GCC1 | turquoise |
| GCC2 | turquoise |
| GCDH | turquoise |
| GCFC2 | turquoise |
| GCHFR | turquoise |
| GCNT1 | turquoise |
| GCNT2 | turquoise |
| GCNT4 | turquoise |
| GCSAM | turquoise |
| GDAP2 | turquoise |
| GDF11 | turquoise |
| GDPD1 | turquoise |
| GEM | turquoise |
| GEMIN4 | turquoise |
| GEMIN5 | turquoise |
| GEN1 | turquoise |
| GFPT1 | turquoise |
| GFRA1 | turquoise |
| GGCX | turquoise |
| GGNBP2 | turquoise |
| GGT1 | turquoise |
| GGT5 | turquoise |
| GHDC | turquoise |
| GHRL | turquoise |
| GID4 | turquoise |
| GIGYF2 | turquoise |
| GIMAP5 | turquoise |
| GIN1 | turquoise |
| GIPC1 | turquoise |
| GIT1 | turquoise |
| GIT2 | turquoise |
| GJC1 | turquoise |
| GK | turquoise |
| GK-AS1 | turquoise |
| GK-IT1 | turquoise |
| GK5 | turquoise |
| GKAP1 | turquoise |
| GLA | turquoise |
| GLB1 | turquoise |
| GLCCI1 | turquoise |
| GLCE | turquoise |
| GLI1 | turquoise |
| GLIPR1 | turquoise |
| GLIS2 | turquoise |
| GLIS2-AS1 | turquoise |
| GLIS3 | turquoise |
| GLMN | turquoise |
| GLS | turquoise |
| GLTSCR1L | turquoise |
| GLTSCR2 | turquoise |
| GLUD1P3 | turquoise |
| GLYATL2 | turquoise |
| GMCL1 | turquoise |
| GMDS-AS1 | turquoise |
| GMEB1 | turquoise |
| GMFB | turquoise |
| GMPPB | turquoise |
| GMPR | turquoise |
| GMPR2 | turquoise |
| GNA11 | turquoise |
| GNA13 | turquoise |
| GNA14 | turquoise |
| GNA15 | turquoise |
| GNAI1 | turquoise |
| GNAI3 | turquoise |
| GNAL | turquoise |
| GNAS | turquoise |
| GNB2 | turquoise |
| GNB4 | turquoise |
| GNE | turquoise |
| GNG12 | turquoise |
| GNL1 | turquoise |
| GNL3L | turquoise |
| GNPDA2 | turquoise |
| GNPTAB | turquoise |
| GNPTG | turquoise |
| GNRH1 | turquoise |
| GOLGA1 | turquoise |
| GOLGA3 | turquoise |
| GOLGA4 | turquoise |
| GOLGA6L9 | turquoise |
| GOLGA8A | turquoise |
| GOLGB1 | turquoise |
| GOLIM4 | turquoise |
| GOLT1A | turquoise |
| GON4L | turquoise |
| GOPC | turquoise |
| GORAB | turquoise |
| GOSR1 | turquoise |
| GOSR2 | turquoise |
| GPAA1 | turquoise |
| GPALPP1 | turquoise |
| GPAM | turquoise |
| GPANK1 | turquoise |
| GPATCH11 | turquoise |
| GPATCH2 | turquoise |
| GPATCH2L | turquoise |
| GPATCH3 | turquoise |
| GPATCH8 | turquoise |
| GPBP1 | turquoise |
| GPBP1L1 | turquoise |
| GPC1 | turquoise |
| GPC4 | turquoise |
| GPC6 | turquoise |
| GPCPD1 | turquoise |
| GPD2 | turquoise |
| GPHN | turquoise |
| GPKOW | turquoise |
| GPR107 | turquoise |
| GPR108 | turquoise |
| GPR110 | turquoise |
| GPR125 | turquoise |
| GPR126 | turquoise |
| GPR132 | turquoise |
| GPR137 | turquoise |
| GPR141 | turquoise |
| GPR153 | turquoise |
| GPR155 | turquoise |
| GPR160 | turquoise |
| GPR171 | turquoise |
| GPR174 | turquoise |
| GPR18 | turquoise |
| GPR180 | turquoise |
| GPR183 | turquoise |
| GPR27 | turquoise |
| GPR34 | turquoise |
| GPR75 | turquoise |
| GPR82 | turquoise |
| GPR89A | turquoise |
| GPR89B | turquoise |
| GPR97 | turquoise |
| GPR98 | turquoise |
| GPRASP1 | turquoise |
| GPRC5C | turquoise |
| GPRC5D | turquoise |
| GPRIN3 | turquoise |
| GPSM2 | turquoise |
| GPX1P1 | turquoise |
| GPX4 | turquoise |
| GRAMD1A | turquoise |
| GRAMD1B | turquoise |
| GRAMD1C | turquoise |
| GRAMD3 | turquoise |
| GRB10 | turquoise |
| GRHL1 | turquoise |
| GRHPR | turquoise |
| GRID2IP | turquoise |
| GRINA | turquoise |
| GRIP1 | turquoise |
| GRK4 | turquoise |
| GRK6 | turquoise |
| GRPEL2 | turquoise |
| GRWD1 | turquoise |
| GS1-124K5.10 | turquoise |
| GS1-124K5.2 | turquoise |
| GS1-124K5.3 | turquoise |
| GS1-124K5.6 | turquoise |
| GS1-279B7.2 | turquoise |
| GS1-293C5.1 | turquoise |
| GS1-358P8.4 | turquoise |
| GSAP | turquoise |
| GSDMD | turquoise |
| GSE1 | turquoise |
| GSK3A | turquoise |
| GSK3B | turquoise |
| GSPT1 | turquoise |
| GSTCD | turquoise |
| GSTK1 | turquoise |
| GSTM1 | turquoise |
| GSTM4 | turquoise |
| GSTP1 | turquoise |
| GTDC1 | turquoise |
| GTF2A1 | turquoise |
| GTF2F1 | turquoise |
| GTF2H1 | turquoise |
| GTF2H2C | turquoise |
| GTF2I | turquoise |
| GTF3C4 | turquoise |
| GTPBP10 | turquoise |
| GTPBP6 | turquoise |
| GTPBP8 | turquoise |
| GUCA2A | turquoise |
| GUCD1 | turquoise |
| GUCY1A2 | turquoise |
| GUCY1A3 | turquoise |
| GUCY1B2 | turquoise |
| GUCY1B3 | turquoise |
| GUCY2C | turquoise |
| GUF1 | turquoise |
| GULP1 | turquoise |
| GUSB | turquoise |
| GUSBP1 | turquoise |
| GVINP1 | turquoise |
| GVQW1 | turquoise |
| GXYLT1 | turquoise |
| GXYLT2 | turquoise |
| GYS1 | turquoise |
| GZF1 | turquoise |
| H1FX | turquoise |
| H2AFJ | turquoise |
| H2AFY | turquoise |
| H3F3A | turquoise |
| H3F3AP6 | turquoise |
| H3F3B | turquoise |
| HACE1 | turquoise |
| HACL1 | turquoise |
| HADHA | turquoise |
| HADHB | turquoise |
| HAGH | turquoise |
| HAS3 | turquoise |
| HAUS3 | turquoise |
| HBP1 | turquoise |
| HBS1L | turquoise |
| HCFC1R1 | turquoise |
| HCFC2 | turquoise |
| HCG11 | turquoise |
| HCG18 | turquoise |
| HCG27 | turquoise |
| HCG4 | turquoise |
| HDAC11 | turquoise |
| HDAC3 | turquoise |
| HDAC4 | turquoise |
| HDAC5 | turquoise |
| HDAC8 | turquoise |
| HDAC9 | turquoise |
| HDC | turquoise |
| HDDC3 | turquoise |
| HDGFRP3 | turquoise |
| HDHD1 | turquoise |
| HDHD2 | turquoise |
| HDX | turquoise |
| HEATR1 | turquoise |
| HEATR5A | turquoise |
| HEATR5B | turquoise |
| HEATR6 | turquoise |
| HEBP1 | turquoise |
| HECA | turquoise |
| HECTD1 | turquoise |
| HECTD2 | turquoise |
| HECTD3 | turquoise |
| HECTD4 | turquoise |
| HECW2 | turquoise |
| HEIH | turquoise |
| HELB | turquoise |
| HELLS | turquoise |
| HELQ | turquoise |
| HELZ | turquoise |
| HERC1 | turquoise |
| HERC2 | turquoise |
| HERC2P9 | turquoise |
| HERC3 | turquoise |
| HERC4 | turquoise |
| HERC5 | turquoise |
| HERC6 | turquoise |
| HERPUD2 | turquoise |
| HESX1 | turquoise |
| HEXA | turquoise |
| HEXIM2 | turquoise |
| HGF | turquoise |
| HHAT | turquoise |
| HHATL | turquoise |
| HHLA2 | turquoise |
| HHLA3 | turquoise |
| HIAT1 | turquoise |
| HIATL1 | turquoise |
| HIATL2 | turquoise |
| HIBCH | turquoise |
| HIF1A | turquoise |
| HIF1A-AS2 | turquoise |
| HIF1AN | turquoise |
| HIGD2A | turquoise |
| HINT2 | turquoise |
| HINT3 | turquoise |
| HIPK1 | turquoise |
| HIPK2 | turquoise |
| HIPK3 | turquoise |
| HIRIP3 | turquoise |
| HIST1H1PS1 | turquoise |
| HIST1H2APS3 | turquoise |
| HIST2H2BC | turquoise |
| HIVEP1 | turquoise |
| HIVEP2 | turquoise |
| HIVEP3 | turquoise |
| HK1 | turquoise |
| HK2 | turquoise |
| HKR1 | turquoise |
| HLA-A | turquoise |
| HLA-DQB1-AS1 | turquoise |
| HLA-DQB2 | turquoise |
| HLA-F-AS1 | turquoise |
| HLA-W | turquoise |
| HLCS | turquoise |
| HLF | turquoise |
| HM13-IT1 | turquoise |
| HMBOX1 | turquoise |
| HMCN1 | turquoise |
| HMG20A | turquoise |
| HMG20B | turquoise |
| HMGA1P2 | turquoise |
| HMGA1P4 | turquoise |
| HMGB1P31 | turquoise |
| HMGCL | turquoise |
| HMGCR | turquoise |
| HMGCS1 | turquoise |
| HMGN1P36 | turquoise |
| HMGN1P8 | turquoise |
| HMGN2P3 | turquoise |
| HMGN2P46 | turquoise |
| HMGN2P5 | turquoise |
| HMGN3-AS1 | turquoise |
| HMOX2 | turquoise |
| HNF1A-AS1 | turquoise |
| HNF4G | turquoise |
| HNRNPA0 | turquoise |
| HNRNPA1L2 | turquoise |
| HNRNPA1P27 | turquoise |
| HNRNPA2B1 | turquoise |
| HNRNPA3 | turquoise |
| HNRNPCP7 | turquoise |
| HNRNPDL | turquoise |
| HNRNPH1 | turquoise |
| HNRNPH2 | turquoise |
| HNRNPH3 | turquoise |
| HNRNPLL | turquoise |
| HNRNPU | turquoise |
| HNRNPU-AS1 | turquoise |
| HNRNPUL1 | turquoise |
| HNRNPUP1 | turquoise |
| HOMER1 | turquoise |
| HOMER2 | turquoise |
| HOMER3 | turquoise |
| HOMEZ | turquoise |
| HOOK1 | turquoise |
| HOOK3 | turquoise |
| HORMAD1 | turquoise |
| HOXB-AS2 | turquoise |
| HOXD9 | turquoise |
| HP1BP3 | turquoise |
| HPCAL4 | turquoise |
| HPGD | turquoise |
| HPN | turquoise |
| HPS1 | turquoise |
| HPS3 | turquoise |
| HPS4 | turquoise |
| HPS6 | turquoise |
| HRAS | turquoise |
| HRASLS5 | turquoise |
| HS1BP3 | turquoise |
| HS2ST1 | turquoise |
| HS3ST3A1 | turquoise |
| HS3ST3B1 | turquoise |
| HS3ST5 | turquoise |
| HS6ST1 | turquoise |
| HSD11B1L | turquoise |
| HSD17B12 | turquoise |
| HSD17B14 | turquoise |
| HSD17B8 | turquoise |
| HSD3B7 | turquoise |
| HSDL1 | turquoise |
| HSF1 | turquoise |
| HSP90AB2P | turquoise |
| HSPA12A | turquoise |
| HSPA4L | turquoise |
| HSPB1 | turquoise |
| HSPB11 | turquoise |
| HSPBAP1 | turquoise |
| HSPBP1 | turquoise |
| HSPE1P18 | turquoise |
| HSPE1P2 | turquoise |
| HSPH1 | turquoise |
| HTR2B | turquoise |
| HTR7P1 | turquoise |
| HTT | turquoise |
| HUS1 | turquoise |
| HUWE1 | turquoise |
| HYAL3 | turquoise |
| HYKK | turquoise |
| IBA57 | turquoise |
| IBTK | turquoise |
| ICA1L | turquoise |
| ICE1 | turquoise |
| ICE2 | turquoise |
| ICK | turquoise |
| ID1 | turquoise |
| IDE | turquoise |
| IDH3A | turquoise |
| IDH3B | turquoise |
| IDH3G | turquoise |
| IDNK | turquoise |
| IDS | turquoise |
| IER3 | turquoise |
| IER5 | turquoise |
| IFI16 | turquoise |
| IFI27 | turquoise |
| IFI27L2 | turquoise |
| IFI35 | turquoise |
| IFI44 | turquoise |
| IFI44L | turquoise |
| IFI6 | turquoise |
| IFIT5 | turquoise |
| IFITM1 | turquoise |
| IFITM2 | turquoise |
| IFITM3 | turquoise |
| IFNAR1 | turquoise |
| IFNAR2 | turquoise |
| IFNG | turquoise |
| IFRD1 | turquoise |
| IFRD2 | turquoise |
| IFT74 | turquoise |
| IFT80 | turquoise |
| IFT81 | turquoise |
| IGBP1 | turquoise |
| IGBP1-AS1 | turquoise |
| IGF1R | turquoise |
| IGF2 | turquoise |
| IGF2R | turquoise |
| IGFBP4 | turquoise |
| IGFBP6 | turquoise |
| IGHD1-1 | turquoise |
| IGHD2-15 | turquoise |
| IGHD2-2 | turquoise |
| IGHD4-23 | turquoise |
| IGHD4-4 | turquoise |
| IGHD6-19 | turquoise |
| IGHD6-6 | turquoise |
| IGHV3-79 | turquoise |
| IGIP | turquoise |
| IGSF3 | turquoise |
| IGSF8 | turquoise |
| IGSF9B | turquoise |
| IKBKAP | turquoise |
| IKBKB | turquoise |
| IKBKG | turquoise |
| IKZF2 | turquoise |
| IKZF3 | turquoise |
| IKZF5 | turquoise |
| IL10RB | turquoise |
| IL10RB-AS1 | turquoise |
| IL15 | turquoise |
| IL17RC | turquoise |
| IL18R1 | turquoise |
| IL1A | turquoise |
| IL1R1 | turquoise |
| IL1RAP | turquoise |
| IL27RA | turquoise |
| IL31RA | turquoise |
| IL32 | turquoise |
| IL36RN | turquoise |
| IL37 | turquoise |
| IL6ST | turquoise |
| IL7 | turquoise |
| ILKAP | turquoise |
| ILVBL | turquoise |
| IMMP1L | turquoise |
| IMMP2L | turquoise |
| IMP3 | turquoise |
| IMPA1 | turquoise |
| IMPACT | turquoise |
| IMPAD1 | turquoise |
| IMPDH1 | turquoise |
| IMPDH2 | turquoise |
| INADL | turquoise |
| INAFM1 | turquoise |
| INE1 | turquoise |
| INF2 | turquoise |
| ING2 | turquoise |
| ING3 | turquoise |
| INIP | turquoise |
| INO80 | turquoise |
| INO80D | turquoise |
| INPP4A | turquoise |
| INPP4B | turquoise |
| INPP5B | turquoise |
| INPP5F | turquoise |
| INSIG2 | turquoise |
| INSR | turquoise |
| INTS10 | turquoise |
| INTS12 | turquoise |
| INTS2 | turquoise |
| INTS4 | turquoise |
| INTS5 | turquoise |
| INTS6 | turquoise |
| INTS8 | turquoise |
| INTS9 | turquoise |
| INTU | turquoise |
| INVS | turquoise |
| IP6K3 | turquoise |
| IPCEF1 | turquoise |
| IPMK | turquoise |
| IPO11 | turquoise |
| IPO13 | turquoise |
| IPO5P1 | turquoise |
| IPO7 | turquoise |
| IPO8 | turquoise |
| IPP | turquoise |
| IPPK | turquoise |
| IQCB1 | turquoise |
| IQCH-AS1 | turquoise |
| IQGAP1 | turquoise |
| IQGAP2 | turquoise |
| IQSEC2 | turquoise |
| IRAK1BP1 | turquoise |
| IRAK2 | turquoise |
| IRAK3 | turquoise |
| IRAK4 | turquoise |
| IREB2 | turquoise |
| IRF2BP1 | turquoise |
| IRF2BP2 | turquoise |
| IRGQ | turquoise |
| IRS1 | turquoise |
| IRX3 | turquoise |
| IRX6 | turquoise |
| ISG15 | turquoise |
| ISOC2 | turquoise |
| ISPD | turquoise |
| ITCH | turquoise |
| ITCH-IT1 | turquoise |
| ITFG1 | turquoise |
| ITFG3 | turquoise |
| ITGA1 | turquoise |
| ITGA2 | turquoise |
| ITGA3 | turquoise |
| ITGA4 | turquoise |
| ITGA9 | turquoise |
| ITGAV | turquoise |
| ITGB3BP | turquoise |
| ITGB8 | turquoise |
| ITK | turquoise |
| ITM2C | turquoise |
| ITPK1 | turquoise |
| ITPKA | turquoise |
| ITPR1 | turquoise |
| ITPR2 | turquoise |
| ITPRIPL2 | turquoise |
| ITSN1 | turquoise |
| ITSN2 | turquoise |
| IVL | turquoise |
| IVNS1ABP | turquoise |
| JADE1 | turquoise |
| JADE3 | turquoise |
| JAG1 | turquoise |
| JAGN1 | turquoise |
| JAK2 | turquoise |
| JARID2 | turquoise |
| JAZF1 | turquoise |
| JKAMP | turquoise |
| JMJD1C | turquoise |
| JMJD1C-AS1 | turquoise |
| JMJD8 | turquoise |
| JMY | turquoise |
| JOSD1 | turquoise |
| JOSD2 | turquoise |
| JPH1 | turquoise |
| JPX | turquoise |
| JRK | turquoise |
| JRKL | turquoise |
| JUP | turquoise |
| KALRN | turquoise |
| KANK1 | turquoise |
| KANSL1 | turquoise |
| KANSL1-AS1 | turquoise |
| KANSL1L | turquoise |
| KANSL3 | turquoise |
| KANTR | turquoise |
| KAT2B | turquoise |
| KAT5 | turquoise |
| KAT6A | turquoise |
| KAT6B | turquoise |
| KAT7 | turquoise |
| KATNAL1 | turquoise |
| KATNAL2 | turquoise |
| KATNB1 | turquoise |
| KATNBL1 | turquoise |
| KB-1208A12.3 | turquoise |
| KB-1410C5.5 | turquoise |
| KB-1440D3.13 | turquoise |
| KB-1460A1.5 | turquoise |
| KB-1507C5.2 | turquoise |
| KB-1507C5.4 | turquoise |
| KB-1991G8.1 | turquoise |
| KB-226F1.2 | turquoise |
| KB-318B8.7 | turquoise |
| KB-431C1.4 | turquoise |
| KB-431C1.5 | turquoise |
| KBTBD11 | turquoise |
| KBTBD12 | turquoise |
| KBTBD2 | turquoise |
| KBTBD6 | turquoise |
| KBTBD7 | turquoise |
| KBTBD8 | turquoise |
| KCNA3 | turquoise |
| KCNAB1 | turquoise |
| KCND2 | turquoise |
| KCND3 | turquoise |
| KCNJ12 | turquoise |
| KCNJ2 | turquoise |
| KCNMA1 | turquoise |
| KCNMB4 | turquoise |
| KCNN3 | turquoise |
| KCNN4 | turquoise |
| KCNQ3 | turquoise |
| KCNQ5 | turquoise |
| KCTD11 | turquoise |
| KCTD14 | turquoise |
| KCTD17 | turquoise |
| KCTD18 | turquoise |
| KCTD20 | turquoise |
| KCTD3 | turquoise |
| KCTD5 | turquoise |
| KCTD6 | turquoise |
| KCTD7 | turquoise |
| KCTD9 | turquoise |
| KDELC2 | turquoise |
| KDELR1 | turquoise |
| KDM1B | turquoise |
| KDM2A | turquoise |
| KDM2B | turquoise |
| KDM3A | turquoise |
| KDM3B | turquoise |
| KDM4A | turquoise |
| KDM4A-AS1 | turquoise |
| KDM4C | turquoise |
| KDM5A | turquoise |
| KDM5B | turquoise |
| KDM5D | turquoise |
| KDM6A | turquoise |
| KDM7A | turquoise |
| KDR | turquoise |
| KDSR | turquoise |
| KEAP1 | turquoise |
| KHDRBS2 | turquoise |
| KHNYN | turquoise |
| KIAA0020 | turquoise |
| KIAA0100 | turquoise |
| KIAA0196 | turquoise |
| KIAA0226 | turquoise |
| KIAA0232 | turquoise |
| KIAA0355 | turquoise |
| KIAA0368 | turquoise |
| KIAA0391 | turquoise |
| KIAA0430 | turquoise |
| KIAA0586 | turquoise |
| KIAA0753 | turquoise |
| KIAA0895 | turquoise |
| KIAA0922 | turquoise |
| KIAA0930 | turquoise |
| KIAA1033 | turquoise |
| KIAA1107 | turquoise |
| KIAA1109 | turquoise |
| KIAA1143 | turquoise |
| KIAA1147 | turquoise |
| KIAA1161 | turquoise |
| KIAA1217 | turquoise |
| KIAA1244 | turquoise |
| KIAA1328 | turquoise |
| KIAA1429 | turquoise |
| KIAA1468 | turquoise |
| KIAA1549 | turquoise |
| KIAA1551 | turquoise |
| KIAA1586 | turquoise |
| KIAA1598 | turquoise |
| KIAA1644 | turquoise |
| KIAA1671 | turquoise |
| KIAA1715 | turquoise |
| KIAA1841 | turquoise |
| KIAA1919 | turquoise |
| KIAA1958 | turquoise |
| KIAA2013 | turquoise |
| KIAA2018 | turquoise |
| KIAA2026 | turquoise |
| KIDINS220 | turquoise |
| KIF13A | turquoise |
| KIF16B | turquoise |
| KIF1B | turquoise |
| KIF21A | turquoise |
| KIF24 | turquoise |
| KIF27 | turquoise |
| KIF2A | turquoise |
| KIF3A | turquoise |
| KIF3B | turquoise |
| KIF5B | turquoise |
| KIFAP3 | turquoise |
| KIN | turquoise |
| KITLG | turquoise |
| KIZ | turquoise |
| KLB | turquoise |
| KLC1 | turquoise |
| KLF12 | turquoise |
| KLF16 | turquoise |
| KLF3 | turquoise |
| KLF7 | turquoise |
| KLF7-IT1 | turquoise |
| KLF8 | turquoise |
| KLHDC1 | turquoise |
| KLHDC10 | turquoise |
| KLHDC2 | turquoise |
| KLHDC8B | turquoise |
| KLHL15 | turquoise |
| KLHL18 | turquoise |
| KLHL2 | turquoise |
| KLHL20 | turquoise |
| KLHL21 | turquoise |
| KLHL23 | turquoise |
| KLHL24 | turquoise |
| KLHL26 | turquoise |
| KLHL28 | turquoise |
| KLHL29 | turquoise |
| KLHL3 | turquoise |
| KLHL36 | turquoise |
| KLHL42 | turquoise |
| KLHL5 | turquoise |
| KLHL7 | turquoise |
| KLHL8 | turquoise |
| KLHL9 | turquoise |
| KLK11 | turquoise |
| KLRAP1 | turquoise |
| KLRG1 | turquoise |
| KLRG2 | turquoise |
| KMT2A | turquoise |
| KMT2C | turquoise |
| KMT2D | turquoise |
| KMT2E | turquoise |
| KPNA1 | turquoise |
| KPNA3 | turquoise |
| KPNA4 | turquoise |
| KPNA5 | turquoise |
| KPNA6 | turquoise |
| KRAS | turquoise |
| KRBOX4 | turquoise |
| KRIT1 | turquoise |
| KRR1 | turquoise |
| KRR1P1 | turquoise |
| KRT18 | turquoise |
| KRT19 | turquoise |
| KRT7 | turquoise |
| KRT8 | turquoise |
| KRT87P | turquoise |
| KRT8P11 | turquoise |
| KRT8P3 | turquoise |
| KRT8P33 | turquoise |
| KRT8P45 | turquoise |
| KRTAP5-AS1 | turquoise |
| KTN1 | turquoise |
| KXD1 | turquoise |
| L3HYPDH | turquoise |
| L3MBTL3 | turquoise |
| L3MBTL4 | turquoise |
| LA16c-306A4.1 | turquoise |
| LA16c-321D4.2 | turquoise |
| LA16c-360H6.1 | turquoise |
| LA16c-361A3.3 | turquoise |
| LA16c-380F5.3 | turquoise |
| LACC1 | turquoise |
| LACE1 | turquoise |
| LAMA2 | turquoise |
| LAMA3 | turquoise |
| LAMB3 | turquoise |
| LAMP2 | turquoise |
| LANCL1 | turquoise |
| LAPTM4A | turquoise |
| LARGE | turquoise |
| LARP1B | turquoise |
| LARP4 | turquoise |
| LARP4B | turquoise |
| LARP7 | turquoise |
| LARS | turquoise |
| LASP1 | turquoise |
| LATS1 | turquoise |
| LCA5 | turquoise |
| LCA5L | turquoise |
| LCLAT1 | turquoise |
| LCMT2 | turquoise |
| LCOR | turquoise |
| LCORL | turquoise |
| LDLRAD4 | turquoise |
| LDLRAP1 | turquoise |
| LDOC1 | turquoise |
| LDOC1L | turquoise |
| LECT1 | turquoise |
| LEF1 | turquoise |
| LEMD3 | turquoise |
| LENG1 | turquoise |
| LENG9 | turquoise |
| LEPROTL1 | turquoise |
| LETM2 | turquoise |
| LGALS3 | turquoise |
| LGALS3BP | turquoise |
| LGALS8 | turquoise |
| LGI2 | turquoise |
| LGR4 | turquoise |
| LGSN | turquoise |
| LHFPL2 | turquoise |
| LHFPL3 | turquoise |
| LHFPL4 | turquoise |
| LHPP | turquoise |
| LIFR | turquoise |
| LIG4 | turquoise |
| LILRA2 | turquoise |
| LIMA1 | turquoise |
| LIMS1 | turquoise |
| LIN52 | turquoise |
| LIN54 | turquoise |
| LIN7C | turquoise |
| LINC-PINT | turquoise |
| LINC00162 | turquoise |
| LINC00205 | turquoise |
| LINC00216 | turquoise |
| LINC00294 | turquoise |
| LINC00342 | turquoise |
| LINC00473 | turquoise |
| LINC00476 | turquoise |
| LINC00578 | turquoise |
| LINC00630 | turquoise |
| LINC00641 | turquoise |
| LINC00649 | turquoise |
| LINC00654 | turquoise |
| LINC00657 | turquoise |
| LINC00662 | turquoise |
| LINC00667 | turquoise |
| LINC00674 | turquoise |
| LINC00852 | turquoise |
| LINC00854 | turquoise |
| LINC00861 | turquoise |
| LINC00863 | turquoise |
| LINC00865 | turquoise |
| LINC00883 | turquoise |
| LINC00885 | turquoise |
| LINC00886 | turquoise |
| LINC00894 | turquoise |
| LINC00909 | turquoise |
| LINC00921 | turquoise |
| LINC00960 | turquoise |
| LINC00969 | turquoise |
| LINC00973 | turquoise |
| LINC00997 | turquoise |
| LINC01004 | turquoise |
| LINC01023 | turquoise |
| LINC01057 | turquoise |
| LINC01088 | turquoise |
| LINC01106 | turquoise |
| LINC01123 | turquoise |
| LINC01125 | turquoise |
| LINC01128 | turquoise |
| LINC01137 | turquoise |
| LINC01138 | turquoise |
| LINC01184 | turquoise |
| LINC01214 | turquoise |
| LINC01232 | turquoise |
| LINC01278 | turquoise |
| LINC01290 | turquoise |
| LINC01291 | turquoise |
| LINC01348 | turquoise |
| LINC01355 | turquoise |
| LINC01376 | turquoise |
| LINC01389 | turquoise |
| LINC01420 | turquoise |
| LINC01480 | turquoise |
| LINC01504 | turquoise |
| LINC01511 | turquoise |
| LINC01534 | turquoise |
| LINC01540 | turquoise |
| LINC01547 | turquoise |
| LINC01560 | turquoise |
| LINC01578 | turquoise |
| LINS | turquoise |
| LIPM | turquoise |
| LIPT1 | turquoise |
| LIX1L | turquoise |
| LL0XNC01-131B10.2 | turquoise |
| LL0XNC01-237H1.2 | turquoise |
| LL0XNC01-36H8.1 | turquoise |
| LL0XNC01-7P3.1 | turquoise |
| LL21NC02-1C16.2 | turquoise |
| LL21NC02-21A1.1 | turquoise |
| LMAN1 | turquoise |
| LMAN2 | turquoise |
| LMAN2L | turquoise |
| LMBR1 | turquoise |
| LMBRD2 | turquoise |
| LMF2 | turquoise |
| LMLN | turquoise |
| LMNA | turquoise |
| LMO3 | turquoise |
| LMO4 | turquoise |
| LMO7-AS1 | turquoise |
| LMTK2 | turquoise |
| LNPEP | turquoise |
| LNX2 | turquoise |
| LOH12CR1 | turquoise |
| LONP1 | turquoise |
| LONP2 | turquoise |
| LONRF1 | turquoise |
| LONRF2 | turquoise |
| LONRF3 | turquoise |
| LOXL4 | turquoise |
| LPAR6 | turquoise |
| LPCAT1 | turquoise |
| LPCAT2 | turquoise |
| LPGAT1 | turquoise |
| LPIN1 | turquoise |
| LPIN2 | turquoise |
| LPP | turquoise |
| LPP-AS2 | turquoise |
| LPPR2 | turquoise |
| LPPR4 | turquoise |
| LRBA | turquoise |
| LRCH2 | turquoise |
| LRCH3 | turquoise |
| LRFN1 | turquoise |
| LRFN3 | turquoise |
| LRIF1 | turquoise |
| LRIG2 | turquoise |
| LRIG3 | turquoise |
| LRP10 | turquoise |
| LRP12 | turquoise |
| LRP2 | turquoise |
| LRP2BP | turquoise |
| LRP3 | turquoise |
| LRP6 | turquoise |
| LRPAP1 | turquoise |
| LRRC16A | turquoise |
| LRRC28 | turquoise |
| LRRC31 | turquoise |
| LRRC37A16P | turquoise |
| LRRC37A17P | turquoise |
| LRRC37A2 | turquoise |
| LRRC37A3 | turquoise |
| LRRC37A4P | turquoise |
| LRRC37B | turquoise |
| LRRC37BP1 | turquoise |
| LRRC39 | turquoise |
| LRRC40 | turquoise |
| LRRC57 | turquoise |
| LRRC58 | turquoise |
| LRRC61 | turquoise |
| LRRC75A | turquoise |
| LRRC8A | turquoise |
| LRRC8B | turquoise |
| LRRC8C | turquoise |
| LRRCC1 | turquoise |
| LRRFIP1P1 | turquoise |
| LRRFIP2 | turquoise |
| LRRK1 | turquoise |
| LRRN1 | turquoise |
| LSAMP | turquoise |
| LSM11 | turquoise |
| LSM8 | turquoise |
| LSMEM1 | turquoise |
| LSR | turquoise |
| LTB4R2 | turquoise |
| LTBR | turquoise |
| LTN1 | turquoise |
| LUCAT1 | turquoise |
| LUZP1 | turquoise |
| LY6E | turquoise |
| LY75 | turquoise |
| LYN | turquoise |
| LYPLA2 | turquoise |
| LYPLAL1 | turquoise |
| LYRM5 | turquoise |
| LYRM7 | turquoise |
| LYSMD3 | turquoise |
| LYSMD4 | turquoise |
| LYST | turquoise |
| LZIC | turquoise |
| MACC1 | turquoise |
| MACC1-AS1 | turquoise |
| MACF1 | turquoise |
| MACROD2 | turquoise |
| MAF | turquoise |
| MAF1 | turquoise |
| MAGED2 | turquoise |
| MAGEE1 | turquoise |
| MAGEH1 | turquoise |
| MAGI1 | turquoise |
| MAGI2 | turquoise |
| MAGI2-AS3 | turquoise |
| MAGI3 | turquoise |
| MAGOH2P | turquoise |
| MAK | turquoise |
| MAK16 | turquoise |
| MALAT1 | turquoise |
| MALT1 | turquoise |
| MAML1 | turquoise |
| MAML2 | turquoise |
| MAML3 | turquoise |
| MAN1A1 | turquoise |
| MAN1A2 | turquoise |
| MAN1B1 | turquoise |
| MAN1B1-AS1 | turquoise |
| MAN2A1 | turquoise |
| MANBA | turquoise |
| MANBAL | turquoise |
| MANEA | turquoise |
| MAP10 | turquoise |
| MAP1B | turquoise |
| MAP1LC3A | turquoise |
| MAP1S | turquoise |
| MAP2 | turquoise |
| MAP2K2 | turquoise |
| MAP2K3 | turquoise |
| MAP2K4 | turquoise |
| MAP2K5 | turquoise |
| MAP3K1 | turquoise |
| MAP3K11 | turquoise |
| MAP3K12 | turquoise |
| MAP3K13 | turquoise |
| MAP3K2 | turquoise |
| MAP3K4 | turquoise |
| MAP3K5 | turquoise |
| MAP3K7 | turquoise |
| MAP3K9 | turquoise |
| MAP4K3 | turquoise |
| MAP4K5 | turquoise |
| MAP7D1 | turquoise |
| MAP7D3 | turquoise |
| MAP9 | turquoise |
| MAPK1 | turquoise |
| MAPK10 | turquoise |
| MAPK14 | turquoise |
| MAPK1IP1L | turquoise |
| MAPK3 | turquoise |
| MAPK4 | turquoise |
| MAPK6 | turquoise |
| MAPK7 | turquoise |
| MAPK8 | turquoise |
| MAPK9 | turquoise |
| MAPKAPK2 | turquoise |
| MAPKAPK3 | turquoise |
| MAPKAPK5 | turquoise |
| 1-Mar | turquoise |
| 2-Mar | turquoise |
| 3-Mar | turquoise |
| 6-Mar | turquoise |
| 7-Mar | turquoise |
| MARCKS | turquoise |
| MARK3 | turquoise |
| MARK4 | turquoise |
| MARVELD2 | turquoise |
| MAST4 | turquoise |
| MAST4-AS1 | turquoise |
| MAT2A | turquoise |
| MATN2 | turquoise |
| MAVS | turquoise |
| MBD2 | turquoise |
| MBD4 | turquoise |
| MBD5 | turquoise |
| MBIP | turquoise |
| MBL1P | turquoise |
| MBLAC2 | turquoise |
| MBNL1 | turquoise |
| MBNL1-AS1 | turquoise |
| MBNL2 | turquoise |
| MBNL3 | turquoise |
| MBOAT1 | turquoise |
| MBOAT2 | turquoise |
| MBOAT7 | turquoise |
| MBP | turquoise |
| MBTD1 | turquoise |
| MBTPS1 | turquoise |
| MBTPS2 | turquoise |
| MCAT | turquoise |
| MCC | turquoise |
| MCCC1 | turquoise |
| MCCC1-AS1 | turquoise |
| MCL1 | turquoise |
| MCM3AP | turquoise |
| MCM3AP-AS1 | turquoise |
| MCM9 | turquoise |
| MCOLN2 | turquoise |
| MCOLN3 | turquoise |
| MCPH1 | turquoise |
| MCRS1 | turquoise |
| MCTP1 | turquoise |
| MCTP2 | turquoise |
| MCTS2P | turquoise |
| MCU | turquoise |
| MDFIC | turquoise |
| MDM1 | turquoise |
| MDM2 | turquoise |
| MDM4 | turquoise |
| MDN1 | turquoise |
| ME2 | turquoise |
| MECOM | turquoise |
| MECP2 | turquoise |
| MED1 | turquoise |
| MED11 | turquoise |
| MED12 | turquoise |
| MED13 | turquoise |
| MED13L | turquoise |
| MED14 | turquoise |
| MED16 | turquoise |
| MED17 | turquoise |
| MED21 | turquoise |
| MED23 | turquoise |
| MED28 | turquoise |
| MED29 | turquoise |
| MED4 | turquoise |
| MED6 | turquoise |
| MED7 | turquoise |
| MEF2A | turquoise |
| MEF2C | turquoise |
| MEGF9 | turquoise |
| MEIS1 | turquoise |
| MEIS2 | turquoise |
| MEIS3P1 | turquoise |
| MEIS3P2 | turquoise |
| MEMO1 | turquoise |
| MEPCE | turquoise |
| MERTK | turquoise |
| MESDC1 | turquoise |
| MET | turquoise |
| METRNL | turquoise |
| METTL1 | turquoise |
| METTL10 | turquoise |
| METTL12 | turquoise |
| METTL14 | turquoise |
| METTL15 | turquoise |
| METTL15P1 | turquoise |
| METTL16 | turquoise |
| METTL20 | turquoise |
| METTL21A | turquoise |
| METTL22 | turquoise |
| METTL25 | turquoise |
| METTL4 | turquoise |
| METTL8 | turquoise |
| MEX3C | turquoise |
| MFAP1 | turquoise |
| MFAP3 | turquoise |
| MFHAS1 | turquoise |
| MFN1 | turquoise |
| MFN2 | turquoise |
| MFSD10 | turquoise |
| MFSD11 | turquoise |
| MFSD3 | turquoise |
| MFSD4 | turquoise |
| MFSD5 | turquoise |
| MFSD6 | turquoise |
| MFSD8 | turquoise |
| MGA | turquoise |
| MGAT1 | turquoise |
| MGAT2 | turquoise |
| MGAT4A | turquoise |
| MGAT4B | turquoise |
| MGAT5 | turquoise |
| MGC32805 | turquoise |
| MGEA5 | turquoise |
| MGST2 | turquoise |
| MIA3 | turquoise |
| MIATNB | turquoise |
| MIB1 | turquoise |
| MICA | turquoise |
| MICAL2 | turquoise |
| MICAL3 | turquoise |
| MICALCL | turquoise |
| MICALL1 | turquoise |
| MICE | turquoise |
| MICU3 | turquoise |
| MID1 | turquoise |
| MID1IP1-AS1 | turquoise |
| MID2 | turquoise |
| MIDN | turquoise |
| MIEF2 | turquoise |
| MIEN1 | turquoise |
| MIER1 | turquoise |
| MIER3 | turquoise |
| MIF-AS1 | turquoise |
| MIF4GD | turquoise |
| MIOS | turquoise |
| MIPOL1 | turquoise |
| MIR1205 | turquoise |
| MIR1245A | turquoise |
| MIR1254-1 | turquoise |
| MIR1270 | turquoise |
| MIR1285-1 | turquoise |
| MIR135A1 | turquoise |
| MIR155HG | turquoise |
| MIR181A2HG | turquoise |
| MIR186 | turquoise |
| MIR1972-1 | turquoise |
| MIR221 | turquoise |
| MIR222HG | turquoise |
| MIR2355 | turquoise |
| MIR23A | turquoise |
| MIR23B | turquoise |
| MIR27B | turquoise |
| MIR30C2 | turquoise |
| MIR3125 | turquoise |
| MIR3140 | turquoise |
| MIR3153 | turquoise |
| MIR3164 | turquoise |
| MIR3174 | turquoise |
| MIR3186 | turquoise |
| MIR320E | turquoise |
| MIR3609 | turquoise |
| MIR3671 | turquoise |
| MIR3682 | turquoise |
| MIR374B | turquoise |
| MIR378G | turquoise |
| MIR378H | turquoise |
| MIR378J | turquoise |
| MIR3936 | turquoise |
| MIR421 | turquoise |
| MIR4257 | turquoise |
| MIR4263 | turquoise |
| MIR4284 | turquoise |
| MIR4312 | turquoise |
| MIR4324 | turquoise |
| MIR4420 | turquoise |
| MIR4442 | turquoise |
| MIR4477B | turquoise |
| MIR4480 | turquoise |
| MIR4482 | turquoise |
| MIR4520-1 | turquoise |
| MIR4635 | turquoise |
| MIR4639 | turquoise |
| MIR4664 | turquoise |
| MIR4668 | turquoise |
| MIR4677 | turquoise |
| MIR4768 | turquoise |
| MIR4802 | turquoise |
| MIR499A | turquoise |
| MIR5010 | turquoise |
| MIR5195 | turquoise |
| MIR548AA1 | turquoise |
| MIR548AT | turquoise |
| MIR548D2 | turquoise |
| MIR548V | turquoise |
| MIR553 | turquoise |
| MIR554 | turquoise |
| MIR5581 | turquoise |
| MIR5587 | turquoise |
| MIR559 | turquoise |
| MIR561 | turquoise |
| MIR568 | turquoise |
| MIR5685 | turquoise |
| MIR5690 | turquoise |
| MIR5692A1 | turquoise |
| MIR5692C2 | turquoise |
| MIR570 | turquoise |
| MIR573 | turquoise |
| MIR5739 | turquoise |
| MIR589 | turquoise |
| MIR590 | turquoise |
| MIR593 | turquoise |
| MIR6071 | turquoise |
| MIR6075 | turquoise |
| MIR6124 | turquoise |
| MIR616 | turquoise |
| MIR624 | turquoise |
| MIR635 | turquoise |
| MIR644A | turquoise |
| MIR659 | turquoise |
| MIR663AHG | turquoise |
| MIR6730 | turquoise |
| MIR6739 | turquoise |
| MIR6757 | turquoise |
| MIR6819 | turquoise |
| MIR7111 | turquoise |
| MIR765 | turquoise |
| MIR7848 | turquoise |
| MIR7856 | turquoise |
| MIR93 | turquoise |
| MIRLET7D | turquoise |
| MIS12 | turquoise |
| MIS18BP1 | turquoise |
| MISP | turquoise |
| MITD1 | turquoise |
| MITF | turquoise |
| MKL2 | turquoise |
| MKLN1 | turquoise |
| MKLN1-AS | turquoise |
| MKNK1 | turquoise |
| MKNK2 | turquoise |
| MKRN1 | turquoise |
| MKRN2 | turquoise |
| MLF2 | turquoise |
| MLH1 | turquoise |
| MLH3 | turquoise |
| MLK4 | turquoise |
| MLKL | turquoise |
| MLLT10 | turquoise |
| MLLT3 | turquoise |
| MLLT4 | turquoise |
| MLX | turquoise |
| MLYCD | turquoise |
| MMAA | turquoise |
| MMAB | turquoise |
| MMD | turquoise |
| MMGT1 | turquoise |
| MMP15 | turquoise |
| MMP24-AS1 | turquoise |
| MMS22L | turquoise |
| MNAT1 | turquoise |
| MOB1A | turquoise |
| MOB1B | turquoise |
| MOB2 | turquoise |
| MOB3B | turquoise |
| MOK | turquoise |
| MON1A | turquoise |
| MON1B | turquoise |
| MON2 | turquoise |
| MORC3 | turquoise |
| MORC4 | turquoise |
| MOSPD2 | turquoise |
| MOSPD3 | turquoise |
| MPDU1 | turquoise |
| MPDZ | turquoise |
| MPG | turquoise |
| MPHOSPH6 | turquoise |
| MPHOSPH9 | turquoise |
| MPLKIP | turquoise |
| MPND | turquoise |
| MPP5 | turquoise |
| MPP6 | turquoise |
| MPP7 | turquoise |
| MPPE1 | turquoise |
| MPRIP | turquoise |
| MPST | turquoise |
| MPV17L2 | turquoise |
| MPZL3 | turquoise |
| MRE11A | turquoise |
| MRFAP1 | turquoise |
| MRPL10 | turquoise |
| MRPL20 | turquoise |
| MRPL23 | turquoise |
| MRPL4 | turquoise |
| MRPL42 | turquoise |
| MRPS18B | turquoise |
| MRPS18C | turquoise |
| MRPS2 | turquoise |
| MRPS30 | turquoise |
| MRPS31P4 | turquoise |
| MRRF | turquoise |
| MSANTD2 | turquoise |
| MSANTD4 | turquoise |
| MSH3 | turquoise |
| MSI2 | turquoise |
| MSL1 | turquoise |
| MSL2 | turquoise |
| MSL3 | turquoise |
| MSS51 | turquoise |
| MT-ATP6 | turquoise |
| MT-ATP8 | turquoise |
| MT-ND3 | turquoise |
| MT-ND6 | turquoise |
| MT-RNR1 | turquoise |
| MT-RNR2 | turquoise |
| MT-TF | turquoise |
| MT-TM | turquoise |
| MT-TP | turquoise |
| MT-TS2 | turquoise |
| MT-TT | turquoise |
| MT-TV | turquoise |
| MT1A | turquoise |
| MT1E | turquoise |
| MT1L | turquoise |
| MT1X | turquoise |
| MT1XP1 | turquoise |
| MT2A | turquoise |
| MT2P1 | turquoise |
| MTAP | turquoise |
| MTCH1 | turquoise |
| MTCL1 | turquoise |
| MTCP1 | turquoise |
| MTDH | turquoise |
| MTERF1 | turquoise |
| MTERF2 | turquoise |
| MTERF4 | turquoise |
| MTF1 | turquoise |
| MTF2 | turquoise |
| MTFMT | turquoise |
| MTHFD2L | turquoise |
| MTIF3 | turquoise |
| MTM1 | turquoise |
| MTMR1 | turquoise |
| MTMR10 | turquoise |
| MTMR12 | turquoise |
| MTMR2 | turquoise |
| MTMR3 | turquoise |
| MTMR4 | turquoise |
| MTMR6 | turquoise |
| MTMR7 | turquoise |
| MTMR9 | turquoise |
| MTND5P11 | turquoise |
| MTO1 | turquoise |
| MTOR | turquoise |
| MTPAP | turquoise |
| MTR | turquoise |
| MTRF1 | turquoise |
| MTRF1L | turquoise |
| MTRNR2L1 | turquoise |
| MTRNR2L12 | turquoise |
| MTRNR2L8 | turquoise |
| MTRR | turquoise |
| MTSS1 | turquoise |
| MTUS1 | turquoise |
| MTX3 | turquoise |
| MUC1 | turquoise |
| MUC16 | turquoise |
| MUL1 | turquoise |
| MUM1L1 | turquoise |
| MVB12A | turquoise |
| MVD | turquoise |
| MVK | turquoise |
| MVP | turquoise |
| MXD1 | turquoise |
| MXI1 | turquoise |
| MYBL1 | turquoise |
| MYBPH | turquoise |
| MYCBP2 | turquoise |
| MYDGF | turquoise |
| MYEF2 | turquoise |
| MYH14 | turquoise |
| MYL12A | turquoise |
| MYLIP | turquoise |
| MYLK-AS1 | turquoise |
| MYNN | turquoise |
| MYO10 | turquoise |
| MYO1B | turquoise |
| MYO5A | turquoise |
| MYO5B | turquoise |
| MYO5C | turquoise |
| MYO6 | turquoise |
| MYO9A | turquoise |
| MYOF | turquoise |
| MYRIP | turquoise |
| MYSM1 | turquoise |
| N4BP2 | turquoise |
| N4BP2L2 | turquoise |
| N4BP2L2-IT2 | turquoise |
| N6AMT1 | turquoise |
| NAA15 | turquoise |
| NAA16 | turquoise |
| NAA25 | turquoise |
| NAA30 | turquoise |
| NAA35 | turquoise |
| NAALADL2 | turquoise |
| NAALADL2-AS2 | turquoise |
| NAB1 | turquoise |
| NAB2 | turquoise |
| NABP1 | turquoise |
| NACA | turquoise |
| NACC1 | turquoise |
| NADK2 | turquoise |
| NAF1 | turquoise |
| NAGA | turquoise |
| NAGLU | turquoise |
| NAGPA | turquoise |
| NAGS | turquoise |
| NAIF1 | turquoise |
| NAMPT | turquoise |
| NANP | turquoise |
| NANS | turquoise |
| NAP1L1 | turquoise |
| NAP1L5 | turquoise |
| NAPA | turquoise |
| NAPB | turquoise |
| NAPEPLD | turquoise |
| NAPG | turquoise |
| NAPRT | turquoise |
| NARF-IT1 | turquoise |
| NAT6 | turquoise |
| NAV1 | turquoise |
| NAV2 | turquoise |
| NBAS | turquoise |
| NBEA | turquoise |
| NBEAL1 | turquoise |
| NBN | turquoise |
| NBPF1 | turquoise |
| NBPF11 | turquoise |
| NBPF12 | turquoise |
| NBPF14 | turquoise |
| NBPF15 | turquoise |
| NBPF2P | turquoise |
| NBPF3 | turquoise |
| NBPF8P | turquoise |
| NBPF9 | turquoise |
| NBR1 | turquoise |
| NCAM2 | turquoise |
| NCAPH2 | turquoise |
| NCBP1 | turquoise |
| NCCRP1 | turquoise |
| NCK1 | turquoise |
| NCK1-AS1 | turquoise |
| NCK2 | turquoise |
| NCKAP1 | turquoise |
| NCKIPSD | turquoise |
| NCLN | turquoise |
| NCMAP | turquoise |
| NCOA1 | turquoise |
| NCOA2 | turquoise |
| NCOA3 | turquoise |
| NCOA6 | turquoise |
| NCOA7 | turquoise |
| NCOR1 | turquoise |
| NDEL1 | turquoise |
| NDNL2 | turquoise |
| NDST2 | turquoise |
| NDUFA4L2 | turquoise |
| NDUFA5 | turquoise |
| NDUFAF1 | turquoise |
| NDUFAF3 | turquoise |
| NDUFAF5 | turquoise |
| NDUFAF6 | turquoise |
| NDUFAF7 | turquoise |
| NDUFS1 | turquoise |
| NDUFS7 | turquoise |
| NDUFV2 | turquoise |
| NDUFV2-AS1 | turquoise |
| NEAT1 | turquoise |
| NEDD1 | turquoise |
| NEDD4 | turquoise |
| NEIL2 | turquoise |
| NEK1 | turquoise |
| NEK3 | turquoise |
| NEK4 | turquoise |
| NEK6 | turquoise |
| NEK7 | turquoise |
| NEK9 | turquoise |
| NELFB | turquoise |
| NEMF | turquoise |
| NEO1 | turquoise |
| NEU3 | turquoise |
| NF1 | turquoise |
| NFAT5 | turquoise |
| NFATC2 | turquoise |
| NFATC3 | turquoise |
| NFE2 | turquoise |
| NFE2L1 | turquoise |
| NFE2L3 | turquoise |
| NFIA | turquoise |
| NFIB | turquoise |
| NFKB2 | turquoise |
| NFKBIB | turquoise |
| NFKBIE | turquoise |
| NFKBIL1 | turquoise |
| NFKBIZ | turquoise |
| NFRKB | turquoise |
| NFX1 | turquoise |
| NFXL1 | turquoise |
| NFYA | turquoise |
| NFYB | turquoise |
| NFYC-AS1 | turquoise |
| NGFRAP1 | turquoise |
| NGLY1 | turquoise |
| NGRN | turquoise |
| NHLRC1 | turquoise |
| NHLRC2 | turquoise |
| NHLRC3 | turquoise |
| NHP2L1 | turquoise |
| NHS | turquoise |
| NIFK-AS1 | turquoise |
| NIN | turquoise |
| NINJ1 | turquoise |
| NIPA1 | turquoise |
| NIPA2 | turquoise |
| NIPAL2 | turquoise |
| NIPBL | turquoise |
| NIPSNAP3B | turquoise |
| NKAP | turquoise |
| NKD1 | turquoise |
| NKIRAS1 | turquoise |
| NKRF | turquoise |
| NKTR | turquoise |
| NKX1-2 | turquoise |
| NKX3-1 | turquoise |
| NLK | turquoise |
| NLRP3 | turquoise |
| NLRX1 | turquoise |
| NMB | turquoise |
| NME6 | turquoise |
| NME7 | turquoise |
| NMNAT1 | turquoise |
| NMNAT2 | turquoise |
| NMRK1 | turquoise |
| NMT2 | turquoise |
| NNMT | turquoise |
| NNT | turquoise |
| NNT-AS1 | turquoise |
| NOC3L | turquoise |
| NOC4L | turquoise |
| NOD1 | turquoise |
| NOG | turquoise |
| NOL4L | turquoise |
| NOL8 | turquoise |
| NOL9 | turquoise |
| NOM1 | turquoise |
| NOMO2 | turquoise |
| NOMO3 | turquoise |
| NONOP2 | turquoise |
| NOP14-AS1 | turquoise |
| NOP56P3 | turquoise |
| NOS1AP | turquoise |
| NOS2 | turquoise |
| NOS3 | turquoise |
| NOTCH2 | turquoise |
| NOVA1-AS1 | turquoise |
| NOX1 | turquoise |
| NPAT | turquoise |
| NPC1 | turquoise |
| NPDC1 | turquoise |
| NPEPPS | turquoise |
| NPHP3 | turquoise |
| NPM1P25 | turquoise |
| NPM1P26 | turquoise |
| NPTN-IT1 | turquoise |
| NPTXR | turquoise |
| NPY1R | turquoise |
| NR1D1 | turquoise |
| NR1D2 | turquoise |
| NR1H2 | turquoise |
| NR2C1 | turquoise |
| NR2C2 | turquoise |
| NR2F1-AS1 | turquoise |
| NR3C1 | turquoise |
| NR3C2 | turquoise |
| NR6A1 | turquoise |
| NRBP1 | turquoise |
| NRDE2 | turquoise |
| NRF1 | turquoise |
| NRIP1 | turquoise |
| NRIP2 | turquoise |
| NRP1 | turquoise |
| NRP2 | turquoise |
| NRSN2 | turquoise |
| NRXN3 | turquoise |
| NSD1 | turquoise |
| NSDHL | turquoise |
| NSFL1C | turquoise |
| NSMAF | turquoise |
| NSMCE1 | turquoise |
| NSRP1 | turquoise |
| NSUN3 | turquoise |
| NSUN4 | turquoise |
| NSUN6 | turquoise |
| NSUN7 | turquoise |
| NT5C | turquoise |
| NT5C2 | turquoise |
| NT5C3A | turquoise |
| NT5DC2 | turquoise |
| NT5DC3 | turquoise |
| NTMT1 | turquoise |
| NTSR1 | turquoise |
| NUBP1 | turquoise |
| NUBPL | turquoise |
| NUCB1 | turquoise |
| NUCKS1 | turquoise |
| NUDC | turquoise |
| NUDT12 | turquoise |
| NUDT13 | turquoise |
| NUDT14 | turquoise |
| NUDT16 | turquoise |
| NUDT16L1 | turquoise |
| NUDT16P1 | turquoise |
| NUDT18 | turquoise |
| NUDT21 | turquoise |
| NUDT22 | turquoise |
| NUDT3 | turquoise |
| NUDT4 | turquoise |
| NUDT9 | turquoise |
| NUFIP2 | turquoise |
| NUMB | turquoise |
| NUP160 | turquoise |
| NUP214 | turquoise |
| NUP43 | turquoise |
| NUP50 | turquoise |
| NUP62CL | turquoise |
| NUP98 | turquoise |
| NUPL1 | turquoise |
| NUPL2 | turquoise |
| NUPR1 | turquoise |
| NUS1 | turquoise |
| NUTM2A-AS1 | turquoise |
| NUTM2B-AS1 | turquoise |
| NVL | turquoise |
| NXPE3 | turquoise |
| OAZ1 | turquoise |
| OAZ2 | turquoise |
| OBFC1 | turquoise |
| OCEL1 | turquoise |
| OCIAD1-AS1 | turquoise |
| OCLM | turquoise |
| OCLN | turquoise |
| OCRL | turquoise |
| ODF2L | turquoise |
| OFD1 | turquoise |
| OGDH | turquoise |
| OGFOD3 | turquoise |
| OGFRL1 | turquoise |
| OGG1 | turquoise |
| OGT | turquoise |
| OIP5-AS1 | turquoise |
| OLFM2 | turquoise |
| OLFM4 | turquoise |
| OLFML3 | turquoise |
| OMA1 | turquoise |
| OMG | turquoise |
| OPA3 | turquoise |
| OPHN1 | turquoise |
| OPN1SW | turquoise |
| OR2A1-AS1 | turquoise |
| OR2A7 | turquoise |
| OR7E38P | turquoise |
| OR7E7P | turquoise |
| ORAI1 | turquoise |
| ORAI3 | turquoise |
| ORC2 | turquoise |
| ORC3 | turquoise |
| ORC4 | turquoise |
| ORM1 | turquoise |
| ORM2 | turquoise |
| ORMDL1 | turquoise |
| ORMDL3 | turquoise |
| OS9 | turquoise |
| OSBP | turquoise |
| OSBPL10 | turquoise |
| OSBPL11 | turquoise |
| OSBPL1A | turquoise |
| OSBPL3 | turquoise |
| OSBPL5 | turquoise |
| OSBPL8 | turquoise |
| OSBPL9 | turquoise |
| OSGEPL1 | turquoise |
| OSGIN2 | turquoise |
| OSMR | turquoise |
| OSMR-AS1 | turquoise |
| OTUB1 | turquoise |
| OTUD3 | turquoise |
| OTUD4 | turquoise |
| OTUD6B | turquoise |
| OTUD6B-AS1 | turquoise |
| OTUD7B | turquoise |
| OTULIN | turquoise |
| OXA1L | turquoise |
| OXNAD1 | turquoise |
| OXR1 | turquoise |
| OXSR1 | turquoise |
| P2RX4 | turquoise |
| P2RY2 | turquoise |
| P4HA2 | turquoise |
| PABPC1P3 | turquoise |
| PABPC1P4 | turquoise |
| PACERR | turquoise |
| PACRGL | turquoise |
| PACSIN2 | turquoise |
| PADI1 | turquoise |
| PAF1 | turquoise |
| PAFAH1B1 | turquoise |
| PAFAH1B2 | turquoise |
| PAFAH2 | turquoise |
| PAG1 | turquoise |
| PAGE2 | turquoise |
| PAIP2B | turquoise |
| PAK2 | turquoise |
| PAK4 | turquoise |
| PALB2 | turquoise |
| PAM | turquoise |
| PAN3 | turquoise |
| PAN3-AS1 | turquoise |
| PANK1 | turquoise |
| PANK2 | turquoise |
| PANK3 | turquoise |
| PANX1 | turquoise |
| PAPD4 | turquoise |
| PAPD5 | turquoise |
| PAPD7 | turquoise |
| PAPOLA | turquoise |
| PAPOLG | turquoise |
| PAPPA | turquoise |
| PAQR3 | turquoise |
| PAQR7 | turquoise |
| PARD3 | turquoise |
| PARD6A | turquoise |
| PARD6B | turquoise |
| PARD6G | turquoise |
| PARG | turquoise |
| PARP11 | turquoise |
| PARP14 | turquoise |
| PARP15 | turquoise |
| PARP3 | turquoise |
| PARP4 | turquoise |
| PARP6 | turquoise |
| PARP8 | turquoise |
| PARP9 | turquoise |
| PARPG1 | turquoise |
| PATL2 | turquoise |
| PAWR | turquoise |
| PAX8-AS1 | turquoise |
| PAXBP1 | turquoise |
| PAXBP1-AS1 | turquoise |
| PAXIP1 | turquoise |
| PAXIP1-AS2 | turquoise |
| PBDC1 | turquoise |
| PBRM1 | turquoise |
| PBX1 | turquoise |
| PBX3 | turquoise |
| PBXIP1 | turquoise |
| PCBD1 | turquoise |
| PCBD2 | turquoise |
| PCBP1 | turquoise |
| PCBP1-AS1 | turquoise |
| PCBP4 | turquoise |
| PCCA | turquoise |
| PCDH18 | turquoise |
| PCDH7 | turquoise |
| PCDHAC1 | turquoise |
| PCDHAC2 | turquoise |
| PCDHB10 | turquoise |
| PCDHB11 | turquoise |
| PCDHB12 | turquoise |
| PCDHB13 | turquoise |
| PCDHB14 | turquoise |
| PCDHB15 | turquoise |
| PCDHB16 | turquoise |
| PCDHB4 | turquoise |
| PCDHB7 | turquoise |
| PCDHB9 | turquoise |
| PCDHGA10 | turquoise |
| PCDHGB2 | turquoise |
| PCDHGB5 | turquoise |
| PCF11 | turquoise |
| PCGF2 | turquoise |
| PCGF3 | turquoise |
| PCGF5 | turquoise |
| PCIF1 | turquoise |
| PCK2 | turquoise |
| PCM1 | turquoise |
| PCMTD1 | turquoise |
| PCMTD2 | turquoise |
| PCNT | turquoise |
| PCNX | turquoise |
| PCNXL2 | turquoise |
| PCNXL4 | turquoise |
| PCSK5 | turquoise |
| PCSK7 | turquoise |
| PCSK9 | turquoise |
| PCYT1A | turquoise |
| PCYT2 | turquoise |
| PDAP1 | turquoise |
| PDCD4 | turquoise |
| PDCD4-AS1 | turquoise |
| PDCD6IP | turquoise |
| PDCD7 | turquoise |
| PDCL | turquoise |
| PDE12 | turquoise |
| PDE1A | turquoise |
| PDE3A | turquoise |
| PDE3B | turquoise |
| PDE4B | turquoise |
| PDE4D | turquoise |
| PDE4DIP | turquoise |
| PDE5A | turquoise |
| PDE7A | turquoise |
| PDE7B | turquoise |
| PDE8A | turquoise |
| PDE8B | turquoise |
| PDGFC | turquoise |
| PDGFD | turquoise |
| PDGFRA | turquoise |
| PDHB | turquoise |
| PDIK1L | turquoise |
| PDK1 | turquoise |
| PDK2 | turquoise |
| PDK3 | turquoise |
| PDLIM3 | turquoise |
| PDLIM4 | turquoise |
| PDLIM5 | turquoise |
| PDP1 | turquoise |
| PDP2 | turquoise |
| PDPK1 | turquoise |
| PDPR | turquoise |
| PDS5A | turquoise |
| PDS5B | turquoise |
| PDSS2 | turquoise |
| PDXDC2P | turquoise |
| PDZD8 | turquoise |
| PDZK1IP1 | turquoise |
| PEBP1 | turquoise |
| PECR | turquoise |
| PEF1 | turquoise |
| PELI1 | turquoise |
| PELI2 | turquoise |
| PELI3 | turquoise |
| PELO | turquoise |
| PELP1 | turquoise |
| PEPD | turquoise |
| PER2 | turquoise |
| PER3 | turquoise |
| PEX1 | turquoise |
| PEX10 | turquoise |
| PEX11G | turquoise |
| PEX12 | turquoise |
| PEX13 | turquoise |
| PEX14 | turquoise |
| PEX16 | turquoise |
| PEX26 | turquoise |
| PEX5 | turquoise |
| PFDN1 | turquoise |
| PFKFB2 | turquoise |
| PFKFB3 | turquoise |
| PFKL | turquoise |
| PFN1 | turquoise |
| PFN1P7 | turquoise |
| PFN4 | turquoise |
| PGAP1 | turquoise |
| PGBD4 | turquoise |
| PGD | turquoise |
| PGF | turquoise |
| PGGT1B | turquoise |
| PGLS | turquoise |
| PGM1 | turquoise |
| PGM2L1 | turquoise |
| PGM3 | turquoise |
| PGRMC2 | turquoise |
| PHACTR2 | turquoise |
| PHACTR4 | turquoise |
| PHAX | turquoise |
| PHBP9 | turquoise |
| PHC2 | turquoise |
| PHC3 | turquoise |
| PHF10 | turquoise |
| PHF11 | turquoise |
| PHF14 | turquoise |
| PHF20 | turquoise |
| PHF20L1 | turquoise |
| PHF21A | turquoise |
| PHF23 | turquoise |
| PHF3 | turquoise |
| PHF6 | turquoise |
| PHIP | turquoise |
| PHKB | turquoise |
| PHKG1 | turquoise |
| PHLDA1 | turquoise |
| PHLDA3 | turquoise |
| PHLDB1 | turquoise |
| PHLDB2 | turquoise |
| PHLPP1 | turquoise |
| PHLPP2 | turquoise |
| PHOSPHO2 | turquoise |
| PHTF1 | turquoise |
| PHTF2 | turquoise |
| PHYKPL | turquoise |
| PI4K2B | turquoise |
| PI4KA | turquoise |
| PIAS1 | turquoise |
| PIAS2 | turquoise |
| PIBF1 | turquoise |
| PICALM | turquoise |
| PIEZO2 | turquoise |
| PIGA | turquoise |
| PIGB | turquoise |
| PIGCP1 | turquoise |
| PIGF | turquoise |
| PIGG | turquoise |
| PIGK | turquoise |
| PIGL | turquoise |
| PIGM | turquoise |
| PIGN | turquoise |
| PIGO | turquoise |
| PIGR | turquoise |
| PIGS | turquoise |
| PIGT | turquoise |
| PIGV | turquoise |
| PIH1D1 | turquoise |
| PIK3C2A | turquoise |
| PIK3C2G | turquoise |
| PIK3C3 | turquoise |
| PIK3CA | turquoise |
| PIK3CB | turquoise |
| PIK3CG | turquoise |
| PIK3R1 | turquoise |
| PIK3R3 | turquoise |
| PIK3R4 | turquoise |
| PIKFYVE | turquoise |
| PIM1 | turquoise |
| PIM3 | turquoise |
| PIN1 | turquoise |
| PINK1 | turquoise |
| PINK1-AS | turquoise |
| PIP4K2A | turquoise |
| PIP5K1A | turquoise |
| PIP5K1C | turquoise |
| PITHD1 | turquoise |
| PITPNB | turquoise |
| PITPNM1 | turquoise |
| PIWIL4 | turquoise |
| PJA1 | turquoise |
| PJA2 | turquoise |
| pk | turquoise |
| PKD2 | turquoise |
| PKM | turquoise |
| PKN1 | turquoise |
| PKN2 | turquoise |
| PKN3 | turquoise |
| PKNOX1 | turquoise |
| PKP4 | turquoise |
| PLA2G12A | turquoise |
| PLA2G16 | turquoise |
| PLA2R1 | turquoise |
| PLAC4 | turquoise |
| PLAG1 | turquoise |
| PLAGL1 | turquoise |
| PLAT | turquoise |
| PLAUR | turquoise |
| PLB1 | turquoise |
| PLBD2 | turquoise |
| PLCB1 | turquoise |
| PLCB4 | turquoise |
| PLCD1 | turquoise |
| PLCD4 | turquoise |
| PLCH1 | turquoise |
| PLCL2 | turquoise |
| PLCXD3 | turquoise |
| PLD1 | turquoise |
| PLD3 | turquoise |
| PLEKHA1 | turquoise |
| PLEKHA2 | turquoise |
| PLEKHA3 | turquoise |
| PLEKHA4 | turquoise |
| PLEKHA5 | turquoise |
| PLEKHA7 | turquoise |
| PLEKHA8 | turquoise |
| PLEKHB1 | turquoise |
| PLEKHF1 | turquoise |
| PLEKHF2 | turquoise |
| PLEKHG1 | turquoise |
| PLEKHG7 | turquoise |
| PLEKHH1 | turquoise |
| PLEKHH2 | turquoise |
| PLEKHM1P | turquoise |
| PLEKHM2 | turquoise |
| PLEKHM3 | turquoise |
| PLIN3 | turquoise |
| PLK2 | turquoise |
| PLN | turquoise |
| PLOD1 | turquoise |
| PLOD3 | turquoise |
| PLP2 | turquoise |
| PLS3 | turquoise |
| PLSCR1 | turquoise |
| PLTP | turquoise |
| PLVAP | turquoise |
| PLXDC1 | turquoise |
| PLXDC2 | turquoise |
| PLXNB2 | turquoise |
| PLXNC1 | turquoise |
| PM20D2 | turquoise |
| PMF1-BGLAP | turquoise |
| PMM1 | turquoise |
| PMM2 | turquoise |
| PMPCA | turquoise |
| PMS1 | turquoise |
| PMS2 | turquoise |
| PMS2CL | turquoise |
| PMS2P3 | turquoise |
| PMS2P4 | turquoise |
| PMVK | turquoise |
| PNISR | turquoise |
| PNKD | turquoise |
| PNMAL1 | turquoise |
| PNPLA2 | turquoise |
| PNPLA4 | turquoise |
| PNPLA8 | turquoise |
| PNRC1 | turquoise |
| PNRC2 | turquoise |
| POC1B | turquoise |
| POC5 | turquoise |
| PODNL1 | turquoise |
| POGK | turquoise |
| POGLUT1 | turquoise |
| POGZ | turquoise |
| POLA1 | turquoise |
| POLD4 | turquoise |
| POLDIP3 | turquoise |
| POLH | turquoise |
| POLI | turquoise |
| POLK | turquoise |
| POLR2A | turquoise |
| POLR2C | turquoise |
| POLR2E | turquoise |
| POLR2I | turquoise |
| POLR2L | turquoise |
| POLR2M | turquoise |
| POLR3A | turquoise |
| POLR3B | turquoise |
| POLR3E | turquoise |
| POLR3F | turquoise |
| POLR3GL | turquoise |
| POMGNT1 | turquoise |
| POMGNT2 | turquoise |
| POMK | turquoise |
| POMZP3 | turquoise |
| PON1 | turquoise |
| POR | turquoise |
| PORCN | turquoise |
| POT1 | turquoise |
| POU2F1 | turquoise |
| POU3F2 | turquoise |
| POU6F2 | turquoise |
| POU6F2-AS2 | turquoise |
| PPAP2A | turquoise |
| PPAPDC2 | turquoise |
| PPARA | turquoise |
| PPARD | turquoise |
| PPARGC1B | turquoise |
| PPDPF | turquoise |
| PPFIA1 | turquoise |
| PPFIBP1 | turquoise |
| PPHLN1 | turquoise |
| PPIB | turquoise |
| PPIEL | turquoise |
| PPIG | turquoise |
| PPIL4 | turquoise |
| PPIP5K2 | turquoise |
| PPM1A | turquoise |
| PPM1B | turquoise |
| PPM1K | turquoise |
| PPM1L | turquoise |
| PPP1CB | turquoise |
| PPP1R11 | turquoise |
| PPP1R12A | turquoise |
| PPP1R12B | turquoise |
| PPP1R15B | turquoise |
| PPP1R1B | turquoise |
| PPP1R1C | turquoise |
| PPP1R2 | turquoise |
| PPP1R21 | turquoise |
| PPP1R3D | turquoise |
| PPP1R7 | turquoise |
| PPP1R9A | turquoise |
| PPP1R9B | turquoise |
| PPP2CA | turquoise |
| PPP2R1A | turquoise |
| PPP2R1B | turquoise |
| PPP2R2A | turquoise |
| PPP2R2D | turquoise |
| PPP2R3A | turquoise |
| PPP2R3C | turquoise |
| PPP2R4 | turquoise |
| PPP2R5B | turquoise |
| PPP2R5C | turquoise |
| PPP2R5E | turquoise |
| PPP3CA | turquoise |
| PPP3CB | turquoise |
| PPP3CB-AS1 | turquoise |
| PPP3CC | turquoise |
| PPP3R1 | turquoise |
| PPP4R1 | turquoise |
| PPP4R1-AS1 | turquoise |
| PPP4R1L | turquoise |
| PPP4R2 | turquoise |
| PPP5C | turquoise |
| PPP6C | turquoise |
| PPP6R1 | turquoise |
| PPP6R3 | turquoise |
| PPTC7 | turquoise |
| PPWD1 | turquoise |
| PQBP1 | turquoise |
| PQLC1 | turquoise |
| PQLC2 | turquoise |
| PRAF2 | turquoise |
| PRDM1 | turquoise |
| PRDM10 | turquoise |
| PRDM15 | turquoise |
| PRDM2 | turquoise |
| PRDM4 | turquoise |
| PRDM5 | turquoise |
| PRDM6 | turquoise |
| PRDM8 | turquoise |
| PRDX1 | turquoise |
| PRDX3P1 | turquoise |
| PRDX5 | turquoise |
| PRDX6 | turquoise |
| PRELID2 | turquoise |
| PREP | turquoise |
| PREPL | turquoise |
| PREX2 | turquoise |
| PRIMPOL | turquoise |
| PRINS | turquoise |
| PRKAA1 | turquoise |
| PRKAA2 | turquoise |
| PRKAB2 | turquoise |
| PRKACA | turquoise |
| PRKACB | turquoise |
| PRKAG2 | turquoise |
| PRKAR1A | turquoise |
| PRKAR1B | turquoise |
| PRKAR2A | turquoise |
| PRKAR2B | turquoise |
| PRKCA | turquoise |
| PRKCD | turquoise |
| PRKCDBP | turquoise |
| PRKCI | turquoise |
| PRKCQ-AS1 | turquoise |
| PRKCSH | turquoise |
| PRKD1 | turquoise |
| PRKD3 | turquoise |
| PRKG1 | turquoise |
| PRKRIR | turquoise |
| PRKX | turquoise |
| PRKY | turquoise |
| PRLR | turquoise |
| PRMT3 | turquoise |
| PRMT9 | turquoise |
| PROCR | turquoise |
| PRODH | turquoise |
| PRORSD1P | turquoise |
| PROX1 | turquoise |
| PRPF18 | turquoise |
| PRPF31 | turquoise |
| PRPF38A | turquoise |
| PRPF38B | turquoise |
| PRPF39 | turquoise |
| PRPF4B | turquoise |
| PRPF6 | turquoise |
| PRPS1P2 | turquoise |
| PRR13 | turquoise |
| PRR13P5 | turquoise |
| PRR14L | turquoise |
| PRR15L | turquoise |
| PRR34-AS1 | turquoise |
| PRR5L | turquoise |
| PRRC1 | turquoise |
| PRRC2B | turquoise |
| PRRC2C | turquoise |
| PRRG1 | turquoise |
| PRRG2 | turquoise |
| PRRG4 | turquoise |
| PRRT2 | turquoise |
| PRRX2 | turquoise |
| PRSS21 | turquoise |
| PRSS8 | turquoise |
| PRUNE2 | turquoise |
| PSD3 | turquoise |
| PSEN1 | turquoise |
| PSEN2 | turquoise |
| PSENEN | turquoise |
| PSIP1 | turquoise |
| PSKH1 | turquoise |
| PSMA3-AS1 | turquoise |
| PSMA6P1 | turquoise |
| PSMB7 | turquoise |
| PSMB8 | turquoise |
| PSMC3 | turquoise |
| PSMC4 | turquoise |
| PSMD10P1 | turquoise |
| PSMD5 | turquoise |
| PSMD5-AS1 | turquoise |
| PSMD6 | turquoise |
| PSME2P2 | turquoise |
| PSME4 | turquoise |
| PSMF1 | turquoise |
| PSMG4 | turquoise |
| PSORS1C3 | turquoise |
| PSPC1 | turquoise |
| PSTK | turquoise |
| PSTPIP2 | turquoise |
| PTAR1 | turquoise |
| PTBP2 | turquoise |
| PTBP3 | turquoise |
| PTCD2 | turquoise |
| PTCD3 | turquoise |
| PTCH1 | turquoise |
| PTCSC3 | turquoise |
| PTDSS2 | turquoise |
| PTEN | turquoise |
| PTER | turquoise |
| PTGER1 | turquoise |
| PTGER2 | turquoise |
| PTGES3P2 | turquoise |
| PTGFR | turquoise |
| PTGR2 | turquoise |
| PTK2 | turquoise |
| PTMS | turquoise |
| PTP4A1 | turquoise |
| PTP4A2 | turquoise |
| PTP4A2P1 | turquoise |
| PTPDC1 | turquoise |
| PTPLB | turquoise |
| PTPN11 | turquoise |
| PTPN12 | turquoise |
| PTPN13 | turquoise |
| PTPN14 | turquoise |
| PTPN2 | turquoise |
| PTPN22 | turquoise |
| PTPN23 | turquoise |
| PTPN3 | turquoise |
| PTPN4 | turquoise |
| PTPRA | turquoise |
| PTPRD | turquoise |
| PTPRE | turquoise |
| PTPRG | turquoise |
| PTPRJ | turquoise |
| PTPRK | turquoise |
| PTPRS | turquoise |
| PTPRZ1 | turquoise |
| PTTG1IP | turquoise |
| PUF60 | turquoise |
| PUM1 | turquoise |
| PUM2 | turquoise |
| PURA | turquoise |
| PURB | turquoise |
| PUS10 | turquoise |
| PUS7L | turquoise |
| PVRL2 | turquoise |
| PVRL3 | turquoise |
| PVT1 | turquoise |
| PWAR5 | turquoise |
| PWAR6 | turquoise |
| PWWP2A | turquoise |
| PWWP2B | turquoise |
| PXK | turquoise |
| PXN | turquoise |
| PXYLP1 | turquoise |
| PYCARD | turquoise |
| PYGB | turquoise |
| PYGO1 | turquoise |
| PYROXD1 | turquoise |
| PYURF | turquoise |
| QARS | turquoise |
| QKI | turquoise |
| QPCTL | turquoise |
| QRICH1 | turquoise |
| QRSL1 | turquoise |
| QSER1 | turquoise |
| QSOX1 | turquoise |
| QTRTD1 | turquoise |
| R3HCC1 | turquoise |
| R3HCC1L | turquoise |
| R3HDM2 | turquoise |
| R3HDM4 | turquoise |
| RAB11B | turquoise |
| RAB11B-AS1 | turquoise |
| RAB11FIP1P1 | turquoise |
| RAB11FIP2 | turquoise |
| RAB11FIP5 | turquoise |
| RAB12 | turquoise |
| RAB13 | turquoise |
| RAB14 | turquoise |
| RAB18 | turquoise |
| RAB1B | turquoise |
| RAB20 | turquoise |
| RAB21 | turquoise |
| RAB22A | turquoise |
| RAB23 | turquoise |
| RAB27A | turquoise |
| RAB27B | turquoise |
| RAB28 | turquoise |
| RAB29 | turquoise |
| RAB2B | turquoise |
| RAB30 | turquoise |
| RAB30-AS1 | turquoise |
| RAB33B | turquoise |
| RAB34 | turquoise |
| RAB35 | turquoise |
| RAB3A | turquoise |
| RAB3GAP1 | turquoise |
| RAB3GAP2 | turquoise |
| RAB3IL1 | turquoise |
| RAB43 | turquoise |
| RAB43P1 | turquoise |
| RAB4B | turquoise |
| RAB5A | turquoise |
| RAB5B | turquoise |
| RAB5C | turquoise |
| RAB7A | turquoise |
| RAB7B | turquoise |
| RAB8B | turquoise |
| RABAC1 | turquoise |
| RABEP1 | turquoise |
| RABGAP1 | turquoise |
| RABGAP1L | turquoise |
| RABGEF1 | turquoise |
| RABGGTA | turquoise |
| RABGGTB | turquoise |
| RABL3 | turquoise |
| RAD1 | turquoise |
| RAD17 | turquoise |
| RAD18 | turquoise |
| RAD23A | turquoise |
| RAD50 | turquoise |
| RAD51B | turquoise |
| RAD51D | turquoise |
| RAD52 | turquoise |
| RAD54L2 | turquoise |
| RAF1 | turquoise |
| RAI14 | turquoise |
| RALBP1 | turquoise |
| RALGAPA1 | turquoise |
| RALGAPA2 | turquoise |
| RALGAPB | turquoise |
| RALGPS1 | turquoise |
| RALGPS2 | turquoise |
| RALY | turquoise |
| RAMP1 | turquoise |
| RANBP17 | turquoise |
| RANBP2 | turquoise |
| RANBP6 | turquoise |
| RANBP9 | turquoise |
| RANGRF | turquoise |
| RANP4 | turquoise |
| RAP1AP | turquoise |
| RAP1B | turquoise |
| RAP1GDS1 | turquoise |
| RAP2A | turquoise |
| RAP2B | turquoise |
| RAP2C | turquoise |
| RAP2C-AS1 | turquoise |
| RAPGEF2 | turquoise |
| RAPGEF5 | turquoise |
| RAPGEF6 | turquoise |
| RAPH1 | turquoise |
| RARA | turquoise |
| RARB | turquoise |
| RARG | turquoise |
| RASA1 | turquoise |
| RASA2 | turquoise |
| RASAL2 | turquoise |
| RASEF | turquoise |
| RASGEF1B | turquoise |
| RASGRF2 | turquoise |
| RASGRP1 | turquoise |
| RASGRP3 | turquoise |
| RASL11A | turquoise |
| RASSF8 | turquoise |
| RASSF8-AS1 | turquoise |
| RASSF9 | turquoise |
| RAVER1 | turquoise |
| RAVER2 | turquoise |
| RB1 | turquoise |
| RB1CC1 | turquoise |
| RBAK | turquoise |
| RBBP6 | turquoise |
| RBBP9 | turquoise |
| RBCK1 | turquoise |
| RBFOX2 | turquoise |
| RBL2 | turquoise |
| RBM11 | turquoise |
| RBM12 | turquoise |
| RBM12B | turquoise |
| RBM12B-AS1 | turquoise |
| RBM14 | turquoise |
| RBM14-RBM4 | turquoise |
| RBM15 | turquoise |
| RBM18 | turquoise |
| RBM22 | turquoise |
| RBM23 | turquoise |
| RBM25 | turquoise |
| RBM26 | turquoise |
| RBM27 | turquoise |
| RBM28 | turquoise |
| RBM33 | turquoise |
| RBM34 | turquoise |
| RBM39 | turquoise |
| RBM4 | turquoise |
| RBM41 | turquoise |
| RBM42 | turquoise |
| RBM43 | turquoise |
| RBM45 | turquoise |
| RBM47 | turquoise |
| RBM48 | turquoise |
| RBM4B | turquoise |
| RBM7 | turquoise |
| RBMS1 | turquoise |
| RBMS1P1 | turquoise |
| RBMS3 | turquoise |
| RBMS3-AS3 | turquoise |
| RBMX | turquoise |
| RBMXL1 | turquoise |
| RBP7 | turquoise |
| RBPJ | turquoise |
| RBPMS-AS1 | turquoise |
| RBSN | turquoise |
| RC3H1 | turquoise |
| RC3H2 | turquoise |
| RCAN3 | turquoise |
| RCBTB1 | turquoise |
| RCBTB2 | turquoise |
| RCHY1 | turquoise |
| RCL1 | turquoise |
| RCOR1 | turquoise |
| RCOR3 | turquoise |
| RDX | turquoise |
| REEP3 | turquoise |
| REEP4 | turquoise |
| REEP6 | turquoise |
| REL | turquoise |
| RELA | turquoise |
| RELB | turquoise |
| RELL1 | turquoise |
| REPS1 | turquoise |
| REPS2 | turquoise |
| RERE | turquoise |
| REST | turquoise |
| RETSAT | turquoise |
| REV1 | turquoise |
| REV3L | turquoise |
| REXO4 | turquoise |
| RFC1 | turquoise |
| RFFL | turquoise |
| RFTN2 | turquoise |
| RFWD2 | turquoise |
| RFX1 | turquoise |
| RFX3 | turquoise |
| RFX3-AS1 | turquoise |
| RFX5 | turquoise |
| RFX7 | turquoise |
| RFXAP | turquoise |
| RGL1 | turquoise |
| RGMB | turquoise |
| RGP1 | turquoise |
| RGS1 | turquoise |
| RGS14 | turquoise |
| RGS17 | turquoise |
| RGS20 | turquoise |
| RGS3 | turquoise |
| RHBDD1 | turquoise |
| RHBDD2 | turquoise |
| RHOA | turquoise |
| RHOA-IT1 | turquoise |
| RHOBTB1 | turquoise |
| RHOBTB3 | turquoise |
| RHOC | turquoise |
| RHOD | turquoise |
| RHOF | turquoise |
| RHOG | turquoise |
| RHOQ | turquoise |
| RHOT1 | turquoise |
| RHOU | turquoise |
| RIC1 | turquoise |
| RIC3 | turquoise |
| RIC8A | turquoise |
| RIC8B | turquoise |
| RICTOR | turquoise |
| RIF1 | turquoise |
| RILP | turquoise |
| RIMKLB | turquoise |
| RIMKLBP2 | turquoise |
| RIMS3 | turquoise |
| RIN1 | turquoise |
| RINT1 | turquoise |
| RIOK2 | turquoise |
| RIOK3 | turquoise |
| RIPK1 | turquoise |
| RIPK2 | turquoise |
| RIT1 | turquoise |
| RLF | turquoise |
| RLIM | turquoise |
| RMDN1 | turquoise |
| RMND1 | turquoise |
| RMND5A | turquoise |
| RN7SKP16 | turquoise |
| RN7SKP173 | turquoise |
| RN7SKP203 | turquoise |
| RN7SKP227 | turquoise |
| RN7SKP239 | turquoise |
| RN7SKP287 | turquoise |
| RN7SKP51 | turquoise |
| RN7SKP70 | turquoise |
| RN7SKP71 | turquoise |
| RN7SKP9 | turquoise |
| RN7SKP97 | turquoise |
| RN7SL116P | turquoise |
| RN7SL130P | turquoise |
| RN7SL138P | turquoise |
| RN7SL141P | turquoise |
| RN7SL145P | turquoise |
| RN7SL151P | turquoise |
| RN7SL181P | turquoise |
| RN7SL329P | turquoise |
| RN7SL333P | turquoise |
| RN7SL368P | turquoise |
| RN7SL381P | turquoise |
| RN7SL431P | turquoise |
| RN7SL434P | turquoise |
| RN7SL481P | turquoise |
| RN7SL546P | turquoise |
| RN7SL558P | turquoise |
| RN7SL564P | turquoise |
| RN7SL566P | turquoise |
| RN7SL5P | turquoise |
| RN7SL608P | turquoise |
| RN7SL648P | turquoise |
| RN7SL665P | turquoise |
| RN7SL674P | turquoise |
| RN7SL689P | turquoise |
| RN7SL767P | turquoise |
| RN7SL809P | turquoise |
| RN7SL81P | turquoise |
| RN7SL834P | turquoise |
| RN7SL838P | turquoise |
| RN7SL8P | turquoise |
| RNA5SP118 | turquoise |
| RNA5SP187 | turquoise |
| RNA5SP202 | turquoise |
| RNA5SP217 | turquoise |
| RNA5SP219 | turquoise |
| RNA5SP282 | turquoise |
| RNA5SP311 | turquoise |
| RNA5SP317 | turquoise |
| RNA5SP37 | turquoise |
| RNA5SP82 | turquoise |
| RNASEH2B | turquoise |
| RNASEK | turquoise |
| RNASEL | turquoise |
| RND1 | turquoise |
| RND3 | turquoise |
| RNF10 | turquoise |
| RNF103 | turquoise |
| RNF11 | turquoise |
| RNF111 | turquoise |
| RNF113A | turquoise |
| RNF115 | turquoise |
| RNF125 | turquoise |
| RNF126 | turquoise |
| RNF135 | turquoise |
| RNF138 | turquoise |
| RNF141 | turquoise |
| RNF144A | turquoise |
| RNF146 | turquoise |
| RNF149 | turquoise |
| RNF150 | turquoise |
| RNF152 | turquoise |
| RNF167 | turquoise |
| RNF168 | turquoise |
| RNF169 | turquoise |
| RNF170 | turquoise |
| RNF175 | turquoise |
| RNF180 | turquoise |
| RNF185 | turquoise |
| RNF187 | turquoise |
| RNF19A | turquoise |
| RNF19B | turquoise |
| RNF2 | turquoise |
| RNF212 | turquoise |
| RNF213 | turquoise |
| RNF214 | turquoise |
| RNF216P1 | turquoise |
| RNF217 | turquoise |
| RNF219 | turquoise |
| RNF223 | turquoise |
| RNF24 | turquoise |
| RNF25 | turquoise |
| RNF32 | turquoise |
| RNF38 | turquoise |
| RNF39 | turquoise |
| RNF5 | turquoise |
| RNF6 | turquoise |
| RNFT1 | turquoise |
| RNGTT | turquoise |
| RNH1 | turquoise |
| RNLS | turquoise |
| RNMT | turquoise |
| RNPC3 | turquoise |
| RNPEP | turquoise |
| RNPEPL1 | turquoise |
| RNU1-103P | turquoise |
| RNU1-106P | turquoise |
| RNU1-11P | turquoise |
| RNU1-13P | turquoise |
| RNU1-67P | turquoise |
| RNU1-88P | turquoise |
| RNU2-23P | turquoise |
| RNU2-27P | turquoise |
| RNU2-59P | turquoise |
| RNU4-1 | turquoise |
| RNU4-2 | turquoise |
| RNU4-24P | turquoise |
| RNU4-47P | turquoise |
| RNU4-51P | turquoise |
| RNU4-62P | turquoise |
| RNU4-78P | turquoise |
| RNU4ATAC | turquoise |
| RNU4ATAC12P | turquoise |
| RNU4ATAC16P | turquoise |
| RNU4ATAC18P | turquoise |
| RNU5A-1 | turquoise |
| RNU5A-8P | turquoise |
| RNU5B-1 | turquoise |
| RNU5D-1 | turquoise |
| RNU5E-1 | turquoise |
| RNU5F-1 | turquoise |
| RNU6-1005P | turquoise |
| RNU6-100P | turquoise |
| RNU6-1010P | turquoise |
| RNU6-1011P | turquoise |
| RNU6-1016P | turquoise |
| RNU6-1045P | turquoise |
| RNU6-1048P | turquoise |
| RNU6-1053P | turquoise |
| RNU6-107P | turquoise |
| RNU6-1095P | turquoise |
| RNU6-1099P | turquoise |
| RNU6-1136P | turquoise |
| RNU6-1157P | turquoise |
| RNU6-1161P | turquoise |
| RNU6-1165P | turquoise |
| RNU6-1176P | turquoise |
| RNU6-1189P | turquoise |
| RNU6-118P | turquoise |
| RNU6-1209P | turquoise |
| RNU6-1223P | turquoise |
| RNU6-122P | turquoise |
| RNU6-1262P | turquoise |
| RNU6-126P | turquoise |
| RNU6-1280P | turquoise |
| RNU6-1283P | turquoise |
| RNU6-130P | turquoise |
| RNU6-137P | turquoise |
| RNU6-195P | turquoise |
| RNU6-2 | turquoise |
| RNU6-216P | turquoise |
| RNU6-247P | turquoise |
| RNU6-251P | turquoise |
| RNU6-26P | turquoise |
| RNU6-282P | turquoise |
| RNU6-312P | turquoise |
| RNU6-322P | turquoise |
| RNU6-341P | turquoise |
| RNU6-343P | turquoise |
| RNU6-37P | turquoise |
| RNU6-387P | turquoise |
| RNU6-403P | turquoise |
| RNU6-407P | turquoise |
| RNU6-415P | turquoise |
| RNU6-418P | turquoise |
| RNU6-431P | turquoise |
| RNU6-476P | turquoise |
| RNU6-481P | turquoise |
| RNU6-510P | turquoise |
| RNU6-522P | turquoise |
| RNU6-531P | turquoise |
| RNU6-548P | turquoise |
| RNU6-574P | turquoise |
| RNU6-595P | turquoise |
| RNU6-608P | turquoise |
| RNU6-610P | turquoise |
| RNU6-611P | turquoise |
| RNU6-638P | turquoise |
| RNU6-652P | turquoise |
| RNU6-658P | turquoise |
| RNU6-681P | turquoise |
| RNU6-759P | turquoise |
| RNU6-761P | turquoise |
| RNU6-762P | turquoise |
| RNU6-767P | turquoise |
| RNU6-795P | turquoise |
| RNU6-807P | turquoise |
| RNU6-82P | turquoise |
| RNU6-850P | turquoise |
| RNU6-853P | turquoise |
| RNU6-856P | turquoise |
| RNU6-882P | turquoise |
| RNU6-883P | turquoise |
| RNU6-890P | turquoise |
| RNU6-915P | turquoise |
| RNU6-920P | turquoise |
| RNU6-925P | turquoise |
| RNU6-930P | turquoise |
| RNU6-942P | turquoise |
| RNU6-96P | turquoise |
| RNU6-998P | turquoise |
| RNU6ATAC | turquoise |
| RNU6ATAC18P | turquoise |
| RNU6ATAC24P | turquoise |
| RNU6ATAC39P | turquoise |
| RNU7-123P | turquoise |
| RNU7-124P | turquoise |
| RNU7-140P | turquoise |
| RNU7-171P | turquoise |
| RNU7-181P | turquoise |
| RNU7-18P | turquoise |
| RNU7-3P | turquoise |
| RNU7-40P | turquoise |
| RNU7-41P | turquoise |
| RNU7-45P | turquoise |
| RNU7-48P | turquoise |
| RNU7-49P | turquoise |
| RNU7-4P | turquoise |
| RNU7-70P | turquoise |
| RNU7-71P | turquoise |
| RNU7-75P | turquoise |
| RNU7-79P | turquoise |
| RNU7-80P | turquoise |
| RNU7-84P | turquoise |
| RNVU1-10 | turquoise |
| RNVU1-15 | turquoise |
| RNVU1-3 | turquoise |
| RNVU1-7 | turquoise |
| RNY1 | turquoise |
| RNY1P13 | turquoise |
| RNY1P16 | turquoise |
| RNY1P9 | turquoise |
| RNY3 | turquoise |
| RNY4 | turquoise |
| RNY4P19 | turquoise |
| RNY4P25 | turquoise |
| RNY4P7 | turquoise |
| ROBO1 | turquoise |
| ROBO2 | turquoise |
| ROCK1 | turquoise |
| ROCK2 | turquoise |
| ROM1 | turquoise |
| RORA | turquoise |
| ROS1 | turquoise |
| RP1-101A2.1 | turquoise |
| RP1-102E24.10 | turquoise |
| RP1-122P22.2 | turquoise |
| RP1-127H14.3 | turquoise |
| RP1-12G14.7 | turquoise |
| RP1-134E15.3 | turquoise |
| RP1-151F17.2 | turquoise |
| RP1-152L7.5 | turquoise |
| RP1-161P9.5 | turquoise |
| RP1-168P16.2 | turquoise |
| RP1-179N16.3 | turquoise |
| RP1-193H18.2 | turquoise |
| RP1-197B17.5 | turquoise |
| RP1-206D15.5 | turquoise |
| RP1-206D15.6 | turquoise |
| RP1-224A6.8 | turquoise |
| RP1-228H13.5 | turquoise |
| RP1-228P16.1 | turquoise |
| RP1-229K20.5 | turquoise |
| RP1-244F24.1 | turquoise |
| RP1-257I20.14 | turquoise |
| RP1-261D10.2 | turquoise |
| RP1-267L14.6 | turquoise |
| RP1-27K12.4 | turquoise |
| RP1-283E3.4 | turquoise |
| RP1-283E3.8 | turquoise |
| RP1-30M3.5 | turquoise |
| RP1-30M3.6 | turquoise |
| RP1-43E13.2 | turquoise |
| RP1-50J22.4 | turquoise |
| RP1-68D18.2 | turquoise |
| RP1-68D18.4 | turquoise |
| RP1-92O14.6 | turquoise |
| RP1-95L4.4 | turquoise |
| RP11-101E13.5 | turquoise |
| RP11-1020A11.2 | turquoise |
| RP11-1023L17.1 | turquoise |
| RP11-1024P17.1 | turquoise |
| RP11-103B5.4 | turquoise |
| RP11-1046B16.3 | turquoise |
| RP11-104L21.2 | turquoise |
| RP11-104L21.3 | turquoise |
| RP11-104N10.1 | turquoise |
| RP11-104N10.2 | turquoise |
| RP11-105N14.1 | turquoise |
| RP11-108M9.5 | turquoise |
| RP11-1094H24.4 | turquoise |
| RP11-1094M14.5 | turquoise |
| RP11-10A14.3 | turquoise |
| RP11-10C24.1 | turquoise |
| RP11-10C24.2 | turquoise |
| RP11-10J21.4 | turquoise |
| RP11-10L12.4 | turquoise |
| RP11-10N23.2 | turquoise |
| RP11-1100L3.8 | turquoise |
| RP11-1105G2.3 | turquoise |
| RP11-1109F11.3 | turquoise |
| RP11-110I1.11 | turquoise |
| RP11-110I1.12 | turquoise |
| RP11-110I1.5 | turquoise |
| RP11-1114A5.4 | turquoise |
| RP11-111M22.4 | turquoise |
| RP11-112J3.16 | turquoise |
| RP11-1136G4.2 | turquoise |
| RP11-113K21.6 | turquoise |
| RP11-1148L6.5 | turquoise |
| RP11-1148L6.8 | turquoise |
| RP11-1149O23.2 | turquoise |
| RP11-114M1.1 | turquoise |
| RP11-115H15.2 | turquoise |
| RP11-116N8.4 | turquoise |
| RP11-119B16.2 | turquoise |
| RP11-119F19.5 | turquoise |
| RP11-119F7.5 | turquoise |
| RP11-11N7.5 | turquoise |
| RP11-120E11.2 | turquoise |
| RP11-120M18.5 | turquoise |
| RP11-121A14.2 | turquoise |
| RP11-121A14.3 | turquoise |
| RP11-121C2.2 | turquoise |
| RP11-124A7.1 | turquoise |
| RP11-1252D15.1 | turquoise |
| RP11-1260E13.3 | turquoise |
| RP11-1275H24.3 | turquoise |
| RP11-1277A3.1 | turquoise |
| RP11-1277A3.2 | turquoise |
| RP11-127B20.2 | turquoise |
| RP11-129M16.4 | turquoise |
| RP11-131L12.3 | turquoise |
| RP11-131L23.2 | turquoise |
| RP11-132F7.2 | turquoise |
| RP11-134G8.5 | turquoise |
| RP11-134L10.1 | turquoise |
| RP11-135F9.3 | turquoise |
| RP11-136H19.1 | turquoise |
| RP11-1376P16.2 | turquoise |
| RP11-138A9.1 | turquoise |
| RP11-138A9.2 | turquoise |
| RP11-138I1.3 | turquoise |
| RP11-1398P2.1 | turquoise |
| RP11-1399P15.1 | turquoise |
| RP11-139H15.5 | turquoise |
| RP11-139H15.6 | turquoise |
| RP11-140H17.2 | turquoise |
| RP11-140I16.3 | turquoise |
| RP11-140K17.3 | turquoise |
| RP11-141O11.2 | turquoise |
| RP11-143J12.3 | turquoise |
| RP11-143K11.7 | turquoise |
| RP11-145M9.6 | turquoise |
| RP11-147I3.1 | turquoise |
| RP11-147L13.11 | turquoise |
| RP11-147L13.12 | turquoise |
| RP11-147L13.15 | turquoise |
| RP11-150C16.1 | turquoise |
| RP11-152N13.16 | turquoise |
| RP11-153F5.7 | turquoise |
| RP11-156E6.1 | turquoise |
| RP11-156K23.3 | turquoise |
| RP11-156P1.3 | turquoise |
| RP11-158H5.2 | turquoise |
| RP11-158H5.7 | turquoise |
| RP11-158K1.3 | turquoise |
| RP11-159D12.2 | turquoise |
| RP11-159D12.8 | turquoise |
| RP11-159G9.5 | turquoise |
| RP11-15A1.8 | turquoise |
| RP11-15F12.1 | turquoise |
| RP11-15H20.6 | turquoise |
| RP11-15I11.3 | turquoise |
| RP11-15L13.4 | turquoise |
| RP11-161H23.8 | turquoise |
| RP11-161M6.6 | turquoise |
| RP11-162A12.2 | turquoise |
| RP11-162A12.4 | turquoise |
| RP11-162J8.3 | turquoise |
| RP11-163E9.1 | turquoise |
| RP11-163E9.2 | turquoise |
| RP11-164O23.8 | turquoise |
| RP11-164P12.4 | turquoise |
| RP11-165J3.6 | turquoise |
| RP11-166B2.3 | turquoise |
| RP11-166B2.8 | turquoise |
| RP11-166D19.1 | turquoise |
| RP11-167N4.2 | turquoise |
| RP11-168F9.2 | turquoise |
| RP11-16E12.1 | turquoise |
| RP11-16E23.3 | turquoise |
| RP11-173A6.2 | turquoise |
| RP11-173M1.5 | turquoise |
| RP11-173M11.2 | turquoise |
| RP11-174G6.5 | turquoise |
| RP11-174N3.4 | turquoise |
| RP11-175O19.4 | turquoise |
| RP11-177C12.1 | turquoise |
| RP11-177J6.1 | turquoise |
| RP11-17E13.2 | turquoise |
| RP11-17P16.2 | turquoise |
| RP11-180M15.7 | turquoise |
| RP11-181K3.4 | turquoise |
| RP11-182I10.1 | turquoise |
| RP11-182J1.12 | turquoise |
| RP11-187A9.3 | turquoise |
| RP11-187C18.3 | turquoise |
| RP11-191N8.2 | turquoise |
| RP11-195B17.1 | turquoise |
| RP11-196G11.4 | turquoise |
| RP11-196G11.5 | turquoise |
| RP11-196G18.22 | turquoise |
| RP11-200A1.1 | turquoise |
| RP11-203M5.7 | turquoise |
| RP11-204M4.2 | turquoise |
| RP11-206L10.5 | turquoise |
| RP11-208G20.2 | turquoise |
| RP11-20I20.4 | turquoise |
| RP11-20O24.1 | turquoise |
| RP11-211G3.2 | turquoise |
| RP11-213G2.3 | turquoise |
| RP11-213H15.1 | turquoise |
| RP11-214K3.19 | turquoise |
| RP11-214K3.20 | turquoise |
| RP11-214K3.21 | turquoise |
| RP11-214N15.5 | turquoise |
| RP11-216B9.6 | turquoise |
| RP11-218F10.3 | turquoise |
| RP11-219D15.3 | turquoise |
| RP11-219E7.1 | turquoise |
| RP11-225B17.2 | turquoise |
| RP11-227F19.5 | turquoise |
| RP11-227G15.10 | turquoise |
| RP11-22C11.2 | turquoise |
| RP11-230F18.6 | turquoise |
| RP11-231G3.1 | turquoise |
| RP11-231L11.3 | turquoise |
| RP11-235E17.5 | turquoise |
| RP11-23J18.1 | turquoise |
| RP11-23N2.4 | turquoise |
| RP11-242C19.2 | turquoise |
| RP11-242D8.1 | turquoise |
| RP11-242D8.2 | turquoise |
| RP11-242D8.3 | turquoise |
| RP11-243J16.7 | turquoise |
| RP11-244H3.1 | turquoise |
| RP11-245J9.5 | turquoise |
| RP11-248J18.2 | turquoise |
| RP11-248J18.3 | turquoise |
| RP11-249L21.4 | turquoise |
| RP11-250B2.6 | turquoise |
| RP11-251G23.5 | turquoise |
| RP11-252A24.2 | turquoise |
| RP11-252K23.2 | turquoise |
| RP11-254B13.4 | turquoise |
| RP11-255H23.2 | turquoise |
| RP11-255H23.5 | turquoise |
| RP11-257O5.2 | turquoise |
| RP11-257O5.4 | turquoise |
| RP11-258C19.4 | turquoise |
| RP11-258C19.7 | turquoise |
| RP11-258F22.1 | turquoise |
| RP11-259K15.2 | turquoise |
| RP11-259K5.2 | turquoise |
| RP11-25C19.3 | turquoise |
| RP11-260M2.1 | turquoise |
| RP11-264I13.2 | turquoise |
| RP11-267M23.1 | turquoise |
| RP11-269G24.7 | turquoise |
| RP11-26J3.3 | turquoise |
| RP11-273B20.1 | turquoise |
| RP11-274B21.1 | turquoise |
| RP11-274B21.10 | turquoise |
| RP11-274B21.13 | turquoise |
| RP11-274B21.14 | turquoise |
| RP11-274E7.2 | turquoise |
| RP11-277P12.20 | turquoise |
| RP11-278A23.2 | turquoise |
| RP11-278C7.1 | turquoise |
| RP11-278C7.4 | turquoise |
| RP11-278C7.5 | turquoise |
| RP11-278J6.5 | turquoise |
| RP11-279F6.2 | turquoise |
| RP11-27I1.4 | turquoise |
| RP11-27M24.2 | turquoise |
| RP11-282O18.3 | turquoise |
| RP11-283G6.3 | turquoise |
| RP11-283I3.6 | turquoise |
| RP11-284N8.3 | turquoise |
| RP11-286N22.16 | turquoise |
| RP11-288C18.1 | turquoise |
| RP11-288E14.2 | turquoise |
| RP11-288H12.3 | turquoise |
| RP11-28F1.2 | turquoise |
| RP11-290D2.3 | turquoise |
| RP11-290H9.4 | turquoise |
| RP11-293A21.1 | turquoise |
| RP11-295D4.3 | turquoise |
| RP11-295H24.4 | turquoise |
| RP11-295I5.3 | turquoise |
| RP11-295K3.1 | turquoise |
| RP11-295P9.12 | turquoise |
| RP11-295P9.3 | turquoise |
| RP11-296O14.3 | turquoise |
| RP11-297D21.4 | turquoise |
| RP11-298C2.1 | turquoise |
| RP11-298I3.1 | turquoise |
| RP11-298I3.6 | turquoise |
| RP11-299G20.5 | turquoise |
| RP11-299J3.6 | turquoise |
| RP11-29G8.3 | turquoise |
| RP11-29H23.7 | turquoise |
| RP11-2E11.9 | turquoise |
| RP11-2J18.1 | turquoise |
| RP11-304F15.3 | turquoise |
| RP11-305K5.1 | turquoise |
| RP11-305M3.2 | turquoise |
| RP11-305O6.4 | turquoise |
| RP11-307C18.1 | turquoise |
| RP11-308D16.2 | turquoise |
| RP11-309L24.4 | turquoise |
| RP11-314B1.2 | turquoise |
| RP11-314N13.3 | turquoise |
| RP11-316K19.3 | turquoise |
| RP11-316M21.7 | turquoise |
| RP11-319G6.1 | turquoise |
| RP11-31H5.3 | turquoise |
| RP11-320L11.2 | turquoise |
| RP11-320N7.2 | turquoise |
| RP11-321A17.6 | turquoise |
| RP11-324E6.8 | turquoise |
| RP11-324I22.3 | turquoise |
| RP11-324I22.4 | turquoise |
| RP11-324O2.3 | turquoise |
| RP11-325F22.2 | turquoise |
| RP11-325K4.2 | turquoise |
| RP11-325K4.3 | turquoise |
| RP11-326C3.16 | turquoise |
| RP11-326I11.5 | turquoise |
| RP11-327O17.2 | turquoise |
| RP11-329B9.4 | turquoise |
| RP11-330L19.1 | turquoise |
| RP11-332H14.2 | turquoise |
| RP11-332M2.1 | turquoise |
| RP11-333E1.1 | turquoise |
| RP11-333E13.2 | turquoise |
| RP11-334A14.2 | turquoise |
| RP11-336A10.5 | turquoise |
| RP11-336K24.5 | turquoise |
| RP11-337C18.8 | turquoise |
| RP11-337C18.9 | turquoise |
| RP11-337N6.3 | turquoise |
| RP11-338K17.10 | turquoise |
| RP11-338K17.6 | turquoise |
| RP11-33B1.1 | turquoise |
| RP11-33B1.2 | turquoise |
| RP11-33E12.2 | turquoise |
| RP11-342K2.1 | turquoise |
| RP11-342K6.1 | turquoise |
| RP11-342K6.2 | turquoise |
| RP11-342M1.3 | turquoise |
| RP11-343H5.4 | turquoise |
| RP11-345J18.2 | turquoise |
| RP11-345K20.2 | turquoise |
| RP11-345P4.6 | turquoise |
| RP11-345P4.7 | turquoise |
| RP11-346C20.4 | turquoise |
| RP11-347C12.12 | turquoise |
| RP11-347C18.3 | turquoise |
| RP11-347C18.5 | turquoise |
| RP11-347P5.1 | turquoise |
| RP11-348P10.2 | turquoise |
| RP11-350J20.12 | turquoise |
| RP11-350N15.4 | turquoise |
| RP11-351C21.2 | turquoise |
| RP11-351O1.4 | turquoise |
| RP11-352G18.2 | turquoise |
| RP11-352M15.2 | turquoise |
| RP11-353K11.1 | turquoise |
| RP11-354E23.5 | turquoise |
| RP11-355B11.2 | turquoise |
| RP11-355O1.11 | turquoise |
| RP11-356B19.11 | turquoise |
| RP11-356I2.4 | turquoise |
| RP11-356J5.12 | turquoise |
| RP11-357P18.2 | turquoise |
| RP11-359B12.2 | turquoise |
| RP11-359E10.1 | turquoise |
| RP11-359J14.2 | turquoise |
| RP11-359K18.4 | turquoise |
| RP11-359M6.1 | turquoise |
| RP11-35G9.3 | turquoise |
| RP11-35G9.5 | turquoise |
| RP11-35N6.1 | turquoise |
| RP11-360D2.2 | turquoise |
| RP11-360F5.3 | turquoise |
| RP11-364L4.1 | turquoise |
| RP11-366M4.11 | turquoise |
| RP11-367H1.1 | turquoise |
| RP11-36N20.1 | turquoise |
| RP11-372B4.3 | turquoise |
| RP11-373D23.2 | turquoise |
| RP11-373D23.3 | turquoise |
| RP11-378J18.8 | turquoise |
| RP11-379B8.1 | turquoise |
| RP11-379F4.6 | turquoise |
| RP11-379F4.7 | turquoise |
| RP11-379H18.1 | turquoise |
| RP11-37B2.1 | turquoise |
| RP11-380G5.2 | turquoise |
| RP11-380G5.3 | turquoise |
| RP11-380M21.2 | turquoise |
| RP11-380M21.4 | turquoise |
| RP11-383H13.1 | turquoise |
| RP11-384C4.3 | turquoise |
| RP11-384O8.1 | turquoise |
| RP11-385D13.3 | turquoise |
| RP11-385F5.4 | turquoise |
| RP11-386I14.4 | turquoise |
| RP11-388C12.8 | turquoise |
| RP11-388K2.1 | turquoise |
| RP11-390E23.6 | turquoise |
| RP11-390G14.1 | turquoise |
| RP11-391L3.5 | turquoise |
| RP11-392P7.6 | turquoise |
| RP11-394O4.5 | turquoise |
| RP11-395I14.2 | turquoise |
| RP11-395I6.3 | turquoise |
| RP11-395N3.2 | turquoise |
| RP11-396K3.1 | turquoise |
| RP11-397P13.6 | turquoise |
| RP11-397P13.7 | turquoise |
| RP11-399B17.1 | turquoise |
| RP11-399C16.3 | turquoise |
| RP11-399E6.4 | turquoise |
| RP11-399O19.9 | turquoise |
| RP11-3B12.5 | turquoise |
| RP11-3D4.2 | turquoise |
| RP11-3K16.2 | turquoise |
| RP11-400F19.18 | turquoise |
| RP11-400F19.6 | turquoise |
| RP11-400K9.4 | turquoise |
| RP11-400L8.2 | turquoise |
| RP11-400N13.2 | turquoise |
| RP11-400N13.3 | turquoise |
| RP11-403P17.6 | turquoise |
| RP11-404P21.9 | turquoise |
| RP11-407G23.4 | turquoise |
| RP11-407G23.7 | turquoise |
| RP11-407N17.6 | turquoise |
| RP11-408A13.4 | turquoise |
| RP11-408O19.5 | turquoise |
| RP11-40C6.2 | turquoise |
| RP11-415F23.2 | turquoise |
| RP11-415F23.3 | turquoise |
| RP11-415F23.4 | turquoise |
| RP11-415J8.3 | turquoise |
| RP11-417F21.1 | turquoise |
| RP11-417L19.6 | turquoise |
| RP11-418J17.1 | turquoise |
| RP11-420A23.1 | turquoise |
| RP11-420L9.2 | turquoise |
| RP11-421F16.3 | turquoise |
| RP11-421L21.3 | turquoise |
| RP11-422P24.10 | turquoise |
| RP11-422P24.11 | turquoise |
| RP11-424M22.3 | turquoise |
| RP11-425M5.7 | turquoise |
| RP11-427L15.2 | turquoise |
| RP11-427P5.3 | turquoise |
| RP11-429J17.7 | turquoise |
| RP11-432J22.2 | turquoise |
| RP11-434B12.1 | turquoise |
| RP11-434H6.6 | turquoise |
| RP11-434H6.7 | turquoise |
| RP11-438L19.1 | turquoise |
| RP11-440D17.4 | turquoise |
| RP11-440L14.1 | turquoise |
| RP11-440L14.4 | turquoise |
| RP11-446N19.1 | turquoise |
| RP11-447D11.3 | turquoise |
| RP11-448A19.1 | turquoise |
| RP11-448G15.3 | turquoise |
| RP11-449G16.1 | turquoise |
| RP11-449P15.2 | turquoise |
| RP11-44F14.10 | turquoise |
| RP11-44F14.2 | turquoise |
| RP11-44F14.6 | turquoise |
| RP11-44F14.8 | turquoise |
| RP11-44F14.9 | turquoise |
| RP11-44I10.3 | turquoise |
| RP11-44K6.2 | turquoise |
| RP11-452F19.3 | turquoise |
| RP11-452G18.2 | turquoise |
| RP11-452I5.2 | turquoise |
| RP11-452L6.5 | turquoise |
| RP11-454L9.2 | turquoise |
| RP11-457D2.3 | turquoise |
| RP11-458D21.1 | turquoise |
| RP11-458F8.1 | turquoise |
| RP11-458F8.2 | turquoise |
| RP11-458J1.1 | turquoise |
| RP11-45A17.2 | turquoise |
| RP11-45M22.2 | turquoise |
| RP11-45P15.4 | turquoise |
| RP11-460N20.4 | turquoise |
| RP11-460N20.5 | turquoise |
| RP11-463O12.5 | turquoise |
| RP11-468E2.10 | turquoise |
| RP11-46B11.2 | turquoise |
| RP11-46D6.1 | turquoise |
| RP11-46J23.1 | turquoise |
| RP11-471B22.2 | turquoise |
| RP11-473O4.4 | turquoise |
| RP11-474O21.5 | turquoise |
| RP11-475J5.4 | turquoise |
| RP11-477H21.2 | turquoise |
| RP11-477N3.1 | turquoise |
| RP11-478C6.4 | turquoise |
| RP11-478C6.6 | turquoise |
| RP11-479O9.3 | turquoise |
| RP11-480A16.1 | turquoise |
| RP11-480I12.2 | turquoise |
| RP11-481C4.1 | turquoise |
| RP11-481C4.2 | turquoise |
| RP11-481J2.4 | turquoise |
| RP11-483L5.1 | turquoise |
| RP11-484D2.4 | turquoise |
| RP11-485M7.1 | turquoise |
| RP11-486B10.4 | turquoise |
| RP11-488L18.10 | turquoise |
| RP11-488L18.4 | turquoise |
| RP11-488L18.6 | turquoise |
| RP11-48B14.1 | turquoise |
| RP11-48B3.4 | turquoise |
| RP11-48B3.5 | turquoise |
| RP11-490B18.6 | turquoise |
| RP11-490K7.4 | turquoise |
| RP11-490M8.1 | turquoise |
| RP11-492I21.1 | turquoise |
| RP11-493L12.5 | turquoise |
| RP11-495P10.1 | turquoise |
| RP11-496H1.2 | turquoise |
| RP11-497H17.1 | turquoise |
| RP11-498C9.15 | turquoise |
| RP11-498C9.4 | turquoise |
| RP11-499O7.4 | turquoise |
| RP11-499O7.7 | turquoise |
| RP11-49I11.4 | turquoise |
| RP11-4C20.3 | turquoise |
| RP11-4C20.4 | turquoise |
| RP11-4K16.2 | turquoise |
| RP11-4O1.2 | turquoise |
| RP11-500G22.5 | turquoise |
| RP11-503E24.2 | turquoise |
| RP11-504P24.2 | turquoise |
| RP11-504P24.3 | turquoise |
| RP11-504P24.9 | turquoise |
| RP11-506H20.1 | turquoise |
| RP11-507E23.1 | turquoise |
| RP11-50I19.2 | turquoise |
| RP11-513M16.7 | turquoise |
| RP11-513M16.8 | turquoise |
| RP11-517B11.7 | turquoise |
| RP11-517I3.2 | turquoise |
| RP11-521B24.4 | turquoise |
| RP11-521C22.2 | turquoise |
| RP11-523H20.3 | turquoise |
| RP11-523H24.3 | turquoise |
| RP11-524D16__A.3 | turquoise |
| RP11-526D8.11 | turquoise |
| RP11-526I2.5 | turquoise |
| RP11-527J8.1 | turquoise |
| RP11-529E10.6 | turquoise |
| RP11-529F4.1 | turquoise |
| RP11-530C5.1 | turquoise |
| RP11-530N7.3 | turquoise |
| RP11-531F16.4 | turquoise |
| RP11-532F12.5 | turquoise |
| RP11-532M24.1 | turquoise |
| RP11-533O20.2 | turquoise |
| RP11-534C12.1 | turquoise |
| RP11-535M15.2 | turquoise |
| RP11-536K7.3 | turquoise |
| RP11-537E18.1 | turquoise |
| RP11-537H15.3 | turquoise |
| RP11-539G18.1 | turquoise |
| RP11-539I5.1 | turquoise |
| RP11-53B2.6 | turquoise |
| RP11-53O19.3 | turquoise |
| RP11-540B6.6 | turquoise |
| RP11-544M22.3 | turquoise |
| RP11-546D6.3 | turquoise |
| RP11-546K22.3 | turquoise |
| RP11-548P2.2 | turquoise |
| RP11-54A4.2 | turquoise |
| RP11-54O7.3 | turquoise |
| RP11-553K8.5 | turquoise |
| RP11-553L6.5 | turquoise |
| RP11-554E23.4 | turquoise |
| RP11-554I8.2 | turquoise |
| RP11-556O9.2 | turquoise |
| RP11-562A8.4 | turquoise |
| RP11-565F19.2 | turquoise |
| RP11-566E18.1 | turquoise |
| RP11-567F11.1 | turquoise |
| RP11-568K15.1 | turquoise |
| RP11-569A11.1 | turquoise |
| RP11-571F15.2 | turquoise |
| RP11-571I18.4 | turquoise |
| RP11-571L19.8 | turquoise |
| RP11-571M6.17 | turquoise |
| RP11-571M6.18 | turquoise |
| RP11-572O6.1 | turquoise |
| RP11-572P18.1 | turquoise |
| RP11-574F21.2 | turquoise |
| RP11-574K11.24 | turquoise |
| RP11-574K11.29 | turquoise |
| RP11-574K11.5 | turquoise |
| RP11-575L7.8 | turquoise |
| RP11-576C12.1 | turquoise |
| RP11-578O24.2 | turquoise |
| RP11-57H14.4 | turquoise |
| RP11-582E3.6 | turquoise |
| RP11-583F2.5 | turquoise |
| RP11-583F2.6 | turquoise |
| RP11-585P4.5 | turquoise |
| RP11-585P4.6 | turquoise |
| RP11-588K22.2 | turquoise |
| RP11-589N15.2 | turquoise |
| RP11-58O9.2 | turquoise |
| RP11-596D21.1 | turquoise |
| RP11-598P20.3 | turquoise |
| RP11-59C5.3 | turquoise |
| RP11-59N23.1 | turquoise |
| RP11-59N23.3 | turquoise |
| RP11-5C23.1 | turquoise |
| RP11-5C23.2 | turquoise |
| RP11-603B24.1 | turquoise |
| RP11-603J24.7 | turquoise |
| RP11-609N14.4 | turquoise |
| RP11-610P16.1 | turquoise |
| RP11-611E13.2 | turquoise |
| RP11-611L7.2 | turquoise |
| RP11-611O2.6 | turquoise |
| RP11-617F23.1 | turquoise |
| RP11-617F23.2 | turquoise |
| RP11-61K9.3 | turquoise |
| RP11-61L19.3 | turquoise |
| RP11-626G11.6 | turquoise |
| RP11-62I21.1 | turquoise |
| RP11-630A13.4 | turquoise |
| RP11-631M6.3 | turquoise |
| RP11-631N16.2 | turquoise |
| RP11-631N16.4 | turquoise |
| RP11-632K20.7 | turquoise |
| RP11-635L1.2 | turquoise |
| RP11-635N19.1 | turquoise |
| RP11-638I2.10 | turquoise |
| RP11-638I2.9 | turquoise |
| RP11-641D5.2 | turquoise |
| RP11-644F5.11 | turquoise |
| RP11-647K16.1 | turquoise |
| RP11-649A18.4 | turquoise |
| RP11-649A18.5 | turquoise |
| RP11-649E7.8 | turquoise |
| RP11-649G15.2 | turquoise |
| RP11-656D10.3 | turquoise |
| RP11-658F2.8 | turquoise |
| RP11-65L3.4 | turquoise |
| RP11-664H17.1 | turquoise |
| RP11-665C16.1 | turquoise |
| RP11-667K14.3 | turquoise |
| RP11-667M19.10 | turquoise |
| RP11-669E14.6 | turquoise |
| RP11-66B24.2 | turquoise |
| RP11-66H6.4 | turquoise |
| RP11-66N24.6 | turquoise |
| RP11-676J12.7 | turquoise |
| RP11-676M6.1 | turquoise |
| RP11-677M14.2 | turquoise |
| RP11-677M14.6 | turquoise |
| RP11-67C2.2 | turquoise |
| RP11-67L2.2 | turquoise |
| RP11-67L3.5 | turquoise |
| RP11-680G24.6 | turquoise |
| RP11-685N10.1 | turquoise |
| RP11-686D22.10 | turquoise |
| RP11-686D22.4 | turquoise |
| RP11-686D22.7 | turquoise |
| RP11-686D22.8 | turquoise |
| RP11-690D19.3 | turquoise |
| RP11-690I21.1 | turquoise |
| RP11-691N7.6 | turquoise |
| RP11-692D12.1 | turquoise |
| RP11-692N5.2 | turquoise |
| RP11-694I15.7 | turquoise |
| RP11-697N18.3 | turquoise |
| RP11-69E11.8 | turquoise |
| RP11-6B19.1 | turquoise |
| RP11-6J21.2 | turquoise |
| RP11-6N17.3 | turquoise |
| RP11-700J17.1 | turquoise |
| RP11-701H24.3 | turquoise |
| RP11-701H24.5 | turquoise |
| RP11-701H24.7 | turquoise |
| RP11-701P16.2 | turquoise |
| RP11-705C15.2 | turquoise |
| RP11-705C15.3 | turquoise |
| RP11-706O15.1 | turquoise |
| RP11-707M3.3 | turquoise |
| RP11-707O23.1 | turquoise |
| RP11-707P17.2 | turquoise |
| RP11-714G18.1 | turquoise |
| RP11-717A5.2 | turquoise |
| RP11-720N19.1 | turquoise |
| RP11-722E23.2 | turquoise |
| RP11-723D22.3 | turquoise |
| RP11-727A23.5 | turquoise |
| RP11-727F15.13 | turquoise |
| RP11-730B22.1 | turquoise |
| RP11-731C17.2 | turquoise |
| RP11-731D1.4 | turquoise |
| RP11-736K20.5 | turquoise |
| RP11-738E22.3 | turquoise |
| RP11-739N20.2 | turquoise |
| RP11-73E17.2 | turquoise |
| RP11-73K9.3 | turquoise |
| RP11-73M18.10 | turquoise |
| RP11-73M18.6 | turquoise |
| RP11-73M18.7 | turquoise |
| RP11-73M18.8 | turquoise |
| RP11-745A24.1 | turquoise |
| RP11-747H7.3 | turquoise |
| RP11-74C1.2 | turquoise |
| RP11-74J13.9 | turquoise |
| RP11-755F10.1 | turquoise |
| RP11-755F10.3 | turquoise |
| RP11-757F18.5 | turquoise |
| RP11-760H22.2 | turquoise |
| RP11-762H8.2 | turquoise |
| RP11-762I7.4 | turquoise |
| RP11-764K9.4 | turquoise |
| RP11-767N6.2 | turquoise |
| RP11-767N6.7 | turquoise |
| RP11-771K4.3 | turquoise |
| RP11-774O3.3 | turquoise |
| RP11-792A8.3 | turquoise |
| RP11-792A8.4 | turquoise |
| RP11-797A18.5 | turquoise |
| RP11-797A18.6 | turquoise |
| RP11-798M19.6 | turquoise |
| RP11-79N23.1 | turquoise |
| RP11-7O11.3 | turquoise |
| RP11-806L2.6 | turquoise |
| RP11-806O11.1 | turquoise |
| RP11-809O17.1 | turquoise |
| RP11-80A15.1 | turquoise |
| RP11-80I15.1 | turquoise |
| RP11-80P20.3 | turquoise |
| RP11-815J21.1 | turquoise |
| RP11-815J21.3 | turquoise |
| RP11-815J21.4 | turquoise |
| RP11-815M8.1 | turquoise |
| RP11-817I4.1 | turquoise |
| RP11-818F20.5 | turquoise |
| RP11-818O24.2 | turquoise |
| RP11-819C21.1 | turquoise |
| RP11-81A1.6 | turquoise |
| RP11-81A22.5 | turquoise |
| RP11-823E8.3 | turquoise |
| RP11-829H16.5 | turquoise |
| RP11-831H9.3 | turquoise |
| RP11-832A4.7 | turquoise |
| RP11-83A24.2 | turquoise |
| RP11-83J16.1 | turquoise |
| RP11-843B15.4 | turquoise |
| RP11-843P14.1 | turquoise |
| RP11-843P14.2 | turquoise |
| RP11-848P1.2 | turquoise |
| RP11-848P1.3 | turquoise |
| RP11-848P1.9 | turquoise |
| RP11-849F2.5 | turquoise |
| RP11-84A14.4 | turquoise |
| RP11-84A19.4 | turquoise |
| RP11-857B24.5 | turquoise |
| RP11-85K15.3 | turquoise |
| RP11-86H7.7 | turquoise |
| RP11-872J21.5 | turquoise |
| RP11-876N24.3 | turquoise |
| RP11-876N24.4 | turquoise |
| RP11-876N24.5 | turquoise |
| RP11-87H9.2 | turquoise |
| RP11-87H9.4 | turquoise |
| RP11-87N3.6 | turquoise |
| RP11-881L2.1 | turquoise |
| RP11-884K10.6 | turquoise |
| RP11-884K10.7 | turquoise |
| RP11-888D10.4 | turquoise |
| RP11-890B15.3 | turquoise |
| RP11-894P9.1 | turquoise |
| RP11-896J10.3 | turquoise |
| RP11-89K11.1 | turquoise |
| RP11-91J19.3 | turquoise |
| RP11-923I11.6 | turquoise |
| RP11-92K15.3 | turquoise |
| RP11-93B14.10 | turquoise |
| RP11-93H24.3 | turquoise |
| RP11-93O14.2 | turquoise |
| RP11-946P6.6 | turquoise |
| RP11-95D17.1 | turquoise |
| RP11-95P2.3 | turquoise |
| RP11-964E11.2 | turquoise |
| RP11-967K21.1 | turquoise |
| RP11-968A15.2 | turquoise |
| RP11-96D1.5 | turquoise |
| RP11-96D1.8 | turquoise |
| RP11-96H19.1 | turquoise |
| RP11-972P1.10 | turquoise |
| RP11-972P1.11 | turquoise |
| RP11-973H7.3 | turquoise |
| RP11-973N13.4 | turquoise |
| RP11-977G19.11 | turquoise |
| RP11-977G19.12 | turquoise |
| RP11-97C16.1 | turquoise |
| RP11-98I9.4 | turquoise |
| RP11-996F15.5 | turquoise |
| RP11-996F15.6 | turquoise |
| RP11-9E17.1 | turquoise |
| RP13-104F24.2 | turquoise |
| RP13-188A5.1 | turquoise |
| RP13-20L14.10 | turquoise |
| RP13-20L14.6 | turquoise |
| RP13-216E22.4 | turquoise |
| RP13-216E22.5 | turquoise |
| RP13-225O21.2 | turquoise |
| RP13-314C10.5 | turquoise |
| RP13-349O20.2 | turquoise |
| RP13-36G14.4 | turquoise |
| RP13-46H24.1 | turquoise |
| RP13-554M15.2 | turquoise |
| RP13-554M15.7 | turquoise |
| RP13-638C3.2 | turquoise |
| RP13-638C3.3 | turquoise |
| RP13-638C3.6 | turquoise |
| RP13-766D20.2 | turquoise |
| RP13-870H17.3 | turquoise |
| RP13-923O23.7 | turquoise |
| RP3-323N1.2 | turquoise |
| RP3-327A19.7 | turquoise |
| RP3-331H24.7 | turquoise |
| RP3-337H4.8 | turquoise |
| RP3-340N1.2 | turquoise |
| RP3-368A4.5 | turquoise |
| RP3-368A4.6 | turquoise |
| RP3-388E23.2 | turquoise |
| RP3-406P24.5 | turquoise |
| RP3-407E4.2 | turquoise |
| RP3-407E4.3 | turquoise |
| RP3-416H24.1 | turquoise |
| RP3-449M8.6 | turquoise |
| RP3-449M8.9 | turquoise |
| RP3-449O17.1 | turquoise |
| RP3-453C12.14 | turquoise |
| RP3-465N24.5 | turquoise |
| RP3-467L1.6 | turquoise |
| RP3-468K18.6 | turquoise |
| RP3-473L9.4 | turquoise |
| RP3-475N16.1 | turquoise |
| RP3-476K8.3 | turquoise |
| RP3-508I15.10 | turquoise |
| RP3-508I15.20 | turquoise |
| RP3-508I15.22 | turquoise |
| RP3-508I15.9 | turquoise |
| RP3-523K23.2 | turquoise |
| RP3-525L6.2 | turquoise |
| RP4-539M6.20 | turquoise |
| RP4-545K15.5 | turquoise |
| RP4-563E14.1 | turquoise |
| RP4-569M23.2 | turquoise |
| RP4-584D14.5 | turquoise |
| RP4-605O3.4 | turquoise |
| RP4-613B23.1 | turquoise |
| RP4-614O4.13 | turquoise |
| RP4-620E11.8 | turquoise |
| RP4-631H13.6 | turquoise |
| RP4-647C14.2 | turquoise |
| RP4-647J21.1 | turquoise |
| RP4-657E11.10 | turquoise |
| RP4-681L3.2 | turquoise |
| RP4-681N20.5 | turquoise |
| RP4-694B14.8 | turquoise |
| RP4-710M16.2 | turquoise |
| RP4-714D9.5 | turquoise |
| RP4-717I23.2 | turquoise |
| RP4-717I23.3 | turquoise |
| RP4-724E16.2 | turquoise |
| RP4-730K3.3 | turquoise |
| RP4-740C4.7 | turquoise |
| RP4-744I24.4 | turquoise |
| RP4-751H13.5 | turquoise |
| RP4-753P9.3 | turquoise |
| RP4-756H11.3 | turquoise |
| RP4-756H11.5 | turquoise |
| RP4-769N13.6 | turquoise |
| RP4-778K6.3 | turquoise |
| RP4-782L23.1 | turquoise |
| RP4-794H19.1 | turquoise |
| RP4-798A17.5 | turquoise |
| RP4-800M22.1 | turquoise |
| RP5-1009E24.8 | turquoise |
| RP5-1033H22.2 | turquoise |
| RP5-1042I8.7 | turquoise |
| RP5-1057I20.5 | turquoise |
| RP5-1061H20.4 | turquoise |
| RP5-1074L1.4 | turquoise |
| RP5-1085F17.3 | turquoise |
| RP5-1112D6.4 | turquoise |
| RP5-1125A11.6 | turquoise |
| RP5-1132H15.3 | turquoise |
| RP5-1142J19.2 | turquoise |
| RP5-1148A21.3 | turquoise |
| RP5-1159O4.2 | turquoise |
| RP5-1180D12.1 | turquoise |
| RP5-821D11.7 | turquoise |
| RP5-837J1.6 | turquoise |
| RP5-855D21.1 | turquoise |
| RP5-855D21.2 | turquoise |
| RP5-855D21.3 | turquoise |
| RP5-864K19.4 | turquoise |
| RP5-874C20.6 | turquoise |
| RP5-875H18.9 | turquoise |
| RP5-884M6.1 | turquoise |
| RP5-890E16.5 | turquoise |
| RP5-894A10.2 | turquoise |
| RP5-894A10.6 | turquoise |
| RP5-903G2.2 | turquoise |
| RP5-906C1.1 | turquoise |
| RP5-965G21.6 | turquoise |
| RP5-981O7.2 | turquoise |
| RP5-991G20.1 | turquoise |
| RP5-994D16.9 | turquoise |
| RP5-997D16.2 | turquoise |
| RP5-999L4.2 | turquoise |
| RP6-159A1.4 | turquoise |
| RP6-74O6.6 | turquoise |
| RP6-99M1.3 | turquoise |
| RP9P | turquoise |
| RPAIN | turquoise |
| RPAP2 | turquoise |
| RPE | turquoise |
| RPGR | turquoise |
| RPGRIP1L | turquoise |
| RPL13AP20 | turquoise |
| RPL17P50 | turquoise |
| RPL23AP53 | turquoise |
| RPL23AP64 | turquoise |
| RPL23AP82 | turquoise |
| RPL29P14 | turquoise |
| RPL32P1 | turquoise |
| RPL32P3 | turquoise |
| RPL36AL | turquoise |
| RPL36AP43 | turquoise |
| RPL39P36 | turquoise |
| RPL39P38 | turquoise |
| RPL41P1 | turquoise |
| RPL5P23 | turquoise |
| RPL5P30 | turquoise |
| RPL5P4 | turquoise |
| RPL7AP10 | turquoise |
| RPL7P32 | turquoise |
| RPL9P32 | turquoise |
| RPLP1P6 | turquoise |
| RPP14 | turquoise |
| RPRD1A | turquoise |
| RPRD1B | turquoise |
| RPRD2 | turquoise |
| RPS10L | turquoise |
| RPS11P5 | turquoise |
| RPS12P26 | turquoise |
| RPS15AP10 | turquoise |
| RPS15AP11 | turquoise |
| RPS15AP16 | turquoise |
| RPS19BP1 | turquoise |
| RPS19P3 | turquoise |
| RPS20P15 | turquoise |
| RPS20P33 | turquoise |
| RPS2P5 | turquoise |
| RPS4XP16 | turquoise |
| RPS4XP6 | turquoise |
| RPS4Y1 | turquoise |
| RPS6KA2-IT1 | turquoise |
| RPS6KA3 | turquoise |
| RPS6KA4 | turquoise |
| RPS6KA6 | turquoise |
| RPS6KB1 | turquoise |
| RPS6KC1 | turquoise |
| RPUSD1 | turquoise |
| RPUSD2 | turquoise |
| RPUSD3 | turquoise |
| RRAGA | turquoise |
| RRAGB | turquoise |
| RRAGC | turquoise |
| RRAGD | turquoise |
| RRAS2 | turquoise |
| RREB1 | turquoise |
| RRM2B | turquoise |
| RRN3 | turquoise |
| RRN3P1 | turquoise |
| RRN3P2 | turquoise |
| RRN3P3 | turquoise |
| RRP15 | turquoise |
| RRP7A | turquoise |
| RRP8 | turquoise |
| RRP9 | turquoise |
| RSAD2 | turquoise |
| RSBN1 | turquoise |
| RSBN1L | turquoise |
| RSF1 | turquoise |
| RSL24D1P6 | turquoise |
| RSPH3 | turquoise |
| RSPRY1 | turquoise |
| RSRC2 | turquoise |
| RSU1 | turquoise |
| RTCA-AS1 | turquoise |
| RTCB | turquoise |
| RTF1 | turquoise |
| RTFDC1 | turquoise |
| RTN2 | turquoise |
| RTN4R | turquoise |
| RTTN | turquoise |
| RUFY2 | turquoise |
| RUFY3 | turquoise |
| RUNX1 | turquoise |
| RUNX1T1 | turquoise |
| RUNX2 | turquoise |
| RUSC1-AS1 | turquoise |
| RWDD3 | turquoise |
| RWDD4 | turquoise |
| RWDD4P1 | turquoise |
| RYBP | turquoise |
| RYK | turquoise |
| RYR1 | turquoise |
| S100A10 | turquoise |
| S100A11 | turquoise |
| S100A14 | turquoise |
| S100A16 | turquoise |
| S100A2 | turquoise |
| S100A4 | turquoise |
| S100A6 | turquoise |
| S100PBP | turquoise |
| SACM1L | turquoise |
| SACS | turquoise |
| SAMD11 | turquoise |
| SAMD12 | turquoise |
| SAMD12-AS1 | turquoise |
| SAMD4A | turquoise |
| SAMD5 | turquoise |
| SAMD8 | turquoise |
| SAMD9 | turquoise |
| SAMD9L | turquoise |
| SAMM50 | turquoise |
| SAMSN1 | turquoise |
| SAP30L-AS1 | turquoise |
| SAR1A | turquoise |
| SAR1B | turquoise |
| SARM1 | turquoise |
| SARNP | turquoise |
| SARS | turquoise |
| SART1 | turquoise |
| SAT2 | turquoise |
| SATB1 | turquoise |
| SATB2 | turquoise |
| SAV1 | turquoise |
| SBF2 | turquoise |
| SBF2-AS1 | turquoise |
| SBNO1 | turquoise |
| SBSPON | turquoise |
| SC5D | turquoise |
| SCAANT1 | turquoise |
| SCAF1 | turquoise |
| SCAF11 | turquoise |
| SCAF4 | turquoise |
| SCAF8 | turquoise |
| SCAI | turquoise |
| SCAMP1 | turquoise |
| SCAMP2 | turquoise |
| SCAMP4 | turquoise |
| SCAND1 | turquoise |
| SCAP | turquoise |
| SCAPER | turquoise |
| SCARB2 | turquoise |
| SCARNA1 | turquoise |
| SCARNA22 | turquoise |
| SCARNA24 | turquoise |
| SCARNA3 | turquoise |
| SCARNA7 | turquoise |
| SCARNA8 | turquoise |
| SCARNA9 | turquoise |
| SCFD1 | turquoise |
| SCFD2 | turquoise |
| SCIN | turquoise |
| SCLT1 | turquoise |
| SCML1 | turquoise |
| SCN1B | turquoise |
| SCN9A | turquoise |
| SCNN1G | turquoise |
| SCO1 | turquoise |
| SCPEP1 | turquoise |
| SCRN1 | turquoise |
| SCRN3 | turquoise |
| SCUBE2 | turquoise |
| SCUBE3 | turquoise |
| SCYL1 | turquoise |
| SCYL2 | turquoise |
| SCYL3 | turquoise |
| SDAD1P1 | turquoise |
| SDC1 | turquoise |
| SDC4 | turquoise |
| SDCBP2-AS1 | turquoise |
| SDCCAG8 | turquoise |
| SDE2 | turquoise |
| SDF2L1 | turquoise |
| SDF4 | turquoise |
| SDK1 | turquoise |
| SDR42E1 | turquoise |
| SDSL | turquoise |
| SEC14L2 | turquoise |
| SEC16A | turquoise |
| SEC22A | turquoise |
| SEC22B | turquoise |
| SEC23A | turquoise |
| SEC23IP | turquoise |
| SEC24A | turquoise |
| SEC24B | turquoise |
| SEC24C | turquoise |
| SEC24D | turquoise |
| SEC31A | turquoise |
| SEC61A2 | turquoise |
| SEC63 | turquoise |
| SEC63P1 | turquoise |
| SECISBP2 | turquoise |
| SECTM1 | turquoise |
| SEL1L | turquoise |
| SEL1L3 | turquoise |
| SEMA3B-AS1 | turquoise |
| SEMA3C | turquoise |
| SEMA3E | turquoise |
| SEMA3F | turquoise |
| SEMA3F-AS1 | turquoise |
| SEMA4F | turquoise |
| SENCR | turquoise |
| SENP5 | turquoise |
| SENP6 | turquoise |
| SENP7 | turquoise |
| SENP8 | turquoise |
| SEPN1 | turquoise |
| SEPSECS | turquoise |
| 11-Sep | turquoise |
| 2-Sep | turquoise |
| 7-Sep | turquoise |
| SEPT7P2 | turquoise |
| 9-Sep | turquoise |
| SERAC1 | turquoise |
| SERINC2 | turquoise |
| SERINC5 | turquoise |
| SERPINB1 | turquoise |
| SERPINB6 | turquoise |
| SERPINB7 | turquoise |
| SERPINB9 | turquoise |
| SERTAD2 | turquoise |
| SERTAD3 | turquoise |
| SERTAD4 | turquoise |
| SESN3 | turquoise |
| SESTD1 | turquoise |
| SETBP1 | turquoise |
| SETD2 | turquoise |
| SETD5 | turquoise |
| SETD7 | turquoise |
| SETDB2 | turquoise |
| SETMAR | turquoise |
| SETX | turquoise |
| SF3A1 | turquoise |
| SF3A2 | turquoise |
| SF3B1 | turquoise |
| SFMBT1 | turquoise |
| SFMBT2 | turquoise |
| SFN | turquoise |
| SFPQ | turquoise |
| SFSWAP | turquoise |
| SFT2D2 | turquoise |
| SFTA2 | turquoise |
| SFXN2 | turquoise |
| SGIP1 | turquoise |
| SGK223 | turquoise |
| SGK3 | turquoise |
| SGMS1 | turquoise |
| SGMS1-AS1 | turquoise |
| SGPP1 | turquoise |
| SGSM1 | turquoise |
| SGTA | turquoise |
| SGTB | turquoise |
| SH2B2 | turquoise |
| SH2D3A | turquoise |
| SH3BGRL3 | turquoise |
| SH3BP2 | turquoise |
| SH3GL1 | turquoise |
| SH3GLB1 | turquoise |
| SH3PXD2A | turquoise |
| SH3RF1 | turquoise |
| SH3RF2 | turquoise |
| SH3YL1 | turquoise |
| SHANK2 | turquoise |
| SHARPIN | turquoise |
| SHB | turquoise |
| SHISA3 | turquoise |
| SHISA4 | turquoise |
| SHISA5 | turquoise |
| SHKBP1 | turquoise |
| SHOC2 | turquoise |
| SHPRH | turquoise |
| SHQ1 | turquoise |
| SHROOM2 | turquoise |
| SHROOM3 | turquoise |
| SIAH1 | turquoise |
| SIDT1 | turquoise |
| SIGLEC6 | turquoise |
| SIK2 | turquoise |
| SIK3 | turquoise |
| SIKE1 | turquoise |
| SIL1 | turquoise |
| SIMC1 | turquoise |
| SIN3A | turquoise |
| SIPA1 | turquoise |
| SIPA1L1 | turquoise |
| SIPA1L2 | turquoise |
| SIRT1 | turquoise |
| SIRT2 | turquoise |
| SIRT6 | turquoise |
| SIX4 | turquoise |
| SIX5 | turquoise |
| SKAP2 | turquoise |
| SKIL | turquoise |
| SKIV2L2 | turquoise |
| SKP1P1 | turquoise |
| SLAIN1 | turquoise |
| SLAIN2 | turquoise |
| SLAMF9 | turquoise |
| SLC10A2 | turquoise |
| SLC10A3 | turquoise |
| SLC10A7 | turquoise |
| SLC11A2 | turquoise |
| SLC12A2 | turquoise |
| SLC12A6 | turquoise |
| SLC15A4 | turquoise |
| SLC16A10 | turquoise |
| SLC16A12 | turquoise |
| SLC16A13 | turquoise |
| SLC16A3 | turquoise |
| SLC16A7 | turquoise |
| SLC16A9 | turquoise |
| SLC17A5 | turquoise |
| SLC18A2 | turquoise |
| SLC18B1 | turquoise |
| SLC19A2 | turquoise |
| SLC1A5 | turquoise |
| SLC20A1 | turquoise |
| SLC20A2 | turquoise |
| SLC22A15 | turquoise |
| SLC22A5 | turquoise |
| SLC23A2 | turquoise |
| SLC24A1 | turquoise |
| SLC25A1 | turquoise |
| SLC25A11 | turquoise |
| SLC25A12 | turquoise |
| SLC25A15 | turquoise |
| SLC25A16 | turquoise |
| SLC25A20 | turquoise |
| SLC25A21-AS1 | turquoise |
| SLC25A23 | turquoise |
| SLC25A24 | turquoise |
| SLC25A27 | turquoise |
| SLC25A3 | turquoise |
| SLC25A30 | turquoise |
| SLC25A32 | turquoise |
| SLC25A36 | turquoise |
| SLC25A38 | turquoise |
| SLC25A4 | turquoise |
| SLC25A40 | turquoise |
| SLC25A46 | turquoise |
| SLC25A5-AS1 | turquoise |
| SLC25A51 | turquoise |
| SLC25A6 | turquoise |
| SLC26A2 | turquoise |
| SLC27A4 | turquoise |
| SLC28A3 | turquoise |
| SLC29A1 | turquoise |
| SLC2A12 | turquoise |
| SLC2A13 | turquoise |
| SLC2A4RG | turquoise |
| SLC2A8 | turquoise |
| SLC30A1 | turquoise |
| SLC30A4 | turquoise |
| SLC30A5 | turquoise |
| SLC30A6 | turquoise |
| SLC30A7 | turquoise |
| SLC30A9 | turquoise |
| SLC33A1 | turquoise |
| SLC35A3 | turquoise |
| SLC35A4 | turquoise |
| SLC35A5 | turquoise |
| SLC35B3 | turquoise |
| SLC35B4 | turquoise |
| SLC35C1 | turquoise |
| SLC35D1 | turquoise |
| SLC35E2 | turquoise |
| SLC35E3 | turquoise |
| SLC35E4 | turquoise |
| SLC35F5 | turquoise |
| SLC35F6 | turquoise |
| SLC35G1 | turquoise |
| SLC35G2 | turquoise |
| SLC36A1 | turquoise |
| SLC36A4 | turquoise |
| SLC37A3 | turquoise |
| SLC38A2 | turquoise |
| SLC38A4 | turquoise |
| SLC38A6 | turquoise |
| SLC38A9 | turquoise |
| SLC39A1 | turquoise |
| SLC39A10 | turquoise |
| SLC39A13 | turquoise |
| SLC39A3 | turquoise |
| SLC39A4 | turquoise |
| SLC39A6 | turquoise |
| SLC39A9 | turquoise |
| SLC3A2 | turquoise |
| SLC41A2 | turquoise |
| SLC41A3 | turquoise |
| SLC43A3 | turquoise |
| SLC44A1 | turquoise |
| SLC44A5 | turquoise |
| SLC45A1 | turquoise |
| SLC46A3 | turquoise |
| SLC48A1 | turquoise |
| SLC4A4 | turquoise |
| SLC4A7 | turquoise |
| SLC4A8 | turquoise |
| SLC51A | turquoise |
| SLC5A1 | turquoise |
| SLC5A3 | turquoise |
| SLC5A8 | turquoise |
| SLC6A1 | turquoise |
| SLC6A14 | turquoise |
| SLC6A16 | turquoise |
| SLC6A9 | turquoise |
| SLC7A4 | turquoise |
| SLC7A6 | turquoise |
| SLC7A6OS | turquoise |
| SLC7A8 | turquoise |
| SLC8A1 | turquoise |
| SLC8B1 | turquoise |
| SLC9A3R1 | turquoise |
| SLC9A3R2 | turquoise |
| SLC9A6 | turquoise |
| SLC9A7 | turquoise |
| SLCO1B3 | turquoise |
| SLCO4A1 | turquoise |
| SLCO4A1-AS1 | turquoise |
| SLFN12 | turquoise |
| SLFN12L | turquoise |
| SLFN13 | turquoise |
| SLFN5 | turquoise |
| SLITRK6 | turquoise |
| SLK | turquoise |
| SLMAP | turquoise |
| SLPI | turquoise |
| SLTM | turquoise |
| SLU7 | turquoise |
| SLX4IP | turquoise |
| SMAD1 | turquoise |
| SMAD2 | turquoise |
| SMAD4 | turquoise |
| SMAD5 | turquoise |
| SMAD9 | turquoise |
| SMAGP | turquoise |
| SMAP1 | turquoise |
| SMARCA5 | turquoise |
| SMARCAD1 | turquoise |
| SMARCC2 | turquoise |
| SMARCD2 | turquoise |
| SMARCE1 | turquoise |
| SMC5 | turquoise |
| SMC5-AS1 | turquoise |
| SMCHD1 | turquoise |
| SMCO4 | turquoise |
| SMCR8 | turquoise |
| SMEK1 | turquoise |
| SMEK2 | turquoise |
| SMG1 | turquoise |
| SMG6 | turquoise |
| SMG7 | turquoise |
| SMIM1 | turquoise |
| SMIM13 | turquoise |
| SMIM14 | turquoise |
| SMIM8 | turquoise |
| SMN2 | turquoise |
| SMOX | turquoise |
| SMPD1 | turquoise |
| SMPDL3A | turquoise |
| SMTNL1 | turquoise |
| SMU1 | turquoise |
| SMURF1 | turquoise |
| SMURF2 | turquoise |
| SMYD3 | turquoise |
| SMYD4 | turquoise |
| SNAI1 | turquoise |
| SNAP23 | turquoise |
| SNAPC2 | turquoise |
| SNAPC3 | turquoise |
| SNCAIP | turquoise |
| SNCG | turquoise |
| SNHG14 | turquoise |
| SNHG16 | turquoise |
| SNHG3 | turquoise |
| SNHG4 | turquoise |
| SNIP1 | turquoise |
| SNN | turquoise |
| SNORA11 | turquoise |
| SNORA12 | turquoise |
| SNORA13 | turquoise |
| SNORA14A | turquoise |
| SNORA14B | turquoise |
| SNORA16B | turquoise |
| SNORA2 | turquoise |
| SNORA20 | turquoise |
| SNORA22 | turquoise |
| SNORA23 | turquoise |
| SNORA26 | turquoise |
| SNORA28 | turquoise |
| SNORA2A | turquoise |
| SNORA33 | turquoise |
| SNORA34 | turquoise |
| SNORA37 | turquoise |
| SNORA38 | turquoise |
| SNORA38B | turquoise |
| SNORA42 | turquoise |
| SNORA45B | turquoise |
| SNORA46 | turquoise |
| SNORA47 | turquoise |
| SNORA49 | turquoise |
| SNORA53 | turquoise |
| SNORA54 | turquoise |
| SNORA55 | turquoise |
| SNORA5A | turquoise |
| SNORA60 | turquoise |
| SNORA65 | turquoise |
| SNORA66 | turquoise |
| SNORA70G | turquoise |
| SNORA71A | turquoise |
| SNORA71B | turquoise |
| SNORA71D | turquoise |
| SNORA74A | turquoise |
| SNORA74B | turquoise |
| SNORA7B | turquoise |
| SNORA80A | turquoise |
| SNORA80B | turquoise |
| SNORA80E | turquoise |
| SNORD100 | turquoise |
| SNORD101 | turquoise |
| SNORD11 | turquoise |
| SNORD112 | turquoise |
| SNORD116-24 | turquoise |
| SNORD116-4 | turquoise |
| SNORD117 | turquoise |
| SNORD123 | turquoise |
| SNORD12B | turquoise |
| SNORD12C | turquoise |
| SNORD14A | turquoise |
| SNORD14E | turquoise |
| SNORD15B | turquoise |
| SNORD19 | turquoise |
| SNORD1B | turquoise |
| SNORD36C | turquoise |
| SNORD46 | turquoise |
| SNORD53_SNORD92 | turquoise |
| SNORD56B | turquoise |
| SNORD59A | turquoise |
| SNORD6 | turquoise |
| SNORD60 | turquoise |
| SNORD62B | turquoise |
| SNORD63 | turquoise |
| SNORD67 | turquoise |
| SNORD69 | turquoise |
| SNORD7 | turquoise |
| SNORD71 | turquoise |
| SNORD72 | turquoise |
| SNORD73 | turquoise |
| SNORD8 | turquoise |
| SNORD83A | turquoise |
| SNORD88 | turquoise |
| SNORD89 | turquoise |
| SNORD9 | turquoise |
| SNORD93 | turquoise |
| SNORD94 | turquoise |
| SNRNP48 | turquoise |
| SNRPEP10 | turquoise |
| SNRPGP14 | turquoise |
| SNRPGP15 | turquoise |
| SNRPGP18 | turquoise |
| SNRPGP4 | turquoise |
| SNRPN | turquoise |
| SNTA1 | turquoise |
| SNTB1 | turquoise |
| SNTB2 | turquoise |
| SNX12 | turquoise |
| SNX13 | turquoise |
| SNX14 | turquoise |
| SNX16 | turquoise |
| SNX17 | turquoise |
| SNX18 | turquoise |
| SNX19 | turquoise |
| SNX21 | turquoise |
| SNX24 | turquoise |
| SNX27 | turquoise |
| SNX29 | turquoise |
| SNX30 | turquoise |
| SNX4 | turquoise |
| SNX5 | turquoise |
| SNX7 | turquoise |
| SOAT1 | turquoise |
| SOCS4 | turquoise |
| SOCS5 | turquoise |
| SOCS6 | turquoise |
| SOCS7 | turquoise |
| SOD2 | turquoise |
| SOGA1 | turquoise |
| SON | turquoise |
| SORBS2 | turquoise |
| SORL1 | turquoise |
| SOS1 | turquoise |
| SOS1-IT1 | turquoise |
| SOS2 | turquoise |
| SOWAHC | turquoise |
| SP1 | turquoise |
| SP100 | turquoise |
| SP110 | turquoise |
| SP140L | turquoise |
| SP2 | turquoise |
| SP2-AS1 | turquoise |
| SP3 | turquoise |
| SP3P | turquoise |
| SP4 | turquoise |
| SPAG1 | turquoise |
| SPAG7 | turquoise |
| SPAG9 | turquoise |
| SPAST | turquoise |
| SPATA13 | turquoise |
| SPATA2L | turquoise |
| SPATA5 | turquoise |
| SPATA6 | turquoise |
| SPATA6L | turquoise |
| SPATA7 | turquoise |
| SPATC1L | turquoise |
| SPCS3 | turquoise |
| SPDYE3 | turquoise |
| SPECC1 | turquoise |
| SPEN | turquoise |
| SPG11 | turquoise |
| SPICE1 | turquoise |
| SPIDR | turquoise |
| SPIN1 | turquoise |
| SPIN3 | turquoise |
| SPIN4 | turquoise |
| SPINK13 | turquoise |
| SPINT1 | turquoise |
| SPINT2 | turquoise |
| SPIRE1 | turquoise |
| SPOP | turquoise |
| SPOPL | turquoise |
| SPPL2A | turquoise |
| SPR | turquoise |
| SPRED1 | turquoise |
| SPRED2 | turquoise |
| SPRTN | turquoise |
| SPRY1 | turquoise |
| SPRY4-IT1 | turquoise |
| SPRYD3 | turquoise |
| SPRYD4 | turquoise |
| SPSB2 | turquoise |
| SPTB | turquoise |
| SPTLC2 | turquoise |
| SPTLC3 | turquoise |
| SPTY2D1 | turquoise |
| SQRDL | turquoise |
| SQSTM1 | turquoise |
| SRBD1 | turquoise |
| SRC | turquoise |
| SRD5A1 | turquoise |
| SRD5A3-AS1 | turquoise |
| SREBF2 | turquoise |
| SREK1 | turquoise |
| SREK1IP1 | turquoise |
| SRFBP1 | turquoise |
| SRGAP1 | turquoise |
| SRGAP2 | turquoise |
| SRP14 | turquoise |
| SRP54-AS1 | turquoise |
| SRPK2 | turquoise |
| SRRM1 | turquoise |
| SRSF10 | turquoise |
| SRSF11 | turquoise |
| SRSF12 | turquoise |
| SRSF6 | turquoise |
| SS18 | turquoise |
| SS18L1 | turquoise |
| SSBP2 | turquoise |
| SSH1 | turquoise |
| SSH2 | turquoise |
| SSH3 | turquoise |
| SSR1 | turquoise |
| SSU72 | turquoise |
| ST13 | turquoise |
| ST20 | turquoise |
| ST3GAL4 | turquoise |
| ST3GAL5 | turquoise |
| ST3GAL5-AS1 | turquoise |
| ST3GAL6 | turquoise |
| ST6GAL1 | turquoise |
| ST6GALNAC4 | turquoise |
| ST7L | turquoise |
| ST8SIA4 | turquoise |
| STAG1 | turquoise |
| STAG2 | turquoise |
| STAG3 | turquoise |
| STAG3L2 | turquoise |
| STAG3L4 | turquoise |
| STAM | turquoise |
| STAM2 | turquoise |
| STAMBPL1 | turquoise |
| STAP2 | turquoise |
| STARD10 | turquoise |
| STARD4 | turquoise |
| STARD4-AS1 | turquoise |
| STARD7 | turquoise |
| STARD7-AS1 | turquoise |
| STAT4 | turquoise |
| STAU2 | turquoise |
| STEAP2 | turquoise |
| STEAP4 | turquoise |
| STIM1 | turquoise |
| STIM2 | turquoise |
| STK17A | turquoise |
| STK17B | turquoise |
| STK3 | turquoise |
| STK31 | turquoise |
| STK32A | turquoise |
| STK32B | turquoise |
| STK32C | turquoise |
| STK38 | turquoise |
| STK38L | turquoise |
| STK4 | turquoise |
| STK40 | turquoise |
| STMN3 | turquoise |
| STOML1 | turquoise |
| STON1 | turquoise |
| STON2 | turquoise |
| STOX2 | turquoise |
| STRADA | turquoise |
| STRADB | turquoise |
| STRBP | turquoise |
| STRIP1 | turquoise |
| STRIP2 | turquoise |
| STRN | turquoise |
| STRN3 | turquoise |
| STRN4 | turquoise |
| STS | turquoise |
| STT3B | turquoise |
| STUB1 | turquoise |
| STX17 | turquoise |
| STX19 | turquoise |
| STX2 | turquoise |
| STX3 | turquoise |
| STX5 | turquoise |
| STX6 | turquoise |
| STX7 | turquoise |
| STXBP2 | turquoise |
| STXBP3 | turquoise |
| STXBP4 | turquoise |
| STXBP5 | turquoise |
| STYX | turquoise |
| SUCNR1 | turquoise |
| SUCO | turquoise |
| SUDS3 | turquoise |
| SUGP2 | turquoise |
| SUGT1 | turquoise |
| SUGT1P | turquoise |
| SUGT1P3 | turquoise |
| SULT1A2 | turquoise |
| SULT1C2 | turquoise |
| SULT2B1 | turquoise |
| SUMF1 | turquoise |
| SUMO1P3 | turquoise |
| SUN1 | turquoise |
| SUPT20H | turquoise |
| SUPT3H | turquoise |
| SUPT7L | turquoise |
| SURF2 | turquoise |
| SUSD1 | turquoise |
| SUV420H1 | turquoise |
| SUZ12P1 | turquoise |
| SVIL | turquoise |
| SVIL-AS1 | turquoise |
| SVIP | turquoise |
| SWAP70 | turquoise |
| SWSAP1 | turquoise |
| SWT1 | turquoise |
| SYCE3 | turquoise |
| SYCP2 | turquoise |
| SYDE2 | turquoise |
| SYF2 | turquoise |
| SYNCRIP | turquoise |
| SYNE1 | turquoise |
| SYNE2 | turquoise |
| SYNGR2 | turquoise |
| SYNJ1 | turquoise |
| SYNJ2 | turquoise |
| SYNJ2BP | turquoise |
| SYNPO2 | turquoise |
| SYNRG | turquoise |
| SYS1 | turquoise |
| SYT2 | turquoise |
| SYTL2 | turquoise |
| SYTL4 | turquoise |
| SZRD1 | turquoise |
| TAB1 | turquoise |
| TAB2 | turquoise |
| TAB3 | turquoise |
| TAC4 | turquoise |
| TACSTD2 | turquoise |
| TADA1 | turquoise |
| TADA2A | turquoise |
| TADA3 | turquoise |
| TAF1 | turquoise |
| TAF1A | turquoise |
| TAF1B | turquoise |
| TAF1D | turquoise |
| TAF2 | turquoise |
| TAF3 | turquoise |
| TAF4 | turquoise |
| TAF4B | turquoise |
| TAF8 | turquoise |
| TAF9B | turquoise |
| TAF9P3 | turquoise |
| TAGLN2 | turquoise |
| TALDO1 | turquoise |
| TAMM41 | turquoise |
| TANC1 | turquoise |
| TANC2 | turquoise |
| TANGO6 | turquoise |
| TANK | turquoise |
| TAOK1 | turquoise |
| TAOK3 | turquoise |
| TAPBPL | turquoise |
| TAPT1 | turquoise |
| TAPT1-AS1 | turquoise |
| TARBP1 | turquoise |
| TARSL2 | turquoise |
| TAS2R14 | turquoise |
| TAS2R15P | turquoise |
| TAS2R20 | turquoise |
| TAS2R5 | turquoise |
| TASP1 | turquoise |
| TATDN2P2 | turquoise |
| TAX1BP3 | turquoise |
| TBC1D12 | turquoise |
| TBC1D14 | turquoise |
| TBC1D15 | turquoise |
| TBC1D19 | turquoise |
| TBC1D22A | turquoise |
| TBC1D23 | turquoise |
| TBC1D25 | turquoise |
| TBC1D32 | turquoise |
| TBC1D4 | turquoise |
| TBC1D5 | turquoise |
| TBC1D8B | turquoise |
| TBC1D9 | turquoise |
| TBCAP1 | turquoise |
| TBCB | turquoise |
| TBCEL | turquoise |
| TBCK | turquoise |
| TBK1 | turquoise |
| TBL1XR1 | turquoise |
| TBL3 | turquoise |
| TBRG1 | turquoise |
| TC2N | turquoise |
| TCAF1 | turquoise |
| TCAF2 | turquoise |
| TCAIM | turquoise |
| TCEA1 | turquoise |
| TCEA2 | turquoise |
| TCEA3 | turquoise |
| TCEAL3 | turquoise |
| TCEAL4 | turquoise |
| TCEANC | turquoise |
| TCEANC2 | turquoise |
| TCEB1P19 | turquoise |
| TCEB3 | turquoise |
| TCEB3-AS1 | turquoise |
| TCERG1 | turquoise |
| TCF12 | turquoise |
| TCF20 | turquoise |
| TCF4 | turquoise |
| TCF7L1 | turquoise |
| TCF7L2 | turquoise |
| TCHP | turquoise |
| TCN2 | turquoise |
| TCP11L1 | turquoise |
| TCP11L2 | turquoise |
| TCTA | turquoise |
| TCTE3 | turquoise |
| TCTN3 | turquoise |
| TDRD3 | turquoise |
| TDRD7 | turquoise |
| TDRP | turquoise |
| TEAD1 | turquoise |
| TEAD2 | turquoise |
| TEAD4 | turquoise |
| TEC | turquoise |
| TECR | turquoise |
| TEFM | turquoise |
| TENM1 | turquoise |
| TEP1 | turquoise |
| TERF1 | turquoise |
| TERF2 | turquoise |
| TESK1 | turquoise |
| TESK2 | turquoise |
| TET2 | turquoise |
| TET3 | turquoise |
| TEX10 | turquoise |
| TEX2 | turquoise |
| TEX261 | turquoise |
| TEX264 | turquoise |
| TEX9 | turquoise |
| TFCP2L1 | turquoise |
| TFDP2 | turquoise |
| TFE3 | turquoise |
| TFEC | turquoise |
| TFIP11 | turquoise |
| TFPI | turquoise |
| TFPT | turquoise |
| TGFBR1 | turquoise |
| TGFBRAP1 | turquoise |
| TGS1 | turquoise |
| THADA | turquoise |
| THAP11 | turquoise |
| THAP2 | turquoise |
| THAP4 | turquoise |
| THAP5 | turquoise |
| THAP6 | turquoise |
| THAP7 | turquoise |
| THAP7-AS1 | turquoise |
| THAP8 | turquoise |
| THAP9 | turquoise |
| THAP9-AS1 | turquoise |
| THBS1 | turquoise |
| THEM4 | turquoise |
| THEM6 | turquoise |
| THOC1 | turquoise |
| THOC2 | turquoise |
| THRAP3 | turquoise |
| THRB | turquoise |
| THSD7A | turquoise |
| THTPA | turquoise |
| THUMPD1 | turquoise |
| THUMPD2 | turquoise |
| THUMPD3 | turquoise |
| THUMPD3-AS1 | turquoise |
| TIA1 | turquoise |
| TIAF1 | turquoise |
| TIAL1 | turquoise |
| TIAM1 | turquoise |
| TIAM2 | turquoise |
| TICAM1 | turquoise |
| TIFA | turquoise |
| TIGD2 | turquoise |
| TIGD6 | turquoise |
| TIGD7 | turquoise |
| TIMM10B | turquoise |
| TIMM17B | turquoise |
| TIMM22 | turquoise |
| TIMM23B | turquoise |
| TIMM9P1 | turquoise |
| TIMM9P2 | turquoise |
| TIMP1 | turquoise |
| TINF2 | turquoise |
| TIPARP | turquoise |
| TIRAP | turquoise |
| TKT | turquoise |
| TLDC1 | turquoise |
| TLDC2 | turquoise |
| TLE4 | turquoise |
| TLK1 | turquoise |
| TLK2 | turquoise |
| TLN2 | turquoise |
| TLR1 | turquoise |
| TLR2 | turquoise |
| TLR3 | turquoise |
| TLR6 | turquoise |
| TM2D1 | turquoise |
| TM2D2 | turquoise |
| TM2D3 | turquoise |
| TM4SF1 | turquoise |
| TM4SF18 | turquoise |
| TM7SF2 | turquoise |
| TM9SF1 | turquoise |
| TMA16 | turquoise |
| TMBIM1 | turquoise |
| TMBIM6 | turquoise |
| TMC4 | turquoise |
| TMC5 | turquoise |
| TMC7 | turquoise |
| TMCC1 | turquoise |
| TMED1 | turquoise |
| TMED10 | turquoise |
| TMED10P2 | turquoise |
| TMED11P | turquoise |
| TMED5 | turquoise |
| TMED7 | turquoise |
| TMED8 | turquoise |
| TMED9 | turquoise |
| TMEM101 | turquoise |
| TMEM102 | turquoise |
| TMEM104 | turquoise |
| TMEM106A | turquoise |
| TMEM106B | turquoise |
| TMEM108 | turquoise |
| TMEM109 | turquoise |
| TMEM11 | turquoise |
| TMEM115 | turquoise |
| TMEM116 | turquoise |
| TMEM117 | turquoise |
| TMEM120A | turquoise |
| TMEM121 | turquoise |
| TMEM123 | turquoise |
| TMEM127 | turquoise |
| TMEM129 | turquoise |
| TMEM131 | turquoise |
| TMEM133 | turquoise |
| TMEM135 | turquoise |
| TMEM136 | turquoise |
| TMEM144 | turquoise |
| TMEM147 | turquoise |
| TMEM14E | turquoise |
| TMEM150A | turquoise |
| TMEM154 | turquoise |
| TMEM156 | turquoise |
| TMEM160 | turquoise |
| TMEM161A | turquoise |
| TMEM161B | turquoise |
| TMEM161B-AS1 | turquoise |
| TMEM165 | turquoise |
| TMEM167A | turquoise |
| TMEM167B | turquoise |
| TMEM168 | turquoise |
| TMEM170A | turquoise |
| TMEM170B | turquoise |
| TMEM176A | turquoise |
| TMEM176B | turquoise |
| TMEM179B | turquoise |
| TMEM180 | turquoise |
| TMEM181 | turquoise |
| TMEM184B | turquoise |
| TMEM184C | turquoise |
| TMEM185A | turquoise |
| TMEM187 | turquoise |
| TMEM191A | turquoise |
| TMEM192 | turquoise |
| TMEM194B | turquoise |
| TMEM2 | turquoise |
| TMEM203 | turquoise |
| TMEM205 | turquoise |
| TMEM209 | turquoise |
| TMEM213 | turquoise |
| TMEM214 | turquoise |
| TMEM217 | turquoise |
| TMEM218 | turquoise |
| TMEM219 | turquoise |
| TMEM222 | turquoise |
| TMEM229A | turquoise |
| TMEM233 | turquoise |
| TMEM238 | turquoise |
| TMEM243 | turquoise |
| TMEM245 | turquoise |
| TMEM248 | turquoise |
| TMEM260 | turquoise |
| TMEM263 | turquoise |
| TMEM30A | turquoise |
| TMEM33 | turquoise |
| TMEM35 | turquoise |
| TMEM39A | turquoise |
| TMEM39B | turquoise |
| TMEM41B | turquoise |
| TMEM45A | turquoise |
| TMEM50B | turquoise |
| TMEM51 | turquoise |
| TMEM53 | turquoise |
| TMEM54 | turquoise |
| TMEM55A | turquoise |
| TMEM55B | turquoise |
| TMEM56 | turquoise |
| TMEM57 | turquoise |
| TMEM59 | turquoise |
| TMEM61 | turquoise |
| TMEM64 | turquoise |
| TMEM65 | turquoise |
| TMEM67 | turquoise |
| TMEM71 | turquoise |
| TMEM87B | turquoise |
| TMEM8A | turquoise |
| TMEM92 | turquoise |
| TMEM98 | turquoise |
| TMEM9B-AS1 | turquoise |
| TMF1 | turquoise |
| TMIE | turquoise |
| TMLHE | turquoise |
| TMOD2 | turquoise |
| TMOD3 | turquoise |
| TMPPE | turquoise |
| TMPRSS6 | turquoise |
| TMSB4X | turquoise |
| TMSB4Y | turquoise |
| TMTC2 | turquoise |
| TMTC3 | turquoise |
| TMX1 | turquoise |
| TMX2P1 | turquoise |
| TMX3 | turquoise |
| TMX4 | turquoise |
| TNFAIP8 | turquoise |
| TNFRSF10B | turquoise |
| TNFRSF10D | turquoise |
| TNFRSF11A | turquoise |
| TNFRSF12A | turquoise |
| TNFRSF1A | turquoise |
| TNFSF14 | turquoise |
| TNFSF15 | turquoise |
| TNFSF8 | turquoise |
| TNFSF9 | turquoise |
| TNIK | turquoise |
| TNIP1 | turquoise |
| TNIP2 | turquoise |
| TNIP3 | turquoise |
| TNKS | turquoise |
| TNKS1BP1 | turquoise |
| TNKS2 | turquoise |
| TNPO1 | turquoise |
| TNPO3 | turquoise |
| TNRC6A | turquoise |
| TNRC6B | turquoise |
| TNRC6C | turquoise |
| TNS4 | turquoise |
| TOLLIP | turquoise |
| TOM1 | turquoise |
| TOMM20P2 | turquoise |
| TOMM7 | turquoise |
| TOP2B | turquoise |
| TOPORS | turquoise |
| TOR1A | turquoise |
| TOR1AIP1 | turquoise |
| TOR1AIP2 | turquoise |
| TOR1B | turquoise |
| TOR2A | turquoise |
| TOR4A | turquoise |
| TOX | turquoise |
| TP53 | turquoise |
| TP53BP1 | turquoise |
| TP53BP2 | turquoise |
| TP53I11 | turquoise |
| TP53I3 | turquoise |
| TP53INP1 | turquoise |
| TP53TG1 | turquoise |
| TPGS2 | turquoise |
| TPK1 | turquoise |
| TPM3P9 | turquoise |
| TPP2 | turquoise |
| TPR | turquoise |
| TPRA1 | turquoise |
| TPRG1 | turquoise |
| TPRG1L | turquoise |
| TPRN | turquoise |
| TPST1 | turquoise |
| TPT1-AS1 | turquoise |
| TRA2A | turquoise |
| TRA2B | turquoise |
| TRABD2A | turquoise |
| TRADD | turquoise |
| TRAF3 | turquoise |
| TRAF3IP2-AS1 | turquoise |
| TRAF5 | turquoise |
| TRAF6 | turquoise |
| TRAF7 | turquoise |
| TRAJ1 | turquoise |
| TRAJ10 | turquoise |
| TRAJ12 | turquoise |
| TRAJ13 | turquoise |
| TRAJ14 | turquoise |
| TRAJ16 | turquoise |
| TRAJ17 | turquoise |
| TRAJ18 | turquoise |
| TRAJ2 | turquoise |
| TRAJ21 | turquoise |
| TRAJ3 | turquoise |
| TRAJ31 | turquoise |
| TRAJ37 | turquoise |
| TRAJ38 | turquoise |
| TRAJ39 | turquoise |
| TRAJ5 | turquoise |
| TRAJ6 | turquoise |
| TRAJ8 | turquoise |
| TRAK2 | turquoise |
| TRAM1 | turquoise |
| TRAM1L1 | turquoise |
| TRANK1 | turquoise |
| TRAPPC1 | turquoise |
| TRAPPC10 | turquoise |
| TRAPPC11 | turquoise |
| TRAPPC12-AS1 | turquoise |
| TRAPPC13 | turquoise |
| TRAPPC2 | turquoise |
| TRAPPC4 | turquoise |
| TRAPPC6A | turquoise |
| TRAPPC6B | turquoise |
| TRAPPC8 | turquoise |
| TRAPPC9 | turquoise |
| TRDMT1 | turquoise |
| TREM1 | turquoise |
| TRERF1 | turquoise |
| TRIB1 | turquoise |
| TRIM13 | turquoise |
| TRIM2 | turquoise |
| TRIM23 | turquoise |
| TRIM24 | turquoise |
| TRIM25 | turquoise |
| TRIM26 | turquoise |
| TRIM32 | turquoise |
| TRIM33 | turquoise |
| TRIM36 | turquoise |
| TRIM38 | turquoise |
| TRIM4 | turquoise |
| TRIM44 | turquoise |
| TRIM47 | turquoise |
| TRIM48 | turquoise |
| TRIM5 | turquoise |
| TRIM52 | turquoise |
| TRIM52-AS1 | turquoise |
| TRIM6 | turquoise |
| TRIM60P18 | turquoise |
| TRIM66 | turquoise |
| TRIM8 | turquoise |
| TRIO | turquoise |
| TRIP10 | turquoise |
| TRIP11 | turquoise |
| TRIP12 | turquoise |
| TRIP6 | turquoise |
| TRIQK | turquoise |
| TRMT10A | turquoise |
| TRMT10B | turquoise |
| TRMT11 | turquoise |
| TRMT13 | turquoise |
| TRMT1L | turquoise |
| TRMT2B | turquoise |
| TRMT44 | turquoise |
| TRMT5 | turquoise |
| TRNT1 | turquoise |
| TROVE2 | turquoise |
| TRPC1 | turquoise |
| TRPC6 | turquoise |
| TRPM7 | turquoise |
| TRPM8 | turquoise |
| TRPS1 | turquoise |
| TRRAP | turquoise |
| TSACC | turquoise |
| TSC1 | turquoise |
| TSC22D2 | turquoise |
| TSC22D4 | turquoise |
| TSEN2 | turquoise |
| TSEN34 | turquoise |
| TSGA10 | turquoise |
| TSHZ1 | turquoise |
| TSHZ2 | turquoise |
| TSPAN15 | turquoise |
| TSPAN17 | turquoise |
| TSPAN2 | turquoise |
| TSPAN31 | turquoise |
| TSPAN5 | turquoise |
| TSPO | turquoise |
| TSPYL5 | turquoise |
| TSR2 | turquoise |
| TSR3 | turquoise |
| TSSC4 | turquoise |
| TSSK4 | turquoise |
| TSSK5P | turquoise |
| TST | turquoise |
| TSTD2 | turquoise |
| TSTD3 | turquoise |
| TTBK2 | turquoise |
| TTC1 | turquoise |
| TTC13 | turquoise |
| TTC14 | turquoise |
| TTC17 | turquoise |
| TTC19 | turquoise |
| TTC21B | turquoise |
| TTC23 | turquoise |
| TTC28-AS1 | turquoise |
| TTC3 | turquoise |
| TTC30A | turquoise |
| TTC33 | turquoise |
| TTC37 | turquoise |
| TTC38 | turquoise |
| TTC39B | turquoise |
| TTC3P1 | turquoise |
| TTC5 | turquoise |
| TTC6 | turquoise |
| TTC8 | turquoise |
| TTF1 | turquoise |
| TTF2 | turquoise |
| TTLL11 | turquoise |
| TTLL12 | turquoise |
| TTLL5 | turquoise |
| TTLL7 | turquoise |
| TTPAL | turquoise |
| TTTY15 | turquoise |
| TUBA4A | turquoise |
| TUBB4B | turquoise |
| TUBD1 | turquoise |
| TUBE1 | turquoise |
| TUBGCP2 | turquoise |
| TUBGCP4 | turquoise |
| TUBGCP5 | turquoise |
| TUFM | turquoise |
| TUG1 | turquoise |
| TULP4 | turquoise |
| TUSC1 | turquoise |
| TUSC2 | turquoise |
| TVP23B | turquoise |
| TVP23C | turquoise |
| TWF2 | turquoise |
| TWISTNB | turquoise |
| TWSG1 | turquoise |
| TXK | turquoise |
| TXLNG | turquoise |
| TXLNGY | turquoise |
| TXN | turquoise |
| TXN2 | turquoise |
| TXNDC16 | turquoise |
| TXNP6 | turquoise |
| TXNRD3 | turquoise |
| TYMP | turquoise |
| TYRO3 | turquoise |
| TYRP1 | turquoise |
| TYW1 | turquoise |
| TYW1B | turquoise |
| TYW3 | turquoise |
| TYW5 | turquoise |
| U1 | turquoise |
| U2AF2 | turquoise |
| U2SURP | turquoise |
| U47924.27 | turquoise |
| U47924.6 | turquoise |
| U52111.14 | turquoise |
| UBA1 | turquoise |
| UBA3 | turquoise |
| UBA5 | turquoise |
| UBA52 | turquoise |
| UBA52P5 | turquoise |
| UBA52P8 | turquoise |
| UBA6 | turquoise |
| UBA6-AS1 | turquoise |
| UBAC1 | turquoise |
| UBALD2 | turquoise |
| UBAP1 | turquoise |
| UBAP2 | turquoise |
| UBB | turquoise |
| UBBP4 | turquoise |
| UBC | turquoise |
| UBE2D1 | turquoise |
| UBE2D3 | turquoise |
| UBE2FP1 | turquoise |
| UBE2FP3 | turquoise |
| UBE2G1 | turquoise |
| UBE2G2 | turquoise |
| UBE2H | turquoise |
| UBE2I | turquoise |
| UBE2K | turquoise |
| UBE2L3 | turquoise |
| UBE2M | turquoise |
| UBE2Q1-AS1 | turquoise |
| UBE2Q2P1 | turquoise |
| UBE2Q2P2 | turquoise |
| UBE2V1 | turquoise |
| UBE2W | turquoise |
| UBE3A | turquoise |
| UBE3B | turquoise |
| UBE3C | turquoise |
| UBE3D | turquoise |
| UBE4A | turquoise |
| UBE4B | turquoise |
| UBIAD1 | turquoise |
| UBL7 | turquoise |
| UBLCP1 | turquoise |
| UBN2 | turquoise |
| UBP1 | turquoise |
| UBQLN1 | turquoise |
| UBR1 | turquoise |
| UBR2 | turquoise |
| UBR3 | turquoise |
| UBR4 | turquoise |
| UBR5 | turquoise |
| UBTD1 | turquoise |
| UBTD2 | turquoise |
| UBXN1 | turquoise |
| UBXN2A | turquoise |
| UBXN2B | turquoise |
| UBXN4 | turquoise |
| UBXN6 | turquoise |
| UBXN7 | turquoise |
| UCHL5 | turquoise |
| UCK1 | turquoise |
| UCP2 | turquoise |
| UEVLD | turquoise |
| UFC1 | turquoise |
| UFL1 | turquoise |
| UFM1 | turquoise |
| UFSP2 | turquoise |
| UGCG | turquoise |
| UGDH-AS1 | turquoise |
| UGGT1 | turquoise |
| UGGT2 | turquoise |
| UGP2 | turquoise |
| UGT8 | turquoise |
| UHMK1 | turquoise |
| UHRF1BP1 | turquoise |
| UHRF1BP1L | turquoise |
| UHRF2 | turquoise |
| UIMC1 | turquoise |
| ULBP2 | turquoise |
| ULBP3 | turquoise |
| ULK4 | turquoise |
| UMAD1 | turquoise |
| UNC45A | turquoise |
| UNC5C | turquoise |
| UNC93B1 | turquoise |
| UPF2 | turquoise |
| UPF3A | turquoise |
| UPP1 | turquoise |
| UPRT | turquoise |
| UQCC1 | turquoise |
| UQCR10 | turquoise |
| UQCRBP1 | turquoise |
| UQCRC1 | turquoise |
| URB1 | turquoise |
| URI1 | turquoise |
| URM1 | turquoise |
| USE1 | turquoise |
| USF2 | turquoise |
| USMG5P1 | turquoise |
| USO1 | turquoise |
| USP10 | turquoise |
| USP11 | turquoise |
| USP12 | turquoise |
| USP13 | turquoise |
| USP15 | turquoise |
| USP16 | turquoise |
| USP22 | turquoise |
| USP24 | turquoise |
| USP25 | turquoise |
| USP28 | turquoise |
| USP3 | turquoise |
| USP30 | turquoise |
| USP31 | turquoise |
| USP32 | turquoise |
| USP33 | turquoise |
| USP34 | turquoise |
| USP37 | turquoise |
| USP38 | turquoise |
| USP4 | turquoise |
| USP40 | turquoise |
| USP42 | turquoise |
| USP44 | turquoise |
| USP45 | turquoise |
| USP46 | turquoise |
| USP46-AS1 | turquoise |
| USP47 | turquoise |
| USP48 | turquoise |
| USP49 | turquoise |
| USP5 | turquoise |
| USP53 | turquoise |
| USP6NL | turquoise |
| USP7 | turquoise |
| USP8 | turquoise |
| USP9X | turquoise |
| USP9Y | turquoise |
| USPL1 | turquoise |
| UTP14C | turquoise |
| UTP15 | turquoise |
| UTP20 | turquoise |
| UTP23 | turquoise |
| UTP3 | turquoise |
| UTRN | turquoise |
| UTY | turquoise |
| UVRAG | turquoise |
| UVSSA | turquoise |
| UXS1 | turquoise |
| VAMP4 | turquoise |
| VAMP7 | turquoise |
| VAPA | turquoise |
| VAPB | turquoise |
| VASH2 | turquoise |
| VASN | turquoise |
| VASP | turquoise |
| VAT1 | turquoise |
| VAV3 | turquoise |
| VCL | turquoise |
| VCP | turquoise |
| VCPIP1 | turquoise |
| VCPKMT | turquoise |
| VDAC1P8 | turquoise |
| VEGFB | turquoise |
| VEGFC | turquoise |
| VEZF1 | turquoise |
| VEZT | turquoise |
| VGLL1 | turquoise |
| VGLL3 | turquoise |
| VGLL4 | turquoise |
| VHL | turquoise |
| VIM-AS1 | turquoise |
| VKORC1 | turquoise |
| VKORC1L1 | turquoise |
| VMA21 | turquoise |
| VMO1 | turquoise |
| VMP1 | turquoise |
| VN1R1 | turquoise |
| VN1R81P | turquoise |
| VN1R83P | turquoise |
| VPRBP | turquoise |
| VPS11 | turquoise |
| VPS13A | turquoise |
| VPS13B | turquoise |
| VPS13C | turquoise |
| VPS13D | turquoise |
| VPS18 | turquoise |
| VPS25 | turquoise |
| VPS28 | turquoise |
| VPS35 | turquoise |
| VPS36 | turquoise |
| VPS37A | turquoise |
| VPS37B | turquoise |
| VPS41 | turquoise |
| VPS45 | turquoise |
| VPS4A | turquoise |
| VPS4B | turquoise |
| VPS51 | turquoise |
| VPS53 | turquoise |
| VPS54 | turquoise |
| VPS8 | turquoise |
| VRK2 | turquoise |
| VTCN1 | turquoise |
| VTI1A | turquoise |
| VTRNA1-1 | turquoise |
| VWA1 | turquoise |
| VWA2 | turquoise |
| VWA8 | turquoise |
| VWDE | turquoise |
| WAC | turquoise |
| WAPAL | turquoise |
| WARS2 | turquoise |
| WASL | turquoise |
| WBP1LP2 | turquoise |
| WBP2 | turquoise |
| WBP5 | turquoise |
| WDFY1 | turquoise |
| WDFY2 | turquoise |
| WDFY3 | turquoise |
| WDFY3-AS2 | turquoise |
| WDR1 | turquoise |
| WDR11 | turquoise |
| WDR13 | turquoise |
| WDR18 | turquoise |
| WDR20 | turquoise |
| WDR26 | turquoise |
| WDR27 | turquoise |
| WDR33 | turquoise |
| WDR34 | turquoise |
| WDR35 | turquoise |
| WDR36 | turquoise |
| WDR37 | turquoise |
| WDR41 | turquoise |
| WDR47 | turquoise |
| WDR48 | turquoise |
| WDR5B | turquoise |
| WDR60 | turquoise |
| WDR7 | turquoise |
| WDR70 | turquoise |
| WDR73 | turquoise |
| WDR82 | turquoise |
| WDR83OS | turquoise |
| WDR88 | turquoise |
| WDR89 | turquoise |
| WDSUB1 | turquoise |
| WDTC1 | turquoise |
| WEE1 | turquoise |
| WFDC2 | turquoise |
| WHAMM | turquoise |
| WHSC1L1 | turquoise |
| WI2-87327B8.2 | turquoise |
| WIPF2 | turquoise |
| WISP1 | turquoise |
| WLS | turquoise |
| WNK1 | turquoise |
| WNT10A | turquoise |
| WNT16 | turquoise |
| WNT5A | turquoise |
| WNT5A-AS1 | turquoise |
| WNT7B | turquoise |
| WRN | turquoise |
| WSB1 | turquoise |
| WTAP | turquoise |
| WWP1 | turquoise |
| XAB2 | turquoise |
| XAF1 | turquoise |
| XAGE2B | turquoise |
| XIAP | turquoise |
| XIST | turquoise |
| XK | turquoise |
| XKR8 | turquoise |
| XKR9 | turquoise |
| XKRX | turquoise |
| XPA | turquoise |
| XPNPEP3 | turquoise |
| XPO4 | turquoise |
| XPO7 | turquoise |
| XPR1 | turquoise |
| XRCC1 | turquoise |
| XRCC6 | turquoise |
| XRN1 | turquoise |
| XRN2 | turquoise |
| XRRA1 | turquoise |
| XXbac-BPG248L24.12 | turquoise |
| XXbac-BPG283O16.9 | turquoise |
| XXbac-BPG299F13.14 | turquoise |
| XXbac-BPG55C20.7 | turquoise |
| XXyac-YX65C7_A.2 | turquoise |
| XYLB | turquoise |
| XYLT1 | turquoise |
| YAF2 | turquoise |
| YAP1 | turquoise |
| YBX1 | turquoise |
| YBX3 | turquoise |
| YES1 | turquoise |
| YIF1B | turquoise |
| YIPF3 | turquoise |
| YIPF4 | turquoise |
| YIPF6 | turquoise |
| YLPM1 | turquoise |
| YOD1 | turquoise |
| YPEL1 | turquoise |
| YPEL2 | turquoise |
| YTHDC1 | turquoise |
| YTHDC2 | turquoise |
| YTHDF3 | turquoise |
| YWHAE | turquoise |
| YWHAH | turquoise |
| YY1 | turquoise |
| Z83826.1 | turquoise |
| Z98750.1 | turquoise |
| ZADH2 | turquoise |
| ZBED1 | turquoise |
| ZBED3 | turquoise |
| ZBED4 | turquoise |
| ZBED5 | turquoise |
| ZBED6 | turquoise |
| ZBED8 | turquoise |
| ZBED9 | turquoise |
| ZBTB1 | turquoise |
| ZBTB10 | turquoise |
| ZBTB11 | turquoise |
| ZBTB11-AS1 | turquoise |
| ZBTB14 | turquoise |
| ZBTB16 | turquoise |
| ZBTB18 | turquoise |
| ZBTB2 | turquoise |
| ZBTB20 | turquoise |
| ZBTB21 | turquoise |
| ZBTB22 | turquoise |
| ZBTB24 | turquoise |
| ZBTB25 | turquoise |
| ZBTB26 | turquoise |
| ZBTB3 | turquoise |
| ZBTB33 | turquoise |
| ZBTB34 | turquoise |
| ZBTB37 | turquoise |
| ZBTB38 | turquoise |
| ZBTB39 | turquoise |
| ZBTB40 | turquoise |
| ZBTB41 | turquoise |
| ZBTB42 | turquoise |
| ZBTB43 | turquoise |
| ZBTB44 | turquoise |
| ZBTB45 | turquoise |
| ZBTB49 | turquoise |
| ZBTB5 | turquoise |
| ZBTB6 | turquoise |
| ZBTB8A | turquoise |
| ZC2HC1A | turquoise |
| ZC3H10 | turquoise |
| ZC3H11A | turquoise |
| ZC3H12C | turquoise |
| ZC3H12D | turquoise |
| ZC3H13 | turquoise |
| ZC3H14 | turquoise |
| ZC3H6 | turquoise |
| ZC3H7A | turquoise |
| ZC3H8 | turquoise |
| ZC3HAV1 | turquoise |
| ZC3HAV1L | turquoise |
| ZCCHC11 | turquoise |
| ZCCHC12 | turquoise |
| ZCCHC14 | turquoise |
| ZCCHC2 | turquoise |
| ZCCHC4 | turquoise |
| ZCCHC6 | turquoise |
| ZCCHC7 | turquoise |
| ZCCHC8 | turquoise |
| ZCCHC9 | turquoise |
| ZDBF2 | turquoise |
| ZDHHC12 | turquoise |
| ZDHHC13 | turquoise |
| ZDHHC17 | turquoise |
| ZDHHC2 | turquoise |
| ZDHHC20 | turquoise |
| ZDHHC21 | turquoise |
| ZDHHC23 | turquoise |
| ZDHHC24 | turquoise |
| ZDHHC6 | turquoise |
| ZDHHC8P1 | turquoise |
| ZEB1 | turquoise |
| ZEB1-AS1 | turquoise |
| ZEB2 | turquoise |
| ZER1 | turquoise |
| ZFAND1 | turquoise |
| ZFAND2B | turquoise |
| ZFAND4 | turquoise |
| ZFAND5 | turquoise |
| ZFAND6 | turquoise |
| ZFAT | turquoise |
| ZFC3H1 | turquoise |
| ZFHX3 | turquoise |
| ZFP1 | turquoise |
| ZFP14 | turquoise |
| ZFP2 | turquoise |
| ZFP28 | turquoise |
| ZFP3 | turquoise |
| ZFP30 | turquoise |
| ZFP36L1 | turquoise |
| ZFP36L2 | turquoise |
| ZFP62 | turquoise |
| ZFP69 | turquoise |
| ZFP69B | turquoise |
| ZFP82 | turquoise |
| ZFP90 | turquoise |
| ZFP91 | turquoise |
| ZFPM2 | turquoise |
| ZFPM2-AS1 | turquoise |
| ZFR | turquoise |
| ZFX | turquoise |
| ZFY | turquoise |
| ZFYVE16 | turquoise |
| ZFYVE21 | turquoise |
| ZFYVE26 | turquoise |
| ZGRF1 | turquoise |
| ZHX1 | turquoise |
| ZHX1-C8orf76 | turquoise |
| ZHX3 | turquoise |
| ZIK1 | turquoise |
| ZKSCAN1 | turquoise |
| ZKSCAN2 | turquoise |
| ZKSCAN3 | turquoise |
| ZKSCAN4 | turquoise |
| ZKSCAN5 | turquoise |
| ZKSCAN7 | turquoise |
| ZKSCAN8 | turquoise |
| ZMAT1 | turquoise |
| ZMAT2 | turquoise |
| ZMAT3 | turquoise |
| ZMAT5 | turquoise |
| ZMPSTE24 | turquoise |
| ZMYM1 | turquoise |
| ZMYM2 | turquoise |
| ZMYM4 | turquoise |
| ZMYM5 | turquoise |
| ZMYM6 | turquoise |
| ZMYM6NB | turquoise |
| ZMYND11 | turquoise |
| ZMYND8 | turquoise |
| ZNF10 | turquoise |
| ZNF100 | turquoise |
| ZNF101 | turquoise |
| ZNF107 | turquoise |
| ZNF112 | turquoise |
| ZNF114 | turquoise |
| ZNF117 | turquoise |
| ZNF12 | turquoise |
| ZNF121 | turquoise |
| ZNF124 | turquoise |
| ZNF131 | turquoise |
| ZNF132 | turquoise |
| ZNF134 | turquoise |
| ZNF135 | turquoise |
| ZNF136 | turquoise |
| ZNF137P | turquoise |
| ZNF138 | turquoise |
| ZNF14 | turquoise |
| ZNF140 | turquoise |
| ZNF141 | turquoise |
| ZNF143 | turquoise |
| ZNF148 | turquoise |
| ZNF154 | turquoise |
| ZNF155 | turquoise |
| ZNF160 | turquoise |
| ZNF169 | turquoise |
| ZNF17 | turquoise |
| ZNF175 | turquoise |
| ZNF180 | turquoise |
| ZNF181 | turquoise |
| ZNF182 | turquoise |
| ZNF184 | turquoise |
| ZNF185 | turquoise |
| ZNF189 | turquoise |
| ZNF19 | turquoise |
| ZNF195 | turquoise |
| ZNF197 | turquoise |
| ZNF2 | turquoise |
| ZNF200 | turquoise |
| ZNF202 | turquoise |
| ZNF207 | turquoise |
| ZNF211 | turquoise |
| ZNF213 | turquoise |
| ZNF214 | turquoise |
| ZNF215 | turquoise |
| ZNF217 | turquoise |
| ZNF219 | turquoise |
| ZNF22 | turquoise |
| ZNF222 | turquoise |
| ZNF224 | turquoise |
| ZNF225 | turquoise |
| ZNF226 | turquoise |
| ZNF227 | turquoise |
| ZNF229 | turquoise |
| ZNF230 | turquoise |
| ZNF234 | turquoise |
| ZNF235 | turquoise |
| ZNF236 | turquoise |
| ZNF24 | turquoise |
| ZNF248 | turquoise |
| ZNF25 | turquoise |
| ZNF250 | turquoise |
| ZNF252P | turquoise |
| ZNF253 | turquoise |
| ZNF254 | turquoise |
| ZNF256 | turquoise |
| ZNF26 | turquoise |
| ZNF260 | turquoise |
| ZNF264 | turquoise |
| ZNF266 | turquoise |
| ZNF267 | turquoise |
| ZNF268 | turquoise |
| ZNF271P | turquoise |
| ZNF273 | turquoise |
| ZNF274 | turquoise |
| ZNF275 | turquoise |
| ZNF277 | turquoise |
| ZNF28 | turquoise |
| ZNF280C | turquoise |
| ZNF280D | turquoise |
| ZNF281 | turquoise |
| ZNF283 | turquoise |
| ZNF284 | turquoise |
| ZNF287 | turquoise |
| ZNF292 | turquoise |
| ZNF30 | turquoise |
| ZNF300 | turquoise |
| ZNF302 | turquoise |
| ZNF304 | turquoise |
| ZNF311 | turquoise |
| ZNF317 | turquoise |
| ZNF318 | turquoise |
| ZNF319 | turquoise |
| ZNF32-AS2 | turquoise |
| ZNF320 | turquoise |
| ZNF322 | turquoise |
| ZNF324 | turquoise |
| ZNF326 | turquoise |
| ZNF329 | turquoise |
| ZNF333 | turquoise |
| ZNF334 | turquoise |
| ZNF33A | turquoise |
| ZNF33B | turquoise |
| ZNF343 | turquoise |
| ZNF345 | turquoise |
| ZNF346 | turquoise |
| ZNF347 | turquoise |
| ZNF35 | turquoise |
| ZNF350 | turquoise |
| ZNF350-AS1 | turquoise |
| ZNF354A | turquoise |
| ZNF354B | turquoise |
| ZNF354C | turquoise |
| ZNF358 | turquoise |
| ZNF37A | turquoise |
| ZNF37BP | turquoise |
| ZNF383 | turquoise |
| ZNF385A | turquoise |
| ZNF391 | turquoise |
| ZNF394 | turquoise |
| ZNF396 | turquoise |
| ZNF397 | turquoise |
| ZNF398 | turquoise |
| ZNF404 | turquoise |
| ZNF407 | turquoise |
| ZNF408 | turquoise |
| ZNF41 | turquoise |
| ZNF415 | turquoise |
| ZNF416 | turquoise |
| ZNF417 | turquoise |
| ZNF418 | turquoise |
| ZNF419 | turquoise |
| ZNF420 | turquoise |
| ZNF425 | turquoise |
| ZNF426 | turquoise |
| ZNF428 | turquoise |
| ZNF429 | turquoise |
| ZNF43 | turquoise |
| ZNF430 | turquoise |
| ZNF431 | turquoise |
| ZNF432 | turquoise |
| ZNF433 | turquoise |
| ZNF436 | turquoise |
| ZNF439 | turquoise |
| ZNF44 | turquoise |
| ZNF441 | turquoise |
| ZNF443 | turquoise |
| ZNF445 | turquoise |
| ZNF449 | turquoise |
| ZNF45 | turquoise |
| ZNF451 | turquoise |
| ZNF460 | turquoise |
| ZNF461 | turquoise |
| ZNF462 | turquoise |
| ZNF467 | turquoise |
| ZNF468 | turquoise |
| ZNF470 | turquoise |
| ZNF471 | turquoise |
| ZNF480 | turquoise |
| ZNF484 | turquoise |
| ZNF485 | turquoise |
| ZNF486 | turquoise |
| ZNF487 | turquoise |
| ZNF491 | turquoise |
| ZNF493 | turquoise |
| ZNF496 | turquoise |
| ZNF501 | turquoise |
| ZNF502 | turquoise |
| ZNF503 | turquoise |
| ZNF503-AS1 | turquoise |
| ZNF503-AS2 | turquoise |
| ZNF506 | turquoise |
| ZNF507 | turquoise |
| ZNF510 | turquoise |
| ZNF512 | turquoise |
| ZNF514 | turquoise |
| ZNF516 | turquoise |
| ZNF518A | turquoise |
| ZNF518B | turquoise |
| ZNF524 | turquoise |
| ZNF525 | turquoise |
| ZNF527 | turquoise |
| ZNF528 | turquoise |
| ZNF529 | turquoise |
| ZNF529-AS1 | turquoise |
| ZNF530 | turquoise |
| ZNF532 | turquoise |
| ZNF540 | turquoise |
| ZNF542P | turquoise |
| ZNF543 | turquoise |
| ZNF544 | turquoise |
| ZNF546 | turquoise |
| ZNF547 | turquoise |
| ZNF548 | turquoise |
| ZNF549 | turquoise |
| ZNF550 | turquoise |
| ZNF551 | turquoise |
| ZNF552 | turquoise |
| ZNF554 | turquoise |
| ZNF555 | turquoise |
| ZNF557 | turquoise |
| ZNF558 | turquoise |
| ZNF559 | turquoise |
| ZNF561 | turquoise |
| ZNF562 | turquoise |
| ZNF563 | turquoise |
| ZNF565 | turquoise |
| ZNF566 | turquoise |
| ZNF567 | turquoise |
| ZNF568 | turquoise |
| ZNF569 | turquoise |
| ZNF57 | turquoise |
| ZNF570 | turquoise |
| ZNF571 | turquoise |
| ZNF572 | turquoise |
| ZNF573 | turquoise |
| ZNF574 | turquoise |
| ZNF575 | turquoise |
| ZNF577 | turquoise |
| ZNF582 | turquoise |
| ZNF584 | turquoise |
| ZNF585A | turquoise |
| ZNF585B | turquoise |
| ZNF586 | turquoise |
| ZNF587 | turquoise |
| ZNF587B | turquoise |
| ZNF589 | turquoise |
| ZNF594 | turquoise |
| ZNF595 | turquoise |
| ZNF596 | turquoise |
| ZNF597 | turquoise |
| ZNF599 | turquoise |
| ZNF600 | turquoise |
| ZNF605 | turquoise |
| ZNF606 | turquoise |
| ZNF607 | turquoise |
| ZNF608 | turquoise |
| ZNF609 | turquoise |
| ZNF610 | turquoise |
| ZNF611 | turquoise |
| ZNF613 | turquoise |
| ZNF614 | turquoise |
| ZNF615 | turquoise |
| ZNF616 | turquoise |
| ZNF618 | turquoise |
| ZNF619 | turquoise |
| ZNF620 | turquoise |
| ZNF621 | turquoise |
| ZNF622 | turquoise |
| ZNF623 | turquoise |
| ZNF624 | turquoise |
| ZNF626 | turquoise |
| ZNF628 | turquoise |
| ZNF630 | turquoise |
| ZNF638 | turquoise |
| ZNF638-IT1 | turquoise |
| ZNF641 | turquoise |
| ZNF644 | turquoise |
| ZNF649 | turquoise |
| ZNF652 | turquoise |
| ZNF654 | turquoise |
| ZNF655 | turquoise |
| ZNF66 | turquoise |
| ZNF662 | turquoise |
| ZNF664 | turquoise |
| ZNF669 | turquoise |
| ZNF670 | turquoise |
| ZNF671 | turquoise |
| ZNF672 | turquoise |
| ZNF674 | turquoise |
| ZNF675 | turquoise |
| ZNF677 | turquoise |
| ZNF678 | turquoise |
| ZNF680 | turquoise |
| ZNF681 | turquoise |
| ZNF682 | turquoise |
| ZNF684 | turquoise |
| ZNF688 | turquoise |
| ZNF689 | turquoise |
| ZNF69 | turquoise |
| ZNF697 | turquoise |
| ZNF699 | turquoise |
| ZNF7 | turquoise |
| ZNF70 | turquoise |
| ZNF700 | turquoise |
| ZNF701 | turquoise |
| ZNF702P | turquoise |
| ZNF704 | turquoise |
| ZNF706 | turquoise |
| ZNF708 | turquoise |
| ZNF711 | turquoise |
| ZNF714 | turquoise |
| ZNF717 | turquoise |
| ZNF718 | turquoise |
| ZNF720 | turquoise |
| ZNF721 | turquoise |
| ZNF724P | turquoise |
| ZNF726 | turquoise |
| ZNF736 | turquoise |
| ZNF737 | turquoise |
| ZNF738 | turquoise |
| ZNF740 | turquoise |
| ZNF746 | turquoise |
| ZNF749 | turquoise |
| ZNF75A | turquoise |
| ZNF75D | turquoise |
| ZNF761 | turquoise |
| ZNF763 | turquoise |
| ZNF765 | turquoise |
| ZNF766 | turquoise |
| ZNF77 | turquoise |
| ZNF770 | turquoise |
| ZNF772 | turquoise |
| ZNF773 | turquoise |
| ZNF774 | turquoise |
| ZNF776 | turquoise |
| ZNF777 | turquoise |
| ZNF778 | turquoise |
| ZNF780A | turquoise |
| ZNF780B | turquoise |
| ZNF782 | turquoise |
| ZNF787 | turquoise |
| ZNF788 | turquoise |
| ZNF789 | turquoise |
| ZNF79 | turquoise |
| ZNF790 | turquoise |
| ZNF790-AS1 | turquoise |
| ZNF791 | turquoise |
| ZNF792 | turquoise |
| ZNF793 | turquoise |
| ZNF799 | turquoise |
| ZNF8 | turquoise |
| ZNF800 | turquoise |
| ZNF805 | turquoise |
| ZNF808 | turquoise |
| ZNF81 | turquoise |
| ZNF812 | turquoise |
| ZNF813 | turquoise |
| ZNF814 | turquoise |
| ZNF816 | turquoise |
| ZNF816-ZNF321P | turquoise |
| ZNF818P | turquoise |
| ZNF823 | turquoise |
| ZNF826P | turquoise |
| ZNF827 | turquoise |
| ZNF829 | turquoise |
| ZNF83 | turquoise |
| ZNF836 | turquoise |
| ZNF84 | turquoise |
| ZNF841 | turquoise |
| ZNF844 | turquoise |
| ZNF845 | turquoise |
| ZNF846 | turquoise |
| ZNF85 | turquoise |
| ZNF850 | turquoise |
| ZNF852 | turquoise |
| ZNF860 | turquoise |
| ZNF865 | turquoise |
| ZNF879 | turquoise |
| ZNF880 | turquoise |
| ZNF883 | turquoise |
| ZNF888 | turquoise |
| ZNF90 | turquoise |
| ZNF91 | turquoise |
| ZNF92 | turquoise |
| ZNF93 | turquoise |
| ZNHIT6 | turquoise |
| ZNRF2 | turquoise |
| ZNRF2P1 | turquoise |
| ZRANB1 | turquoise |
| ZRANB2 | turquoise |
| ZRANB3 | turquoise |
| ZSCAN12 | turquoise |
| ZSCAN12P1 | turquoise |
| ZSCAN20 | turquoise |
| ZSCAN22 | turquoise |
| ZSCAN25 | turquoise |
| ZSCAN26 | turquoise |
| ZSCAN29 | turquoise |
| ZSCAN30 | turquoise |
| ZSCAN31 | turquoise |
| ZSCAN32 | turquoise |
| ZSCAN5A | turquoise |
| ZSCAN9 | turquoise |
| ZSWIM5 | turquoise |
| ZSWIM6 | turquoise |
| ZSWIM7 | turquoise |
| ZUFSP | turquoise |
| ZXDA | turquoise |
| ZXDB | turquoise |
| ZXDC | turquoise |
| ZYG11A | turquoise |
| ZYG11B | turquoise |
| ZZEF1 | turquoise |
| ZZZ3 | turquoise |

Supplementary Table 3

| probes | moduleColor |
| --- | --- |
| AADAT | red |
| AATBC | red |
| ABCC3 | red |
| ABCC6 | red |
| ABCC6P1 | red |
| ABCD4 | red |
| AC002310.12 | red |
| AC004000.1 | red |
| AC004932.1 | red |
| AC007383.3 | red |
| AC008391.1 | red |
| AC009133.12 | red |
| AC009299.5 | red |
| AC024937.1 | red |
| AC024995.1 | red |
| AC068134.10 | red |
| AC068580.5 | red |
| AC072052.7 | red |
| AC074117.10 | red |
| AC079145.4 | red |
| AC079807.2 | red |
| AC079922.3 | red |
| AC091849.1 | red |
| AC092071.1 | red |
| AC142472.6 | red |
| ACAD10 | red |
| ACAD8 | red |
| ACCS | red |
| ACSF3 | red |
| ACSS1 | red |
| ADAMTS10 | red |
| ADCY6 | red |
| ADHFE1 | red |
| ADIRF-AS1 | red |
| AF001548.5 | red |
| AF064858.11 | red |
| AFG3L1P | red |
| AGAP10 | red |
| AGAP6 | red |
| AHSA2 | red |
| AKR7L | red |
| AL590226.1 | red |
| ALKBH6 | red |
| ALS2CL | red |
| AMFR | red |
| AMT | red |
| AMZ2P1 | red |
| ANKRD34A | red |
| ANKS6 | red |
| ANXA5 | red |
| AP000347.4 | red |
| AP001062.7 | red |
| AP001462.6 | red |
| AP006285.2 | red |
| AP006621.9 | red |
| AP4B1 | red |
| APBB3 | red |
| AQP7 | red |
| ARGLU1 | red |
| ARHGEF11 | red |
| ARHGEF2 | red |
| ARMC9 | red |
| ASB16 | red |
| ATG16L2 | red |
| ATHL1 | red |
| ATP6AP2 | red |
| AZIN2 | red |
| B3GAT1 | red |
| B3GNTL1 | red |
| BACE1-AS | red |
| BAMBI | red |
| BCRP3 | red |
| BMS1P20 | red |
| BPGM | red |
| BRD8 | red |
| BRD9P2 | red |
| BTN2A1 | red |
| BZRAP1 | red |
| C11orf71 | red |
| C19orf18 | red |
| C19orf71 | red |
| C1orf204 | red |
| C22orf29 | red |
| C3orf62 | red |
| C5orf45 | red |
| CAMK1D | red |
| CAMK2G | red |
| CAP1 | red |
| CAPN10-AS1 | red |
| CAPN8 | red |
| CCDC183-AS1 | red |
| CCDC84 | red |
| CCT8P1 | red |
| CDRT15P1 | red |
| CEP164 | red |
| CES4A | red |
| CFL1P5 | red |
| CHDH | red |
| CHKA | red |
| CHMP4A | red |
| CICP14 | red |
| CIRBP | red |
| CLCN6 | red |
| CLK3 | red |
| CLUHP3 | red |
| CMB9-55F22.1 | red |
| CNTNAP1 | red |
| COG8 | red |
| COL27A1 | red |
| COQ10A | red |
| COX19 | red |
| CPSF7 | red |
| CRAMP1L | red |
| CROCCP2 | red |
| CRYGS | red |
| CSAD | red |
| CSPG4P8 | red |
| CTA-14H9.5 | red |
| CTA-204B4.2 | red |
| CTA-223H9.9 | red |
| CTA-228A9.3 | red |
| CTA-38K21.4 | red |
| CTA-984G1.5 | red |
| CTB-129P6.11 | red |
| CTB-31O20.2 | red |
| CTB-31O20.4 | red |
| CTB-39G8.3 | red |
| CTB-43E15.2 | red |
| CTB-50L17.8 | red |
| CTB-50L17.9 | red |
| CTBP1-AS | red |
| CTC-1337H24.4 | red |
| CTC-241N9.1 | red |
| CTC-429P9.3 | red |
| CTC-435M10.12 | red |
| CTC-510F12.2 | red |
| CTC-524C5.2 | red |
| CTC1 | red |
| CTD-2007H13.3 | red |
| CTD-2013N17.6 | red |
| CTD-2017F17.2 | red |
| CTD-2020K17.1 | red |
| CTD-2026K11.6 | red |
| CTD-2095E4.5 | red |
| CTD-2124B8.2 | red |
| CTD-2203K17.1 | red |
| CTD-2227E11.1 | red |
| CTD-2287O16.5 | red |
| CTD-2319I12.4 | red |
| CTD-2527I21.14 | red |
| CTD-2561B21.11 | red |
| CTD-2575K13.6 | red |
| CTD-2583A14.11 | red |
| CTD-2619J13.14 | red |
| CTD-2619J13.9 | red |
| CTD-3035K23.7 | red |
| CTD-3099C6.11 | red |
| CTD-3128G10.6 | red |
| CTD-3195I5.3 | red |
| CTD-3252C9.4 | red |
| CUL9 | red |
| CWC25 | red |
| CYB561D1 | red |
| DCAF8 | red |
| DDB2 | red |
| DDTL | red |
| DDX39B | red |
| DFFB | red |
| DGKA | red |
| DGKD | red |
| DICER1-AS1 | red |
| DIRAS3 | red |
| DNASE1 | red |
| DOC2A | red |
| DOCK9-AS2 | red |
| DTX4 | red |
| DUSP8P5 | red |
| DYNC1I2 | red |
| ECHDC2 | red |
| EFCAB2 | red |
| EGLN2 | red |
| EIF3J-AS1 | red |
| EIF4HP2 | red |
| ELMO2 | red |
| ELMOD3 | red |
| ENDOV | red |
| EPN2 | red |
| ERCC5 | red |
| EWSR1 | red |
| EXT2 | red |
| EZH1 | red |
| FAM160A2 | red |
| FAM201A | red |
| FAM20A | red |
| FAM219B | red |
| FAM229A | red |
| FAM86B3P | red |
| FBXL19-AS1 | red |
| FER1L4 | red |
| FEZF1 | red |
| FEZF1-AS1 | red |
| FLCN | red |
| FUS | red |
| GABBR1 | red |
| GAGE2A | red |
| GIPR | red |
| GLB1L3 | red |
| GOLGA2P5 | red |
| GOLGA8B | red |
| GRB14 | red |
| GS1-124K5.11 | red |
| GSDMB | red |
| GSTM2 | red |
| GTF2IRD2B | red |
| GUCA1B | red |
| H1FX-AS1 | red |
| HCG4P3 | red |
| HEMK1 | red |
| HERC2P2 | red |
| HINFP | red |
| HMGN2P41 | red |
| HMGXB3 | red |
| HOXB-AS1 | red |
| HOXB3 | red |
| HOXD1 | red |
| HSD17B1P1 | red |
| HSD17B7P2 | red |
| HSPA7 | red |
| HSPA8 | red |
| IKZF4 | red |
| INCA1 | red |
| INTS3 | red |
| IQCC | red |
| IRF9 | red |
| IRX2 | red |
| IST1 | red |
| ITFG2 | red |
| ITGB2-AS1 | red |
| JMJD7-PLA2G4B | red |
| KCNC3 | red |
| KIAA0319L | red |
| KIAA0907 | red |
| KRT16P1 | red |
| KSR1 | red |
| L3MBTL1 | red |
| LA16c-316G12.2 | red |
| LA16c-358B7.3 | red |
| LA16c-390E6.4 | red |
| LA16c-OS12.2 | red |
| LBHD1 | red |
| LCAT | red |
| LDLRAD2 | red |
| LENG8-AS1 | red |
| LETMD1 | red |
| LINC00106 | red |
| LINC00115 | red |
| LINC00174 | red |
| LINC00672 | red |
| LINC00685 | red |
| LINC00893 | red |
| LINC00899 | red |
| LINC00910 | red |
| LINC00982 | red |
| LINC01011 | red |
| LINC01024 | red |
| LINC01089 | red |
| LINC01176 | red |
| LINC01372 | red |
| LL22NC03-2H8.4 | red |
| LL22NC03-75H12.2 | red |
| LMBR1L | red |
| LRP5L | red |
| LTB4R | red |
| LUC7L | red |
| LUC7L3 | red |
| LY6G5B | red |
| LYG1 | red |
| MAMDC4 | red |
| MAN2A2 | red |
| MAP2K1 | red |
| MAP3K14-AS1 | red |
| MAU2 | red |
| MEG3 | red |
| METAP1D | red |
| METTL21B | red |
| METTL3 | red |
| MIR1249 | red |
| MIR148A | red |
| MIR200A | red |
| MIR200B | red |
| MIR3176 | red |
| MIR3189 | red |
| MIR331 | red |
| MIR34A | red |
| MIR3653 | red |
| MIR3685 | red |
| MIR4258 | red |
| MIR4292 | red |
| MIR4326 | red |
| MIR4697HG | red |
| MIR4701 | red |
| MIR4728 | red |
| MIR4786 | red |
| MIR600HG | red |
| MIR647 | red |
| MIR6753 | red |
| MIR6772 | red |
| MIR6835 | red |
| MIR6859-3 | red |
| MIR762HG | red |
| MLLT4-AS1 | red |
| MLLT6 | red |
| MLXIP | red |
| MMS19 | red |
| MPP3 | red |
| MRI1 | red |
| MRPS25 | red |
| MSH5 | red |
| MST1L | red |
| MT-TL1 | red |
| MTG1 | red |
| MTHFR | red |
| MTHFSD | red |
| MTMR9LP | red |
| MUM1 | red |
| MYO15B | red |
| MZF1 | red |
| MZF1-AS1 | red |
| NAMPTP1 | red |
| NBR2 | red |
| NEIL1 | red |
| NFATC2IP | red |
| NICN1 | red |
| NISCH | red |
| NLRP1 | red |
| NPHP4 | red |
| NPPA-AS1 | red |
| NRBP2 | red |
| NRIP3 | red |
| NSMCE4A | red |
| OBSCN | red |
| ORAOV1 | red |
| ORAOV1P1 | red |
| OVGP1 | red |
| PAGE2B | red |
| PAN2 | red |
| PASK | red |
| PBX4 | red |
| PCYOX1L | red |
| PDCL3P4 | red |
| PGBD2 | red |
| PGLYRP4 | red |
| PGPEP1 | red |
| PGS1 | red |
| PHC1 | red |
| PHF12 | red |
| PHF7 | red |
| PHKA2 | red |
| PILRB | red |
| PITPNM2 | red |
| PKD1P6 | red |
| PLIN5 | red |
| PLXNB1 | red |
| PNMA3 | red |
| PNN | red |
| POLG2 | red |
| POLR2J4 | red |
| POU5F1 | red |
| POU6F1 | red |
| PPFIA4 | red |
| PPFIBP2 | red |
| PPIP5K1 | red |
| PPP1R13B | red |
| PPP1R26-AS1 | red |
| PPP1R3E | red |
| PRKAB1 | red |
| PROCA1 | red |
| PRPF3 | red |
| PSD4 | red |
| PSORS1C1 | red |
| PTOV1-AS1 | red |
| PYROXD2 | red |
| QRICH2 | red |
| RAD51-AS1 | red |
| RBM5 | red |
| RBM6 | red |
| RN7SL268P | red |
| RN7SL574P | red |
| RNA5SP122 | red |
| RNA5SP203 | red |
| RNA5SP383 | red |
| RNF207 | red |
| RNF41 | red |
| RNU5B-2P | red |
| RNU6-30P | red |
| RNU6-516P | red |
| RNY3P16 | red |
| RNY4P10 | red |
| RP1-102E24.8 | red |
| RP1-151F17.1 | red |
| RP1-178F15.4 | red |
| RP1-179N16.6 | red |
| RP1-20C7.6 | red |
| RP1-267D11.6 | red |
| RP1-315G1.3 | red |
| RP1-39G22.7 | red |
| RP1-59D14.5 | red |
| RP1-63M2.7 | red |
| RP11-1055B8.4 | red |
| RP11-122K13.12 | red |
| RP11-1275H24.2 | red |
| RP11-130L8.2 | red |
| RP11-133K1.12 | red |
| RP11-1391J7.1 | red |
| RP11-143N13.3 | red |
| RP11-155O18.6 | red |
| RP11-156K13.3 | red |
| RP11-157E21.1 | red |
| RP11-158L12.4 | red |
| RP11-159D12.6 | red |
| RP11-164J13.1 | red |
| RP11-164P12.5 | red |
| RP11-167B3.2 | red |
| RP11-167B3.3 | red |
| RP11-181G12.2 | red |
| RP11-182L21.6 | red |
| RP11-192H23.8 | red |
| RP11-196G11.2 | red |
| RP11-206P5.2 | red |
| RP11-20I23.2 | red |
| RP11-212P7.2 | red |
| RP11-214N9.1 | red |
| RP11-226L15.5 | red |
| RP11-228B15.4 | red |
| RP11-244O19.1 | red |
| RP11-245P10.4 | red |
| RP11-254F7.2 | red |
| RP11-261P9.4 | red |
| RP11-264B17.2 | red |
| RP11-266K4.9 | red |
| RP11-274B21.2 | red |
| RP11-285E9.6 | red |
| RP11-285F7.2 | red |
| RP11-295G24.5 | red |
| RP11-2B6.3 | red |
| RP11-302F12.3 | red |
| RP11-304L19.11 | red |
| RP11-326I11.3 | red |
| RP11-329B9.3 | red |
| RP11-329L6.2 | red |
| RP11-334C17.5 | red |
| RP11-338N10.3 | red |
| RP11-33B1.4 | red |
| RP11-344P13.6 | red |
| RP11-347I19.8 | red |
| RP11-352E6.2 | red |
| RP11-353N14.2 | red |
| RP11-358L22.3 | red |
| RP11-384K6.6 | red |
| RP11-385F5.5 | red |
| RP11-386M24.4 | red |
| RP11-390K5.6 | red |
| RP11-390P24.1 | red |
| RP11-42O15.3 | red |
| RP11-434E6.4 | red |
| RP11-43N16.4 | red |
| RP11-449J21.3 | red |
| RP11-452L6.1 | red |
| RP11-455O6.9 | red |
| RP11-462G12.2 | red |
| RP11-464F9.20 | red |
| RP11-464F9.22 | red |
| RP11-465N4.5 | red |
| RP11-468E2.11 | red |
| RP11-468E2.5 | red |
| RP11-46C24.7 | red |
| RP11-473M20.5 | red |
| RP11-474N24.6 | red |
| RP11-488C13.5 | red |
| RP11-496H1.1 | red |
| RP11-497G19.1 | red |
| RP11-497G19.2 | red |
| RP11-498P14.3 | red |
| RP11-499P20.2 | red |
| RP11-4K3__A.5 | red |
| RP11-502I4.3 | red |
| RP11-506M12.1 | red |
| RP11-50C13.1 | red |
| RP11-510N19.5 | red |
| RP11-521B24.5 | red |
| RP11-539L10.2 | red |
| RP11-566K19.6 | red |
| RP11-567M16.6 | red |
| RP11-575H3.1 | red |
| RP11-582J16.5 | red |
| RP11-589P10.5 | red |
| RP11-599J14.2 | red |
| RP11-600F24.7 | red |
| RP11-612B6.2 | red |
| RP11-613M10.6 | red |
| RP11-616M22.5 | red |
| RP11-627G23.1 | red |
| RP11-629O1.2 | red |
| RP11-62H7.2 | red |
| RP11-66N24.3 | red |
| RP11-680A11.5 | red |
| RP11-68I3.4 | red |
| RP11-712L6.5 | red |
| RP11-715J22.3 | red |
| RP11-727F15.9 | red |
| RP11-764K9.2 | red |
| RP11-77H9.2 | red |
| RP11-793H13.10 | red |
| RP11-802E16.3 | red |
| RP11-81A22.4 | red |
| RP11-82L18.2 | red |
| RP11-849F2.9 | red |
| RP11-900F13.3 | red |
| RP11-95O2.5 | red |
| RP11-96D1.6 | red |
| RP11-983P16.4 | red |
| RP13-104F24.3 | red |
| RP13-128O4.3 | red |
| RP13-516M14.1 | red |
| RP13-516M14.10 | red |
| RP3-337H4.9 | red |
| RP3-339A18.6 | red |
| RP3-342P20.2 | red |
| RP3-402G11.25 | red |
| RP3-402G11.27 | red |
| RP3-439F8.1 | red |
| RP3-508I15.19 | red |
| RP3-508I15.21 | red |
| RP3-510D11.2 | red |
| RP4-591C20.9 | red |
| RP4-610C12.3 | red |
| RP4-610C12.4 | red |
| RP4-635E18.8 | red |
| RP4-669P10.20 | red |
| RP4-758J18.13 | red |
| RP4-761J14.10 | red |
| RP4-773N10.4 | red |
| RP4-800G7.2 | red |
| RP5-1050D4.5 | red |
| RP5-1056L3.3 | red |
| RP5-1057J7.7 | red |
| RP5-1068E13.7 | red |
| RP5-1092A3.4 | red |
| RP5-1125A11.7 | red |
| RP5-1142A6.9 | red |
| RP5-1159O4.1 | red |
| RP5-831C21.1 | red |
| RP5-882C2.2 | red |
| RP5-890O3.9 | red |
| RP5-901A4.1 | red |
| RPARP-AS1 | red |
| RPL23P2 | red |
| RPS6KL1 | red |
| RRP7B | red |
| RSRP1 | red |
| SALL4 | red |
| SAT1 | red |
| SCMH1 | red |
| SDHAP1 | red |
| SDHAP3 | red |
| SEC31B | red |
| SERHL2 | red |
| SETD4 | red |
| SETD6 | red |
| SFI1 | red |
| SGK494 | red |
| SH3BP5-AS1 | red |
| SIRT5 | red |
| SLC25A14 | red |
| SLC25A25-AS1 | red |
| SLC25A37 | red |
| SLC26A1 | red |
| SLC2A11 | red |
| SLC35E2B | red |
| SLC46A1 | red |
| SLC9A5 | red |
| SLC9A8 | red |
| SMIM3 | red |
| SNHG1 | red |
| SNHG20 | red |
| SNORA59A | red |
| SNORD124 | red |
| SNORD99 | red |
| snoZ196 | red |
| SPACA4 | red |
| SPACA6P | red |
| SPATA25 | red |
| SPTBN5 | red |
| SRP14-AS1 | red |
| SRRM2 | red |
| SRSF2 | red |
| SRSF5 | red |
| STAG3L5P | red |
| STAT2 | red |
| STX16 | red |
| SYT17 | red |
| SZT2 | red |
| TARDBP | red |
| TBC1D20 | red |
| TBC1D24 | red |
| TBC1D8 | red |
| TBX19 | red |
| TDRD10 | red |
| TES | red |
| THBS3 | red |
| TIMM8AP1 | red |
| TMEM130 | red |
| TMEM147-AS1 | red |
| TMEM198B | red |
| TMEM254-AS1 | red |
| TMEM63A | red |
| TMEM92-AS1 | red |
| TNFAIP2 | red |
| TNNT2 | red |
| TP73-AS1 | red |
| TPCN2 | red |
| TRIM17 | red |
| TRIM3 | red |
| TRIM39 | red |
| TRIM45 | red |
| TRIM46 | red |
| TSPYL2 | red |
| TTC32 | red |
| TTLL3 | red |
| U47924.32 | red |
| UNK | red |
| UNKL | red |
| VAMP1 | red |
| VEGFA | red |
| VENTX | red |
| VMAC | red |
| WAC-AS1 | red |
| WDR59 | red |
| WDR91 | red |
| WTIP | red |
| XX-FW83563B9.5 | red |
| XXbac-BPG299F13.17 | red |
| ZDHHC11 | red |
| ZDHHC11B | red |
| ZNF133 | red |
| ZNF18 | red |
| ZNF213-AS1 | red |
| ZNF251 | red |
| ZNF276 | red |
| ZNF324B | red |
| ZNF337 | red |
| ZNF436-AS1 | red |
| ZNF528-AS1 | red |
| ZNF674-AS1 | red |
| ZNF767P | red |
| ZNF783 | red |
| ZNF785 | red |
| ZNF839 | red |
| ZNF862 | red |
| ZRSR2 | red |

Supplementary Table 4

|  | logFC | AveExpr | t | P.Value | adj.P.Val | B |
| --- | --- | --- | --- | --- | --- | --- |
| RP11-371A19.2 | -2.051255828 | 0.353659219 | -44.35169401 | 7.40E-191 | 1.40E-186 | 425.6942434 |
| GPM6A | -2.770347948 | 0.644538805 | -38.19242811 | 3.02E-162 | 2.85E-158 | 360.204933 |
| SERTM1 | -2.357458702 | 0.398571227 | -37.68028489 | 8.94E-160 | 5.63E-156 | 354.544767 |
| SLC6A4 | -3.868631039 | 0.699951926 | -36.67312325 | 7.10E-155 | 3.35E-151 | 343.3199734 |
| SGCG | -1.730182825 | 0.458699048 | -35.10287005 | 3.93E-147 | 1.48E-143 | 325.5768497 |
| ITLN2 | -3.830581752 | 0.882498902 | -33.69268895 | 4.49E-140 | 1.41E-136 | 309.3996235 |
| WNT3A | -2.176594329 | 0.537051939 | -33.39711336 | 1.39E-138 | 3.76E-135 | 305.9808663 |
| CD300LG | -1.897924305 | 0.341680204 | -32.67813826 | 6.14E-135 | 1.45E-131 | 297.6258694 |
| RTKN2 | -3.451216392 | 1.616103839 | -31.07027908 | 1.05E-126 | 2.20E-123 | 278.7512262 |
| LANCL1-AS1 | -1.849283785 | 0.559761979 | -30.88226181 | 9.76E-126 | 1.84E-122 | 276.5278988 |
| LINC00968 | -1.468223264 | 0.455882322 | -30.81847443 | 2.08E-125 | 3.58E-122 | 275.7728728 |
| SCUBE1 | -1.751584679 | 0.521787839 | -30.33765028 | 6.39E-123 | 1.01E-119 | 270.0698763 |
| LGI3 | -3.645301757 | 0.992728616 | -30.28504146 | 1.20E-122 | 1.74E-119 | 269.4446629 |
| LINC00961 | -1.387482244 | 0.73830328 | -30.27821324 | 1.30E-122 | 1.75E-119 | 269.3634976 |
| CA4 | -3.322217791 | 1.021509298 | -29.99845828 | 3.67E-121 | 4.63E-118 | 266.0347233 |
| MYZAP | -2.183447552 | 0.983280275 | -29.57370231 | 5.95E-119 | 7.02E-116 | 260.9682463 |
| FAM107A | -3.854893134 | 1.727682189 | -29.55817423 | 7.16E-119 | 7.96E-116 | 260.7827536 |
| FABP4 | -4.933107452 | 1.99723538 | -29.26154097 | 2.52E-117 | 2.65E-114 | 257.2356829 |
| RP11-251M1.1 | -2.018165665 | 0.723650771 | -29.25340176 | 2.78E-117 | 2.76E-114 | 257.1382612 |
| FENDRR | -2.477700994 | 0.843836676 | -29.12181693 | 1.35E-116 | 1.28E-113 | 255.5625737 |
| GRIA1 | -1.597431053 | 0.418312521 | -28.3895902 | 9.20E-113 | 8.28E-110 | 246.7716493 |
| FXYD1 | -1.336987152 | 0.53386429 | -28.26264288 | 4.27E-112 | 3.66E-109 | 245.2438378 |
| NCKAP5 | -2.261248145 | 0.782309808 | -27.8163967 | 9.44E-110 | 7.75E-107 | 239.8652225 |
| GPD1 | -3.084917103 | 1.208371891 | -27.51428025 | 3.67E-108 | 2.89E-105 | 236.2171195 |
| STX11 | -2.706733832 | 2.324984246 | -27.46714826 | 6.51E-108 | 4.92E-105 | 235.6475353 |
| TEK | -2.706089056 | 1.852879758 | -27.39309477 | 1.60E-107 | 1.16E-104 | 234.7523671 |
| CNTN6 | -1.668530291 | 0.479330206 | -27.28770767 | 5.74E-107 | 4.02E-104 | 233.477936 |
| STXBP6 | -2.14316065 | 0.815107095 | -27.06955669 | 8.13E-106 | 5.48E-103 | 230.8380736 |
| NECAB1 | -1.720559719 | 0.662291529 | -27.03170254 | 1.29E-105 | 8.39E-103 | 230.3797596 |
| KANK3 | -2.055213174 | 1.301306686 | -26.8694061 | 9.26E-105 | 5.83E-102 | 228.4140126 |
| TAL1 | -1.379362993 | 0.662051588 | -26.80788351 | 1.96E-104 | 1.19E-101 | 227.668531 |
| RP11-701P16.5 | -1.854445896 | 0.677868314 | -26.5341603 | 5.47E-103 | 3.23E-100 | 224.3497849 |
| CTB-134H23.3 | -2.328868231 | 0.694797325 | -26.46997691 | 1.20E-102 | 6.85E-100 | 223.5711485 |
| EDNRB | -3.14111172 | 2.146478427 | -26.40946171 | 2.50E-102 | 1.39E-99 | 222.8368647 |
| LIMS2 | -2.233735863 | 1.676696419 | -26.33572313 | 6.14E-102 | 3.31E-99 | 221.9419413 |
| ST8SIA6 | -1.641866335 | 0.573485826 | -26.20863545 | 2.89E-101 | 1.51E-98 | 220.3990808 |
| HSPA12B | -2.126483976 | 1.642473556 | -26.11894598 | 8.61E-101 | 4.40E-98 | 219.3098973 |
| ECSCR | -1.668033947 | 1.355992994 | -26.0753419 | 1.47E-100 | 7.29E-98 | 218.7802739 |
| RP11-673E1.3 | -1.338644927 | 0.603008541 | -26.02593916 | 2.68E-100 | 1.30E-97 | 218.1801435 |
| GPIHBP1 | -3.171706258 | 1.428167601 | -25.99004972 | 4.15E-100 | 1.96E-97 | 217.7441198 |
| UPK3B | -4.394593471 | 1.820772069 | -25.92639914 | 9.01E-100 | 4.15E-97 | 216.9707249 |
| AC093110.3 | -2.344075831 | 1.208616901 | -25.84959937 | 2.30E-99 | 1.03E-96 | 216.037396 |
| ANKRD1 | -3.760572395 | 1.292333711 | -25.84765804 | 2.36E-99 | 1.03E-96 | 216.0138012 |
| ADAMTS8 | -2.696071288 | 1.136585475 | -25.63000909 | 3.35E-98 | 1.44E-95 | 213.3678519 |
| PRX | -2.706252436 | 1.749495194 | -25.61811806 | 3.88E-98 | 1.63E-95 | 213.2232572 |
| RSPO1 | -1.357356477 | 0.399390157 | -25.53281255 | 1.10E-97 | 4.51E-95 | 212.1858415 |
| SEMA3G | -2.305334616 | 1.559087496 | -25.48062509 | 2.08E-97 | 8.34E-95 | 211.5510955 |
| ADRB2 | -2.438155935 | 1.493261916 | -25.45482644 | 2.84E-97 | 1.12E-94 | 211.2372886 |
| RP11-354P11.2 | -1.745415737 | 0.428300926 | -25.42345546 | 4.17E-97 | 1.61E-94 | 210.8556819 |
| HBA1 | -1.551483904 | 0.371928845 | -25.19041147 | 7.18E-96 | 2.71E-93 | 208.0202349 |
| JAM2 | -1.954448429 | 1.643248698 | -25.09054614 | 2.43E-95 | 9.01E-93 | 206.8048728 |
| ACVRL1 | -2.310065333 | 2.996813911 | -24.85704828 | 4.22E-94 | 1.53E-91 | 203.9626365 |
| OTUD1 | -1.802986052 | 2.97972129 | -24.81273858 | 7.25E-94 | 2.58E-91 | 203.4232053 |
| ROBO4 | -2.386897773 | 2.082577557 | -24.80506476 | 7.96E-94 | 2.78E-91 | 203.3297813 |
| RP1-78O14.1 | -2.010171532 | 0.736395761 | -24.74177691 | 1.72E-93 | 5.92E-91 | 202.5592694 |
| TMEM100 | -3.831233668 | 1.824157621 | -24.64883196 | 5.37E-93 | 1.81E-90 | 201.4276294 |
| NOVA2 | -1.132976105 | 0.641656889 | -24.60474624 | 9.20E-93 | 3.05E-90 | 200.8908475 |
| PYCR1 | 3.240552943 | 4.863005454 | 24.57539871 | 1.32E-92 | 4.29E-90 | 200.5335094 |
| CCM2L | -1.694456559 | 1.153282713 | -24.53721132 | 2.10E-92 | 6.72E-90 | 200.0685295 |
| RPL13AP17 | -2.310222966 | 0.662444972 | -24.48864927 | 3.80E-92 | 1.20E-89 | 199.4772154 |
| AGER | -6.257027257 | 4.337782205 | -24.40307533 | 1.08E-91 | 3.35E-89 | 198.4352082 |
| ACADL | -2.166924797 | 0.911552536 | -24.38734764 | 1.31E-91 | 3.99E-89 | 198.2436952 |
| PTPN21 | -1.980897404 | 1.963884622 | -24.37649092 | 1.50E-91 | 4.49E-89 | 198.1114948 |
| CLEC1A | -1.40638626 | 1.03920902 | -24.33422429 | 2.51E-91 | 7.41E-89 | 197.5968202 |
| S1PR1 | -2.62191688 | 3.196765219 | -24.32805464 | 2.71E-91 | 7.86E-89 | 197.5216931 |
| BTNL9 | -2.514998801 | 1.138442167 | -24.3011165 | 3.76E-91 | 1.08E-88 | 197.1936708 |
| CLEC3B | -3.884035879 | 3.022857697 | -24.28892782 | 4.37E-91 | 1.23E-88 | 197.0452508 |
| LDB2 | -2.160516696 | 2.079670628 | -24.157381 | 2.18E-90 | 6.05E-88 | 195.4434348 |
| GS1-600G8.5 | -1.770924062 | 0.520604038 | -24.13649968 | 2.81E-90 | 7.70E-88 | 195.1891717 |
| CLIC5 | -3.660613765 | 2.173408719 | -24.0832553 | 5.39E-90 | 1.45E-87 | 194.5408447 |
| FHL1 | -3.434765679 | 2.693584826 | -24.05412728 | 7.70E-90 | 2.05E-87 | 194.1861748 |
| NMUR1 | -1.102838488 | 0.510140532 | -23.90698364 | 4.65E-89 | 1.22E-86 | 192.3945996 |
| TCF21 | -2.470136642 | 1.303509477 | -23.90177935 | 4.95E-89 | 1.28E-86 | 192.3312368 |
| ARHGAP6 | -1.224495374 | 0.732763126 | -23.88111882 | 6.37E-89 | 1.63E-86 | 192.0796949 |
| RAMP2 | -2.754873163 | 3.674688374 | -23.8503637 | 9.28E-89 | 2.34E-86 | 191.7052589 |
| RP4-575N6.4 | -1.197016222 | 0.649957408 | -23.80083612 | 1.70E-88 | 4.22E-86 | 191.1022926 |
| EPAS1 | -2.591750173 | 5.819857155 | -23.71912653 | 4.61E-88 | 1.13E-85 | 190.1075913 |
| PECAM1 | -2.236201978 | 4.744676094 | -23.61222426 | 1.70E-87 | 4.12E-85 | 188.8063348 |
| RGCC | -2.841564812 | 5.526425647 | -23.53020676 | 4.64E-87 | 1.11E-84 | 187.8081018 |
| PRKG2 | -1.291520446 | 0.618316991 | -23.45404083 | 1.18E-86 | 2.78E-84 | 186.8811905 |
| CD101 | -1.703587187 | 1.147860497 | -23.43551226 | 1.47E-86 | 3.44E-84 | 186.6557208 |
| FCN3 | -4.064777353 | 2.325080168 | -23.42246333 | 1.73E-86 | 3.98E-84 | 186.4969353 |
| FOXF1 | -2.18633908 | 1.834032018 | -23.39707514 | 2.36E-86 | 5.36E-84 | 186.1880098 |
| WWC2 | -1.971196851 | 2.219471591 | -23.28222838 | 9.58E-86 | 2.15E-83 | 184.7907124 |
| EMP2 | -2.758934558 | 4.937142017 | -23.27363047 | 1.06E-85 | 2.36E-83 | 184.6861166 |
| ADAMTS7P3 | -1.669795641 | 0.459284065 | -23.15243003 | 4.67E-85 | 1.03E-82 | 183.2118685 |
| SIRPB1 | -1.788383378 | 0.965946193 | -23.14928876 | 4.86E-85 | 1.05E-82 | 183.1736637 |
| ECEL1P2 | -2.139878269 | 0.777050394 | -23.10509602 | 8.33E-85 | 1.79E-82 | 182.6362117 |
| USHBP1 | -1.115400928 | 0.682185261 | -23.09315158 | 9.64E-85 | 2.04E-82 | 182.4909577 |
| RAMP3 | -2.922202497 | 3.388675833 | -23.08006017 | 1.13E-84 | 2.37E-82 | 182.3317602 |
| GPR146 | -1.006810226 | 0.62189919 | -23.01855174 | 2.39E-84 | 4.94E-82 | 181.5838537 |
| MCEMP1 | -4.137990052 | 2.314133608 | -23.01811486 | 2.41E-84 | 4.94E-82 | 181.5785419 |
| GRK5 | -2.14594741 | 2.110329876 | -23.01593743 | 2.47E-84 | 5.02E-82 | 181.5520676 |
| FGFBP2 | -1.855447898 | 0.632812827 | -22.9893462 | 3.42E-84 | 6.87E-82 | 181.2287697 |
| SLC19A3 | -1.849534861 | 0.839027156 | -22.98479439 | 3.62E-84 | 7.19E-82 | 181.1734305 |
| GSTM5 | -1.212756887 | 0.675774088 | -22.98091692 | 3.79E-84 | 7.46E-82 | 181.1262903 |
| SPOCK2 | -3.254971891 | 3.59856688 | -22.91675755 | 8.29E-84 | 1.61E-81 | 180.3463405 |
| CAV1 | -3.871494789 | 4.881680394 | -22.809427 | 3.07E-83 | 5.92E-81 | 179.0418763 |
| LYVE1 | -2.581085481 | 1.68534144 | -22.77399642 | 4.73E-83 | 9.02E-81 | 178.6113483 |
| TNNC1 | -3.672725516 | 2.603859984 | -22.70414601 | 1.11E-82 | 2.09E-80 | 177.7627044 |
| AC011899.9 | -1.20946867 | 0.753161031 | -22.68162041 | 1.46E-82 | 2.73E-80 | 177.4890687 |
| FIGF | -3.318544963 | 1.567295947 | -22.66476767 | 1.79E-82 | 3.32E-80 | 177.284358 |
| ARHGEF15 | -1.874639929 | 1.636433315 | -22.65387841 | 2.05E-82 | 3.75E-80 | 177.1520915 |
| RGS9 | -1.044247877 | 0.571724841 | -22.64112702 | 2.39E-82 | 4.34E-80 | 176.9972124 |
| MGAT3 | -2.585315743 | 1.345938398 | -22.639201 | 2.45E-82 | 4.40E-80 | 176.9738194 |
| FGD5 | -1.831498742 | 1.95404797 | -22.6273584 | 2.83E-82 | 5.04E-80 | 176.829985 |
| SCN4B | -1.951904846 | 1.138587025 | -22.61774068 | 3.18E-82 | 5.61E-80 | 176.7131769 |
| PCAT19 | -1.723501641 | 1.250510835 | -22.51937593 | 1.05E-81 | 1.84E-79 | 175.5187389 |
| CDH5 | -2.360099374 | 3.182369198 | -22.47387306 | 1.84E-81 | 3.18E-79 | 174.9663343 |
| ARHGEF26 | -2.307833048 | 2.046372665 | -22.38641679 | 5.33E-81 | 9.14E-79 | 173.9048662 |
| PLAC9 | -2.25928144 | 2.146246232 | -22.38221364 | 5.61E-81 | 9.54E-79 | 173.8538604 |
| GDF10 | -2.582844485 | 1.182534786 | -22.36599551 | 6.83E-81 | 1.15E-78 | 173.6570592 |
| NXPH3 | -1.133638176 | 0.628145154 | -22.2834436 | 1.87E-80 | 3.12E-78 | 172.655509 |
| SH2D3C | -1.943137967 | 2.346964771 | -22.22536727 | 3.78E-80 | 6.27E-78 | 171.9510992 |
| FMO2 | -3.109494125 | 2.621794139 | -22.21125554 | 4.49E-80 | 7.38E-78 | 171.7799623 |
| EFCC1 | -1.905567446 | 1.368695127 | -22.20591307 | 4.80E-80 | 7.81E-78 | 171.7151753 |
| FHL5 | -1.91430494 | 1.135125313 | -22.1723126 | 7.22E-80 | 1.17E-77 | 171.3077421 |
| PDLIM2 | -1.449636721 | 1.595378098 | -22.15175869 | 9.27E-80 | 1.48E-77 | 171.0585372 |
| EMCN | -2.272406884 | 1.831388703 | -22.10806563 | 1.58E-79 | 2.50E-77 | 170.5288548 |
| RP11-598F7.3 | -2.164100567 | 0.986883862 | -22.06538376 | 2.65E-79 | 4.17E-77 | 170.0115273 |
| C14orf132 | -2.163980377 | 1.642229178 | -22.03587087 | 3.80E-79 | 5.93E-77 | 169.6538718 |
| SOX7 | -2.057119487 | 1.348395367 | -21.97946548 | 7.54E-79 | 1.17E-76 | 168.9704483 |
| FRMD3 | -1.311301808 | 0.744796965 | -21.93893114 | 1.23E-78 | 1.89E-76 | 168.4794323 |
| ABCA8 | -1.901547515 | 0.926318134 | -21.89433579 | 2.12E-78 | 3.23E-76 | 167.9393309 |
| SDPR | -3.417324567 | 3.696445147 | -21.86714349 | 2.95E-78 | 4.46E-76 | 167.6100572 |
| CDO1 | -1.731969721 | 0.95097807 | -21.7912942 | 7.42E-78 | 1.11E-75 | 166.691824 |
| RP11-64B16.2 | -1.344765373 | 0.873097292 | -21.71895786 | 1.79E-77 | 2.66E-75 | 165.8164451 |
| FAM46B | -2.416586224 | 1.802885663 | -21.69734668 | 2.32E-77 | 3.43E-75 | 165.5549806 |
| CD36 | -2.757856168 | 1.78761382 | -21.69287542 | 2.45E-77 | 3.59E-75 | 165.5008885 |
| MYCT1 | -1.818360516 | 1.955254407 | -21.6861727 | 2.66E-77 | 3.87E-75 | 165.419803 |
| CALCRL | -2.466025768 | 2.701551022 | -21.59102991 | 8.44E-77 | 1.22E-74 | 164.2691372 |
| CYYR1 | -1.897298318 | 2.339182031 | -21.58260405 | 9.35E-77 | 1.34E-74 | 164.1672629 |
| LIN7A | -1.534884886 | 0.747965589 | -21.56442432 | 1.17E-76 | 1.66E-74 | 163.9474736 |
| SOX17 | -1.663732347 | 1.068475689 | -21.50567094 | 2.38E-76 | 3.35E-74 | 163.2373102 |
| SOSTDC1 | -2.813884745 | 0.979079327 | -21.47917552 | 3.28E-76 | 4.59E-74 | 162.9171327 |
| ALDH18A1 | 1.563811401 | 4.754694189 | 21.44153359 | 5.18E-76 | 7.19E-74 | 162.462342 |
| HIGD1B | -2.394237696 | 2.032826192 | -21.4064139 | 7.93E-76 | 1.09E-73 | 162.0381157 |
| RASIP1 | -1.898664388 | 1.88665521 | -21.40442954 | 8.12E-76 | 1.11E-73 | 162.0141484 |
| FEZ1 | -1.409956081 | 1.216486679 | -21.38776974 | 9.94E-76 | 1.35E-73 | 161.8129406 |
| THSD1 | -1.492363805 | 1.287741765 | -21.37377727 | 1.18E-75 | 1.59E-73 | 161.6439629 |
| GIMAP8 | -2.143150871 | 2.381463607 | -21.30909228 | 2.58E-75 | 3.45E-73 | 160.8629919 |
| FAM189A2 | -2.655232173 | 1.98549163 | -21.30122369 | 2.84E-75 | 3.77E-73 | 160.7680121 |
| STARD8 | -1.61615578 | 1.688915507 | -21.28855331 | 3.31E-75 | 4.37E-73 | 160.6150807 |
| STARD13 | -1.442748143 | 1.520785829 | -21.28495254 | 3.45E-75 | 4.53E-73 | 160.5716216 |
| FAM150B | -1.442936063 | 0.561069199 | -21.28335405 | 3.52E-75 | 4.59E-73 | 160.5523292 |
| VIPR1 | -2.479622762 | 1.722125043 | -21.26160973 | 4.58E-75 | 5.93E-73 | 160.2899123 |
| EMR3 | -1.122150027 | 0.479204837 | -21.17836722 | 1.26E-74 | 1.61E-72 | 159.2856523 |
| RP11-672A2.4 | -1.321059414 | 0.822118554 | -21.03005898 | 7.55E-74 | 9.64E-72 | 157.4977821 |
| AGRP | -1.68808312 | 0.770559394 | -20.98037202 | 1.38E-73 | 1.75E-71 | 156.8992046 |
| NPR1 | -2.342910595 | 1.872306245 | -20.9095342 | 3.24E-73 | 4.08E-71 | 156.0461848 |
| INMT | -3.635327886 | 2.949898105 | -20.86641494 | 5.46E-73 | 6.83E-71 | 155.5271606 |
| FAM110D | -1.360976912 | 0.970101735 | -20.83731215 | 7.76E-73 | 9.64E-71 | 155.1769443 |
| ADRB1 | -2.389180068 | 1.194335141 | -20.81814206 | 9.78E-73 | 1.21E-70 | 154.9462968 |
| ERG | -1.629982543 | 1.743833496 | -20.79638558 | 1.27E-72 | 1.56E-70 | 154.6845708 |
| LRRC36 | -2.137557441 | 0.908069173 | -20.77729012 | 1.60E-72 | 1.95E-70 | 154.4548914 |
| HBA2 | -4.148303299 | 2.853886229 | -20.70421265 | 3.87E-72 | 4.69E-70 | 153.5762262 |
| PTPRB | -2.149279423 | 1.982803209 | -20.66796616 | 5.99E-72 | 7.21E-70 | 153.140591 |
| SEMA6A | -1.54664376 | 0.940897715 | -20.65166344 | 7.30E-72 | 8.72E-70 | 152.944694 |
| PHACTR1 | -1.687147298 | 1.311042276 | -20.63823511 | 8.58E-72 | 1.02E-69 | 152.7833552 |
| SMAD6 | -1.663887541 | 1.409730799 | -20.56792725 | 2.00E-71 | 2.36E-69 | 151.9389013 |
| ARC | -1.532357437 | 0.578342922 | -20.50757437 | 4.14E-71 | 4.86E-69 | 151.214398 |
| DNASE1L3 | -2.025960107 | 0.880855682 | -20.49558604 | 4.79E-71 | 5.58E-69 | 151.0705275 |
| PKNOX2 | -1.259226528 | 0.699440275 | -20.40650414 | 1.40E-70 | 1.62E-68 | 150.0019181 |
| RP5-839B4.8 | -1.953615645 | 0.836428481 | -20.38491402 | 1.81E-70 | 2.09E-68 | 149.7430487 |
| TGFBR3 | -2.063857464 | 1.567425681 | -20.31399132 | 4.26E-70 | 4.88E-68 | 148.8930131 |
| IGSF10 | -1.633581892 | 0.742417828 | -20.28001435 | 6.41E-70 | 7.29E-68 | 148.4859733 |
| GOLM1 | 2.433849681 | 5.740692651 | 20.18754237 | 1.95E-69 | 2.20E-67 | 147.3787975 |
| RP11-253E3.3 | -1.145461603 | 0.857328462 | -20.05557682 | 9.50E-69 | 1.07E-66 | 145.800391 |
| CLEC14A | -2.150395089 | 3.559378529 | -20.02215072 | 1.42E-68 | 1.59E-66 | 145.4009017 |
| DPEP2 | -1.643713764 | 1.408510097 | -19.97495347 | 2.50E-68 | 2.78E-66 | 144.8370471 |
| CHIAP2 | -2.040245055 | 0.53335011 | -19.97038141 | 2.64E-68 | 2.90E-66 | 144.7824395 |
| SLC39A8 | -2.588502025 | 4.27192838 | -19.90195224 | 5.99E-68 | 6.54E-66 | 143.9654314 |
| COL6A6 | -1.823206616 | 0.931741832 | -19.87380703 | 8.40E-68 | 9.11E-66 | 143.6295537 |
| MMRN2 | -1.81558668 | 2.467136792 | -19.84834057 | 1.14E-67 | 1.23E-65 | 143.3257255 |
| GRASP | -1.907195275 | 2.102634857 | -19.8480581 | 1.14E-67 | 1.23E-65 | 143.3223559 |
| NLRC4 | -1.371328981 | 1.233778947 | -19.79483142 | 2.16E-67 | 2.31E-65 | 142.6875895 |
| GPA33 | -1.955220091 | 0.742810067 | -19.69858999 | 6.83E-67 | 7.25E-65 | 141.5407279 |
| TMEM177 | 1.171899727 | 2.31096347 | 19.6883953 | 7.72E-67 | 8.15E-65 | 141.4193107 |
| DACH1 | -1.513275029 | 0.838400249 | -19.67803035 | 8.74E-67 | 9.17E-65 | 141.2958791 |
| KAL1 | -2.655791069 | 2.482851754 | -19.67683645 | 8.86E-67 | 9.25E-65 | 141.2816624 |
| PRKCE | -1.299726532 | 1.598768758 | -19.6294865 | 1.56E-66 | 1.62E-64 | 140.7179741 |
| OR7E47P | -1.313472975 | 1.003259255 | -19.60493155 | 2.09E-66 | 2.16E-64 | 140.4257674 |
| PTCRA | -1.050165523 | 0.557452598 | -19.58257715 | 2.73E-66 | 2.81E-64 | 140.1598152 |
| LMO2 | -1.595284764 | 2.309882425 | -19.5701243 | 3.17E-66 | 3.24E-64 | 140.0116907 |
| CAT | -1.891828208 | 4.934029312 | -19.55555292 | 3.77E-66 | 3.83E-64 | 139.8383924 |
| SLIT2 | -2.009897529 | 1.844790969 | -19.5438414 | 4.34E-66 | 4.38E-64 | 139.6991268 |
| CLDN18 | -5.601451244 | 2.989245325 | -19.48897583 | 8.36E-66 | 8.39E-64 | 139.0469428 |
| RP11-736K20.4 | -1.0898618 | 0.588538633 | -19.41411088 | 2.04E-65 | 2.04E-63 | 138.1576764 |
| GLDN | -1.541792022 | 0.95674557 | -19.31038798 | 7.02E-65 | 6.98E-63 | 136.9268903 |
| EMR1 | -1.499781281 | 0.753797356 | -19.260342 | 1.27E-64 | 1.26E-62 | 136.3335749 |
| RASGRP4 | -1.056828528 | 0.859654286 | -19.25421408 | 1.37E-64 | 1.35E-62 | 136.2609503 |
| ADAMTSL3 | -1.32860411 | 0.826396922 | -19.24096861 | 1.60E-64 | 1.57E-62 | 136.1039906 |
| TGM1 | -1.233817013 | 0.710608165 | -19.21309311 | 2.23E-64 | 2.18E-62 | 135.7737456 |
| FPR2 | -1.766891311 | 0.900656295 | -19.15750344 | 4.33E-64 | 4.19E-62 | 135.1155 |
| LRRN3 | -1.592790732 | 0.730844115 | -19.12729383 | 6.19E-64 | 5.97E-62 | 134.7579714 |
| PLA2G4F | -2.456194695 | 1.664116783 | -19.08016815 | 1.08E-63 | 1.04E-61 | 134.2005093 |
| SEMA5A | -2.06446263 | 1.568401398 | -18.98700614 | 3.27E-63 | 3.11E-61 | 133.0994428 |
| RBP2 | -1.662319281 | 0.575630874 | -18.98387628 | 3.40E-63 | 3.21E-61 | 133.0624742 |
| GIMAP1 | -1.422166397 | 1.452267622 | -18.95304851 | 4.90E-63 | 4.60E-61 | 132.6984285 |
| AOC3 | -2.663763342 | 3.595181169 | -18.95015399 | 5.07E-63 | 4.74E-61 | 132.6642546 |
| SRPK1 | 1.437078772 | 3.703274757 | 18.94857216 | 5.16E-63 | 4.81E-61 | 132.6455793 |
| SLC14A1 | -1.138899068 | 0.469350801 | -18.93524166 | 6.05E-63 | 5.60E-61 | 132.4882133 |
| CLDN5 | -2.614602341 | 2.906805571 | -18.83039158 | 2.09E-62 | 1.93E-60 | 131.2514199 |
| LINC01272 | -2.246027663 | 2.402039848 | -18.8223553 | 2.30E-62 | 2.11E-60 | 131.1566961 |
| VEPH1 | -2.405683681 | 1.634307062 | -18.7857032 | 3.55E-62 | 3.24E-60 | 130.7248067 |
| PAICS | 1.646987178 | 3.963808995 | 18.77505586 | 4.03E-62 | 3.66E-60 | 130.5993839 |
| HHIP | -2.429933841 | 1.087751503 | -18.7494879 | 5.45E-62 | 4.93E-60 | 130.2982741 |
| MME | -2.262114509 | 1.395190362 | -18.73405366 | 6.54E-62 | 5.89E-60 | 130.1165582 |
| LRRC32 | -2.006512002 | 3.124760473 | -18.7255433 | 7.24E-62 | 6.48E-60 | 130.0163774 |
| PEAR1 | -1.343491086 | 1.244511197 | -18.6974167 | 1.01E-61 | 8.99E-60 | 129.6853647 |
| PPP1R14B | 1.993647907 | 5.526008479 | 18.64915851 | 1.78E-61 | 1.58E-59 | 129.1177298 |
| CFP | -1.206722564 | 0.896055998 | -18.6093392 | 2.85E-61 | 2.52E-59 | 128.6496445 |
| DKK2 | -1.389890662 | 1.021746996 | -18.59818965 | 3.26E-61 | 2.86E-59 | 128.5186259 |
| ARHGAP31 | -1.911890994 | 2.423762253 | -18.59254862 | 3.48E-61 | 3.04E-59 | 128.452346 |
| CORO2B | -1.292467281 | 0.892712322 | -18.5861336 | 3.75E-61 | 3.27E-59 | 128.3769783 |
| SBSPON | -1.601842618 | 0.901021796 | -18.56750169 | 4.68E-61 | 4.05E-59 | 128.158118 |
| RAI2 | -1.905284627 | 2.281131386 | -18.5456506 | 6.05E-61 | 5.22E-59 | 127.9015175 |
| CH17-360D5.2 | -1.695393715 | 0.766239758 | -18.51721718 | 8.46E-61 | 7.26E-59 | 127.56774 |
| SULT1C4 | -1.338122542 | 0.864365288 | -18.50867329 | 9.35E-61 | 7.99E-59 | 127.4674707 |
| RHOJ | -1.48596684 | 2.018508147 | -18.45742512 | 1.71E-60 | 1.46E-58 | 126.8662936 |
| TMEM88 | -1.693581339 | 1.765220481 | -18.42621979 | 2.47E-60 | 2.08E-58 | 126.5004534 |
| CSRNP1 | -2.085262763 | 4.059442948 | -18.41280319 | 2.89E-60 | 2.43E-58 | 126.3432137 |
| TCEAL2 | -1.637318454 | 0.676009256 | -18.38522715 | 4.00E-60 | 3.34E-58 | 126.0201264 |
| SFXN1 | 1.217420831 | 2.748337694 | 18.37795808 | 4.36E-60 | 3.62E-58 | 125.9349823 |
| F11 | -1.380590937 | 0.499255933 | -18.37775667 | 4.37E-60 | 3.62E-58 | 125.9326232 |
| RCC1 | 1.558385117 | 3.945831615 | 18.37559391 | 4.48E-60 | 3.70E-58 | 125.9072923 |
| ACKR4 | -1.273125609 | 0.652998234 | -18.35594851 | 5.65E-60 | 4.64E-58 | 125.6772361 |
| CGNL1 | -2.024758258 | 2.452386897 | -18.35034234 | 6.03E-60 | 4.93E-58 | 125.6115979 |
| ADPRH | -1.304881647 | 2.207715392 | -18.34512599 | 6.41E-60 | 5.22E-58 | 125.5505287 |
| THBD | -2.346101547 | 3.451260816 | -18.32971551 | 7.68E-60 | 6.23E-58 | 125.370142 |
| AC109642.1 | -1.528297646 | 1.017757661 | -18.3271297 | 7.92E-60 | 6.39E-58 | 125.339878 |
| TIE1 | -1.694601655 | 2.32389352 | -18.31301218 | 9.35E-60 | 7.52E-58 | 125.1746693 |
| KL | -1.507814037 | 0.797087559 | -18.29608673 | 1.14E-59 | 9.13E-58 | 124.9766476 |
| UBE2T | 2.533330075 | 3.684809897 | 18.25600289 | 1.83E-59 | 1.46E-57 | 124.5078848 |
| PI16 | -1.655282804 | 0.602214172 | -18.22900897 | 2.51E-59 | 1.99E-57 | 124.1923651 |
| EFNA4 | 1.869328656 | 3.771414326 | 18.21641336 | 2.91E-59 | 2.30E-57 | 124.0451857 |
| CCL23 | -1.925925716 | 1.305941736 | -18.19618119 | 3.69E-59 | 2.90E-57 | 123.8088333 |
| CASQ2 | -1.127991715 | 0.496988783 | -18.19449889 | 3.76E-59 | 2.95E-57 | 123.7891841 |
| HBB | -4.533742436 | 4.430396362 | -18.18089494 | 4.41E-59 | 3.44E-57 | 123.6303087 |
| ABI3BP | -2.161639113 | 1.839367302 | -18.17687725 | 4.62E-59 | 3.59E-57 | 123.583394 |
| CNOT11 | 1.136772323 | 4.764928615 | 18.15842266 | 5.74E-59 | 4.44E-57 | 123.3679374 |
| CXCR2 | -1.241467331 | 0.683484122 | -18.1507413 | 6.28E-59 | 4.84E-57 | 123.2782762 |
| ODF3L1 | -1.02936998 | 0.59910287 | -18.09556561 | 1.20E-58 | 9.20E-57 | 122.6345538 |
| DES | -3.039764331 | 2.22086198 | -18.06407915 | 1.73E-58 | 1.32E-56 | 122.2674614 |
| GLIPR2 | -2.12697862 | 3.641851295 | -18.06211437 | 1.77E-58 | 1.35E-56 | 122.2445606 |
| GIMAP6 | -1.902658057 | 2.802928543 | -18.05984026 | 1.82E-58 | 1.38E-56 | 122.2180554 |
| SELP | -1.835962104 | 1.704622653 | -18.0407736 | 2.28E-58 | 1.72E-56 | 121.9958675 |
| SPN | -1.963774656 | 2.125298256 | -18.00671508 | 3.39E-58 | 2.55E-56 | 121.5991464 |
| RSPO2 | -1.214679787 | 0.4789018 | -17.99530536 | 3.87E-58 | 2.90E-56 | 121.4662925 |
| SPRYD7 | -1.032105084 | 2.460266107 | -17.9286455 | 8.44E-58 | 6.30E-56 | 120.6906066 |
| PIP5K1B | -1.849391517 | 1.435068366 | -17.90589797 | 1.10E-57 | 8.15E-56 | 120.4261007 |
| ADH1B | -3.701163456 | 2.588872931 | -17.90099313 | 1.17E-57 | 8.60E-56 | 120.3690809 |
| NME1 | 1.925065467 | 3.907610651 | 17.8980108 | 1.21E-57 | 8.87E-56 | 120.3344129 |
| FAM162B | -1.783606769 | 1.677458994 | -17.86772852 | 1.72E-57 | 1.26E-55 | 119.9824956 |
| CPB2 | -3.233983169 | 1.452605395 | -17.86679295 | 1.74E-57 | 1.27E-55 | 119.9716259 |
| PPAP2C | 2.121318102 | 3.402683097 | 17.86624159 | 1.75E-57 | 1.27E-55 | 119.9652202 |
| KANK2 | -1.473372171 | 3.667823866 | -17.84450487 | 2.25E-57 | 1.63E-55 | 119.7127294 |
| OCIAD2 | 1.983607167 | 5.167965034 | 17.8152228 | 3.17E-57 | 2.29E-55 | 119.3727392 |
| RP11-389C8.2 | -1.179446967 | 1.225628029 | -17.81372142 | 3.23E-57 | 2.32E-55 | 119.3553114 |
| AFF3 | -1.352057786 | 0.713935173 | -17.80734577 | 3.48E-57 | 2.49E-55 | 119.2813089 |
| RADIL | -1.046619817 | 0.570487485 | -17.77706976 | 4.95E-57 | 3.52E-55 | 118.9300033 |
| HBEGF | -2.365284016 | 3.204834954 | -17.7600192 | 6.03E-57 | 4.28E-55 | 118.7322384 |
| TNXB | -2.089222379 | 1.322757826 | -17.74517767 | 7.17E-57 | 5.07E-55 | 118.5601426 |
| PTRF | -2.11752102 | 5.651853743 | -17.72775009 | 8.78E-57 | 6.19E-55 | 118.3581165 |
| SLIT3 | -2.038130016 | 1.656063711 | -17.70906138 | 1.09E-56 | 7.66E-55 | 118.1415387 |
| SASH1 | -1.492033102 | 2.273139466 | -17.70429577 | 1.15E-56 | 8.07E-55 | 118.0863227 |
| C1QTNF7 | -1.38934413 | 0.79401426 | -17.69002201 | 1.36E-56 | 9.49E-55 | 117.9209696 |
| MAMDC2 | -2.416392242 | 1.803136459 | -17.67112322 | 1.70E-56 | 1.18E-54 | 117.7021017 |
| FAM136A | 1.129168878 | 3.935112517 | 17.66739377 | 1.77E-56 | 1.23E-54 | 117.6589192 |
| CAV2 | -2.493629428 | 3.646420127 | -17.62614677 | 2.86E-56 | 1.97E-54 | 117.1815193 |
| PALMD | -1.450656942 | 1.358979446 | -17.59605796 | 4.06E-56 | 2.79E-54 | 116.833486 |
| CTHRC1 | 3.240254681 | 4.572641419 | 17.59476088 | 4.12E-56 | 2.82E-54 | 116.8184871 |
| RETN | -3.01617895 | 1.837511725 | -17.56078287 | 6.12E-56 | 4.17E-54 | 116.4257016 |
| IL3RA | -1.352168846 | 2.120532794 | -17.54989309 | 6.94E-56 | 4.71E-54 | 116.2998666 |
| B3GNT3 | 3.081476142 | 3.650004928 | 17.54932595 | 6.99E-56 | 4.73E-54 | 116.2933137 |
| CD93 | -2.08964914 | 3.864194103 | -17.5257906 | 9.18E-56 | 6.19E-54 | 116.0214422 |
| SNX22 | -1.145070266 | 1.107785533 | -17.45365195 | 2.12E-55 | 1.42E-53 | 115.1888476 |
| ABCG2 | -1.223041885 | 1.052097181 | -17.40168463 | 3.87E-55 | 2.58E-53 | 114.5897458 |
| SYNM | -1.234084079 | 1.383398173 | -17.36406341 | 5.97E-55 | 3.96E-53 | 114.1563932 |
| TBX3 | -1.577570161 | 1.55350378 | -17.31405272 | 1.07E-54 | 7.01E-53 | 113.5808023 |
| HSPB6 | -2.454406699 | 2.223530905 | -17.31063087 | 1.11E-54 | 7.27E-53 | 113.5414389 |
| ST6GALNAC5 | -1.746701831 | 1.469881198 | -17.30894911 | 1.13E-54 | 7.38E-53 | 113.5220936 |
| COX4I2 | -2.085100537 | 2.690377131 | -17.30813617 | 1.14E-54 | 7.43E-53 | 113.5127426 |
| ETV4 | 2.62391533 | 3.274516737 | 17.29512352 | 1.33E-54 | 8.60E-53 | 113.3630815 |
| FXYD6 | -1.600051473 | 2.098349092 | -17.28002206 | 1.58E-54 | 1.02E-52 | 113.1894429 |
| BCHE | -1.592219812 | 0.921333631 | -17.270886 | 1.75E-54 | 1.13E-52 | 113.0844197 |
| DNASE2B | -1.24088132 | 0.552823329 | -17.25465876 | 2.11E-54 | 1.36E-52 | 112.8979253 |
| TOP2A | 2.817507694 | 3.661240075 | 17.24504256 | 2.36E-54 | 1.51E-52 | 112.7874369 |
| TSPAN18 | -1.646432623 | 1.966482302 | -17.24273717 | 2.43E-54 | 1.55E-52 | 112.7609514 |
| HEG1 | -1.967206333 | 3.157793888 | -17.22094213 | 3.12E-54 | 1.98E-52 | 112.5106173 |
| COX7A1 | -1.622141332 | 2.574179266 | -17.21920422 | 3.18E-54 | 2.02E-52 | 112.4906605 |
| ATIC | 1.201215077 | 4.570954289 | 17.18963453 | 4.48E-54 | 2.83E-52 | 112.1512082 |
| PPP1R14BP3 | 1.90318164 | 4.747122138 | 17.18199479 | 4.89E-54 | 3.08E-52 | 112.0635378 |
| DUOX1 | -2.71325912 | 2.308580692 | -17.17511965 | 5.29E-54 | 3.32E-52 | 111.9846526 |
| KIAA1324L | -1.693486913 | 1.777286155 | -17.13376318 | 8.52E-54 | 5.31E-52 | 111.5103543 |
| CAMP | -1.556157836 | 0.72700295 | -17.12383375 | 9.55E-54 | 5.92E-52 | 111.3965358 |
| FFAR4 | -1.296810211 | 0.786197894 | -17.10384469 | 1.20E-53 | 7.42E-52 | 111.1674738 |
| TNS1 | -2.132658556 | 4.092911745 | -17.09797349 | 1.29E-53 | 7.91E-52 | 111.1002108 |
| LAMP3 | -3.16716728 | 4.543074973 | -17.09743673 | 1.29E-53 | 7.94E-52 | 111.0940618 |
| CD300C | -1.664576213 | 1.725212529 | -17.01996334 | 3.15E-53 | 1.93E-51 | 110.2072409 |
| PDZD2 | -1.755302848 | 1.413143822 | -17.00653452 | 3.68E-53 | 2.24E-51 | 110.0536646 |
| FGFR4 | -2.306227597 | 1.885480947 | -16.99237137 | 4.33E-53 | 2.63E-51 | 109.8917354 |
| PDIA4 | 1.699332585 | 6.922281634 | 16.96234902 | 6.11E-53 | 3.69E-51 | 109.5486401 |
| DENND3 | -1.391888193 | 2.125973716 | -16.94963172 | 7.07E-53 | 4.25E-51 | 109.4033701 |
| RP11-635O16.2 | -1.38394119 | 0.598876256 | -16.93022669 | 8.84E-53 | 5.30E-51 | 109.1817792 |
| KIAA1462 | -1.811310606 | 2.576342163 | -16.90455624 | 1.19E-52 | 7.09E-51 | 108.8887778 |
| FOLR3 | -1.404400635 | 0.46796795 | -16.90062348 | 1.24E-52 | 7.39E-51 | 108.8439033 |
| ANGPT1 | -1.730267507 | 1.413213465 | -16.87655695 | 1.63E-52 | 9.71E-51 | 108.5693726 |
| DAPK2 | -1.4003411 | 1.356602625 | -16.87252221 | 1.71E-52 | 1.01E-50 | 108.523361 |
| PRAM1 | -1.49338009 | 1.34507738 | -16.84210721 | 2.43E-52 | 1.43E-50 | 108.1766382 |
| SFTPC | -7.741347235 | 5.905968369 | -16.83648955 | 2.59E-52 | 1.52E-50 | 108.1126226 |
| NOTCH4 | -1.363804795 | 1.844438644 | -16.82980312 | 2.79E-52 | 1.64E-50 | 108.0364377 |
| LTBP4 | -2.120002791 | 3.694576375 | -16.79896181 | 3.97E-52 | 2.32E-50 | 107.6851717 |
| PNPLA6 | -1.349414511 | 3.468058463 | -16.78716776 | 4.55E-52 | 2.65E-50 | 107.5509041 |
| TBX2-AS1 | -1.200863936 | 0.939876881 | -16.76102411 | 6.13E-52 | 3.56E-50 | 107.2533957 |
| SESN1 | -1.37363266 | 2.340436018 | -16.7325731 | 8.49E-52 | 4.92E-50 | 106.9298184 |
| NDST1 | -1.406133209 | 3.610623253 | -16.72695781 | 9.05E-52 | 5.23E-50 | 106.8659781 |
| MYO16-AS1 | -1.735594625 | 0.663919274 | -16.70551849 | 1.16E-51 | 6.65E-50 | 106.6223054 |
| TRPV2 | -1.725090292 | 3.096560572 | -16.69920622 | 1.24E-51 | 7.13E-50 | 106.5505835 |
| GYPC | -1.602153422 | 2.797478033 | -16.6889983 | 1.40E-51 | 7.99E-50 | 106.4346188 |
| AC124789.1 | -1.390267219 | 0.61982057 | -16.67881597 | 1.57E-51 | 8.94E-50 | 106.3189702 |
| RP11-401P9.4 | -1.514404011 | 0.967292652 | -16.67360137 | 1.66E-51 | 9.46E-50 | 106.2597539 |
| WFS1 | -1.627679217 | 3.874424139 | -16.67092233 | 1.72E-51 | 9.73E-50 | 106.2293337 |
| LMCD1 | -1.358324172 | 2.200716874 | -16.6565343 | 2.02E-51 | 1.14E-49 | 106.0659893 |
| CASKIN2 | -1.37475634 | 2.909652803 | -16.64701939 | 2.25E-51 | 1.27E-49 | 105.9579965 |
| LGR4 | 2.045393538 | 3.235572934 | 16.6337632 | 2.62E-51 | 1.47E-49 | 105.8075781 |
| SPTBN1 | -1.624374898 | 4.576863032 | -16.63088614 | 2.71E-51 | 1.52E-49 | 105.7749378 |
| RNU6-529P | -1.323405802 | 1.202097806 | -16.59256391 | 4.19E-51 | 2.34E-49 | 105.3403676 |
| GPX3 | -2.522020164 | 5.813884861 | -16.58596514 | 4.52E-51 | 2.52E-49 | 105.2655752 |
| ANGPTL1 | -1.476238395 | 0.866372974 | -16.57812339 | 4.94E-51 | 2.74E-49 | 105.1767087 |
| GPM6B | -1.491381387 | 1.408884267 | -16.57485695 | 5.12E-51 | 2.84E-49 | 105.1396963 |
| TMEM204 | -1.607121245 | 3.256887828 | -16.56399609 | 5.80E-51 | 3.20E-49 | 105.0166501 |
| ADARB1 | -1.284187281 | 1.786649807 | -16.55988196 | 6.08E-51 | 3.35E-49 | 104.9700476 |
| FGR | -1.922396693 | 2.849695397 | -16.52157539 | 9.40E-51 | 5.14E-49 | 104.5363368 |
| CPED1 | -1.440386067 | 1.320967429 | -16.50482665 | 1.14E-50 | 6.20E-49 | 104.3468225 |
| F8 | -1.197445263 | 1.554103429 | -16.46997889 | 1.69E-50 | 9.14E-49 | 103.9527439 |
| TBX4 | -1.828313622 | 1.774854648 | -16.37755895 | 4.82E-50 | 2.59E-48 | 102.9091142 |
| BCL6B | -1.357862212 | 2.042631413 | -16.36231348 | 5.73E-50 | 3.07E-48 | 102.7371707 |
| CXCR1 | -1.118941666 | 0.505280953 | -16.34422297 | 7.03E-50 | 3.76E-48 | 102.5332185 |
| ESAM | -2.015729387 | 3.986441425 | -16.2839984 | 1.39E-49 | 7.41E-48 | 101.854865 |
| PPP1R15A | -1.660988088 | 4.838723738 | -16.24341361 | 2.20E-49 | 1.17E-47 | 101.3982686 |
| ADCY4 | -1.032523027 | 1.109696884 | -16.18303213 | 4.35E-49 | 2.30E-47 | 100.7197626 |
| PEAK1 | -1.171744982 | 1.637679088 | -16.16933581 | 5.07E-49 | 2.68E-47 | 100.5659932 |
| TMEM184A | 1.881217653 | 2.398668159 | 16.14610592 | 6.59E-49 | 3.47E-47 | 100.3053056 |
| SFXN4 | 1.252926664 | 3.721925955 | 16.13457023 | 7.51E-49 | 3.94E-47 | 100.1759054 |
| CDC20 | 2.705963824 | 3.482080134 | 16.11772664 | 9.08E-49 | 4.75E-47 | 99.98702927 |
| C10orf54 | -1.48324873 | 3.473877635 | -16.10282159 | 1.07E-48 | 5.59E-47 | 99.81995536 |
| SCN7A | -1.990302037 | 1.576961443 | -16.09174024 | 1.22E-48 | 6.29E-47 | 99.69578134 |
| SLC5A9 | -1.262720674 | 0.597070831 | -16.08793105 | 1.27E-48 | 6.55E-47 | 99.65310454 |
| GGCT | 1.549521271 | 4.834992484 | 16.0705814 | 1.54E-48 | 7.94E-47 | 99.45877552 |
| LHFP | -1.573262407 | 3.745502962 | -16.06268611 | 1.69E-48 | 8.66E-47 | 99.37036959 |
| MS4A7 | -2.072696342 | 3.110329593 | -16.05365455 | 1.87E-48 | 9.56E-47 | 99.26926161 |
| ITIH5 | -1.562777181 | 1.15636018 | -16.05011623 | 1.94E-48 | 9.92E-47 | 99.2296563 |
| RXRG | -1.284295009 | 0.564507862 | -16.0250811 | 2.57E-48 | 1.31E-46 | 98.9495306 |
| PTH1R | -1.152794732 | 0.951678939 | -16.01362295 | 2.93E-48 | 1.49E-46 | 98.8213796 |
| FLI1 | -1.325173975 | 1.94188847 | -16.00694384 | 3.16E-48 | 1.60E-46 | 98.74669538 |
| RP11-359M6.2 | -2.033631268 | 1.023538179 | -16.00613596 | 3.18E-48 | 1.61E-46 | 98.73766281 |
| KDELR3 | 1.95567845 | 4.427471298 | 15.99894968 | 3.45E-48 | 1.74E-46 | 98.65732292 |
| WIF1 | -4.045116476 | 2.386554092 | -15.99379013 | 3.66E-48 | 1.84E-46 | 98.59964998 |
| ITGA8 | -1.718984076 | 1.713313176 | -15.97006809 | 4.78E-48 | 2.39E-46 | 98.33458264 |
| CCT3 | 1.315503071 | 6.183001 | 15.94594181 | 6.26E-48 | 3.11E-46 | 98.06515928 |
| RILPL2 | -1.092326135 | 2.422864998 | -15.91644583 | 8.71E-48 | 4.32E-46 | 97.7359929 |
| SPP1 | 4.30158016 | 6.493515494 | 15.89649486 | 1.09E-47 | 5.39E-46 | 97.51348477 |
| EML1 | -1.162053906 | 1.307371689 | -15.89547688 | 1.10E-47 | 5.44E-46 | 97.50213446 |
| WASF3 | -1.428589396 | 1.485858255 | -15.89388716 | 1.12E-47 | 5.52E-46 | 97.48441003 |
| S100A3 | -1.679000319 | 1.459292286 | -15.85500244 | 1.73E-47 | 8.50E-46 | 97.05109044 |
| VWF | -2.328467668 | 4.270515783 | -15.85483577 | 1.74E-47 | 8.50E-46 | 97.04923413 |
| AFAP1L1 | -1.301200425 | 1.944286603 | -15.85095633 | 1.81E-47 | 8.86E-46 | 97.00602648 |
| OSCAR | -2.05112401 | 2.651363521 | -15.83663078 | 2.13E-47 | 1.03E-45 | 96.84651161 |
| MRPL3 | 1.058305506 | 5.010430509 | 15.8245744 | 2.44E-47 | 1.18E-45 | 96.71230933 |
| CCNB1 | 2.248074376 | 3.567466408 | 15.8226711 | 2.49E-47 | 1.20E-45 | 96.69112707 |
| CRTAC1 | -3.157106941 | 2.668859235 | -15.81990758 | 2.57E-47 | 1.24E-45 | 96.66037304 |
| CCL14 | -1.012065524 | 0.553069056 | -15.81836541 | 2.61E-47 | 1.26E-45 | 96.64321187 |
| ZWINT | 2.023117548 | 3.397065395 | 15.81317514 | 2.77E-47 | 1.33E-45 | 96.58545977 |
| TACC1 | -1.714995925 | 3.2411863 | -15.80999463 | 2.87E-47 | 1.37E-45 | 96.5500741 |
| CBX7 | -1.396353895 | 2.213258786 | -15.80399807 | 3.07E-47 | 1.46E-45 | 96.48336548 |
| NLN | 1.017171012 | 2.094652114 | 15.78189406 | 3.93E-47 | 1.86E-45 | 96.23755917 |
| AC090616.2 | -1.474001726 | 1.228310522 | -15.77758793 | 4.12E-47 | 1.95E-45 | 96.18968947 |
| MARCO | -3.655704551 | 4.306210198 | -15.77631931 | 4.18E-47 | 1.97E-45 | 96.17558771 |
| FAM83A | 3.46099015 | 3.379508184 | 15.77279832 | 4.35E-47 | 2.05E-45 | 96.13645135 |
| TRAP1 | 1.057251753 | 3.54170069 | 15.76761534 | 4.61E-47 | 2.16E-45 | 96.07884824 |
| LILRA5 | -1.608722382 | 1.625341551 | -15.76381447 | 4.81E-47 | 2.25E-45 | 96.03661071 |
| RCAN1 | -1.4596269 | 2.703651971 | -15.75823654 | 5.12E-47 | 2.39E-45 | 95.97463297 |
| P2RY1 | -1.029605761 | 1.017260429 | -15.72969827 | 7.03E-47 | 3.27E-45 | 95.65767805 |
| RMI2 | 1.584444961 | 2.467855005 | 15.72966982 | 7.04E-47 | 3.27E-45 | 95.65736213 |
| CRYAB | -1.79155523 | 2.097995297 | -15.72766624 | 7.20E-47 | 3.33E-45 | 95.63511861 |
| GNL3 | 1.096466393 | 4.586388215 | 15.72674635 | 7.27E-47 | 3.36E-45 | 95.62490656 |
| FLAD1 | 1.245054278 | 3.687134719 | 15.72436027 | 7.47E-47 | 3.44E-45 | 95.59841877 |
| OLR1 | -2.627833963 | 3.434691385 | -15.70882697 | 8.88E-47 | 4.07E-45 | 95.42602445 |
| CASS4 | -1.314116451 | 1.414644492 | -15.69714582 | 1.01E-46 | 4.62E-45 | 95.29642919 |
| SPAG4 | 1.883540468 | 2.614168684 | 15.69576182 | 1.03E-46 | 4.69E-45 | 95.2810771 |
| DLC1 | -2.232112802 | 2.93243984 | -15.68868153 | 1.11E-46 | 5.06E-45 | 95.20254776 |
| AMOTL1 | -1.542986632 | 2.36936577 | -15.68482178 | 1.16E-46 | 5.27E-45 | 95.15974451 |
| PCOLCE2 | -2.023618681 | 1.449215907 | -15.67194287 | 1.34E-46 | 6.07E-45 | 95.01695323 |
| SERINC1 | -1.077155349 | 6.011814931 | -15.61532762 | 2.51E-46 | 1.13E-44 | 94.3898261 |
| DENND2A | -1.306560143 | 1.482336862 | -15.61178067 | 2.62E-46 | 1.18E-44 | 94.35056799 |
| HMGB3 | 2.864278272 | 5.370676609 | 15.60727793 | 2.75E-46 | 1.23E-44 | 94.30073656 |
| FAM83H-AS1 | 1.839889888 | 3.391695634 | 15.57617876 | 3.89E-46 | 1.74E-44 | 93.95672833 |
| NHSL1 | -1.45524414 | 1.942382525 | -15.56886258 | 4.22E-46 | 1.88E-44 | 93.87584113 |
| LGALSL | -1.601159886 | 2.621694907 | -15.56828838 | 4.24E-46 | 1.89E-44 | 93.86949343 |
| MSR1 | -2.258461759 | 3.051514122 | -15.56735531 | 4.29E-46 | 1.90E-44 | 93.85917876 |
| SPTBN2 | 1.697427649 | 2.516727512 | 15.56358797 | 4.47E-46 | 1.98E-44 | 93.81753526 |
| PPAT | 1.25597177 | 2.118122566 | 15.55485765 | 4.93E-46 | 2.17E-44 | 93.72104795 |
| MSRB3 | -1.635470673 | 2.357264525 | -15.55227557 | 5.07E-46 | 2.23E-44 | 93.6925152 |
| LDLR | -1.838756623 | 3.800861198 | -15.54797498 | 5.32E-46 | 2.34E-44 | 93.64499698 |
| MYO19 | 1.13573633 | 2.132141377 | 15.54155915 | 5.71E-46 | 2.50E-44 | 93.57411714 |
| BZW2 | 1.272162949 | 4.595869997 | 15.53769863 | 5.96E-46 | 2.61E-44 | 93.53147337 |
| ST6GALNAC6 | -1.229967739 | 3.607686781 | -15.53334282 | 6.25E-46 | 2.73E-44 | 93.48336395 |
| ANKRD29 | -2.051820601 | 1.763335036 | -15.52750859 | 6.67E-46 | 2.90E-44 | 93.41893445 |
| C2orf40 | -1.950827839 | 1.026077782 | -15.51371023 | 7.78E-46 | 3.38E-44 | 93.26659498 |
| MIR22HG | -1.361481468 | 2.955889754 | -15.51152446 | 7.97E-46 | 3.45E-44 | 93.24246851 |
| MANF | 1.324266012 | 5.178688735 | 15.5111885 | 8.00E-46 | 3.46E-44 | 93.2387603 |
| SLC35F2 | 1.580061087 | 3.382122388 | 15.49714192 | 9.34E-46 | 4.03E-44 | 93.08374984 |
| TNFRSF21 | 1.848969557 | 5.490583911 | 15.4854318 | 1.06E-45 | 4.58E-44 | 92.95456881 |
| MSTO1 | 1.029398388 | 2.123809399 | 15.48426969 | 1.08E-45 | 4.63E-44 | 92.94175111 |
| KAT2A | 1.520898355 | 4.011916446 | 15.48010238 | 1.13E-45 | 4.83E-44 | 92.89579062 |
| CBLC | 2.259474065 | 3.230033232 | 15.47865436 | 1.15E-45 | 4.90E-44 | 92.87982192 |
| MFAP4 | -2.968474875 | 5.102584539 | -15.47471591 | 1.20E-45 | 5.10E-44 | 92.836392 |
| NUSAP1 | 1.927424158 | 3.115775633 | 15.473038 | 1.22E-45 | 5.18E-44 | 92.8178909 |
| RECK | -1.10218321 | 1.54637305 | -15.46834024 | 1.29E-45 | 5.45E-44 | 92.76609638 |
| PCDH12 | -1.280972584 | 1.962412595 | -15.43712469 | 1.82E-45 | 7.68E-44 | 92.42210444 |
| WISP2 | -1.668098001 | 1.636710928 | -15.42740012 | 2.02E-45 | 8.53E-44 | 92.3150013 |
| AQP4 | -3.726220809 | 3.334638192 | -15.41985824 | 2.20E-45 | 9.25E-44 | 92.23195737 |
| ADTRP | -1.431207137 | 1.074092314 | -15.41510346 | 2.32E-45 | 9.73E-44 | 92.17961135 |
| PEBP4 | -3.832745186 | 3.11638588 | -15.37640791 | 3.56E-45 | 1.49E-43 | 91.75386352 |
| FGF2 | -1.08973636 | 0.764023419 | -15.36029885 | 4.25E-45 | 1.78E-43 | 91.57675918 |
| TGFBR2 | -1.679009086 | 5.326847089 | -15.35227167 | 4.64E-45 | 1.94E-43 | 91.48853759 |
| KIF1C | -1.215637322 | 4.17111794 | -15.34315028 | 5.14E-45 | 2.14E-43 | 91.38831438 |
| ZBED2 | -1.793554747 | 1.525316258 | -15.33954403 | 5.34E-45 | 2.22E-43 | 91.34869706 |
| MMRN1 | -1.661204869 | 1.398161983 | -15.33572644 | 5.57E-45 | 2.31E-43 | 91.30676238 |
| AGTR2 | -2.442088609 | 1.144328869 | -15.3287183 | 6.02E-45 | 2.49E-43 | 91.22979243 |
| HN1L | 1.127031003 | 5.037006383 | 15.31930034 | 6.68E-45 | 2.76E-43 | 91.12637978 |
| KCNK3 | -2.061613623 | 1.455591543 | -15.31618486 | 6.92E-45 | 2.85E-43 | 91.09217668 |
| IQGAP3 | 1.865834262 | 2.11137432 | 15.3112021 | 7.31E-45 | 3.00E-43 | 91.03748001 |
| TFB2M | 1.096797653 | 3.679273906 | 15.30685706 | 7.67E-45 | 3.14E-43 | 90.9897901 |
| SLC1A1 | -2.155901288 | 2.427749947 | -15.29750515 | 8.50E-45 | 3.47E-43 | 90.88716607 |
| RP11-609N14.4 | -1.401936435 | 0.762827401 | -15.29672272 | 8.57E-45 | 3.50E-43 | 90.87858121 |
| EIF2AK1 | 1.008877296 | 5.384969193 | 15.29001706 | 9.23E-45 | 3.75E-43 | 90.80501442 |
| CDCA8 | 1.999930701 | 2.709862218 | 15.28338991 | 9.93E-45 | 4.02E-43 | 90.73232272 |
| METTL7A | -1.955623044 | 4.22200871 | -15.27885268 | 1.04E-44 | 4.22E-43 | 90.6825628 |
| KLF6 | -1.640113646 | 4.909149735 | -15.27674274 | 1.07E-44 | 4.31E-43 | 90.65942525 |
| UBE2C | 3.048411056 | 4.021845598 | 15.27081693 | 1.14E-44 | 4.58E-43 | 90.59445036 |
| ADAMTS1 | -1.995390496 | 2.688218047 | -15.26915155 | 1.16E-44 | 4.66E-43 | 90.57619188 |
| FUT2 | 1.969767565 | 2.529192064 | 15.2658796 | 1.20E-44 | 4.82E-43 | 90.54032218 |
| ADM2 | 1.737296419 | 1.97744771 | 15.26105722 | 1.27E-44 | 5.07E-43 | 90.48746169 |
| CD34 | -1.429893278 | 2.66260615 | -15.2367829 | 1.66E-44 | 6.60E-43 | 90.2214893 |
| KRT4 | -2.556607565 | 1.246723205 | -15.20763553 | 2.29E-44 | 9.05E-43 | 89.90236822 |
| GPER1 | -1.338341578 | 1.021702698 | -15.20162684 | 2.44E-44 | 9.65E-43 | 89.83661517 |
| MARCKSL1 | 1.899138669 | 6.561982237 | 15.19151577 | 2.73E-44 | 1.07E-42 | 89.72599564 |
| C8B | -1.805835973 | 0.726784901 | -15.18981618 | 2.78E-44 | 1.09E-42 | 89.70740449 |
| SYNPO | -1.66343891 | 3.668784878 | -15.18837482 | 2.83E-44 | 1.11E-42 | 89.69163879 |
| ITM2A | -1.883869653 | 3.267013469 | -15.1832102 | 2.99E-44 | 1.17E-42 | 89.63515321 |
| ROR1 | -1.305238853 | 1.310368519 | -15.18249336 | 3.02E-44 | 1.18E-42 | 89.62731381 |
| GKN2 | -3.446245858 | 1.785425702 | -15.17952996 | 3.11E-44 | 1.21E-42 | 89.59490754 |
| RP1-186E20.1 | -1.295610513 | 0.753357128 | -15.1765145 | 3.22E-44 | 1.25E-42 | 89.56193474 |
| PPAP2B | -1.246897991 | 3.801750382 | -15.16440869 | 3.68E-44 | 1.43E-42 | 89.42959209 |
| SEMA6D | -1.201593859 | 0.757918742 | -15.15917067 | 3.90E-44 | 1.51E-42 | 89.37234369 |
| NARF | 1.091727085 | 3.220374375 | 15.14782183 | 4.41E-44 | 1.70E-42 | 89.2483376 |
| SLC50A1 | 1.625879582 | 5.266868337 | 15.14273605 | 4.67E-44 | 1.80E-42 | 89.19277982 |
| CNRIP1 | -1.08497891 | 1.663358193 | -15.12875238 | 5.44E-44 | 2.09E-42 | 89.04006289 |
| EPCAM | 1.565450205 | 7.283883883 | 15.12386488 | 5.74E-44 | 2.20E-42 | 88.98670081 |
| LEPR | -1.502394453 | 1.444292025 | -15.11815834 | 6.11E-44 | 2.34E-42 | 88.92440599 |
| POLR2H | 1.101480574 | 3.996632197 | 15.10513094 | 7.05E-44 | 2.69E-42 | 88.78223308 |
| CFD | -2.588495124 | 4.083946308 | -15.07453406 | 9.86E-44 | 3.75E-42 | 88.44853219 |
| CD52 | -2.423923924 | 5.283366331 | -15.07076892 | 1.03E-43 | 3.90E-42 | 88.40748905 |
| SSTR1 | -1.584957433 | 0.880285209 | -15.05446375 | 1.23E-43 | 4.66E-42 | 88.229802 |
| PAFAH1B3 | 1.906984947 | 4.340363125 | 15.02808546 | 1.64E-43 | 6.19E-42 | 87.94252445 |
| KIF20A | 1.784328663 | 2.202888744 | 15.02361387 | 1.72E-43 | 6.49E-42 | 87.89384819 |
| STAC | -1.638811002 | 1.376558002 | -15.02131756 | 1.77E-43 | 6.64E-42 | 87.86885384 |
| ACE | -1.541092611 | 2.506654936 | -15.02060542 | 1.78E-43 | 6.68E-42 | 87.86110289 |
| CCBE1 | -1.679343105 | 0.984992344 | -15.01742328 | 1.84E-43 | 6.91E-42 | 87.82647022 |
| SVEP1 | -1.5619785 | 1.381284474 | -14.9987735 | 2.26E-43 | 8.45E-42 | 87.6235626 |
| TBX2 | -1.619010296 | 2.362689542 | -14.99627938 | 2.32E-43 | 8.67E-42 | 87.59643543 |
| C5AR1 | -1.838301988 | 3.248922143 | -14.99463658 | 2.36E-43 | 8.79E-42 | 87.57856866 |
| DKC1 | 1.072160883 | 4.127041618 | 14.9937802 | 2.39E-43 | 8.85E-42 | 87.56925531 |
| SEC14L6 | -1.826450907 | 1.4356359 | -14.99344795 | 2.40E-43 | 8.87E-42 | 87.56564203 |
| SGCA | -1.522308136 | 1.414294753 | -14.98814707 | 2.54E-43 | 9.38E-42 | 87.50799889 |
| TPX2 | 2.539141511 | 3.378428801 | 14.98341604 | 2.67E-43 | 9.86E-42 | 87.45656025 |
| HSD17B6 | -2.811731707 | 2.94540303 | -14.98303891 | 2.68E-43 | 9.88E-42 | 87.45246026 |
| TRAF4 | 1.272444237 | 4.482126783 | 14.97718813 | 2.86E-43 | 1.05E-41 | 87.38885788 |
| TBX5-AS1 | -1.171848645 | 1.244492825 | -14.9762143 | 2.89E-43 | 1.06E-41 | 87.3782727 |
| EZH2 | 1.565414745 | 2.219497678 | 14.96979344 | 3.10E-43 | 1.14E-41 | 87.30848809 |
| RASGRF1 | -1.901405732 | 1.253682121 | -14.96150098 | 3.40E-43 | 1.24E-41 | 87.2183821 |
| MFNG | -1.357383062 | 2.727152294 | -14.95334688 | 3.71E-43 | 1.35E-41 | 87.12980166 |
| RSPO4 | -1.446663442 | 0.855077721 | -14.95047875 | 3.83E-43 | 1.39E-41 | 87.09864943 |
| CDH13 | -1.128937389 | 1.039011327 | -14.94763289 | 3.95E-43 | 1.43E-41 | 87.06774178 |
| C1orf162 | -1.667870736 | 3.049758007 | -14.92437632 | 5.09E-43 | 1.85E-41 | 86.81526253 |
| KLF4 | -2.233207203 | 3.213468133 | -14.91727507 | 5.50E-43 | 1.99E-41 | 86.73820509 |
| MYADM | -1.829213792 | 5.248833113 | -14.89387247 | 7.10E-43 | 2.55E-41 | 86.48437575 |
| RP3-425C14.4 | -1.198630303 | 1.958698113 | -14.88467913 | 7.85E-43 | 2.82E-41 | 86.38471254 |
| SFRP5 | -1.562621028 | 0.683985359 | -14.86817991 | 9.40E-43 | 3.37E-41 | 86.20591829 |
| BMPER | -1.119151192 | 0.556514936 | -14.86437216 | 9.79E-43 | 3.50E-41 | 86.16466842 |
| CCNB2 | 1.928191012 | 2.581575788 | 14.86101308 | 1.02E-42 | 3.62E-41 | 86.12828302 |
| RNF144B | -1.567893748 | 2.968322654 | -14.85338418 | 1.10E-42 | 3.93E-41 | 86.04566104 |
| CBX3 | 1.068838897 | 5.399431311 | 14.85230142 | 1.12E-42 | 3.97E-41 | 86.03393619 |
| HSPB8 | -2.009173634 | 3.289297862 | -14.83287674 | 1.38E-42 | 4.88E-41 | 85.8236594 |
| MDK | 2.676714669 | 6.448615088 | 14.83197745 | 1.39E-42 | 4.92E-41 | 85.81392746 |
| RRM2 | 2.216015994 | 3.000892808 | 14.82947023 | 1.43E-42 | 5.05E-41 | 85.78679612 |
| COL10A1 | 2.927516439 | 3.188917823 | 14.82833565 | 1.45E-42 | 5.10E-41 | 85.77451921 |
| JDP2 | -1.110384224 | 2.562856316 | -14.82132461 | 1.56E-42 | 5.48E-41 | 85.69866475 |
| TOM1L2 | -1.174460145 | 2.841622609 | -14.81338037 | 1.71E-42 | 5.97E-41 | 85.61273357 |
| SOX4 | 1.620427574 | 5.120059341 | 14.8096525 | 1.78E-42 | 6.20E-41 | 85.57241729 |
| SPAG5 | 1.830335362 | 2.357376323 | 14.80091565 | 1.95E-42 | 6.81E-41 | 85.47794804 |
| PID1 | -1.869931374 | 2.408037026 | -14.7889891 | 2.22E-42 | 7.73E-41 | 85.34903096 |
| GIMAP7 | -1.753116685 | 3.482641046 | -14.78483203 | 2.33E-42 | 8.08E-41 | 85.30410747 |
| IL1RL1 | -1.916591679 | 0.944025363 | -14.76755019 | 2.81E-42 | 9.72E-41 | 85.11741304 |
| AC004540.4 | -1.019237805 | 0.690933071 | -14.76741165 | 2.81E-42 | 9.72E-41 | 85.11591682 |
| PLK1 | 1.847478612 | 2.283708418 | 14.76398119 | 2.92E-42 | 1.01E-40 | 85.07887002 |
| PPP1R14A | -1.753783479 | 2.541651881 | -14.76333734 | 2.94E-42 | 1.01E-40 | 85.0719172 |
| FERMT2 | -1.188004582 | 2.759890592 | -14.75613467 | 3.18E-42 | 1.09E-40 | 84.99414742 |
| C16orf59 | 1.336442657 | 1.446151427 | 14.73806597 | 3.87E-42 | 1.33E-40 | 84.79913059 |
| PVRL4 | 1.977862098 | 3.946497022 | 14.70141405 | 5.75E-42 | 1.96E-40 | 84.40388478 |
| SNRPE | 1.120316639 | 4.99280397 | 14.68684759 | 6.73E-42 | 2.29E-40 | 84.24693069 |
| HIST1H2BD | 2.136579484 | 4.134669579 | 14.68198859 | 7.10E-42 | 2.41E-40 | 84.19459097 |
| TMOD1 | -1.075829814 | 0.993618884 | -14.67152052 | 7.95E-42 | 2.70E-40 | 84.08185953 |
| S1PR4 | -1.48910725 | 2.201987784 | -14.64116753 | 1.10E-41 | 3.72E-40 | 83.7551986 |
| BIRC5 | 2.352801942 | 2.912087498 | 14.63920231 | 1.13E-41 | 3.79E-40 | 83.73405972 |
| LPHN2 | -1.687345833 | 2.544338037 | -14.63482556 | 1.18E-41 | 3.96E-40 | 83.68698595 |
| PLEK2 | 1.95033721 | 3.185312516 | 14.63306516 | 1.21E-41 | 4.03E-40 | 83.66805403 |
| ITPRIP | -1.335477443 | 2.595782892 | -14.60796736 | 1.58E-41 | 5.27E-40 | 83.39825994 |
| PHKA1 | 1.128551757 | 2.169348647 | 14.59367659 | 1.84E-41 | 6.14E-40 | 83.24473575 |
| E2F3 | 1.118123037 | 2.631057808 | 14.59039566 | 1.91E-41 | 6.35E-40 | 83.20949905 |
| GUCY1A2 | -1.12891651 | 0.867041399 | -14.59034177 | 1.91E-41 | 6.35E-40 | 83.20892035 |
| SPARCL1 | -1.970048727 | 5.527496756 | -14.55798644 | 2.71E-41 | 8.92E-40 | 82.86163033 |
| VSIG4 | -2.534465718 | 4.335760733 | -14.55372751 | 2.84E-41 | 9.32E-40 | 82.81594385 |
| SAPCD2 | 1.804253176 | 2.003045708 | 14.5513505 | 2.91E-41 | 9.55E-40 | 82.7904478 |
| KIAA1683 | -1.22188076 | 0.814523317 | -14.54140045 | 3.24E-41 | 1.06E-39 | 82.68374421 |
| SLC2A1 | 2.680957427 | 4.350062438 | 14.53685453 | 3.40E-41 | 1.11E-39 | 82.63500562 |
| SLC39A11 | 1.188085622 | 3.608583561 | 14.53037777 | 3.65E-41 | 1.19E-39 | 82.56557813 |
| PAK1 | 1.084166185 | 3.760512895 | 14.52584193 | 3.83E-41 | 1.25E-39 | 82.5169652 |
| MCM4 | 1.741173592 | 3.463221453 | 14.50683887 | 4.70E-41 | 1.53E-39 | 82.31337776 |
| NPM3 | 1.522026166 | 4.37573409 | 14.50475867 | 4.81E-41 | 1.56E-39 | 82.29109941 |
| TK1 | 2.243044597 | 4.501596072 | 14.49951379 | 5.09E-41 | 1.65E-39 | 82.2349351 |
| WNT7A | -1.461983562 | 0.650546777 | -14.46408955 | 7.45E-41 | 2.41E-39 | 81.85585187 |
| CEP55 | 1.992477205 | 2.544707655 | 14.46056737 | 7.74E-41 | 2.49E-39 | 81.81818444 |
| GAS6 | -1.81437765 | 4.087588834 | -14.43742509 | 9.93E-41 | 3.19E-39 | 81.57080207 |
| C11orf80 | 1.145389901 | 2.698886933 | 14.43716926 | 9.95E-41 | 3.20E-39 | 81.56806843 |
| B4GALT2 | 1.14318947 | 3.979443851 | 14.42157575 | 1.18E-40 | 3.77E-39 | 81.40148831 |
| ADAMTSL4 | -1.711457925 | 2.275669686 | -14.40896148 | 1.35E-40 | 4.31E-39 | 81.2667977 |
| GPT2 | 2.158575588 | 2.972002242 | 14.39056124 | 1.64E-40 | 5.25E-39 | 81.07042849 |
| EPN3 | 1.668020044 | 2.040371528 | 14.38131733 | 1.81E-40 | 5.78E-39 | 80.97182224 |
| NDRG4 | -1.234720691 | 0.981405571 | -14.37960764 | 1.85E-40 | 5.88E-39 | 80.95358805 |
| C10orf128 | -1.286214247 | 1.942511018 | -14.35968035 | 2.29E-40 | 7.26E-39 | 80.74113704 |
| ERO1L | 1.723468338 | 4.59900771 | 14.35595854 | 2.38E-40 | 7.54E-39 | 80.70147344 |
| ANXA3 | -1.986160107 | 3.455518826 | -14.34775101 | 2.60E-40 | 8.22E-39 | 80.61402287 |
| PGM5 | -1.369372536 | 1.225513629 | -14.34366423 | 2.71E-40 | 8.57E-39 | 80.57048757 |
| ASF1B | 1.732588557 | 2.847133433 | 14.33065581 | 3.12E-40 | 9.82E-39 | 80.43195273 |
| DOK2 | -1.798356374 | 3.018899348 | -14.30481371 | 4.11E-40 | 1.29E-38 | 80.15692529 |
| MYL9 | -1.793575216 | 5.756829661 | -14.30464337 | 4.12E-40 | 1.29E-38 | 80.1551132 |
| CXorf36 | -1.132176186 | 1.588508534 | -14.30213747 | 4.23E-40 | 1.33E-38 | 80.1284569 |
| PLA2G1B | -2.958529262 | 2.085542719 | -14.29406215 | 4.62E-40 | 1.44E-38 | 80.04257186 |
| FIBIN | -1.92729342 | 2.304526619 | -14.28975822 | 4.83E-40 | 1.51E-38 | 79.99680704 |
| CH17-360D5.3 | -1.550444309 | 0.754661818 | -14.2881514 | 4.92E-40 | 1.53E-38 | 79.97972301 |
| CAB39L | -1.049578166 | 1.843715278 | -14.2833243 | 5.18E-40 | 1.61E-38 | 79.92840611 |
| QKI | -1.163692417 | 2.853572064 | -14.27901463 | 5.42E-40 | 1.68E-38 | 79.88259709 |
| DOCK4 | -1.229165409 | 1.910928964 | -14.25035089 | 7.36E-40 | 2.28E-38 | 79.57809209 |
| ENG | -1.564771353 | 5.227925132 | -14.23364317 | 8.80E-40 | 2.72E-38 | 79.40073845 |
| FBLN5 | -1.865399482 | 3.29910265 | -14.22507767 | 9.64E-40 | 2.97E-38 | 79.30985473 |
| KIF2C | 2.006595897 | 2.388720124 | 14.21573993 | 1.07E-39 | 3.28E-38 | 79.21080776 |
| DCAF13 | 1.068696459 | 2.69428824 | 14.21069609 | 1.12E-39 | 3.45E-38 | 79.15732022 |
| ARHGEF16 | 1.341540588 | 2.793985394 | 14.20447079 | 1.20E-39 | 3.68E-38 | 79.09131675 |
| PSMG3 | 1.386738567 | 4.214273381 | 14.18432515 | 1.49E-39 | 4.55E-38 | 78.87782115 |
| KIF11 | 1.658799922 | 2.341040636 | 14.18355864 | 1.50E-39 | 4.58E-38 | 78.86970089 |
| ANLN | 2.165767274 | 2.569217843 | 14.17943498 | 1.57E-39 | 4.78E-38 | 78.8260196 |
| PDK4 | -2.424056824 | 3.248155643 | -14.16851831 | 1.76E-39 | 5.36E-38 | 78.71041125 |
| TMEM150B | -1.389124953 | 1.513987229 | -14.16716168 | 1.79E-39 | 5.41E-38 | 78.69604743 |
| GATA2 | -1.445909186 | 1.951264067 | -14.16713649 | 1.79E-39 | 5.41E-38 | 78.6957808 |
| RECQL4 | 1.781235112 | 2.352673013 | 14.16376897 | 1.85E-39 | 5.59E-38 | 78.66012912 |
| GALNT7 | 1.420315022 | 3.43070864 | 14.16043223 | 1.92E-39 | 5.79E-38 | 78.62480736 |
| AC079630.2 | -1.788542239 | 0.905241472 | -14.15693195 | 1.99E-39 | 6.00E-38 | 78.58775891 |
| LHFPL3-AS2 | -2.271257246 | 1.45156469 | -14.15060119 | 2.13E-39 | 6.40E-38 | 78.52076297 |
| TMEM47 | -1.53307012 | 2.530152597 | -14.1426364 | 2.32E-39 | 6.96E-38 | 78.43649591 |
| FABP5 | -1.901911703 | 2.872503206 | -14.13864334 | 2.42E-39 | 7.25E-38 | 78.39425832 |
| IL33 | -2.261816773 | 2.979042222 | -14.13285248 | 2.58E-39 | 7.68E-38 | 78.33301463 |
| TINCR | -1.32591867 | 0.717707518 | -14.13004128 | 2.65E-39 | 7.91E-38 | 78.30328805 |
| GPC3 | -2.603369183 | 3.194505591 | -14.11241291 | 3.20E-39 | 9.49E-38 | 78.11694699 |
| CHRDL1 | -2.388691585 | 2.332412492 | -14.11062343 | 3.26E-39 | 9.66E-38 | 78.09803772 |
| EP300-AS1 | -1.188237751 | 1.195453396 | -14.08872453 | 4.12E-39 | 1.22E-37 | 77.86673062 |
| HSPD1 | 1.23749488 | 6.106660384 | 14.08377226 | 4.34E-39 | 1.28E-37 | 77.81444722 |
| KIAA0101 | 1.617639432 | 2.134518131 | 14.08323788 | 4.36E-39 | 1.29E-37 | 77.80880598 |
| FRY | -1.083923722 | 1.50238001 | -14.08062745 | 4.49E-39 | 1.32E-37 | 77.78125062 |
| CACNA2D2 | -3.040078901 | 2.810650088 | -14.07221128 | 4.91E-39 | 1.44E-37 | 77.69242808 |
| MYBL2 | 2.764884472 | 3.442343121 | 14.06933257 | 5.06E-39 | 1.48E-37 | 77.66205283 |
| OLFML1 | -1.301367016 | 1.944717521 | -14.06412628 | 5.34E-39 | 1.56E-37 | 77.60712562 |
| ACOXL | -1.227104988 | 0.93003739 | -14.06240648 | 5.44E-39 | 1.59E-37 | 77.5889836 |
| CSF3 | -2.174523977 | 0.927352319 | -14.05866844 | 5.66E-39 | 1.65E-37 | 77.54955532 |
| RP6-65G23.3 | 1.73235463 | 2.358295565 | 14.05384662 | 5.96E-39 | 1.73E-37 | 77.49870322 |
| LINC00511 | 1.419810266 | 1.660737553 | 14.03807044 | 7.05E-39 | 2.04E-37 | 77.33238488 |
| CTNNAL1 | -1.256267831 | 3.679626374 | -14.03786584 | 7.06E-39 | 2.05E-37 | 77.33022854 |
| CALCOCO1 | -1.081555899 | 3.296415808 | -14.01790091 | 8.72E-39 | 2.52E-37 | 77.11988675 |
| FERMT1 | 1.829760804 | 2.313107428 | 14.01374808 | 9.12E-39 | 2.63E-37 | 77.07615323 |
| BMPR2 | -1.10305261 | 3.387908835 | -14.01197268 | 9.29E-39 | 2.67E-37 | 77.05745847 |
| RBMS2 | -1.084812888 | 3.193879279 | -13.9788005 | 1.32E-38 | 3.79E-37 | 76.70837822 |
| NEK2 | 1.885262216 | 2.135184533 | 13.97088367 | 1.44E-38 | 4.11E-37 | 76.62512887 |
| DUOXA1 | -2.037354242 | 2.071995421 | -13.95620613 | 1.68E-38 | 4.78E-37 | 76.47085029 |
| RBP4 | -2.379740353 | 1.921546324 | -13.94561621 | 1.87E-38 | 5.34E-37 | 76.35958856 |
| AMICA1 | -1.463100059 | 1.959937247 | -13.94420491 | 1.90E-38 | 5.41E-37 | 76.34476411 |
| P3H4 | 1.601327006 | 3.240954909 | 13.94113269 | 1.97E-38 | 5.57E-37 | 76.31249586 |
| KDELR2 | 1.022521397 | 6.473576349 | 13.93981072 | 1.99E-38 | 5.64E-37 | 76.29861196 |
| SRPX | -1.73807464 | 2.268054334 | -13.93085245 | 2.19E-38 | 6.19E-37 | 76.20454622 |
| HN1 | 1.533173633 | 5.235266394 | 13.92717926 | 2.28E-38 | 6.43E-37 | 76.16598492 |
| TMPRSS4 | 2.689802761 | 2.887551367 | 13.91838837 | 2.50E-38 | 7.04E-37 | 76.07371887 |
| PSAT1 | 2.311330001 | 3.108055159 | 13.91270008 | 2.65E-38 | 7.47E-37 | 76.01403238 |
| UHRF1 | 1.507554947 | 1.713802387 | 13.90143743 | 2.99E-38 | 8.40E-37 | 75.89589145 |
| MS4A15 | -3.332632027 | 2.104190096 | -13.89595855 | 3.17E-38 | 8.87E-37 | 75.83843762 |
| GATA6 | -1.695671167 | 2.131351274 | -13.89195264 | 3.30E-38 | 9.24E-37 | 75.7964373 |
| KIF26B | 1.534275666 | 1.810985156 | 13.86909682 | 4.20E-38 | 1.17E-36 | 75.55692154 |
| CRABP2 | 4.159336137 | 5.665003573 | 13.86799382 | 4.25E-38 | 1.19E-36 | 75.54536779 |
| GNAQ | -1.064801529 | 3.924200608 | -13.86620926 | 4.33E-38 | 1.21E-36 | 75.52667587 |
| ARHGAP44 | -1.375558048 | 1.804700368 | -13.86510518 | 4.38E-38 | 1.22E-36 | 75.515112 |
| MRC1 | -2.617750841 | 3.920247306 | -13.85863669 | 4.69E-38 | 1.30E-36 | 75.44737247 |
| PRC1 | 1.62412304 | 2.450978603 | 13.83281291 | 6.16E-38 | 1.71E-36 | 75.17710095 |
| PTPLAD2 | -1.104136349 | 1.750229571 | -13.82927167 | 6.39E-38 | 1.77E-36 | 75.14005853 |
| FKBP11 | 1.447582116 | 3.224206884 | 13.8111163 | 7.74E-38 | 2.13E-36 | 74.95022411 |
| MT1M | -2.187759657 | 1.837593506 | -13.79687819 | 8.98E-38 | 2.47E-36 | 74.80143854 |
| CFL2 | -1.165070395 | 2.337575665 | -13.78799157 | 9.86E-38 | 2.71E-36 | 74.70861508 |
| PLEKHN1 | 1.380456474 | 1.614212684 | 13.77803765 | 1.10E-37 | 3.01E-36 | 74.60467994 |
| GINS1 | 1.558603125 | 1.932833286 | 13.7731624 | 1.15E-37 | 3.16E-36 | 74.55378854 |
| LDHA | 1.308836099 | 6.843598137 | 13.76160965 | 1.30E-37 | 3.56E-36 | 74.43322942 |
| KIF4A | 1.813847725 | 1.997748173 | 13.75904139 | 1.34E-37 | 3.65E-36 | 74.40643538 |
| HYAL2 | -1.074264739 | 4.102224125 | -13.75744374 | 1.36E-37 | 3.70E-36 | 74.38976881 |
| SLC25A10 | 1.254068288 | 1.913352703 | 13.75735699 | 1.36E-37 | 3.70E-36 | 74.38886388 |
| DONSON | 1.0811158 | 2.290972282 | 13.75716432 | 1.36E-37 | 3.71E-36 | 74.386854 |
| SHC3 | -1.123661893 | 0.974621057 | -13.75334893 | 1.42E-37 | 3.85E-36 | 74.34705649 |
| FZD4 | -1.371243917 | 2.38041916 | -13.74167692 | 1.60E-37 | 4.35E-36 | 74.22534382 |
| ICAM2 | -1.139015188 | 2.204686202 | -13.73829111 | 1.66E-37 | 4.50E-36 | 74.19004746 |
| AGTR1 | -1.070792607 | 0.670661167 | -13.73200295 | 1.78E-37 | 4.80E-36 | 74.12450655 |
| UQCC2 | 1.202167193 | 3.122659831 | 13.73082397 | 1.80E-37 | 4.85E-36 | 74.11221994 |
| CDC6 | 1.703537448 | 1.996451253 | 13.72964908 | 1.82E-37 | 4.90E-36 | 74.09997648 |
| MVB12B | -1.208226829 | 2.092836641 | -13.71209265 | 2.19E-37 | 5.87E-36 | 73.91708627 |
| AC079467.1 | -1.507156067 | 0.69653199 | -13.70751605 | 2.29E-37 | 6.14E-36 | 73.86943047 |
| TIMP3 | -1.551054401 | 1.740169073 | -13.69805242 | 2.53E-37 | 6.77E-36 | 73.77091273 |
| SYNPO2 | -1.18516431 | 1.194969454 | -13.69222033 | 2.69E-37 | 7.18E-36 | 73.71021732 |
| LPL | -2.698309621 | 3.195426696 | -13.68839469 | 2.80E-37 | 7.47E-36 | 73.67041074 |
| KLF9 | -1.465833837 | 3.558040656 | -13.682714 | 2.97E-37 | 7.90E-36 | 73.61131259 |
| EFNA3 | 1.409326923 | 1.713140831 | 13.66777307 | 3.48E-37 | 9.21E-36 | 73.45593784 |
| DLGAP5 | 1.802477942 | 2.031865897 | 13.66360386 | 3.63E-37 | 9.61E-36 | 73.41259694 |
| CCDC69 | -1.506185577 | 3.339069556 | -13.65810787 | 3.85E-37 | 1.02E-35 | 73.35547406 |
| CCNA2 | 1.846691045 | 2.59668697 | 13.65094849 | 4.15E-37 | 1.09E-35 | 73.28108062 |
| PDE5A | -1.246204758 | 1.686618719 | -13.64326825 | 4.49E-37 | 1.18E-35 | 73.20129757 |
| KIAA0907 | 1.279568143 | 3.271824308 | 13.64273956 | 4.52E-37 | 1.19E-35 | 73.19580629 |
| KIAA0040 | -1.068593205 | 3.525318447 | -13.63895961 | 4.70E-37 | 1.23E-35 | 73.15654925 |
| SORBS1 | -1.212289945 | 1.741762426 | -13.63512629 | 4.89E-37 | 1.28E-35 | 73.11674365 |
| HSPE1 | 1.226442618 | 5.277283806 | 13.62275118 | 5.57E-37 | 1.45E-35 | 72.98827935 |
| KPNA2 | 1.749619262 | 4.745146692 | 13.62252605 | 5.58E-37 | 1.46E-35 | 72.98594287 |
| KLF13 | -1.229201167 | 3.516263805 | -13.61822912 | 5.84E-37 | 1.52E-35 | 72.9413517 |
| SLC46A2 | -1.960554628 | 1.391809652 | -13.59811506 | 7.20E-37 | 1.87E-35 | 72.73271704 |
| DPYSL2 | -1.599277508 | 4.50962535 | -13.59673902 | 7.30E-37 | 1.89E-35 | 72.71844986 |
| BUB1B | 1.56814191 | 1.79294256 | 13.57841358 | 8.84E-37 | 2.28E-35 | 72.52851902 |
| ILF2 | 1.076586924 | 6.533062883 | 13.56352354 | 1.03E-36 | 2.66E-35 | 72.37429294 |
| CYBRD1 | -1.938283632 | 4.62931425 | -13.55071906 | 1.18E-36 | 3.03E-35 | 72.24173965 |
| AC008268.1 | -2.84778911 | 1.653390842 | -13.54924279 | 1.20E-36 | 3.07E-35 | 72.22646141 |
| OGN | -1.853064939 | 1.313878385 | -13.54808741 | 1.21E-36 | 3.11E-35 | 72.21450476 |
| DBNDD1 | 1.435872238 | 2.692081412 | 13.547339 | 1.22E-36 | 3.13E-35 | 72.20675999 |
| SAMD10 | 1.231285359 | 2.431045052 | 13.54570742 | 1.24E-36 | 3.18E-35 | 72.18987663 |
| CD300LF | -1.618301417 | 2.3063918 | -13.54505272 | 1.25E-36 | 3.19E-35 | 72.18310222 |
| HYAL1 | -2.185239348 | 2.459266997 | -13.54062221 | 1.31E-36 | 3.34E-35 | 72.13726262 |
| IGSF9 | 1.712188112 | 2.015994319 | 13.52811211 | 1.49E-36 | 3.79E-35 | 72.00787163 |
| PREX2 | -1.024453403 | 0.818333482 | -13.52752698 | 1.50E-36 | 3.81E-35 | 72.00182124 |
| PCDH17 | -1.263804675 | 1.839895443 | -13.52741117 | 1.50E-36 | 3.81E-35 | 72.00062376 |
| ECT2 | 1.60843722 | 3.053828838 | 13.50553355 | 1.89E-36 | 4.78E-35 | 71.77450344 |
| AURKB | 1.941847648 | 2.336856829 | 13.50303095 | 1.94E-36 | 4.90E-35 | 71.74864971 |
| TPPP3 | -2.696450346 | 3.612739471 | -13.49673755 | 2.07E-36 | 5.22E-35 | 71.68364532 |
| CLEC12A | -1.350458909 | 1.256063093 | -13.49576056 | 2.09E-36 | 5.27E-35 | 71.67355554 |
| RTN1 | -1.054169232 | 1.11003892 | -13.49219779 | 2.17E-36 | 5.46E-35 | 71.63676431 |
| A2M | -2.028125221 | 7.300573049 | -13.48835497 | 2.26E-36 | 5.68E-35 | 71.59708694 |
| CTD-2562J17.6 | -1.861167085 | 3.254169203 | -13.48507416 | 2.34E-36 | 5.86E-35 | 71.56321709 |
| RASL12 | -1.33059614 | 2.433670712 | -13.48401114 | 2.36E-36 | 5.92E-35 | 71.55224377 |
| MELK | 1.860521454 | 2.144589151 | 13.47573378 | 2.57E-36 | 6.45E-35 | 71.46681451 |
| LMOD1 | -1.582382725 | 2.383421716 | -13.47261036 | 2.66E-36 | 6.65E-35 | 71.43458537 |
| ACP5 | -1.769180454 | 5.263776198 | -13.47167927 | 2.68E-36 | 6.70E-35 | 71.42497871 |
| ARAP3 | -1.009379765 | 2.103210067 | -13.46843432 | 2.78E-36 | 6.93E-35 | 71.39150105 |
| CYP4B1 | -3.732675793 | 3.868458396 | -13.46614222 | 2.84E-36 | 7.07E-35 | 71.36785637 |
| P3H2 | -2.216033849 | 2.375866402 | -13.46613585 | 2.84E-36 | 7.07E-35 | 71.36779066 |
| RHBDL2 | 1.45947161 | 1.966464465 | 13.44859346 | 3.41E-36 | 8.47E-35 | 71.18689932 |
| MND1 | 1.214700615 | 1.442299037 | 13.44404499 | 3.57E-36 | 8.87E-35 | 71.1400175 |
| RASGEF1B | -1.056162711 | 1.745886909 | -13.43107647 | 4.09E-36 | 1.01E-34 | 71.00639535 |
| 2-Mar | -1.406285728 | 2.884544681 | -13.42384651 | 4.41E-36 | 1.09E-34 | 70.93193071 |
| HMGA1 | 1.987651408 | 6.290852922 | 13.42302704 | 4.44E-36 | 1.10E-34 | 70.92349199 |
| CCRL2 | -1.266319282 | 1.809806522 | -13.40670286 | 5.26E-36 | 1.30E-34 | 70.75544635 |
| IRAK1 | 1.182950966 | 5.317809412 | 13.39364795 | 6.03E-36 | 1.48E-34 | 70.6211341 |
| LMNB1 | 1.558674618 | 3.459615271 | 13.39147201 | 6.16E-36 | 1.51E-34 | 70.59875438 |
| MIR27A | -1.227579716 | 0.713995862 | -13.39138692 | 6.17E-36 | 1.51E-34 | 70.59787926 |
| SERINC2 | 1.823601117 | 5.395720909 | 13.38765579 | 6.41E-36 | 1.56E-34 | 70.55950878 |
| VARS | 1.121435364 | 4.342345115 | 13.3838858 | 6.67E-36 | 1.62E-34 | 70.52074458 |
| AHNAK | -2.02882347 | 5.008163279 | -13.37972571 | 6.96E-36 | 1.69E-34 | 70.47797592 |
| SCARA5 | -1.733047471 | 1.009604152 | -13.37379637 | 7.40E-36 | 1.80E-34 | 70.41703066 |
| SHROOM4 | -1.365429518 | 1.769109256 | -13.36903299 | 7.77E-36 | 1.88E-34 | 70.3680802 |
| CENPF | 1.762017198 | 2.066370602 | 13.36198833 | 8.36E-36 | 2.02E-34 | 70.29570358 |
| PCAT6 | 1.612578528 | 2.266017423 | 13.36092446 | 8.45E-36 | 2.04E-34 | 70.28477527 |
| MKI67 | 1.837796951 | 2.363298385 | 13.35327541 | 9.15E-36 | 2.21E-34 | 70.20621582 |
| SLC11A1 | -1.539152199 | 2.213093901 | -13.34839566 | 9.62E-36 | 2.32E-34 | 70.15611105 |
| STX1A | 1.641276603 | 2.11785327 | 13.34688074 | 9.77E-36 | 2.35E-34 | 70.14055793 |
| TMEM139 | -1.817362428 | 1.548287405 | -13.32223929 | 1.26E-35 | 3.02E-34 | 69.88770839 |
| FOXM1 | 1.972465674 | 2.491995873 | 13.32069353 | 1.28E-35 | 3.06E-34 | 69.87185543 |
| PHACTR2 | -1.23175878 | 2.18820849 | -13.31650529 | 1.34E-35 | 3.19E-34 | 69.82890708 |
| FGFR2 | -1.678217508 | 2.03699577 | -13.31045273 | 1.42E-35 | 3.39E-34 | 69.76685383 |
| NEBL | -1.49209867 | 2.466815823 | -13.29065865 | 1.75E-35 | 4.14E-34 | 69.56402358 |
| CBX4 | 1.017282443 | 3.878711344 | 13.28885659 | 1.78E-35 | 4.21E-34 | 69.54556592 |
| ALOX5 | -1.812123066 | 3.795347704 | -13.27828531 | 1.98E-35 | 4.67E-34 | 69.43731673 |
| CENPU | 1.52669571 | 2.355776289 | 13.27381269 | 2.08E-35 | 4.88E-34 | 69.39153152 |
| HJURP | 1.76382981 | 1.924532501 | 13.27237333 | 2.11E-35 | 4.95E-34 | 69.37679889 |
| CCNF | 1.130522072 | 1.857525186 | 13.25890469 | 2.42E-35 | 5.67E-34 | 69.23898187 |
| PDCD2L | 1.119526113 | 2.619143132 | 13.25044561 | 2.64E-35 | 6.17E-34 | 69.15246381 |
| ACKR1 | -2.257366857 | 2.541186501 | -13.23749691 | 3.02E-35 | 7.04E-34 | 69.02008463 |
| CHAF1B | 1.059442042 | 1.684252655 | 13.22842472 | 3.31E-35 | 7.70E-34 | 68.92737841 |
| GPR4 | -1.06945086 | 1.69271651 | -13.2254413 | 3.42E-35 | 7.91E-34 | 68.89689929 |
| GAPDH | 1.592566003 | 9.163380547 | 13.22176039 | 3.55E-35 | 8.20E-34 | 68.85929958 |
| PRKCQ | -1.130454778 | 1.339210074 | -13.22166668 | 3.55E-35 | 8.20E-34 | 68.85834241 |
| PTGER4 | -1.400114894 | 2.206591571 | -13.21486437 | 3.81E-35 | 8.77E-34 | 68.78887351 |
| EEF1A1P6 | -1.607521937 | 3.279249275 | -13.20646393 | 4.15E-35 | 9.55E-34 | 68.70311057 |
| P4HB | 1.115311277 | 7.7830883 | 13.20166593 | 4.36E-35 | 1.00E-33 | 68.6541395 |
| C1orf198 | -1.12642834 | 4.56144665 | -13.18493538 | 5.18E-35 | 1.19E-33 | 68.48345437 |
| KCNJ5 | -1.319961955 | 1.149276197 | -13.1834239 | 5.26E-35 | 1.20E-33 | 68.46804009 |
| PROM2 | 1.966316075 | 3.453194689 | 13.18130438 | 5.37E-35 | 1.23E-33 | 68.44642648 |
| TIMELESS | 1.314328586 | 2.925746752 | 13.16962581 | 6.06E-35 | 1.38E-33 | 68.32736977 |
| UBASH3B | -1.11450189 | 1.380946484 | -13.12396969 | 9.67E-35 | 2.20E-33 | 67.86248587 |
| TLCD1 | 1.477281093 | 3.812788316 | 13.12345423 | 9.72E-35 | 2.20E-33 | 67.85724232 |
| CDCA5 | 1.75912665 | 2.331583265 | 13.105806 | 1.16E-34 | 2.63E-33 | 67.67778484 |
| HS6ST2 | 2.233875279 | 2.683271384 | 13.09954841 | 1.24E-34 | 2.80E-33 | 67.61418588 |
| LHFPL3 | -1.108777911 | 0.552811677 | -13.09452729 | 1.31E-34 | 2.94E-33 | 67.56316593 |
| HLA-E | -1.331229619 | 8.229237549 | -13.08307991 | 1.47E-34 | 3.30E-33 | 67.44688876 |
| GAB1 | -1.012761254 | 1.819361627 | -13.07219018 | 1.64E-34 | 3.68E-33 | 67.33632796 |
| RP11-750H9.5 | -1.102161884 | 1.368492252 | -13.068805 | 1.70E-34 | 3.81E-33 | 67.30196943 |
| HMMR | 1.540670201 | 1.907686469 | 13.06071388 | 1.85E-34 | 4.12E-33 | 67.21986705 |
| MYOZ1 | -1.312312211 | 1.110029383 | -13.05619596 | 1.93E-34 | 4.31E-33 | 67.17403496 |
| NUF2 | 1.658083661 | 1.843665058 | 13.04561372 | 2.15E-34 | 4.78E-33 | 67.06671769 |
| PPIF | 1.067730341 | 4.438904373 | 13.04391225 | 2.19E-34 | 4.86E-33 | 67.04946708 |
| FANCI | 1.239247167 | 2.087187902 | 13.04107756 | 2.26E-34 | 5.00E-33 | 67.02073001 |
| SHMT2 | 1.306027773 | 4.310085692 | 13.0356072 | 2.39E-34 | 5.28E-33 | 66.96528323 |
| KLF2 | -1.737480815 | 3.598253781 | -13.03389935 | 2.43E-34 | 5.36E-33 | 66.94797536 |
| AURKA | 1.748272051 | 2.728413095 | 13.03346603 | 2.44E-34 | 5.38E-33 | 66.94358419 |
| SECISBP2L | -1.330376812 | 3.26918069 | -13.0314164 | 2.49E-34 | 5.48E-33 | 66.92281465 |
| TLR8 | -1.34256895 | 1.443832662 | -13.02687313 | 2.61E-34 | 5.74E-33 | 66.87678285 |
| STIL | 1.059306446 | 1.330296705 | 13.0158117 | 2.92E-34 | 6.41E-33 | 66.76474697 |
| LIMCH1 | -1.617817083 | 3.849077276 | -13.01427355 | 2.97E-34 | 6.51E-33 | 66.74917197 |
| E2F8 | 1.185752159 | 1.332468326 | 13.01248583 | 3.02E-34 | 6.62E-33 | 66.7310712 |
| PCYOX1 | -1.017808554 | 4.559268513 | -13.00959023 | 3.11E-34 | 6.81E-33 | 66.701756 |
| PTTG1 | 1.751859699 | 3.250470888 | 13.00812902 | 3.16E-34 | 6.90E-33 | 66.68696405 |
| GARS | 1.014615234 | 4.908493679 | 12.99100861 | 3.76E-34 | 8.19E-33 | 66.51372118 |
| NRN1 | -1.633160791 | 1.962495367 | -12.98959119 | 3.81E-34 | 8.30E-33 | 66.49938381 |
| ORC6 | 1.122023455 | 1.28340987 | 12.97966855 | 4.22E-34 | 9.14E-33 | 66.3990401 |
| SLC25A39 | 1.178794278 | 5.501653164 | 12.97408747 | 4.47E-34 | 9.65E-33 | 66.34261961 |
| PHLDA2 | 2.13177868 | 4.419876311 | 12.96742431 | 4.78E-34 | 1.03E-32 | 66.2752779 |
| FEN1 | 1.33991728 | 3.595378157 | 12.94984238 | 5.71E-34 | 1.23E-32 | 66.09767764 |
| ZFYVE9 | -1.02293101 | 2.198101376 | -12.9426278 | 6.15E-34 | 1.32E-32 | 66.02484004 |
| ID4 | -1.989971199 | 2.869283459 | -12.93687795 | 6.52E-34 | 1.39E-32 | 65.96680649 |
| AC079630.4 | -2.176237062 | 1.675734645 | -12.93154764 | 6.88E-34 | 1.47E-32 | 65.91302021 |
| FAM167A | -1.719197312 | 1.680946943 | -12.92687059 | 7.22E-34 | 1.53E-32 | 65.86583603 |
| SLC25A25 | -1.147882499 | 2.578193488 | -12.91513926 | 8.13E-34 | 1.73E-32 | 65.74752726 |
| NTN4 | -1.683408654 | 3.707772092 | -12.90865564 | 8.68E-34 | 1.84E-32 | 65.68216666 |
| NCAPH | 1.600178733 | 2.016200513 | 12.89729338 | 9.74E-34 | 2.06E-32 | 65.56766966 |
| ALOX5AP | -2.033846884 | 4.471088736 | -12.88625222 | 1.09E-33 | 2.30E-32 | 65.4564627 |
| XPR1 | 1.560171913 | 3.962762434 | 12.88373765 | 1.12E-33 | 2.36E-32 | 65.43114338 |
| GINS2 | 1.456542502 | 1.872748679 | 12.88217962 | 1.14E-33 | 2.40E-32 | 65.41545687 |
| C1orf106 | 1.589756062 | 2.838280964 | 12.87872932 | 1.18E-33 | 2.48E-32 | 65.38072252 |
| ITLN1 | -2.085915613 | 0.745940027 | -12.8786209 | 1.18E-33 | 2.48E-32 | 65.37963115 |
| COL1A1 | 2.782537406 | 7.329985807 | 12.8676042 | 1.32E-33 | 2.77E-32 | 65.26876097 |
| SOCS2 | -1.594679007 | 1.982987993 | -12.86704998 | 1.32E-33 | 2.78E-32 | 65.26318488 |
| KIFC1 | 1.782215888 | 2.637286171 | 12.86133856 | 1.40E-33 | 2.94E-32 | 65.20572862 |
| CDT1 | 1.605350214 | 2.198685897 | 12.85272831 | 1.53E-33 | 3.20E-32 | 65.11913754 |
| ZNF106 | -1.089336684 | 2.802571511 | -12.84586445 | 1.64E-33 | 3.43E-32 | 65.05013307 |
| GFPT1 | 1.084665729 | 4.338187712 | 12.84140965 | 1.72E-33 | 3.58E-32 | 65.0053587 |
| GIMAP4 | -1.453964777 | 3.887473039 | -12.83813791 | 1.77E-33 | 3.70E-32 | 64.97248056 |
| PCDHGC3 | -1.239920844 | 1.910609718 | -12.83254091 | 1.88E-33 | 3.91E-32 | 64.91624671 |
| THBS2 | 2.360437483 | 4.338173885 | 12.83115293 | 1.90E-33 | 3.96E-32 | 64.90230365 |
| NEXN | -1.193832651 | 1.910550837 | -12.82415178 | 2.04E-33 | 4.24E-32 | 64.83198592 |
| DUS1L | 1.101815919 | 4.08609351 | 12.81375356 | 2.27E-33 | 4.71E-32 | 64.72758904 |
| CDCA7 | 1.839576845 | 2.448048964 | 12.80699946 | 2.43E-33 | 5.03E-32 | 64.65980441 |
| PRDX4 | 1.347059906 | 5.763803527 | 12.8043371 | 2.50E-33 | 5.15E-32 | 64.63309033 |
| RP11-93K22.13 | 1.01644963 | 1.029280232 | 12.80358239 | 2.52E-33 | 5.19E-32 | 64.62551813 |
| TNS2 | -1.336848021 | 3.388449681 | -12.80170003 | 2.56E-33 | 5.28E-32 | 64.6066331 |
| BYSL | 1.106205755 | 3.47519141 | 12.79960909 | 2.62E-33 | 5.39E-32 | 64.58565729 |
| SSFA2 | -1.078793773 | 3.755520561 | -12.78829732 | 2.93E-33 | 6.03E-32 | 64.4722141 |
| ARHGEF6 | -1.228979905 | 2.346435232 | -12.7838149 | 3.07E-33 | 6.30E-32 | 64.42727671 |
| CDC45 | 1.548971611 | 1.86499554 | 12.78250975 | 3.11E-33 | 6.38E-32 | 64.41419397 |
| GMFG | -1.443492109 | 3.939323276 | -12.78221667 | 3.12E-33 | 6.39E-32 | 64.41125622 |
| LINC00857 | 1.016628637 | 1.381590989 | 12.77579045 | 3.33E-33 | 6.81E-32 | 64.34685192 |
| MOCS1 | -1.041865008 | 1.824118868 | -12.77551535 | 3.34E-33 | 6.82E-32 | 64.34409524 |
| PROS1 | -1.542887265 | 3.428370288 | -12.77347716 | 3.41E-33 | 6.96E-32 | 64.32367243 |
| NCF2 | -1.64969216 | 3.906883978 | -12.76972009 | 3.54E-33 | 7.22E-32 | 64.28603098 |
| TDRKH | 1.082013849 | 2.702782097 | 12.76931322 | 3.55E-33 | 7.24E-32 | 64.281955 |
| TONSL | 1.20167576 | 1.85884861 | 12.75459777 | 4.12E-33 | 8.38E-32 | 64.13458708 |
| TOX2 | -1.239457552 | 1.650075716 | -12.73904549 | 4.82E-33 | 9.76E-32 | 63.97894401 |
| CA3 | -1.372452197 | 1.126682794 | -12.73741148 | 4.90E-33 | 9.91E-32 | 63.96259757 |
| SCIMP | -1.174422051 | 1.575808767 | -12.73444739 | 5.05E-33 | 1.02E-31 | 63.93294817 |
| PLSCR4 | -1.256751022 | 2.733511606 | -12.72912815 | 5.33E-33 | 1.08E-31 | 63.87975047 |
| RP11-783K16.5 | 1.339486011 | 1.472537208 | 12.72763747 | 5.41E-33 | 1.09E-31 | 63.8648445 |
| GALNT18 | -1.34394762 | 3.143879258 | -12.71482301 | 6.15E-33 | 1.24E-31 | 63.73674786 |
| SPC24 | 1.327629025 | 1.728534521 | 12.71267137 | 6.29E-33 | 1.26E-31 | 63.71524674 |
| IL7R | -1.935611753 | 3.252245995 | -12.70471463 | 6.81E-33 | 1.36E-31 | 63.63575394 |
| FOSB | -3.278542272 | 3.176690223 | -12.70361729 | 6.89E-33 | 1.38E-31 | 63.6247931 |
| MEX3A | 1.865204059 | 2.013356889 | 12.6993633 | 7.19E-33 | 1.44E-31 | 63.58230686 |
| HOXA5 | -1.261150486 | 2.018713462 | -12.69595851 | 7.44E-33 | 1.48E-31 | 63.54830777 |
| NPNT | -2.108240911 | 3.490001817 | -12.6953688 | 7.48E-33 | 1.49E-31 | 63.54241966 |
| TSPAN7 | -2.2644246 | 2.701368727 | -12.69400766 | 7.59E-33 | 1.51E-31 | 63.52882962 |
| CCDC167 | 1.316178751 | 4.518463311 | 12.69384599 | 7.60E-33 | 1.51E-31 | 63.52721552 |
| PVT1 | 1.278351126 | 1.626032829 | 12.69224331 | 7.72E-33 | 1.53E-31 | 63.51121499 |
| TYMS | 1.454126799 | 3.071773786 | 12.68334019 | 8.44E-33 | 1.67E-31 | 63.42235113 |
| GNG11 | -1.80401625 | 3.461607502 | -12.68325804 | 8.45E-33 | 1.67E-31 | 63.42153136 |
| KIF22 | 1.035175555 | 3.496179794 | 12.68088899 | 8.66E-33 | 1.71E-31 | 63.39789147 |
| TXNDC17 | 1.408091727 | 4.084851479 | 12.67835144 | 8.88E-33 | 1.75E-31 | 63.37257302 |
| GTSE1 | 1.338959605 | 1.558630381 | 12.66615595 | 1.00E-32 | 1.97E-31 | 63.2509329 |
| VAMP2 | -1.081375386 | 4.256087372 | -12.66451293 | 1.02E-32 | 2.00E-31 | 63.2345502 |
| RP11-357D18.1 | -1.614303206 | 0.999766438 | -12.65290813 | 1.15E-32 | 2.25E-31 | 63.11887284 |
| DCSTAMP | -1.028241822 | 0.752774986 | -12.6429145 | 1.27E-32 | 2.48E-31 | 63.01930456 |
| UNC5CL | 1.716340531 | 2.191017895 | 12.64137114 | 1.29E-32 | 2.52E-31 | 63.00393178 |
| BRIX1 | 1.037370808 | 3.144914369 | 12.64017363 | 1.30E-32 | 2.54E-31 | 62.99200474 |
| C1QTNF6 | 1.371513363 | 2.12794071 | 12.63932116 | 1.31E-32 | 2.56E-31 | 62.98351457 |
| SKA3 | 1.281526466 | 1.462497108 | 12.63541146 | 1.37E-32 | 2.66E-31 | 62.94458024 |
| FAM49A | -1.173994586 | 2.109137695 | -12.63000494 | 1.44E-32 | 2.81E-31 | 62.89075139 |
| GGTA1P | -1.023483899 | 1.564724231 | -12.61865733 | 1.62E-32 | 3.13E-31 | 62.77781472 |
| GIPC2 | -1.065233992 | 0.804424574 | -12.61299306 | 1.71E-32 | 3.31E-31 | 62.72146315 |
| SLC7A7 | -1.351970165 | 2.991622639 | -12.5886286 | 2.18E-32 | 4.20E-31 | 62.47923758 |
| AARD | -1.283113137 | 0.786309547 | -12.58548316 | 2.25E-32 | 4.32E-31 | 62.44798615 |
| DARS2 | 1.040317221 | 3.288613154 | 12.58446082 | 2.28E-32 | 4.36E-31 | 62.43782963 |
| AATK | -1.528694329 | 1.463632597 | -12.57969062 | 2.39E-32 | 4.57E-31 | 62.39044634 |
| PC | 1.284750114 | 2.670343157 | 12.57680149 | 2.46E-32 | 4.69E-31 | 62.36175302 |
| LRRK2 | -2.454607334 | 2.643476472 | -12.57380251 | 2.53E-32 | 4.83E-31 | 62.33197279 |
| LAD1 | 1.760691774 | 5.157001022 | 12.56590064 | 2.74E-32 | 5.22E-31 | 62.25352612 |
| MS4A2 | -1.217111298 | 1.035667296 | -12.55030642 | 3.20E-32 | 6.08E-31 | 62.09879667 |
| PILRA | -1.39657881 | 2.67720109 | -12.53716837 | 3.65E-32 | 6.92E-31 | 61.96852448 |
| ABCC3 | 2.074942612 | 4.209888539 | 12.52988202 | 3.93E-32 | 7.43E-31 | 61.89630991 |
| TLR4 | -1.397916653 | 2.219968528 | -12.52397858 | 4.16E-32 | 7.87E-31 | 61.83781911 |
| HDGF | 1.018257007 | 6.601826579 | 12.51998352 | 4.33E-32 | 8.18E-31 | 61.79824557 |
| SNHG1 | 1.301655964 | 3.235412723 | 12.50611554 | 4.98E-32 | 9.37E-31 | 61.6609315 |
| GNPNAT1 | 1.201880023 | 3.272690239 | 12.50574068 | 4.99E-32 | 9.39E-31 | 61.65722103 |
| SH3BP5 | -1.127383105 | 2.004355238 | -12.49771471 | 5.41E-32 | 1.02E-30 | 61.57779362 |
| STK39 | 1.315676429 | 3.767021548 | 12.48154986 | 6.35E-32 | 1.19E-30 | 61.41791186 |
| TPBG | 1.281867198 | 2.613289779 | 12.48100539 | 6.39E-32 | 1.20E-30 | 61.41252876 |
| CLIC3 | -2.309678724 | 3.651702943 | -12.47931737 | 6.50E-32 | 1.22E-30 | 61.39584043 |
| SHANK3 | -1.061829737 | 2.110792297 | -12.47866459 | 6.54E-32 | 1.22E-30 | 61.3893872 |
| CYP27A1 | -1.811776637 | 4.376809574 | -12.46239559 | 7.69E-32 | 1.43E-30 | 61.22861913 |
| METTL1 | 1.127213964 | 3.157308152 | 12.45569007 | 8.22E-32 | 1.53E-30 | 61.16239182 |
| COLEC12 | -1.741533808 | 2.673620367 | -12.45329738 | 8.42E-32 | 1.56E-30 | 61.13876539 |
| RALGPS2 | 1.122505335 | 2.109357438 | 12.44493561 | 9.15E-32 | 1.70E-30 | 61.05621862 |
| EFEMP1 | -1.826602601 | 4.719978612 | -12.44308507 | 9.32E-32 | 1.73E-30 | 61.03795469 |
| PTPRM | -1.29560039 | 2.669029757 | -12.44045175 | 9.56E-32 | 1.77E-30 | 61.01196765 |
| NDC80 | 1.460320403 | 1.757818043 | 12.43144331 | 1.05E-31 | 1.93E-30 | 60.92309224 |
| COL3A1 | 2.436971691 | 7.536646697 | 12.43021132 | 1.06E-31 | 1.95E-30 | 60.91094057 |
| CDK1 | 1.685815608 | 2.841638748 | 12.41556004 | 1.22E-31 | 2.25E-30 | 60.76648314 |
| TRGJP2 | -1.532409002 | 0.852661923 | -12.41473218 | 1.23E-31 | 2.27E-30 | 60.75832365 |
| SUSD2 | -3.172754845 | 4.200653603 | -12.4126284 | 1.26E-31 | 2.31E-30 | 60.73758994 |
| PKP3 | 1.382197681 | 4.239787575 | 12.41092128 | 1.28E-31 | 2.35E-30 | 60.72076707 |
| PAPSS2 | -1.566429511 | 4.351482198 | -12.40923723 | 1.30E-31 | 2.39E-30 | 60.70417291 |
| TBX5 | -1.07734814 | 1.589267725 | -12.40690587 | 1.33E-31 | 2.44E-30 | 60.68120244 |
| SEPP1 | -1.810928568 | 4.042156521 | -12.40414797 | 1.37E-31 | 2.51E-30 | 60.65403272 |
| HSH2D | 1.156470205 | 2.741227984 | 12.40185447 | 1.40E-31 | 2.56E-30 | 60.63144076 |
| GJB2 | 2.545444659 | 2.940607088 | 12.39026734 | 1.57E-31 | 2.87E-30 | 60.51734005 |
| MIR3677 | -1.593590419 | 1.29238176 | -12.38039058 | 1.74E-31 | 3.16E-30 | 60.42013126 |
| PKMYT1 | 1.259768555 | 1.483653686 | 12.3786507 | 1.77E-31 | 3.21E-30 | 60.40301183 |
| TIMP1 | 1.570341885 | 7.933550286 | 12.37688963 | 1.80E-31 | 3.27E-30 | 60.38568526 |
| RBMS3 | -1.16020494 | 1.438383106 | -12.36798866 | 1.96E-31 | 3.56E-30 | 60.2981342 |
| SLC52A2 | 1.310360841 | 4.626673652 | 12.36415657 | 2.04E-31 | 3.69E-30 | 60.26045276 |
| NPR3 | -1.318376682 | 1.574488826 | -12.36286532 | 2.06E-31 | 3.73E-30 | 60.24775725 |
| MMP11 | 2.806244069 | 2.941236992 | 12.36258806 | 2.07E-31 | 3.74E-30 | 60.24503134 |
| NDRG2 | -1.430407201 | 2.597959364 | -12.3601469 | 2.12E-31 | 3.83E-30 | 60.22103253 |
| LMO7 | -1.38425853 | 4.110593525 | -12.35751739 | 2.18E-31 | 3.92E-30 | 60.19518515 |
| DTYMK | 1.12956998 | 3.5987758 | 12.35516436 | 2.23E-31 | 4.01E-30 | 60.17205822 |
| KRT80 | 1.880610875 | 3.401212541 | 12.33762407 | 2.65E-31 | 4.74E-30 | 59.9997443 |
| MCM2 | 1.532276287 | 3.243053085 | 12.33269165 | 2.78E-31 | 4.96E-30 | 59.95131485 |
| EPB41L2 | -1.196804784 | 2.43506281 | -12.32787994 | 2.92E-31 | 5.20E-30 | 59.9040817 |
| MIR4653 | 1.541293432 | 1.756592311 | 12.32235861 | 3.08E-31 | 5.48E-30 | 59.84989609 |
| ENO1 | 1.201167528 | 8.68791317 | 12.32234701 | 3.08E-31 | 5.48E-30 | 59.84978222 |
| MIS18A | 1.018038247 | 2.925600319 | 12.31865227 | 3.20E-31 | 5.67E-30 | 59.81353066 |
| APBB1 | -1.19860727 | 2.336363591 | -12.31811323 | 3.21E-31 | 5.70E-30 | 59.80824224 |
| BAIAP2L1 | 1.120315028 | 4.152476839 | 12.30427902 | 3.68E-31 | 6.51E-30 | 59.67256618 |
| PLLP | -1.415507084 | 2.107379196 | -12.27529886 | 4.90E-31 | 8.63E-30 | 59.38864288 |
| MCM6 | 1.249062123 | 3.612273623 | 12.27385692 | 4.97E-31 | 8.75E-30 | 59.37452642 |
| FCGR3B | -1.195916977 | 0.856843315 | -12.27197484 | 5.07E-31 | 8.90E-30 | 59.35610242 |
| HK3 | -1.658603806 | 2.383480501 | -12.27189271 | 5.07E-31 | 8.90E-30 | 59.35529851 |
| BOP1 | 1.409511366 | 3.827151272 | 12.26732308 | 5.31E-31 | 9.28E-30 | 59.3105728 |
| HMGN2P15 | -1.216747107 | 1.034688627 | -12.2672931 | 5.31E-31 | 9.28E-30 | 59.31027945 |
| ABCG1 | -1.291914355 | 2.929202714 | -12.26588599 | 5.38E-31 | 9.40E-30 | 59.2965093 |
| KNTC1 | 1.129996624 | 1.688259362 | 12.26124483 | 5.63E-31 | 9.83E-30 | 59.25109686 |
| SLC27A3 | -1.177203785 | 2.85535295 | -12.25033693 | 6.27E-31 | 1.09E-29 | 59.14440651 |
| FILIP1 | -1.279179015 | 1.382758607 | -12.24708854 | 6.48E-31 | 1.13E-29 | 59.11264487 |
| NEDD9 | -1.659044046 | 3.798142515 | -12.24690084 | 6.49E-31 | 1.13E-29 | 59.11080975 |
| GJA5 | -1.508389676 | 3.110901646 | -12.24332285 | 6.72E-31 | 1.17E-29 | 59.07583155 |
| FAM111B | 1.362070045 | 1.748629601 | 12.23174369 | 7.53E-31 | 1.31E-29 | 58.96267631 |
| EXO1 | 1.310581939 | 1.422812707 | 12.22842809 | 7.78E-31 | 1.35E-29 | 58.93028692 |
| RFC4 | 1.256363204 | 2.595788063 | 12.22169156 | 8.31E-31 | 1.44E-29 | 58.86449552 |
| FAM65A | -1.115161022 | 3.205239694 | -12.21469517 | 8.91E-31 | 1.54E-29 | 58.7961892 |
| ABCA3 | -2.587579523 | 4.598598987 | -12.20467785 | 9.83E-31 | 1.69E-29 | 58.69842996 |
| APOBR | -1.418783781 | 2.450930684 | -12.19512268 | 1.08E-30 | 1.85E-29 | 58.60522566 |
| TROAP | 1.601328457 | 1.74727907 | 12.18494526 | 1.19E-30 | 2.04E-29 | 58.50599981 |
| CTA-384D8.35 | 1.69504461 | 2.361586382 | 12.18015641 | 1.25E-30 | 2.14E-29 | 58.45932757 |
| NOSTRIN | -1.236926553 | 1.770105211 | -12.17410383 | 1.33E-30 | 2.26E-29 | 58.40035483 |
| CDC25C | 1.05953899 | 1.105822991 | 12.15842802 | 1.55E-30 | 2.62E-29 | 58.24770083 |
| C1orf115 | -1.584231348 | 2.869937552 | -12.15211812 | 1.65E-30 | 2.79E-29 | 58.18628722 |
| BUB1 | 1.456548251 | 1.992163367 | 12.14953374 | 1.69E-30 | 2.85E-29 | 58.16113924 |
| DECR2 | 1.02821689 | 2.540873066 | 12.13794497 | 1.89E-30 | 3.18E-29 | 58.04841133 |
| CD83 | -1.446089586 | 3.533364803 | -12.13613813 | 1.93E-30 | 3.24E-29 | 58.03084142 |
| TNFSF12 | -1.141572658 | 3.88346785 | -12.13586188 | 1.93E-30 | 3.24E-29 | 58.02815525 |
| GJA4 | -1.227374413 | 2.566948361 | -12.13564854 | 1.93E-30 | 3.25E-29 | 58.02608085 |
| LLNLR-470E3.1 | -1.043980291 | 1.146386154 | -12.11652105 | 2.33E-30 | 3.90E-29 | 57.8401843 |
| F10 | -1.111488726 | 1.217224466 | -12.11650328 | 2.33E-30 | 3.90E-29 | 57.84001168 |
| VMP1 | 1.278066754 | 5.139771745 | 12.11005362 | 2.49E-30 | 4.15E-29 | 57.7773686 |
| GPRIN1 | 1.216702247 | 1.54375927 | 12.10593206 | 2.59E-30 | 4.32E-29 | 57.73734794 |
| UBL3 | -1.033511128 | 4.178127221 | -12.10240392 | 2.68E-30 | 4.46E-29 | 57.70309604 |
| SLC7A5 | 2.077170813 | 4.151924536 | 12.10132747 | 2.71E-30 | 4.50E-29 | 57.69264684 |
| NCAPG | 1.430202112 | 1.677628661 | 12.10038616 | 2.73E-30 | 4.54E-29 | 57.68350992 |
| RRAS | -1.371761763 | 5.346185177 | -12.09942549 | 2.76E-30 | 4.58E-29 | 57.67418553 |
| JUND | -1.326489102 | 6.135171099 | -12.09845765 | 2.78E-30 | 4.62E-29 | 57.66479201 |
| ALG3 | 1.018837926 | 3.927797716 | 12.09783812 | 2.80E-30 | 4.64E-29 | 57.65877925 |
| RP11-465B22.3 | 1.289480923 | 1.610407688 | 12.09062952 | 3.01E-30 | 4.98E-29 | 57.58883139 |
| PRG4 | -1.847699573 | 1.193530805 | -12.0891804 | 3.05E-30 | 5.04E-29 | 57.57477302 |
| CDKN3 | 1.614662786 | 2.132748667 | 12.08738652 | 3.10E-30 | 5.13E-29 | 57.55737145 |
| GPRIN2 | -1.709374555 | 2.284140341 | -12.0836557 | 3.22E-30 | 5.30E-29 | 57.52118562 |
| BCL9 | 1.099487589 | 3.028296858 | 12.07698375 | 3.43E-30 | 5.65E-29 | 57.45649021 |
| EGR2 | -1.426600472 | 2.234343364 | -12.07456519 | 3.52E-30 | 5.78E-29 | 57.43304358 |
| PPIL1 | 1.007203617 | 4.294564076 | 12.07351567 | 3.55E-30 | 5.83E-29 | 57.42287001 |
| SELPLG | -1.472964556 | 3.9661206 | -12.07202293 | 3.60E-30 | 5.91E-29 | 57.40840093 |
| CCT5 | 1.164637272 | 5.165811855 | 12.06893036 | 3.71E-30 | 6.09E-29 | 57.3784282 |
| PGM2L1 | 1.260318994 | 2.338892105 | 12.06500644 | 3.86E-30 | 6.31E-29 | 57.34040485 |
| ARRB1 | -1.209330871 | 3.41071481 | -12.06496867 | 3.86E-30 | 6.31E-29 | 57.34003893 |
| ST14 | 1.398799406 | 5.904045569 | 12.0636816 | 3.91E-30 | 6.38E-29 | 57.32756863 |
| SLC15A2 | -1.574093837 | 2.273446672 | -12.05918679 | 4.09E-30 | 6.66E-29 | 57.28402544 |
| FAM167B | -1.132578416 | 2.463628445 | -12.0581725 | 4.13E-30 | 6.72E-29 | 57.27420089 |
| PTGES | 2.107135017 | 3.801868259 | 12.04895818 | 4.51E-30 | 7.35E-29 | 57.18497299 |
| SHCBP1 | 1.140363924 | 1.597765043 | 12.04237502 | 4.81E-30 | 7.82E-29 | 57.12124956 |
| KHDRBS2 | -1.26464525 | 0.885645357 | -12.0404835 | 4.90E-30 | 7.96E-29 | 57.10294398 |
| SIX4 | 1.147782816 | 1.725831381 | 12.0388283 | 4.98E-30 | 8.08E-29 | 57.08692693 |
| CENPA | 1.519895493 | 1.685439744 | 12.03721033 | 5.06E-30 | 8.21E-29 | 57.07127141 |
| LAMC3 | -1.310872491 | 1.652862736 | -12.0370691 | 5.07E-30 | 8.21E-29 | 57.06990486 |
| FLRT3 | -1.958000583 | 3.045654103 | -12.03698295 | 5.07E-30 | 8.21E-29 | 57.06907132 |
| MYH10 | -1.387377609 | 3.67021909 | -12.0329993 | 5.27E-30 | 8.52E-29 | 57.03053128 |
| TACC3 | 1.278021068 | 2.877349604 | 12.02164392 | 5.89E-30 | 9.49E-29 | 56.92071573 |
| KIF18B | 1.459117338 | 1.565674683 | 12.01108098 | 6.53E-30 | 1.05E-28 | 56.81862026 |
| PFKP | 1.774518749 | 4.640332947 | 12.00314066 | 7.05E-30 | 1.13E-28 | 56.74190953 |
| ST6GALNAC2 | -1.267726568 | 1.572169181 | -11.99080205 | 7.95E-30 | 1.28E-28 | 56.62276881 |
| NIPSNAP1 | 1.021336319 | 4.605723548 | 11.98977576 | 8.03E-30 | 1.29E-28 | 56.6128624 |
| CKS1B | 1.364363541 | 3.410362935 | 11.98678663 | 8.27E-30 | 1.32E-28 | 56.58401228 |
| CARD16 | -1.170463597 | 2.46808132 | -11.98666926 | 8.28E-30 | 1.32E-28 | 56.58287954 |
| RP11-77A13.1 | -1.367457991 | 0.550588163 | -11.97992209 | 8.84E-30 | 1.41E-28 | 56.51777464 |
| SLC16A11 | -1.188743477 | 1.09912347 | -11.97989888 | 8.84E-30 | 1.41E-28 | 56.51755075 |
| NES | -1.537927777 | 3.203951984 | -11.97853784 | 8.96E-30 | 1.43E-28 | 56.5044205 |
| HDHD3 | 1.05809189 | 3.756014598 | 11.96816699 | 9.91E-30 | 1.58E-28 | 56.4044007 |
| AP1S1 | 1.077009257 | 5.097092248 | 11.95311194 | 1.15E-29 | 1.82E-28 | 56.25929921 |
| PDGFB | -1.240417217 | 2.802282874 | -11.95154175 | 1.16E-29 | 1.85E-28 | 56.24417204 |
| RP11-476D10.1 | -1.331203845 | 0.704942375 | -11.94567861 | 1.23E-29 | 1.95E-28 | 56.18769754 |
| ZEB1 | -1.042006094 | 1.925795257 | -11.94551144 | 1.23E-29 | 1.95E-28 | 56.18608759 |
| FIGNL1 | 1.031006709 | 2.025500002 | 11.92830007 | 1.46E-29 | 2.31E-28 | 56.020405 |
| CDCA3 | 1.171049619 | 1.336145482 | 11.92810554 | 1.46E-29 | 2.31E-28 | 56.01853314 |
| C17orf53 | 1.145417579 | 1.435090163 | 11.92647063 | 1.49E-29 | 2.34E-28 | 56.00280275 |
| RAD54L | 1.184783897 | 1.321464141 | 11.9056763 | 1.82E-29 | 2.85E-28 | 55.80284376 |
| EPHA10 | 1.066325254 | 1.116773776 | 11.89951751 | 1.93E-29 | 3.02E-28 | 55.74366175 |
| MMP19 | -1.450255615 | 2.227308946 | -11.89503644 | 2.01E-29 | 3.15E-28 | 55.70061336 |
| EDN1 | -1.897230741 | 3.535919082 | -11.89064706 | 2.10E-29 | 3.28E-28 | 55.65845556 |
| NME4 | 1.253744785 | 4.411323692 | 11.89058688 | 2.10E-29 | 3.28E-28 | 55.65787757 |
| AFAP1-AS1 | 2.689106592 | 2.586368394 | 11.88613596 | 2.19E-29 | 3.42E-28 | 55.6151386 |
| ZNF385B | -1.914167138 | 1.542227975 | -11.88095717 | 2.31E-29 | 3.59E-28 | 55.56542282 |
| ESRP1 | 1.068024274 | 4.512772505 | 11.86677355 | 2.65E-29 | 4.10E-28 | 55.42932995 |
| SEMA4B | 1.431715955 | 4.553905829 | 11.8633835 | 2.73E-29 | 4.23E-28 | 55.39681706 |
| GPI | 1.060294953 | 5.177551294 | 11.85869706 | 2.86E-29 | 4.42E-28 | 55.35188028 |
| SOX18 | -1.219276242 | 2.148962003 | -11.84862416 | 3.15E-29 | 4.85E-28 | 55.25533155 |
| FPR1 | -1.592559119 | 2.553354921 | -11.83974686 | 3.44E-29 | 5.27E-28 | 55.17028471 |
| MYRF | -1.807375568 | 1.916543422 | -11.82658536 | 3.90E-29 | 5.98E-28 | 55.04426653 |
| SMAD9 | -1.161842006 | 1.275248463 | -11.82099498 | 4.12E-29 | 6.30E-28 | 54.99076622 |
| CRIM1 | -1.293927903 | 3.672671536 | -11.81609847 | 4.32E-29 | 6.59E-28 | 54.94391921 |
| STOM | -1.242498181 | 6.402300164 | -11.81501439 | 4.36E-29 | 6.65E-28 | 54.93354893 |
| TSTA3 | 1.239887472 | 4.75834336 | 11.81057617 | 4.55E-29 | 6.91E-28 | 54.89109937 |
| POLE2 | 1.06889478 | 1.460328816 | 11.8017384 | 4.96E-29 | 7.52E-28 | 54.8065995 |
| DEPDC1B | 1.232351582 | 1.324439831 | 11.79815816 | 5.13E-29 | 7.78E-28 | 54.77237919 |
| CRY2 | -1.037380029 | 3.130060211 | -11.7933208 | 5.38E-29 | 8.14E-28 | 54.7261535 |
| CEACAM21 | -1.051658786 | 1.304159723 | -11.79070981 | 5.51E-29 | 8.34E-28 | 54.70120778 |
| MAD2L1 | 1.316573418 | 1.829159792 | 11.78195004 | 6.00E-29 | 9.05E-28 | 54.61754091 |
| ZNF217 | 1.158267678 | 3.784009405 | 11.77916524 | 6.16E-29 | 9.29E-28 | 54.59095071 |
| POC1A | 1.063934956 | 2.198073935 | 11.77405161 | 6.47E-29 | 9.74E-28 | 54.54213417 |
| C19orf48 | 1.215934947 | 3.61154511 | 11.77279828 | 6.55E-29 | 9.84E-28 | 54.53017144 |
| MFSD2A | -1.752597778 | 3.408085423 | -11.76998779 | 6.73E-29 | 1.01E-27 | 54.50334887 |
| CKAP2L | 1.192963484 | 1.360754295 | 11.76093801 | 7.34E-29 | 1.10E-27 | 54.41700714 |
| CHEK1 | 1.116739932 | 1.619015804 | 11.75580539 | 7.71E-29 | 1.15E-27 | 54.36805655 |
| HPCAL1 | -1.025490615 | 3.797822397 | -11.74848051 | 8.27E-29 | 1.23E-27 | 54.29822092 |
| ATAD2 | 1.31878927 | 2.694746496 | 11.74720052 | 8.38E-29 | 1.25E-27 | 54.28602023 |
| HRCT1 | -1.191326617 | 1.336272273 | -11.73495938 | 9.42E-29 | 1.40E-27 | 54.16938138 |
| LIFR | -1.510030764 | 2.68256365 | -11.72105495 | 1.08E-28 | 1.59E-27 | 54.03698599 |
| APOLD1 | -1.258908915 | 1.962088095 | -11.71276088 | 1.17E-28 | 1.72E-27 | 53.95805804 |
| ORC1 | 1.182304554 | 1.471065194 | 11.71270704 | 1.17E-28 | 1.72E-27 | 53.95754578 |
| ZEB2 | -1.058752265 | 1.689303387 | -11.71154322 | 1.18E-28 | 1.74E-27 | 53.9464735 |
| RAD51 | 1.051638909 | 1.494344292 | 11.70557746 | 1.25E-28 | 1.84E-27 | 53.88972744 |
| PBK | 1.630066823 | 1.93771176 | 11.69317405 | 1.41E-28 | 2.07E-27 | 53.77180456 |
| NCAPG2 | 1.165996393 | 2.217623177 | 11.686843 | 1.49E-28 | 2.19E-27 | 53.71164359 |
| SCD5 | -1.548235248 | 2.227299806 | -11.68638582 | 1.50E-28 | 2.20E-27 | 53.70730002 |
| KCNN4 | 2.261989026 | 3.364609577 | 11.68411388 | 1.53E-28 | 2.24E-27 | 53.68571636 |
| C11orf96 | -1.69341414 | 3.542339881 | -11.6818902 | 1.57E-28 | 2.29E-27 | 53.66459374 |
| BMP2 | -1.779807269 | 2.751266772 | -11.68103576 | 1.58E-28 | 2.31E-27 | 53.65647809 |
| SERPING1 | -1.48755969 | 6.347081128 | -11.67905146 | 1.61E-28 | 2.35E-27 | 53.63763225 |
| SFTPD | -3.615442713 | 6.150905063 | -11.67497612 | 1.67E-28 | 2.44E-27 | 53.59893326 |
| ZNF692 | 1.269976006 | 2.852054641 | 11.66445572 | 1.85E-28 | 2.69E-27 | 53.49907158 |
| FLVCR2 | -1.034007974 | 2.139153523 | -11.6640419 | 1.86E-28 | 2.70E-27 | 53.49514462 |
| GMNN | 1.081129452 | 2.887243139 | 11.66274697 | 1.88E-28 | 2.73E-27 | 53.48285711 |
| IL20RA | -1.312806566 | 1.455594103 | -11.65816953 | 1.97E-28 | 2.85E-27 | 53.43942876 |
| STYK1 | 1.25045099 | 1.680671641 | 11.6495539 | 2.13E-28 | 3.09E-27 | 53.35771734 |
| SPI1 | -1.612119652 | 4.258411015 | -11.64787987 | 2.17E-28 | 3.13E-27 | 53.34184499 |
| BTK | -1.137933494 | 2.02040071 | -11.62130191 | 2.80E-28 | 4.03E-27 | 53.09003893 |
| FAM101B | -1.054108803 | 2.580967425 | -11.61179289 | 3.06E-28 | 4.41E-27 | 53.00003609 |
| CLDN12 | 1.024234716 | 3.434977337 | 11.60624137 | 3.23E-28 | 4.64E-27 | 52.94751244 |
| SFTA1P | -2.791275671 | 3.833948853 | -11.60081962 | 3.40E-28 | 4.88E-27 | 52.8962318 |
| S100A12 | -1.358855966 | 0.945578553 | -11.59446503 | 3.61E-28 | 5.17E-27 | 52.83614731 |
| NR4A3 | -1.622066553 | 1.485421574 | -11.59377045 | 3.63E-28 | 5.20E-27 | 52.82958112 |
| MAOB | -1.606490538 | 2.749985598 | -11.58428087 | 3.98E-28 | 5.68E-27 | 52.73989644 |
| ABLIM3 | -1.243972152 | 1.746878143 | -11.58212012 | 4.06E-28 | 5.80E-27 | 52.71948201 |
| AC006273.5 | -1.077825701 | 1.138945468 | -11.58161497 | 4.08E-28 | 5.82E-27 | 52.71470976 |
| MYH11 | -1.847820432 | 2.624589976 | -11.57146696 | 4.49E-28 | 6.40E-27 | 52.61886762 |
| ADCK5 | 1.139919035 | 2.376406934 | 11.55948139 | 5.04E-28 | 7.15E-27 | 52.50573926 |
| ASPM | 1.248503954 | 1.326359623 | 11.55616778 | 5.20E-28 | 7.36E-27 | 52.47447608 |
| H1F0 | 1.13155815 | 6.912367098 | 11.53974738 | 6.08E-28 | 8.58E-27 | 52.31963713 |
| KIF23 | 1.249241977 | 1.683198707 | 11.53836594 | 6.16E-28 | 8.69E-27 | 52.30661697 |
| SLCO2A1 | -1.77192798 | 3.567825246 | -11.53390614 | 6.42E-28 | 9.05E-27 | 52.26458981 |
| ARHGAP18 | -1.066868671 | 3.655483183 | -11.53118949 | 6.59E-28 | 9.28E-27 | 52.23899434 |
| PYCRL | 1.082193639 | 2.837000682 | 11.52850153 | 6.76E-28 | 9.52E-27 | 52.21367293 |
| TNFSF13 | -1.143158857 | 3.3600766 | -11.526818 | 6.87E-28 | 9.66E-27 | 52.19781555 |
| PPARG | -1.50960666 | 2.2812434 | -11.52597295 | 6.93E-28 | 9.73E-27 | 52.18985643 |
| PLEKHO2 | -1.057217776 | 3.815860733 | -11.52278301 | 7.14E-28 | 1.00E-26 | 52.15981529 |
| WNT3 | 1.04313709 | 1.597065946 | 11.51605972 | 7.61E-28 | 1.07E-26 | 52.09651621 |
| CYGB | -1.048358684 | 2.633687569 | -11.5060979 | 8.36E-28 | 1.17E-26 | 52.00276987 |
| MTHFD2 | 1.337771731 | 3.890941527 | 11.50423189 | 8.51E-28 | 1.19E-26 | 51.98521538 |
| UTRN | -1.248576441 | 3.147654539 | -11.50209416 | 8.69E-28 | 1.21E-26 | 51.96510693 |
| TTYH3 | 1.316366128 | 4.457185562 | 11.49286132 | 9.48E-28 | 1.32E-26 | 51.87828603 |
| CD97 | -1.272808454 | 4.513966037 | -11.48921596 | 9.82E-28 | 1.37E-26 | 51.84401913 |
| SORBS3 | -1.10818836 | 3.63169 | -11.48515522 | 1.02E-27 | 1.42E-26 | 51.80585568 |
| PAQR5 | -1.212147805 | 1.590654633 | -11.47912251 | 1.08E-27 | 1.50E-26 | 51.74917528 |
| ABHD11 | 1.255370883 | 4.262709027 | 11.47145617 | 1.16E-27 | 1.61E-26 | 51.67717349 |
| ZBTB16 | -1.316632051 | 0.857604728 | -11.4608848 | 1.28E-27 | 1.77E-26 | 51.57793813 |
| FHL2 | 1.670996981 | 2.964879182 | 11.45112301 | 1.41E-27 | 1.94E-26 | 51.48635423 |
| FAM83D | 1.512379817 | 2.406507314 | 11.44733193 | 1.46E-27 | 2.00E-26 | 51.45080013 |
| CTB-193M12.5 | 1.057950157 | 2.847205311 | 11.43122406 | 1.70E-27 | 2.32E-26 | 51.29981902 |
| CLEC4E | -1.191838692 | 1.29429742 | -11.4199275 | 1.89E-27 | 2.58E-26 | 51.19401605 |
| TUBB6 | -1.339501447 | 3.624815551 | -11.41939297 | 1.90E-27 | 2.59E-26 | 51.18901135 |
| DTL | 1.153738137 | 1.881909227 | 11.39079595 | 2.49E-27 | 3.37E-26 | 50.92148048 |
| FBP1 | -1.606821355 | 5.84182678 | -11.38740601 | 2.57E-27 | 3.47E-26 | 50.8897955 |
| RASAL1 | 1.300553765 | 1.346874598 | 11.38696705 | 2.58E-27 | 3.48E-26 | 50.88569299 |
| SLC15A3 | -1.270687127 | 3.072602276 | -11.38175929 | 2.71E-27 | 3.66E-26 | 50.83703022 |
| SPC25 | 1.153532181 | 1.6027104 | 11.38025804 | 2.75E-27 | 3.70E-26 | 50.82300468 |
| PPBP | -1.789436033 | 1.08020208 | -11.37817587 | 2.80E-27 | 3.77E-26 | 50.80355399 |
| TTK | 1.253459448 | 1.422911936 | 11.3586258 | 3.37E-27 | 4.51E-26 | 50.62103688 |
| CD302 | -1.231138255 | 2.266247342 | -11.33627334 | 4.16E-27 | 5.52E-26 | 50.41260446 |
| SYT7 | 2.078255161 | 2.854383199 | 11.33364058 | 4.26E-27 | 5.65E-26 | 50.38807189 |
| SFTPA1 | -4.802982374 | 8.353958199 | -11.33299814 | 4.28E-27 | 5.68E-26 | 50.38208603 |
| GADD45B | -1.439921663 | 4.790212547 | -11.3130917 | 5.17E-27 | 6.81E-26 | 50.19671953 |
| P2RY6 | 1.569952074 | 2.040045079 | 11.31283641 | 5.18E-27 | 6.82E-26 | 50.19434371 |
| RAB11FIP1 | -1.40500886 | 3.661779198 | -11.31108661 | 5.26E-27 | 6.92E-26 | 50.17806006 |
| SCGB1A1 | -5.164922464 | 5.08578338 | -11.30471652 | 5.59E-27 | 7.34E-26 | 50.11879367 |
| CA2 | -2.041666643 | 3.113590877 | -11.303555 | 5.65E-27 | 7.41E-26 | 50.10798944 |
| TPPP | -1.535374363 | 1.982556801 | -11.29235203 | 6.27E-27 | 8.20E-26 | 50.00381794 |
| CKAP4 | 1.03149814 | 5.24402882 | 11.29044869 | 6.39E-27 | 8.34E-26 | 49.98612625 |
| NFIX | -1.570237187 | 3.610433171 | -11.28802584 | 6.53E-27 | 8.52E-26 | 49.96360849 |
| CP | 2.727246668 | 4.604727879 | 11.27216327 | 7.58E-27 | 9.86E-26 | 49.81626019 |
| HGH1 | 1.013585545 | 3.584588289 | 11.27160462 | 7.62E-27 | 9.91E-26 | 49.81107328 |
| NUP155 | 1.006724639 | 2.6596041 | 11.25898801 | 8.58E-27 | 1.11E-25 | 49.69397648 |
| CDCA4 | 1.120105023 | 2.915439268 | 11.25253874 | 9.11E-27 | 1.18E-25 | 49.6341526 |
| ADAM8 | 1.629993839 | 3.470738119 | 11.22700858 | 1.16E-26 | 1.48E-25 | 49.39755141 |
| GRTP1 | 1.036242934 | 2.311962799 | 11.21897849 | 1.25E-26 | 1.59E-25 | 49.32320459 |
| CREB3L4 | 1.104173612 | 3.09905767 | 11.20156111 | 1.47E-26 | 1.87E-25 | 49.16206414 |
| FCN1 | -1.387134612 | 1.717027378 | -11.20150182 | 1.47E-26 | 1.87E-25 | 49.16151588 |
| CCNE1 | 1.443666851 | 1.733958291 | 11.19931843 | 1.50E-26 | 1.90E-25 | 49.14132744 |
| SERPINH1 | 1.094956487 | 5.673459611 | 11.19651798 | 1.54E-26 | 1.95E-25 | 49.11543705 |
| DEPDC1 | 1.183661324 | 1.236983254 | 11.1857049 | 1.70E-26 | 2.15E-25 | 49.01550903 |
| MRPL15 | 1.06564973 | 5.254819902 | 11.18342755 | 1.74E-26 | 2.20E-25 | 48.9944712 |
| SSR4 | 1.266110769 | 5.830544258 | 11.18235691 | 1.75E-26 | 2.22E-25 | 48.98458172 |
| NDNF | -2.269587536 | 3.977382967 | -11.18001426 | 1.79E-26 | 2.26E-25 | 48.96294486 |
| TRIP13 | 1.595326267 | 2.382121367 | 11.17700941 | 1.84E-26 | 2.32E-25 | 48.93519616 |
| GMDS | 1.093660001 | 2.812598939 | 11.1756303 | 1.87E-26 | 2.35E-25 | 48.92246224 |
| KIF14 | 1.018312991 | 1.053712477 | 11.16902975 | 1.99E-26 | 2.49E-25 | 48.86153068 |
| PODXL2 | 2.310798461 | 3.824858471 | 11.16861678 | 1.99E-26 | 2.50E-25 | 48.85771919 |
| ATAD3A | 1.003323604 | 3.107271055 | 11.16745286 | 2.01E-26 | 2.52E-25 | 48.84697744 |
| ASNS | 1.2120305 | 3.006861042 | 11.16524721 | 2.06E-26 | 2.57E-25 | 48.82662361 |
| H2AFX | 1.191756343 | 4.553582746 | 11.15444062 | 2.27E-26 | 2.84E-25 | 48.72693764 |
| CPAMD8 | -1.585516953 | 1.753065367 | -11.15288587 | 2.31E-26 | 2.88E-25 | 48.71260102 |
| ELTD1 | -1.010377537 | 2.536382768 | -11.15085384 | 2.35E-26 | 2.92E-25 | 48.69386523 |
| PHLDB2 | -1.163182232 | 1.669901511 | -11.14149678 | 2.56E-26 | 3.19E-25 | 48.60761968 |
| PDIA6 | 1.011270578 | 6.011506696 | 11.13120928 | 2.82E-26 | 3.50E-25 | 48.51285277 |
| ZDHHC9 | 1.211649704 | 5.04001579 | 11.1264282 | 2.95E-26 | 3.65E-25 | 48.46882969 |
| VIM | -1.281627069 | 7.149390767 | -11.12574228 | 2.97E-26 | 3.67E-25 | 48.46251495 |
| FUT8 | 1.147308368 | 3.188313598 | 11.10864236 | 3.48E-26 | 4.30E-25 | 48.30517067 |
| TPI1 | 1.079357348 | 7.466326842 | 11.10365707 | 3.64E-26 | 4.50E-25 | 48.25932861 |
| TSPAN6 | 1.040865988 | 3.813865883 | 11.10266029 | 3.68E-26 | 4.54E-25 | 48.25016435 |
| TUBG1 | 1.041064472 | 4.091904171 | 11.10192818 | 3.70E-26 | 4.56E-25 | 48.24343382 |
| HYOU1 | 1.066900425 | 4.962138236 | 11.09783555 | 3.85E-26 | 4.73E-25 | 48.20581411 |
| MEST | 1.18198389 | 3.775075938 | 11.09569063 | 3.92E-26 | 4.83E-25 | 48.18610145 |
| MTFR1 | 1.00562863 | 3.050271958 | 11.09392617 | 3.99E-26 | 4.90E-25 | 48.16988731 |
| ANKRD22 | 1.88207772 | 2.966695247 | 11.08708367 | 4.25E-26 | 5.21E-25 | 48.10702552 |
| MCM10 | 1.103618461 | 1.182440651 | 11.08608516 | 4.29E-26 | 5.26E-25 | 48.0978544 |
| F2RL3 | -1.073003837 | 1.395180992 | -11.07489594 | 4.76E-26 | 5.83E-25 | 47.99512038 |
| CYS1 | -1.466402186 | 1.441917883 | -11.0735621 | 4.82E-26 | 5.89E-25 | 47.98287834 |
| NET1 | 1.221761856 | 5.130369021 | 11.0706511 | 4.95E-26 | 6.05E-25 | 47.95616424 |
| CDCA2 | 1.003606882 | 1.151597103 | 11.06591194 | 5.17E-26 | 6.31E-25 | 47.9126831 |
| MAFF | -1.013582076 | 2.730887561 | -11.06571933 | 5.18E-26 | 6.32E-25 | 47.91091619 |
| PABPC1 | 1.013243633 | 8.158712567 | 11.03628928 | 6.80E-26 | 8.25E-25 | 47.64117736 |
| HHIP-AS1 | -1.522941426 | 1.4119293 | -11.02212089 | 7.75E-26 | 9.37E-25 | 47.51148698 |
| PMP22 | -1.194928562 | 4.668852758 | -11.00062266 | 9.44E-26 | 1.14E-24 | 47.31491325 |
| COA6 | 1.051081003 | 4.139940595 | 10.99530772 | 9.92E-26 | 1.20E-24 | 47.26635401 |
| TPSAB1 | -1.897745249 | 3.136048239 | -10.99400455 | 1.00E-25 | 1.21E-24 | 47.25445011 |
| SEMA3B | -1.794296636 | 2.600207631 | -10.99385612 | 1.01E-25 | 1.21E-24 | 47.25309439 |
| SFTPA2 | -4.601699105 | 8.611121326 | -10.99346197 | 1.01E-25 | 1.21E-24 | 47.24949417 |
| FUT3 | 1.602574941 | 2.932390566 | 10.97926449 | 1.15E-25 | 1.38E-24 | 47.11987153 |
| STEAP1 | 1.974178571 | 3.264529437 | 10.97028582 | 1.25E-25 | 1.49E-24 | 47.03795374 |
| FBXO32 | 1.45699644 | 2.890235744 | 10.96851527 | 1.27E-25 | 1.52E-24 | 47.02180525 |
| MROH6 | 1.668323813 | 2.951615614 | 10.96444148 | 1.32E-25 | 1.57E-24 | 46.98465624 |
| CEACAM1 | 1.505326053 | 3.105625361 | 10.95793315 | 1.40E-25 | 1.67E-24 | 46.92532557 |
| MRPL24 | 1.010620639 | 5.052549551 | 10.92770893 | 1.85E-25 | 2.18E-24 | 46.65010513 |
| C20orf24 | 1.060943655 | 4.305215627 | 10.92595906 | 1.88E-25 | 2.22E-24 | 46.63418633 |
| SLCO2B1 | -1.44371363 | 3.238914874 | -10.9233082 | 1.92E-25 | 2.27E-24 | 46.61007441 |
| ALG1L | 1.910653924 | 2.507874645 | 10.91874883 | 2.01E-25 | 2.36E-24 | 46.56861198 |
| MATN3 | -1.395960993 | 1.888469816 | -10.90839906 | 2.20E-25 | 2.59E-24 | 46.47453494 |
| DOCK11 | -1.129457527 | 2.26664331 | -10.90735609 | 2.23E-25 | 2.61E-24 | 46.46505791 |
| IGHG4 | 3.265982661 | 6.682023596 | 10.90628005 | 2.25E-25 | 2.64E-24 | 46.45528098 |
| GYG2 | 1.245816834 | 1.867278837 | 10.90467052 | 2.28E-25 | 2.67E-24 | 46.44065795 |
| VPS9D1-AS1 | 1.2769979 | 1.623046095 | 10.9023924 | 2.33E-25 | 2.72E-24 | 46.41996306 |
| PCSK9 | -1.738524388 | 1.45426315 | -10.89670885 | 2.45E-25 | 2.87E-24 | 46.36834515 |
| CHTF18 | 1.033349395 | 1.780397312 | 10.87784878 | 2.92E-25 | 3.39E-24 | 46.19718705 |
| SLFN13 | 1.179122191 | 2.358349641 | 10.87700209 | 2.94E-25 | 3.42E-24 | 46.18950777 |
| APOL3 | -1.22471564 | 2.791618467 | -10.87163764 | 3.09E-25 | 3.58E-24 | 46.14086327 |
| SKA1 | 1.120791536 | 1.307035556 | 10.85712711 | 3.52E-25 | 4.08E-24 | 46.00936288 |
| KCTD12 | -1.374463447 | 4.337057316 | -10.84319984 | 4.00E-25 | 4.62E-24 | 45.88325869 |
| SNX25 | -1.18496811 | 3.079158097 | -10.82260617 | 4.83E-25 | 5.55E-24 | 45.69699243 |
| MNDA | -1.473239322 | 3.209430326 | -10.81975297 | 4.96E-25 | 5.69E-24 | 45.67120451 |
| AK1 | -1.237577689 | 2.870690218 | -10.81828472 | 5.02E-25 | 5.77E-24 | 45.65793583 |
| MSMO1 | -1.029612859 | 4.813061867 | -10.81611644 | 5.13E-25 | 5.88E-24 | 45.63834312 |
| DERL3 | 1.679452902 | 3.037702578 | 10.80595417 | 5.62E-25 | 6.44E-24 | 45.54655164 |
| RP11-12G12.7 | 1.186896065 | 2.541304857 | 10.80028142 | 5.92E-25 | 6.76E-24 | 45.49533719 |
| MAPK4 | -1.040634889 | 0.513229644 | -10.79334916 | 6.31E-25 | 7.19E-24 | 45.4327763 |
| TSPAN12 | -1.281786613 | 3.711745629 | -10.79269045 | 6.34E-25 | 7.23E-24 | 45.42683313 |
| TNS3 | -1.021436987 | 3.870759157 | -10.79094797 | 6.45E-25 | 7.33E-24 | 45.41111277 |
| SPDEF | 2.241364736 | 3.601206938 | 10.79075721 | 6.46E-25 | 7.34E-24 | 45.4093919 |
| IL6 | -1.832514405 | 1.971990115 | -10.77931693 | 7.17E-25 | 8.13E-24 | 45.30622363 |
| KCNJ8 | -1.299740672 | 2.516291924 | -10.77926919 | 7.17E-25 | 8.13E-24 | 45.30579331 |
| PKIG | -1.180245085 | 3.914766105 | -10.77776783 | 7.27E-25 | 8.23E-24 | 45.29225951 |
| ALDH2 | -1.286867314 | 4.519261408 | -10.77304023 | 7.59E-25 | 8.59E-24 | 45.24965171 |
| TMEM132A | 1.434139251 | 2.782629743 | 10.77256226 | 7.62E-25 | 8.62E-24 | 45.2453447 |
| BCL2L15 | 1.466028971 | 1.676112471 | 10.77150912 | 7.69E-25 | 8.70E-24 | 45.23585517 |
| ZFP36 | -1.71318566 | 6.720363492 | -10.76691988 | 8.02E-25 | 9.05E-24 | 45.19451031 |
| CENPM | 1.194526409 | 2.221834429 | 10.75875812 | 8.64E-25 | 9.71E-24 | 45.12100973 |
| AQP1 | -2.533331069 | 6.175048236 | -10.7539773 | 9.02E-25 | 1.01E-23 | 45.07797347 |
| ALDOA | 1.134424853 | 7.566843576 | 10.75132649 | 9.24E-25 | 1.04E-23 | 45.05411687 |
| GYLTL1B | 1.703350829 | 3.105854329 | 10.74239623 | 1.00E-24 | 1.12E-23 | 44.97377603 |
| AC145343.2 | 1.062242913 | 1.229241848 | 10.73979562 | 1.03E-24 | 1.15E-23 | 44.95038814 |
| SULF1 | 1.933513044 | 3.612731915 | 10.72804642 | 1.14E-24 | 1.27E-23 | 44.84477259 |
| CTD-2510F5.4 | 1.471340751 | 2.397783346 | 10.72399279 | 1.18E-24 | 1.32E-23 | 44.80835195 |
| FAM64A | 1.210990892 | 1.399557441 | 10.72154748 | 1.21E-24 | 1.35E-23 | 44.78638611 |
| ESPL1 | 1.055850691 | 1.196756503 | 10.72063296 | 1.22E-24 | 1.36E-23 | 44.77817199 |
| SAMHD1 | -1.181926405 | 4.710233608 | -10.69240411 | 1.58E-24 | 1.75E-23 | 44.52485614 |
| MMP1 | 3.08568443 | 3.732387337 | 10.69079332 | 1.60E-24 | 1.77E-23 | 44.51041506 |
| PTPRH | 1.682694852 | 1.649358738 | 10.67286116 | 1.88E-24 | 2.07E-23 | 44.34974943 |
| SELE | -1.016517831 | 0.697621973 | -10.65711934 | 2.17E-24 | 2.38E-23 | 44.20885921 |
| SLC51B | -1.401454518 | 1.319470785 | -10.65036283 | 2.30E-24 | 2.52E-23 | 44.14843143 |
| SIRPA | -1.218041171 | 3.592825283 | -10.65030351 | 2.31E-24 | 2.52E-23 | 44.14790101 |
| ATOH8 | -1.84999222 | 2.2143603 | -10.64840879 | 2.35E-24 | 2.56E-23 | 44.13096002 |
| MNX1-AS1 | 1.12032877 | 1.066066756 | 10.64158541 | 2.49E-24 | 2.72E-23 | 44.06996811 |
| DUSP1 | -1.788327659 | 7.018555035 | -10.63490575 | 2.65E-24 | 2.88E-23 | 44.01028661 |
| LY86 | -1.378801278 | 3.014561429 | -10.63441378 | 2.66E-24 | 2.90E-23 | 44.00589198 |
| COL5A2 | 1.767578924 | 4.314935611 | 10.61364799 | 3.21E-24 | 3.47E-23 | 43.82052237 |
| YDJC | 1.064083962 | 3.565923873 | 10.56106908 | 5.15E-24 | 5.52E-23 | 43.35227216 |
| SNHG3 | 1.18631213 | 2.83221071 | 10.55761687 | 5.31E-24 | 5.69E-23 | 43.32158348 |
| PTGDS | -2.01066573 | 4.453654731 | -10.55633085 | 5.37E-24 | 5.75E-23 | 43.31015303 |
| FAM134B | -1.319987448 | 1.805309533 | -10.55013307 | 5.68E-24 | 6.06E-23 | 43.25507931 |
| RAD51AP1 | 1.195776849 | 1.971865997 | 10.54978994 | 5.69E-24 | 6.08E-23 | 43.2520309 |
| RASSF8 | -1.158787018 | 2.031935099 | -10.54550758 | 5.92E-24 | 6.31E-23 | 43.21399136 |
| DNAJB4 | -1.049679038 | 2.503903265 | -10.52246513 | 7.27E-24 | 7.70E-23 | 43.00949085 |
| RHBDL1 | 1.294104923 | 1.549416301 | 10.50988792 | 8.14E-24 | 8.60E-23 | 42.8979982 |
| PTGFRN | 1.056276346 | 3.772934624 | 10.50977751 | 8.15E-24 | 8.60E-23 | 42.89701985 |
| C1QA | -1.791237362 | 7.05012996 | -10.49883865 | 8.99E-24 | 9.45E-23 | 42.80012569 |
| PPP1R35 | 1.094835114 | 3.80007504 | 10.49668762 | 9.16E-24 | 9.63E-23 | 42.7810805 |
| RP4-755D9.1 | -1.209283056 | 0.975273109 | -10.49130214 | 9.61E-24 | 1.01E-22 | 42.73340921 |
| CCL24 | -1.499721132 | 1.389368959 | -10.47086763 | 1.15E-23 | 1.20E-22 | 42.5526797 |
| CADM1 | -1.602999767 | 3.129916176 | -10.45874512 | 1.29E-23 | 1.33E-22 | 42.44557889 |
| MTURN | -1.186500604 | 2.742943671 | -10.45737026 | 1.30E-23 | 1.35E-22 | 42.43343758 |
| CTGF | -1.630439837 | 6.158432268 | -10.45512154 | 1.33E-23 | 1.37E-22 | 42.41358155 |
| C11orf97 | -1.22567261 | 0.619179164 | -10.45483879 | 1.33E-23 | 1.38E-22 | 42.41108517 |
| PRR11 | 1.28768971 | 2.058876612 | 10.45332748 | 1.35E-23 | 1.39E-22 | 42.39774232 |
| IGFBP3 | 1.922724784 | 5.523698739 | 10.45276563 | 1.36E-23 | 1.40E-22 | 42.39278231 |
| RP11-196G18.22 | 1.04747073 | 1.702995973 | 10.44991604 | 1.39E-23 | 1.43E-22 | 42.36762886 |
| PREX1 | -1.065666479 | 2.996226334 | -10.44935577 | 1.40E-23 | 1.44E-22 | 42.36268389 |
| B3GALNT1 | -1.138946222 | 2.110172994 | -10.44401217 | 1.47E-23 | 1.51E-22 | 42.31553025 |
| EXOSC5 | 1.047210579 | 3.66720938 | 10.44309095 | 1.48E-23 | 1.52E-22 | 42.30740273 |
| EMP1 | -1.358785913 | 4.137940725 | -10.43926825 | 1.53E-23 | 1.57E-22 | 42.27368227 |
| FAM83A-AS1 | 1.485260334 | 1.38238992 | 10.43099203 | 1.65E-23 | 1.69E-22 | 42.20070589 |
| TYROBP | -1.623423259 | 6.223774026 | -10.43039492 | 1.65E-23 | 1.69E-22 | 42.19544241 |
| RNASEH2A | 1.063545633 | 3.328873487 | 10.41774833 | 1.85E-23 | 1.89E-22 | 42.0840115 |
| IDH2 | 1.062426836 | 5.518715508 | 10.41320686 | 1.93E-23 | 1.97E-22 | 42.04401878 |
| EHD2 | -1.364479155 | 5.213963863 | -10.38824212 | 2.41E-23 | 2.45E-22 | 41.82439162 |
| OIP5 | 1.017296246 | 1.397061033 | 10.36329677 | 3.00E-23 | 3.04E-22 | 41.60529983 |
| RP4-666F24.3 | -1.086348807 | 0.559936023 | -10.35322498 | 3.28E-23 | 3.32E-22 | 41.51694421 |
| APOA1BP | 1.032237355 | 5.799065214 | 10.34496248 | 3.53E-23 | 3.56E-22 | 41.44450523 |
| RAC3 | 1.526411662 | 2.870323785 | 10.34294741 | 3.59E-23 | 3.62E-22 | 41.42684474 |
| SEC14L4 | -1.185639001 | 1.306061844 | -10.34115803 | 3.65E-23 | 3.67E-22 | 41.41116435 |
| GREM1 | 1.721875873 | 1.719808335 | 10.33350253 | 3.91E-23 | 3.92E-22 | 41.34410002 |
| JAM3 | -1.017210739 | 2.27290852 | -10.32599659 | 4.18E-23 | 4.17E-22 | 41.27837943 |
| PCNA | 1.026283778 | 5.706572584 | 10.32598681 | 4.18E-23 | 4.17E-22 | 41.27829381 |
| ARHGAP11A | 1.058566386 | 1.668983383 | 10.32349049 | 4.27E-23 | 4.26E-22 | 41.25644395 |
| GABARAPL1 | -1.032632163 | 4.148269183 | -10.32312145 | 4.28E-23 | 4.27E-22 | 41.25321408 |
| C11orf88 | -1.146685693 | 0.609249722 | -10.31629375 | 4.55E-23 | 4.53E-22 | 41.19347249 |
| ADCY9 | -1.004544387 | 2.386198675 | -10.31626649 | 4.55E-23 | 4.53E-22 | 41.19323398 |
| WISP1 | 1.242453043 | 1.850840463 | 10.314738 | 4.61E-23 | 4.59E-22 | 41.17986365 |
| MICALL2 | 1.133887518 | 2.547385747 | 10.31454195 | 4.62E-23 | 4.60E-22 | 41.17814885 |
| RPL39L | 1.683132666 | 3.33912459 | 10.30541424 | 5.01E-23 | 4.98E-22 | 41.0983349 |
| AQP9 | -1.435797018 | 2.150641017 | -10.30504883 | 5.02E-23 | 4.99E-22 | 41.09514071 |
| IHH | -1.201740873 | 0.585294867 | -10.30364977 | 5.09E-23 | 5.05E-22 | 41.08291175 |
| LST1 | -1.338785455 | 2.978208017 | -10.30194333 | 5.16E-23 | 5.12E-22 | 41.06799769 |
| BLACAT1 | 1.040354008 | 1.218660754 | 10.2974992 | 5.37E-23 | 5.32E-22 | 41.02916449 |
| PAQR4 | 1.056948967 | 2.567282377 | 10.28332163 | 6.09E-23 | 6.01E-22 | 40.90535781 |
| C7 | -2.257942566 | 3.604237433 | -10.27853494 | 6.35E-23 | 6.26E-22 | 40.86358453 |
| CCDC64 | 1.224562962 | 2.044721225 | 10.27577583 | 6.50E-23 | 6.41E-22 | 40.83951206 |
| COL5A1 | 1.706233731 | 4.346180265 | 10.27494183 | 6.55E-23 | 6.45E-22 | 40.83223656 |
| CHI3L2 | -1.703281379 | 2.30494118 | -10.27150161 | 6.75E-23 | 6.64E-22 | 40.80222957 |
| PABPC1L | 1.477217635 | 2.598917884 | 10.27030839 | 6.82E-23 | 6.70E-22 | 40.79182352 |
| LIMK1 | 1.012731497 | 3.703175243 | 10.26758958 | 6.99E-23 | 6.86E-22 | 40.76811589 |
| DCN | -1.554532587 | 4.802232549 | -10.25789626 | 7.61E-23 | 7.45E-22 | 40.68362717 |
| NSMF | 1.025515258 | 3.399096442 | 10.25680358 | 7.69E-23 | 7.52E-22 | 40.67410668 |
| PRELP | -1.63407327 | 3.679396341 | -10.2539998 | 7.88E-23 | 7.70E-22 | 40.64968067 |
| C1QB | -1.81545635 | 6.95772063 | -10.24694728 | 8.38E-23 | 8.18E-22 | 40.58826098 |
| CES1 | -2.550186176 | 4.125530494 | -10.24229721 | 8.73E-23 | 8.52E-22 | 40.54778022 |
| CYP27B1 | 1.019121084 | 1.418159442 | 10.24166631 | 8.78E-23 | 8.56E-22 | 40.54228899 |
| RP11-295G20.2 | 1.53757343 | 2.444915985 | 10.23987237 | 8.92E-23 | 8.69E-22 | 40.52667606 |
| NFAM1 | -1.026159165 | 1.905414036 | -10.19553033 | 1.32E-22 | 1.27E-21 | 40.14137114 |
| RASSF2 | -1.105421912 | 2.851165208 | -10.19475 | 1.33E-22 | 1.28E-21 | 40.13460106 |
| ARRDC4 | -1.061266894 | 2.725922073 | -10.1945978 | 1.33E-22 | 1.28E-21 | 40.13328061 |
| SMPDL3B | 1.508597393 | 4.188854818 | 10.17123919 | 1.63E-22 | 1.56E-21 | 39.93079389 |
| NCKAP1L | -1.136181552 | 2.481143702 | -10.16879402 | 1.66E-22 | 1.60E-21 | 39.90961648 |
| HCK | -1.377224218 | 3.49292616 | -10.15445621 | 1.89E-22 | 1.80E-21 | 39.78551037 |
| RHPN1 | 1.393988123 | 2.892810044 | 10.1430035 | 2.08E-22 | 1.98E-21 | 39.68646594 |
| NQO1 | 2.247241699 | 5.452540954 | 10.13258474 | 2.28E-22 | 2.17E-21 | 39.59643155 |
| DTX2 | 1.019224099 | 3.01718059 | 10.13141099 | 2.31E-22 | 2.19E-21 | 39.5862926 |
| CNN1 | -1.292340225 | 2.340089346 | -10.12889171 | 2.36E-22 | 2.23E-21 | 39.56453371 |
| FAP | 1.183774855 | 1.768409742 | 10.0960221 | 3.14E-22 | 2.96E-21 | 39.28099023 |
| MYLK | -1.097964206 | 2.467556014 | -10.09024846 | 3.30E-22 | 3.12E-21 | 39.23125227 |
| GRAMD2 | -1.301680036 | 2.252294038 | -10.08999059 | 3.31E-22 | 3.12E-21 | 39.22903125 |
| PDPN | -1.442394558 | 2.995433444 | -10.08980786 | 3.31E-22 | 3.12E-21 | 39.22745742 |
| C15orf48 | 2.280660636 | 4.385712705 | 10.07731875 | 3.69E-22 | 3.48E-21 | 39.11994013 |
| LAPTM4B | 1.296032518 | 6.379750305 | 10.0731106 | 3.83E-22 | 3.60E-21 | 39.08373392 |
| ETV5 | -1.05542329 | 3.242382745 | -10.06194148 | 4.22E-22 | 3.95E-21 | 38.98768859 |
| CELF2 | -1.167327992 | 3.074109136 | -10.05725237 | 4.40E-22 | 4.11E-21 | 38.94738857 |
| TPSB2 | -1.926029029 | 3.089802662 | -10.054495 | 4.50E-22 | 4.21E-21 | 38.92369691 |
| SOX12 | 1.046877447 | 2.811512093 | 10.05197931 | 4.60E-22 | 4.30E-21 | 38.9020858 |
| F12 | 1.031944583 | 1.256331289 | 10.05123833 | 4.63E-22 | 4.32E-21 | 38.89572113 |
| EEF1A1P5 | -1.707454504 | 4.39496377 | -10.0464786 | 4.83E-22 | 4.50E-21 | 38.85484509 |
| CPA3 | -1.938880679 | 3.477402842 | -10.03739867 | 5.22E-22 | 4.85E-21 | 38.77690586 |
| CYR61 | -1.593765307 | 6.0218304 | -10.02509193 | 5.81E-22 | 5.39E-21 | 38.67134859 |
| TNNI2 | -1.23809535 | 1.363810499 | -10.0217956 | 5.98E-22 | 5.54E-21 | 38.64309101 |
| PXDC1 | -1.038030398 | 3.904579258 | -10.01914196 | 6.12E-22 | 5.66E-21 | 38.62034757 |
| AC009005.2 | 1.068201167 | 1.662194428 | 10.01828494 | 6.16E-22 | 5.70E-21 | 38.61300325 |
| MID1IP1 | -1.019317194 | 4.304021714 | -10.00532747 | 6.89E-22 | 6.34E-21 | 38.50201769 |
| C9orf24 | -1.804496071 | 1.342846922 | -9.996444247 | 7.44E-22 | 6.82E-21 | 38.42598861 |
| SNORD99 | 1.376558295 | 2.547849269 | 9.987167766 | 8.07E-22 | 7.37E-21 | 38.34664508 |
| SBK1 | 1.527453083 | 2.403180961 | 9.985070316 | 8.21E-22 | 7.50E-21 | 38.32871246 |
| LSR | 1.228401694 | 6.138519741 | 9.979131112 | 8.65E-22 | 7.90E-21 | 38.27794845 |
| CDH3 | 2.018917662 | 3.531474229 | 9.970157384 | 9.34E-22 | 8.52E-21 | 38.20128839 |
| PKDCC | -1.252586951 | 2.01191226 | -9.950309776 | 1.11E-21 | 1.00E-20 | 38.0319106 |
| HNF4G | 1.110442218 | 1.211570267 | 9.942167809 | 1.19E-21 | 1.08E-20 | 37.96249747 |
| ARHGEF19 | 1.205769644 | 3.184980593 | 9.934271108 | 1.27E-21 | 1.15E-20 | 37.89521412 |
| ETS1 | -1.1069066 | 3.989020593 | -9.926180675 | 1.36E-21 | 1.23E-20 | 37.82631973 |
| MMP12 | 2.448090567 | 2.98334086 | 9.92385185 | 1.39E-21 | 1.25E-20 | 37.80649598 |
| TFAP2A | 1.153661969 | 1.227144902 | 9.919526133 | 1.44E-21 | 1.30E-20 | 37.76968283 |
| FAM83H | 1.06847152 | 4.392774916 | 9.914131275 | 1.51E-21 | 1.36E-20 | 37.72378708 |
| ADAM28 | 1.248687289 | 1.939181341 | 9.913145352 | 1.53E-21 | 1.37E-20 | 37.71540145 |
| XDH | 1.499607833 | 1.645363727 | 9.912589601 | 1.53E-21 | 1.37E-20 | 37.71067485 |
| HPGDS | -1.142753582 | 1.530089996 | -9.911904394 | 1.54E-21 | 1.38E-20 | 37.70484751 |
| RACGAP1 | 1.102134889 | 2.88649403 | 9.90636929 | 1.62E-21 | 1.44E-20 | 37.6577848 |
| FAM216B | -1.649483632 | 1.092428612 | -9.90317003 | 1.66E-21 | 1.48E-20 | 37.63059139 |
| MCM7 | 1.079800004 | 4.585769218 | 9.895554163 | 1.78E-21 | 1.58E-20 | 37.56588258 |
| CXCL16 | -1.057241369 | 5.630741182 | -9.892453861 | 1.82E-21 | 1.62E-20 | 37.53955084 |
| SRD5A3 | 1.290986704 | 3.890169813 | 9.884277267 | 1.96E-21 | 1.73E-20 | 37.47013311 |
| IRX1 | -1.277384445 | 0.83688138 | -9.860154551 | 2.40E-21 | 2.12E-20 | 37.26557597 |
| DAB2 | -1.046344731 | 3.295534915 | -9.854008907 | 2.53E-21 | 2.23E-20 | 37.2135192 |
| VSIG2 | -2.293601911 | 3.452222829 | -9.851601159 | 2.59E-21 | 2.27E-20 | 37.19313069 |
| CX3CR1 | -1.036428928 | 1.168968108 | -9.849574985 | 2.63E-21 | 2.31E-20 | 37.17597609 |
| CCND2 | -1.173384884 | 2.72512868 | -9.847192517 | 2.69E-21 | 2.35E-20 | 37.15580816 |
| PLXNB3 | 1.377598683 | 1.613163653 | 9.829785393 | 3.12E-21 | 2.72E-20 | 37.00856097 |
| GALNT14 | 1.600092101 | 1.865388018 | 9.829707093 | 3.12E-21 | 2.72E-20 | 37.00789905 |
| GPR133 | -1.245141778 | 1.636753975 | -9.829081018 | 3.14E-21 | 2.73E-20 | 37.00260659 |
| SLC22A18 | 1.040225821 | 2.71434369 | 9.822556817 | 3.32E-21 | 2.88E-20 | 36.94746944 |
| CXCL13 | 2.362605021 | 3.49059247 | 9.811202022 | 3.65E-21 | 3.16E-20 | 36.851571 |
| VGLL3 | -1.07867009 | 1.504992597 | -9.807900206 | 3.76E-21 | 3.24E-20 | 36.82370009 |
| CXCL2 | -1.943767932 | 4.243613077 | -9.7916431 | 4.32E-21 | 3.70E-20 | 36.68657115 |
| HCAR2 | -1.462935216 | 1.812595002 | -9.788201333 | 4.44E-21 | 3.81E-20 | 36.65756086 |
| ARHGAP40 | 1.462160968 | 1.624243553 | 9.78348987 | 4.63E-21 | 3.96E-20 | 36.61786038 |
| TNFRSF18 | 1.492465015 | 2.26393755 | 9.779728032 | 4.78E-21 | 4.08E-20 | 36.58617169 |
| KLF15 | -1.293081836 | 1.722051883 | -9.776205495 | 4.92E-21 | 4.20E-20 | 36.55650681 |
| HLF | -1.475944336 | 1.7991748 | -9.776205291 | 4.92E-21 | 4.20E-20 | 36.55650508 |
| TMEM45B | 1.431120595 | 3.911795249 | 9.774299277 | 5.00E-21 | 4.27E-20 | 36.54045689 |
| FOXF2 | -1.119666289 | 2.005327364 | -9.769152188 | 5.23E-21 | 4.45E-20 | 36.4971309 |
| AL450992.2 | 1.381004716 | 3.033088355 | 9.766936419 | 5.33E-21 | 4.54E-20 | 36.47848459 |
| CILP2 | 1.342942552 | 1.473306529 | 9.763429412 | 5.49E-21 | 4.67E-20 | 36.4489784 |
| RP11-664D7.4 | -1.589107536 | 1.796667756 | -9.761209004 | 5.59E-21 | 4.75E-20 | 36.43030097 |
| AADAC | -1.675861828 | 1.290555962 | -9.75992872 | 5.65E-21 | 4.80E-20 | 36.41953299 |
| DHCR24 | -1.063169795 | 7.178375195 | -9.75921133 | 5.69E-21 | 4.83E-20 | 36.41349974 |
| ATF3 | -1.566706029 | 3.553390427 | -9.755955825 | 5.85E-21 | 4.95E-20 | 36.38612499 |
| THY1 | 1.358734152 | 3.608611029 | 9.718470624 | 8.04E-21 | 6.75E-20 | 36.07139784 |
| BANCR | -1.225359019 | 0.757187559 | -9.712001264 | 8.49E-21 | 7.12E-20 | 36.01716976 |
| LAGE3 | 1.042002532 | 4.373336163 | 9.711612569 | 8.52E-21 | 7.14E-20 | 36.01391244 |
| CERCAM | 1.159707528 | 3.114954708 | 9.711129156 | 8.55E-21 | 7.16E-20 | 36.0098615 |
| TBC1D2 | -1.133183967 | 3.83258729 | -9.706969774 | 8.86E-21 | 7.41E-20 | 35.97501246 |
| CPXM1 | 1.399681 | 2.364852016 | 9.702859704 | 9.17E-21 | 7.66E-20 | 35.94058722 |
| MIR559 | 1.157123671 | 1.495623171 | 9.70180451 | 9.26E-21 | 7.72E-20 | 35.9317508 |
| SLC22A18AS | 1.086256027 | 1.506163095 | 9.699157824 | 9.47E-21 | 7.89E-20 | 35.90958998 |
| BIK | 1.33654078 | 2.707786588 | 9.681973129 | 1.09E-20 | 9.08E-20 | 35.76580862 |
| CYP24A1 | 2.638938147 | 2.652711119 | 9.680707336 | 1.11E-20 | 9.18E-20 | 35.75522529 |
| EVI2B | -1.221439803 | 3.685844759 | -9.680466469 | 1.11E-20 | 9.19E-20 | 35.7532115 |
| CXCL12 | -1.257647597 | 2.734816107 | -9.670391532 | 1.21E-20 | 9.97E-20 | 35.66901204 |
| ARHGAP30 | -1.049552074 | 3.111493493 | -9.669695896 | 1.21E-20 | 1.00E-19 | 35.66320075 |
| ITGA11 | 1.406660478 | 1.837489802 | 9.651651147 | 1.41E-20 | 1.16E-19 | 35.51256251 |
| CDKN2B | -1.295355619 | 2.731631837 | -9.648158299 | 1.46E-20 | 1.19E-19 | 35.48342777 |
| SDC2 | -1.150386144 | 3.457923757 | -9.644163277 | 1.51E-20 | 1.23E-19 | 35.45011369 |
| KCNJ15 | -1.681679198 | 2.297469371 | -9.641961652 | 1.53E-20 | 1.25E-19 | 35.43175887 |
| CNFN | 1.326784586 | 1.877114464 | 9.638762948 | 1.58E-20 | 1.29E-19 | 35.4050969 |
| MAL | -1.332686801 | 2.037400413 | -9.633215352 | 1.65E-20 | 1.35E-19 | 35.35887165 |
| TNFRSF25 | 1.156345044 | 1.961549458 | 9.631118352 | 1.68E-20 | 1.37E-19 | 35.3414035 |
| PITX1 | 1.639125073 | 1.690724761 | 9.630355088 | 1.69E-20 | 1.38E-19 | 35.33504615 |
| GPRC5A | -1.564921361 | 5.957262457 | -9.628920607 | 1.71E-20 | 1.39E-19 | 35.32309911 |
| IGHG1 | 2.77848841 | 8.739077179 | 9.620131822 | 1.84E-20 | 1.50E-19 | 35.24993031 |
| NR4A1 | -1.831709309 | 3.639988878 | -9.617441735 | 1.89E-20 | 1.53E-19 | 35.22754443 |
| TEF | -1.009832554 | 2.793695972 | -9.595683411 | 2.26E-20 | 1.83E-19 | 35.04664813 |
| ZFPM2-AS1 | 1.154988158 | 1.096462781 | 9.581568376 | 2.55E-20 | 2.05E-19 | 34.9294576 |
| FAM105A | -1.383105259 | 3.066459017 | -9.579175228 | 2.60E-20 | 2.09E-19 | 34.90960093 |
| EGR1 | -1.719885628 | 5.840648661 | -9.560595094 | 3.04E-20 | 2.44E-19 | 34.75555965 |
| RGS17 | 1.01487995 | 1.069895502 | 9.553416488 | 3.23E-20 | 2.58E-19 | 34.69610311 |
| MANEAL | 1.214699433 | 2.529393884 | 9.549587017 | 3.33E-20 | 2.66E-19 | 34.66439906 |
| IGHG2 | 2.555429947 | 8.045102044 | 9.531337546 | 3.88E-20 | 3.09E-19 | 34.51344055 |
| APLN | -1.330098723 | 2.100527157 | -9.528356729 | 3.98E-20 | 3.16E-19 | 34.48880357 |
| SCEL | -1.784341141 | 3.498510258 | -9.527865817 | 4.00E-20 | 3.17E-19 | 34.48474663 |
| TEKT1 | -1.354829695 | 0.842326316 | -9.526662715 | 4.04E-20 | 3.20E-19 | 34.47480476 |
| SIGLEC14 | -1.016433388 | 1.663728682 | -9.521762556 | 4.21E-20 | 3.33E-19 | 34.43432168 |
| SLC17A9 | 1.355434769 | 2.061128111 | 9.52026153 | 4.26E-20 | 3.37E-19 | 34.42192388 |
| GCNT3 | 1.950048895 | 2.032879798 | 9.508777078 | 4.69E-20 | 3.71E-19 | 34.3271151 |
| IGHGP | 2.371421476 | 5.066351175 | 9.490703322 | 5.45E-20 | 4.29E-19 | 34.17807954 |
| OR7E38P | 1.014538919 | 2.377737451 | 9.484549969 | 5.74E-20 | 4.51E-19 | 34.12738687 |
| AK4 | 1.293756989 | 1.801302788 | 9.482842607 | 5.82E-20 | 4.57E-19 | 34.11332554 |
| ITGAL | -1.158531297 | 2.740131872 | -9.446351945 | 7.88E-20 | 6.13E-19 | 33.81324577 |
| FER1L4 | 1.217228617 | 1.259080498 | 9.445786501 | 7.92E-20 | 6.16E-19 | 33.80860259 |
| ETV1 | -1.165265896 | 2.280836243 | -9.445022747 | 7.97E-20 | 6.19E-19 | 33.80233129 |
| CST1 | 2.619272823 | 2.522417419 | 9.442199362 | 8.16E-20 | 6.34E-19 | 33.77915129 |
| TMSB15A | -1.035182423 | 0.830969316 | -9.440200076 | 8.30E-20 | 6.44E-19 | 33.76274024 |
| SMIM22 | 1.539697888 | 4.196744254 | 9.440100448 | 8.30E-20 | 6.44E-19 | 33.76192251 |
| FBLN1 | -1.392109777 | 4.378749479 | -9.436643667 | 8.55E-20 | 6.61E-19 | 33.73355398 |
| DNAJC12 | 2.088398336 | 2.526998568 | 9.42634054 | 9.31E-20 | 7.19E-19 | 33.64904554 |
| NRGN | -1.930530599 | 3.869860967 | -9.42602086 | 9.33E-20 | 7.21E-19 | 33.64642455 |
| PRR36 | 1.292333898 | 2.210159328 | 9.414479459 | 1.03E-19 | 7.92E-19 | 33.55184301 |
| RHOV | 2.029942536 | 2.936384371 | 9.412866609 | 1.04E-19 | 8.02E-19 | 33.53863257 |
| C10orf107 | -1.172262046 | 1.054416336 | -9.407160193 | 1.09E-19 | 8.39E-19 | 33.49190624 |
| PPP1R14D | 2.471507136 | 2.506717277 | 9.404089462 | 1.12E-19 | 8.61E-19 | 33.46677059 |
| IRF7 | 1.090851702 | 4.126248474 | 9.403606137 | 1.12E-19 | 8.64E-19 | 33.46281487 |
| KRT8 | 1.241898727 | 7.457172469 | 9.382746227 | 1.34E-19 | 1.02E-18 | 33.29223241 |
| CTD-3010D24.3 | 1.190850026 | 1.136196885 | 9.379851144 | 1.37E-19 | 1.04E-18 | 33.26857999 |
| ADAM12 | 1.266171557 | 1.543891017 | 9.376946687 | 1.40E-19 | 1.07E-18 | 33.24485643 |
| FCER1G | -1.321400237 | 5.659535702 | -9.374289524 | 1.43E-19 | 1.09E-18 | 33.22315755 |
| GBP4 | -1.348850989 | 3.289836083 | -9.364336984 | 1.55E-19 | 1.18E-18 | 33.14192383 |
| TUBA1A | -1.173426398 | 5.314219396 | -9.352497003 | 1.71E-19 | 1.30E-18 | 33.04536811 |
| GSDMB | 1.124860536 | 2.220114435 | 9.349132892 | 1.76E-19 | 1.34E-18 | 33.01795016 |
| EGR3 | -1.059296652 | 1.419086393 | -9.336409786 | 1.96E-19 | 1.48E-18 | 32.91432148 |
| DNTTIP1 | 1.196717262 | 4.773520834 | 9.335763118 | 1.97E-19 | 1.49E-18 | 32.90905722 |
| SRGAP3-AS2 | -1.4792414 | 0.913584216 | -9.33522152 | 1.98E-19 | 1.49E-18 | 32.90464851 |
| TSKU | 1.320533155 | 3.772165267 | 9.333797215 | 2.00E-19 | 1.51E-18 | 32.89305529 |
| ITM2C | 1.106157854 | 5.561397149 | 9.328220458 | 2.09E-19 | 1.58E-18 | 32.84767561 |
| ID3 | -1.138404046 | 4.532908581 | -9.327952263 | 2.10E-19 | 1.58E-18 | 32.84549373 |
| GOLGA7B | 1.324507147 | 1.870160257 | 9.326017235 | 2.13E-19 | 1.60E-18 | 32.82975291 |
| DBN1 | 1.055658894 | 4.220689021 | 9.324139445 | 2.17E-19 | 1.63E-18 | 32.81448002 |
| CPNE7 | 1.218220269 | 1.44295417 | 9.318758342 | 2.26E-19 | 1.70E-18 | 32.77072584 |
| DSP | 1.805743064 | 4.587388097 | 9.293605983 | 2.78E-19 | 2.08E-18 | 32.56645965 |
| MB | 1.50483012 | 1.82464038 | 9.292635285 | 2.80E-19 | 2.09E-18 | 32.55858472 |
| IER5L | 1.145381086 | 2.385478359 | 9.28119418 | 3.08E-19 | 2.29E-18 | 32.46581327 |
| DEPTOR | 1.217919661 | 3.239706361 | 9.272109091 | 3.32E-19 | 2.46E-18 | 32.39220662 |
| SLCO4C1 | -1.345942349 | 1.837381022 | -9.268809473 | 3.41E-19 | 2.53E-18 | 32.36548672 |
| SNTN | -1.16600334 | 0.827071167 | -9.259221655 | 3.69E-19 | 2.72E-18 | 32.28788608 |
| RP3-340N1.2 | 1.670035843 | 1.588840358 | 9.257505209 | 3.74E-19 | 2.76E-18 | 32.27400007 |
| DAPK1 | -1.100547766 | 3.408609576 | -9.256921267 | 3.76E-19 | 2.77E-18 | 32.26927644 |
| SRPX2 | 1.558251585 | 3.126136945 | 9.242138255 | 4.24E-19 | 3.11E-18 | 32.14976756 |
| METTL7B | 1.660343824 | 2.884672531 | 9.232604327 | 4.59E-19 | 3.35E-18 | 32.0727692 |
| C2CD4A | 1.566231126 | 1.720926123 | 9.231790919 | 4.62E-19 | 3.37E-18 | 32.06620267 |
| UBE2S | 1.161223476 | 2.624863585 | 9.23136776 | 4.63E-19 | 3.38E-18 | 32.06278674 |
| COL4A3 | -1.282212816 | 1.700894124 | -9.215439329 | 5.27E-19 | 3.83E-18 | 31.93429053 |
| CHPF | 1.121192744 | 5.284567555 | 9.213192794 | 5.37E-19 | 3.90E-18 | 31.9161809 |
| TUBA4B | -1.188146626 | 0.79628154 | -9.2078044 | 5.61E-19 | 4.07E-18 | 31.8727578 |
| C20orf85 | -2.462352656 | 2.090345506 | -9.20041088 | 5.96E-19 | 4.31E-18 | 31.81320716 |
| C9orf171 | -1.007473212 | 0.619523326 | -9.1893665 | 6.52E-19 | 4.70E-18 | 31.72431777 |
| MORN5 | -1.380396302 | 0.953218754 | -9.179772634 | 7.05E-19 | 5.08E-18 | 31.64716776 |
| SELENBP1 | -1.707343007 | 5.446014024 | -9.166774727 | 7.84E-19 | 5.63E-18 | 31.54274045 |
| RP11-350J20.12 | 1.606858448 | 2.57768079 | 9.166150289 | 7.88E-19 | 5.65E-18 | 31.53772641 |
| PTGIS | -1.253385829 | 2.365747287 | -9.165813877 | 7.90E-19 | 5.67E-18 | 31.53502524 |
| SIX1 | 1.438186901 | 2.119162313 | 9.155340127 | 8.60E-19 | 6.16E-18 | 31.45096504 |
| EPHX3 | 1.445983738 | 2.801786368 | 9.151882065 | 8.85E-19 | 6.32E-18 | 31.42322722 |
| EPHB2 | 1.124349791 | 1.716762677 | 9.128798594 | 1.07E-18 | 7.59E-18 | 31.2382722 |
| GJA1 | -1.46982116 | 4.940048201 | -9.125363448 | 1.10E-18 | 7.80E-18 | 31.21077838 |
| AC013264.2 | -1.367662555 | 1.164107942 | -9.124676791 | 1.10E-18 | 7.84E-18 | 31.20528353 |
| GPR56 | 1.209249616 | 4.161417168 | 9.124259619 | 1.11E-18 | 7.86E-18 | 31.20194534 |
| SGCE | -1.156947727 | 3.379013484 | -9.123863036 | 1.11E-18 | 7.88E-18 | 31.198772 |
| FCGR3A | -1.453743521 | 5.001703444 | -9.120419701 | 1.14E-18 | 8.10E-18 | 31.17122389 |
| EGLN3 | 1.653299131 | 2.512063076 | 9.118938446 | 1.16E-18 | 8.19E-18 | 31.15937564 |
| MMP28 | -1.94570021 | 2.686291713 | -9.098520904 | 1.36E-18 | 9.60E-18 | 30.99620808 |
| SGK1 | -1.082984578 | 3.490464607 | -9.083234066 | 1.54E-18 | 1.08E-17 | 30.87422361 |
| C6 | -1.025695197 | 0.693629301 | -9.082735842 | 1.55E-18 | 1.09E-17 | 30.87025054 |
| SIGLEC1 | -1.149325684 | 2.226375018 | -9.067083044 | 1.76E-18 | 1.23E-17 | 30.74551174 |
| DNAJC22 | 1.123716058 | 1.246269364 | 9.066508598 | 1.76E-18 | 1.23E-17 | 30.74093702 |
| EFCAB1 | -1.01761016 | 0.651938669 | -9.065165751 | 1.78E-18 | 1.25E-17 | 30.73024383 |
| PVRL1 | 1.103811571 | 2.641760972 | 9.063987778 | 1.80E-18 | 1.26E-17 | 30.72086452 |
| RNU1-106P | 1.023833343 | 1.177326308 | 9.040849294 | 2.17E-18 | 1.51E-17 | 30.53681757 |
| LAPTM5 | -1.207530021 | 6.458556708 | -9.024528026 | 2.47E-18 | 1.71E-17 | 30.4072103 |
| COL11A1 | 2.024127239 | 1.955475203 | 9.014518094 | 2.68E-18 | 1.85E-17 | 30.32780931 |
| MMP9 | 1.909293366 | 4.284575412 | 9.008553838 | 2.81E-18 | 1.94E-17 | 30.28053132 |
| MAP1LC3C | -1.135391421 | 1.355425286 | -9.008414055 | 2.82E-18 | 1.94E-17 | 30.27942356 |
| NEIL3 | 1.014748612 | 1.029608211 | 9.007908087 | 2.83E-18 | 1.95E-17 | 30.27541395 |
| CSTA | -1.270573281 | 2.562565065 | -8.999188376 | 3.03E-18 | 2.08E-17 | 30.20634033 |
| MS4A4A | -1.192353993 | 3.208742971 | -8.996838001 | 3.09E-18 | 2.12E-17 | 30.18773042 |
| STOML3 | -1.052059674 | 0.647524843 | -8.992123638 | 3.21E-18 | 2.20E-17 | 30.15041395 |
| CFAP52 | -1.033479411 | 0.731766996 | -8.98483756 | 3.40E-18 | 2.33E-17 | 30.09277036 |
| THEM5 | 1.226094219 | 2.54220926 | 8.969433866 | 3.85E-18 | 2.63E-17 | 29.97102155 |
| ENC1 | 1.048926724 | 3.881547483 | 8.968666937 | 3.87E-18 | 2.64E-17 | 29.96496401 |
| DKK3 | -1.16741596 | 3.893499728 | -8.965186569 | 3.98E-18 | 2.71E-17 | 29.93747945 |
| NMU | 1.664954102 | 1.771708393 | 8.95844793 | 4.20E-18 | 2.85E-17 | 29.88428733 |
| AC093850.2 | 1.2908338 | 1.449065962 | 8.952265106 | 4.41E-18 | 2.99E-17 | 29.8355094 |
| BTG2 | -1.343488282 | 5.624328657 | -8.94420576 | 4.71E-18 | 3.18E-17 | 29.77196564 |
| S100A4 | -1.554091371 | 7.021101299 | -8.925244835 | 5.48E-18 | 3.68E-17 | 29.62264081 |
| PLAU | 1.848500617 | 5.034950021 | 8.922865804 | 5.58E-18 | 3.75E-17 | 29.60392207 |
| CTA-384D8.34 | 1.137689338 | 1.443931695 | 8.915490551 | 5.92E-18 | 3.97E-17 | 29.54591619 |
| ITPKA | 1.812146495 | 1.868249044 | 8.904612453 | 6.45E-18 | 4.31E-17 | 29.46042755 |
| MIR25 | 1.198189529 | 2.073952344 | 8.902162463 | 6.58E-18 | 4.39E-17 | 29.44118462 |
| SDF2L1 | 1.001113037 | 4.690746242 | 8.892584341 | 7.10E-18 | 4.73E-17 | 29.36599411 |
| GGTLC1 | -2.187375937 | 2.736358632 | -8.890159684 | 7.24E-18 | 4.81E-17 | 29.3469698 |
| DPT | -1.54976415 | 3.48996151 | -8.88357548 | 7.63E-18 | 5.06E-17 | 29.29532896 |
| ACY3 | 1.193332701 | 1.531790564 | 8.871802453 | 8.37E-18 | 5.55E-17 | 29.20306445 |
| MRPL12 | 1.065324056 | 4.305432594 | 8.848404339 | 1.01E-17 | 6.64E-17 | 29.0199732 |
| SPRY4 | -1.023044446 | 3.400148837 | -8.844350262 | 1.04E-17 | 6.84E-17 | 28.98828756 |
| MIR23A | -1.081552474 | 0.984145672 | -8.841127833 | 1.07E-17 | 7.01E-17 | 28.96310983 |
| DSG2 | 1.184643265 | 4.725141634 | 8.839097227 | 1.09E-17 | 7.12E-17 | 28.94724776 |
| FMO3 | -1.02487165 | 1.959012331 | -8.835281477 | 1.12E-17 | 7.33E-17 | 28.91744859 |
| EFNB2 | -1.135128471 | 3.535958867 | -8.835086208 | 1.12E-17 | 7.33E-17 | 28.9159239 |
| NGEF | 1.113955729 | 1.331802468 | 8.831259367 | 1.15E-17 | 7.55E-17 | 28.88604856 |
| PRF1 | -1.231424065 | 2.686618245 | -8.827304395 | 1.19E-17 | 7.77E-17 | 28.85518337 |
| IGKJ5 | 2.0829578 | 3.032353769 | 8.825224449 | 1.21E-17 | 7.90E-17 | 28.83895542 |
| UGDH | 1.355646418 | 4.75713453 | 8.82364045 | 1.23E-17 | 7.99E-17 | 28.82659886 |
| ABCA12 | 1.004627624 | 0.951678457 | 8.820743945 | 1.25E-17 | 8.16E-17 | 28.80400805 |
| KRTCAP3 | 1.256943835 | 4.778311677 | 8.818767931 | 1.27E-17 | 8.28E-17 | 28.78859972 |
| WFDC3 | 1.497622143 | 1.709225506 | 8.814661684 | 1.32E-17 | 8.54E-17 | 28.756589 |
| AXL | -1.034199599 | 3.528587531 | -8.807918791 | 1.39E-17 | 8.99E-17 | 28.70404888 |
| AOX1 | -1.049289163 | 1.308816483 | -8.805464443 | 1.42E-17 | 9.16E-17 | 28.68493245 |
| DRAM1 | -1.293302462 | 4.920147308 | -8.804661678 | 1.42E-17 | 9.20E-17 | 28.67868076 |
| C10orf10 | -1.258203509 | 5.154449336 | -8.797609309 | 1.51E-17 | 9.69E-17 | 28.62377793 |
| CCNO | 1.351276101 | 2.165444443 | 8.796812942 | 1.52E-17 | 9.75E-17 | 28.61758033 |
| CD44 | -1.100104845 | 5.044433124 | -8.796175814 | 1.52E-17 | 9.79E-17 | 28.61262228 |
| APOC1 | -1.611720855 | 5.965353534 | -8.789160164 | 1.61E-17 | 1.03E-16 | 28.55804574 |
| EMP3 | -1.064634232 | 4.491135237 | -8.774468465 | 1.81E-17 | 1.15E-16 | 28.4438639 |
| CARD11 | 1.254693815 | 2.588112393 | 8.759506891 | 2.03E-17 | 1.30E-16 | 28.32773593 |
| LYPD1 | 1.15037457 | 1.220988186 | 8.757839311 | 2.06E-17 | 1.31E-16 | 28.31480206 |
| SPINK1 | 3.399231688 | 3.924355108 | 8.75755134 | 2.06E-17 | 1.32E-16 | 28.31256873 |
| STK32A | 1.060417354 | 1.697296505 | 8.749647913 | 2.20E-17 | 1.40E-16 | 28.25129657 |
| C1orf194 | -1.427491215 | 1.227327567 | -8.737638048 | 2.41E-17 | 1.53E-16 | 28.15827056 |
| RP11-304L19.1 | 1.26411739 | 1.410841049 | 8.736953749 | 2.43E-17 | 1.54E-16 | 28.15297309 |
| FA2H | 1.422422927 | 2.532756379 | 8.730380931 | 2.55E-17 | 1.62E-16 | 28.10210621 |
| PXMP4 | -1.067636805 | 2.886621115 | -8.723569929 | 2.69E-17 | 1.71E-16 | 28.04942723 |
| ALDH1A1 | -1.824075848 | 4.705194701 | -8.717490088 | 2.83E-17 | 1.79E-16 | 28.00243019 |
| RP11-56A10.1 | -1.017825836 | 0.821248275 | -8.715860874 | 2.86E-17 | 1.81E-16 | 27.98984071 |
| NKD2 | -1.040233965 | 2.131804675 | -8.714745191 | 2.89E-17 | 1.82E-16 | 27.9812205 |
| ATP13A4 | -1.673236729 | 2.454988583 | -8.706552418 | 3.08E-17 | 1.94E-16 | 27.91794604 |
| TOX3 | 1.481947431 | 2.137170533 | 8.700806499 | 3.22E-17 | 2.03E-16 | 27.8735966 |
| RAMP1 | 1.632522307 | 4.12697029 | 8.695163504 | 3.37E-17 | 2.12E-16 | 27.83006362 |
| POSTN | 1.576424782 | 5.031490746 | 8.691503596 | 3.46E-17 | 2.17E-16 | 27.8018409 |
| RP11-259K15.2 | -1.373408764 | 1.60973344 | -8.683064263 | 3.70E-17 | 2.32E-16 | 27.73679757 |
| CYB5A | -1.172289347 | 4.06363515 | -8.682416563 | 3.72E-17 | 2.33E-16 | 27.73180766 |
| BARX2 | 1.301255592 | 1.433049917 | 8.677897732 | 3.85E-17 | 2.41E-16 | 27.69700242 |
| IGHG3 | 2.357259723 | 7.285428138 | 8.669627581 | 4.11E-17 | 2.56E-16 | 27.63333988 |
| RP11-519G16.5 | 1.559378245 | 1.764625768 | 8.662984634 | 4.33E-17 | 2.70E-16 | 27.58223744 |
| ALPL | -2.111386913 | 4.43777142 | -8.657794846 | 4.50E-17 | 2.80E-16 | 27.54233491 |
| IGKV3-15 | 2.24641022 | 5.993984246 | 8.657010402 | 4.53E-17 | 2.82E-16 | 27.5363052 |
| IGKC | 2.318421547 | 9.520399593 | 8.654874917 | 4.61E-17 | 2.87E-16 | 27.51989272 |
| RTN4RL2 | 1.465284844 | 2.148492044 | 8.649607592 | 4.80E-17 | 2.98E-16 | 27.4794236 |
| IL4I1 | 1.291854972 | 2.523976341 | 8.636098293 | 5.33E-17 | 3.30E-16 | 27.37571845 |
| CFB | 1.260277681 | 2.498624661 | 8.635497092 | 5.36E-17 | 3.31E-16 | 27.37110621 |
| CD37 | -1.080679168 | 3.140877149 | -8.62184298 | 5.96E-17 | 3.67E-16 | 27.26642306 |
| CMB9-22P13.1 | 1.074352749 | 1.664458447 | 8.620392696 | 6.02E-17 | 3.71E-16 | 27.25531161 |
| LIPA | -1.028860325 | 4.942813608 | -8.615010076 | 6.28E-17 | 3.86E-16 | 27.214085 |
| LGSN | 1.496943809 | 1.5431829 | 8.613813739 | 6.34E-17 | 3.89E-16 | 27.20492473 |
| CYBB | -1.444152673 | 4.517348214 | -8.610656872 | 6.50E-17 | 3.99E-16 | 27.18075756 |
| MIR34C | -1.068382266 | 0.596920764 | -8.607259913 | 6.67E-17 | 4.09E-16 | 27.15476008 |
| PANX2 | 1.312794018 | 2.125522396 | 8.60400457 | 6.84E-17 | 4.19E-16 | 27.12985391 |
| ALOX15 | -1.446700309 | 1.098610632 | -8.59856762 | 7.14E-17 | 4.36E-16 | 27.08827291 |
| ZMYND10 | -1.315132068 | 1.358054695 | -8.592840255 | 7.46E-17 | 4.56E-16 | 27.044493 |
| CEACAM5 | 3.305713794 | 5.320482391 | 8.588479502 | 7.72E-17 | 4.71E-16 | 27.01117469 |
| BHLHA15 | 1.263787764 | 1.722027911 | 8.584180006 | 7.98E-17 | 4.86E-16 | 26.97833732 |
| SLC29A4 | 1.401583836 | 1.883385647 | 8.579555207 | 8.27E-17 | 5.03E-16 | 26.94302975 |
| CITED2 | -1.131602289 | 5.444934258 | -8.571053669 | 8.83E-17 | 5.36E-16 | 26.87816431 |
| CAPSL | -1.426462169 | 1.093322005 | -8.569592283 | 8.93E-17 | 5.42E-16 | 26.86701921 |
| TMEM190 | -1.616382864 | 1.27996118 | -8.566480747 | 9.15E-17 | 5.54E-16 | 26.84329435 |
| COL4A4 | -1.060586423 | 1.950988178 | -8.563244841 | 9.38E-17 | 5.68E-16 | 26.81862834 |
| RP11-304L19.3 | 1.290653839 | 1.43092762 | 8.555611058 | 9.95E-17 | 6.01E-16 | 26.76046787 |
| TBX15 | 1.239613268 | 1.375552036 | 8.555475966 | 9.96E-17 | 6.02E-16 | 26.75943899 |
| KRT18 | 1.064533237 | 7.585640308 | 8.547742777 | 1.06E-16 | 6.38E-16 | 26.70056317 |
| CLDN4 | 1.088028749 | 6.290486193 | 8.545519426 | 1.08E-16 | 6.48E-16 | 26.68364363 |
| WDR38 | -1.430481128 | 1.13921205 | -8.539874125 | 1.12E-16 | 6.77E-16 | 26.64069872 |
| PER1 | -1.196062966 | 3.837080876 | -8.5302983 | 1.21E-16 | 7.28E-16 | 26.56790425 |
| PLEKHA6 | 1.138153536 | 2.726582198 | 8.522530781 | 1.28E-16 | 7.70E-16 | 26.50890325 |
| RP3-407E4.4 | 1.636125271 | 1.815822495 | 8.517686908 | 1.33E-16 | 7.98E-16 | 26.47213114 |
| NTM | -1.091216719 | 1.61274095 | -8.511866186 | 1.39E-16 | 8.33E-16 | 26.42796496 |
| EFNB1 | -1.044782319 | 3.251730186 | -8.508501637 | 1.43E-16 | 8.54E-16 | 26.40244638 |
| RN7SL8P | -1.163063599 | 0.802728775 | -8.506002208 | 1.46E-16 | 8.70E-16 | 26.38349445 |
| FAM92B | -1.289026088 | 1.115543762 | -8.499064644 | 1.54E-16 | 9.16E-16 | 26.3309132 |
| SCNN1G | -1.333115581 | 2.059188022 | -8.497419705 | 1.56E-16 | 9.27E-16 | 26.31845079 |
| LCP1 | -1.189291412 | 5.285506807 | -8.491871951 | 1.63E-16 | 9.65E-16 | 26.27643376 |
| TFRC | -1.114476242 | 4.222056422 | -8.490925046 | 1.64E-16 | 9.72E-16 | 26.26926433 |
| FOS | -1.577618961 | 6.257209764 | -8.483893107 | 1.73E-16 | 1.02E-15 | 26.21604207 |
| C5orf38 | -1.468174973 | 2.017517775 | -8.470632401 | 1.91E-16 | 1.13E-15 | 26.11577055 |
| BCL2A1 | -1.170748489 | 3.197747933 | -8.467906656 | 1.95E-16 | 1.15E-15 | 26.09517493 |
| C1orf189 | -1.188255299 | 0.977979155 | -8.463111101 | 2.03E-16 | 1.19E-15 | 26.05895254 |
| SLPI | -2.28245568 | 8.228862069 | -8.451281862 | 2.22E-16 | 1.30E-15 | 25.96967131 |
| ANO9 | 1.090568791 | 2.482905862 | 8.448102144 | 2.27E-16 | 1.33E-15 | 25.94568909 |
| PDGFRL | 1.067367594 | 2.629572784 | 8.441596314 | 2.39E-16 | 1.40E-15 | 25.89664263 |
| PLBD1 | -1.23855152 | 3.860273702 | -8.426438675 | 2.68E-16 | 1.57E-15 | 25.78248668 |
| EEF1A2 | 2.696737907 | 2.870199801 | 8.424660752 | 2.72E-16 | 1.59E-15 | 25.76910727 |
| CAMK2N1 | -1.392963874 | 3.59892661 | -8.424164225 | 2.73E-16 | 1.59E-15 | 25.76537115 |
| ARNTL2 | 1.270300817 | 2.059415811 | 8.423471663 | 2.74E-16 | 1.60E-15 | 25.76016026 |
| KIFC2 | 1.071363719 | 2.113502729 | 8.413524686 | 2.96E-16 | 1.72E-15 | 25.68535553 |
| SOD3 | -1.276366349 | 4.035859277 | -8.403550716 | 3.19E-16 | 1.85E-15 | 25.61041767 |
| RP11-799B12.1 | 1.011534284 | 2.236427656 | 8.391126318 | 3.51E-16 | 2.04E-15 | 25.51716683 |
| NDN | -1.017687255 | 3.15989851 | -8.388961353 | 3.57E-16 | 2.07E-15 | 25.50092889 |
| ROPN1L | -1.1320388 | 0.996426145 | -8.384002864 | 3.70E-16 | 2.14E-15 | 25.46375107 |
| MUC20 | 1.496853942 | 3.059572033 | 8.381780271 | 3.77E-16 | 2.18E-15 | 25.4470921 |
| ERICH2 | -1.097360034 | 2.254841197 | -8.36258818 | 4.36E-16 | 2.51E-15 | 25.30338689 |
| HCST | -1.054096896 | 3.243751491 | -8.352852809 | 4.69E-16 | 2.70E-15 | 25.23059046 |
| SNX30 | -1.020985428 | 2.789739409 | -8.347783177 | 4.88E-16 | 2.80E-15 | 25.19270871 |
| SLC2A3 | -1.186164825 | 3.29716019 | -8.344001415 | 5.02E-16 | 2.88E-15 | 25.16446212 |
| SRGN | -1.219264238 | 6.760438676 | -8.338486113 | 5.23E-16 | 2.99E-15 | 25.12328558 |
| COMP | 1.900362855 | 2.837171154 | 8.329094812 | 5.62E-16 | 3.20E-15 | 25.05322086 |
| IGKV4-1 | 2.359462026 | 7.61634856 | 8.310225342 | 6.48E-16 | 3.68E-15 | 24.91263216 |
| RSPH4A | -1.160756874 | 1.075668131 | -8.303515348 | 6.82E-16 | 3.87E-15 | 24.86269959 |
| BEX5 | -1.091126626 | 1.84574977 | -8.294663961 | 7.29E-16 | 4.13E-15 | 24.79688067 |
| CSF3R | -1.352992208 | 2.661297791 | -8.294511745 | 7.29E-16 | 4.13E-15 | 24.79574927 |
| ENTPD8 | 1.047725978 | 1.040781771 | 8.293063256 | 7.37E-16 | 4.17E-15 | 24.78498376 |
| BEX4 | -1.043703162 | 4.309094526 | -8.289729032 | 7.56E-16 | 4.27E-15 | 24.76020868 |
| IGKV3-11 | 2.138927137 | 7.153427352 | 8.284820985 | 7.85E-16 | 4.43E-15 | 24.72375361 |
| B4GALNT4 | 1.374902472 | 1.392536042 | 8.281490374 | 8.05E-16 | 4.54E-15 | 24.69902488 |
| CFTR | -1.193661222 | 1.450504508 | -8.26845584 | 8.88E-16 | 4.98E-15 | 24.60232342 |
| SGPP2 | 1.302773125 | 4.257158456 | 8.265451644 | 9.08E-16 | 5.09E-15 | 24.58005283 |
| ACTG2 | -1.179698436 | 2.21589589 | -8.256606874 | 9.70E-16 | 5.43E-15 | 24.51452243 |
| IGHV4-34 | 2.21529691 | 5.623209248 | 8.252098928 | 1.00E-15 | 5.61E-15 | 24.48114475 |
| PODNL1 | 1.215665184 | 1.685023334 | 8.225097307 | 1.23E-15 | 6.81E-15 | 24.28152297 |
| KCNQ3 | 1.015680039 | 1.640151127 | 8.214236697 | 1.33E-15 | 7.37E-15 | 24.20137773 |
| SCG5 | 1.407884556 | 1.94646578 | 8.202417928 | 1.46E-15 | 8.02E-15 | 24.1142576 |
| HES6 | 1.487640206 | 2.682647798 | 8.200301494 | 1.48E-15 | 8.14E-15 | 24.0986672 |
| CBX2 | 1.107553154 | 1.624035938 | 8.185061724 | 1.66E-15 | 9.07E-15 | 23.98650033 |
| IGHV3-23 | 2.095312155 | 7.079477406 | 8.181459258 | 1.70E-15 | 9.32E-15 | 23.96000998 |
| E2F1 | 1.059134405 | 2.973113563 | 8.18132808 | 1.70E-15 | 9.32E-15 | 23.95904556 |
| FKBP10 | 1.372592438 | 4.465472346 | 8.164631185 | 1.93E-15 | 1.05E-14 | 23.83638976 |
| AKAP12 | -1.344291691 | 2.349205308 | -8.15215241 | 2.12E-15 | 1.15E-14 | 23.744851 |
| SLC16A3 | 1.104546058 | 4.098075985 | 8.151071608 | 2.14E-15 | 1.16E-14 | 23.73692798 |
| TMEM63C | 1.090794556 | 1.137168319 | 8.144814302 | 2.24E-15 | 1.21E-14 | 23.69107411 |
| NFE2L3 | 1.040957002 | 3.263978538 | 8.14149364 | 2.29E-15 | 1.24E-14 | 23.66675156 |
| CABYR | 1.362944389 | 1.424150734 | 8.134083278 | 2.42E-15 | 1.31E-14 | 23.61250214 |
| ESPN | 1.208413583 | 1.777461081 | 8.121254481 | 2.67E-15 | 1.44E-14 | 23.5186791 |
| EIF4EBP1 | 1.218911414 | 4.83517245 | 8.120506845 | 2.68E-15 | 1.44E-14 | 23.51321493 |
| CPD | 1.106235059 | 4.455437076 | 8.118267873 | 2.73E-15 | 1.47E-14 | 23.49685363 |
| ASPHD1 | 1.312046281 | 1.91533002 | 8.11487703 | 2.79E-15 | 1.50E-14 | 23.47208189 |
| LINC00665 | 1.081333027 | 1.675535056 | 8.111812282 | 2.86E-15 | 1.54E-14 | 23.44969955 |
| HIST1H3H | 1.173269784 | 1.700656483 | 8.110610975 | 2.88E-15 | 1.55E-14 | 23.44092807 |
| SLC6A8 | 1.18816268 | 2.649615696 | 8.10860889 | 2.93E-15 | 1.57E-14 | 23.42631192 |
| XKRX | 1.32424423 | 1.889960785 | 8.103545458 | 3.04E-15 | 1.63E-14 | 23.3893594 |
| RCN3 | 1.29422231 | 3.875529272 | 8.099192192 | 3.14E-15 | 1.68E-14 | 23.35760441 |
| IGHV1-24 | 2.408867474 | 5.032437466 | 8.095320485 | 3.23E-15 | 1.73E-14 | 23.32937364 |
| GPR116 | -1.571729727 | 4.797550158 | -8.092642237 | 3.30E-15 | 1.76E-14 | 23.30985137 |
| PLAC8 | -1.336589068 | 2.185650416 | -8.073356904 | 3.80E-15 | 2.02E-14 | 23.16942979 |
| PLOD2 | 1.354808723 | 3.483703411 | 8.060989636 | 4.16E-15 | 2.21E-14 | 23.07952192 |
| STRA6 | 1.102750233 | 1.1154721 | 8.052481019 | 4.43E-15 | 2.35E-14 | 23.01773002 |
| AC007405.6 | -1.013898399 | 2.409502263 | -8.043660968 | 4.73E-15 | 2.50E-14 | 22.95373173 |
| IGLC2 | 2.046242372 | 8.619546226 | 8.033158079 | 5.11E-15 | 2.69E-14 | 22.87759631 |
| MFAP2 | 1.152234529 | 3.026041202 | 8.025293616 | 5.41E-15 | 2.84E-14 | 22.8206392 |
| HSD17B13 | -1.012687483 | 0.741041797 | -8.025065297 | 5.42E-15 | 2.85E-14 | 22.8189863 |
| CECR1 | -1.033034845 | 3.978581938 | -8.017828352 | 5.72E-15 | 3.00E-14 | 22.76661474 |
| REEP6 | 1.34830653 | 2.655999711 | 8.010485537 | 6.04E-15 | 3.16E-14 | 22.7135159 |
| SLC39A4 | 1.038849209 | 3.171639548 | 8.006246471 | 6.23E-15 | 3.25E-14 | 22.68287933 |
| C4orf48 | 1.094252843 | 2.624155117 | 8.004427463 | 6.31E-15 | 3.29E-14 | 22.66973701 |
| IGLL5 | 1.889187349 | 4.740571726 | 8.002357016 | 6.41E-15 | 3.34E-14 | 22.65478098 |
| CA9 | 2.042026686 | 2.086114379 | 7.989392671 | 7.05E-15 | 3.66E-14 | 22.56120285 |
| IGHV1-46 | 2.101469392 | 4.94018113 | 7.985204564 | 7.27E-15 | 3.77E-14 | 22.53099874 |
| GLB1L2 | 1.020497466 | 2.390026982 | 7.976878923 | 7.72E-15 | 4.01E-14 | 22.47099319 |
| TMEM156 | 1.044631525 | 1.464707002 | 7.975603223 | 7.80E-15 | 4.04E-14 | 22.46180327 |
| LYZ | -1.74340467 | 7.558532447 | -7.971932008 | 8.01E-15 | 4.15E-14 | 22.4353631 |
| TRIM2 | 1.099346564 | 3.459197218 | 7.951642879 | 9.29E-15 | 4.79E-14 | 22.28941755 |
| CNTD2 | 1.08009591 | 1.112176009 | 7.948477168 | 9.50E-15 | 4.89E-14 | 22.26667276 |
| PRSS22 | 1.202256225 | 3.28999855 | 7.946315924 | 9.65E-15 | 4.97E-14 | 22.251149 |
| RP11-59D5__B.2 | 1.01061928 | 1.065645681 | 7.943096456 | 9.88E-15 | 5.08E-14 | 22.22803057 |
| FAM166B | -1.106754459 | 1.145295945 | -7.92313949 | 1.14E-14 | 5.83E-14 | 22.08489209 |
| OASL | -1.03041063 | 2.130240699 | -7.916268698 | 1.20E-14 | 6.12E-14 | 22.0356797 |
| DAAM2 | -1.00067086 | 2.041287105 | -7.916010189 | 1.20E-14 | 6.13E-14 | 22.0338288 |
| FEZF1-AS1 | 1.181562221 | 1.109069067 | 7.897482549 | 1.38E-14 | 6.98E-14 | 21.90129944 |
| CENPW | 1.051440228 | 2.928474288 | 7.89519129 | 1.40E-14 | 7.09E-14 | 21.88492739 |
| FAXDC2 | -1.233392741 | 2.581651317 | -7.866884911 | 1.72E-14 | 8.66E-14 | 21.68298365 |
| MFI2 | 1.104570437 | 1.505575627 | 7.86542756 | 1.74E-14 | 8.75E-14 | 21.67260252 |
| IGKV3-20 | 2.113928405 | 8.001696164 | 7.864984308 | 1.74E-14 | 8.77E-14 | 21.66944542 |
| IGLV3-21 | 2.251594699 | 6.684055106 | 7.844879185 | 2.02E-14 | 1.01E-13 | 21.52639675 |
| CCDC42B | -1.045232427 | 0.9590816 | -7.841083467 | 2.07E-14 | 1.04E-13 | 21.49942344 |
| CD53 | -1.056071633 | 4.990744366 | -7.830403013 | 2.24E-14 | 1.12E-13 | 21.42358244 |
| HLA-DOA | -1.270240941 | 3.92794089 | -7.813253041 | 2.53E-14 | 1.25E-13 | 21.3019778 |
| MMP13 | 1.907899843 | 1.903050419 | 7.805550797 | 2.68E-14 | 1.32E-13 | 21.24743439 |
| CD163 | -1.290254177 | 3.896279523 | -7.794183275 | 2.90E-14 | 1.43E-13 | 21.16701534 |
| C1QC | -1.330583912 | 6.949825284 | -7.793827968 | 2.91E-14 | 1.43E-13 | 21.16450327 |
| LRRC46 | -1.106140326 | 1.380659851 | -7.792860045 | 2.93E-14 | 1.44E-13 | 21.15766041 |
| DDIT4L | 1.253308435 | 1.599301489 | 7.782587991 | 3.16E-14 | 1.55E-13 | 21.08508335 |
| IGHV3-33 | 2.116069865 | 5.015910415 | 7.77818082 | 3.26E-14 | 1.60E-13 | 21.05396847 |
| BAIAP2L2 | 1.164239042 | 1.358485375 | 7.759690135 | 3.72E-14 | 1.82E-13 | 20.92357979 |
| CACHD1 | -1.06438097 | 1.99019212 | -7.758951446 | 3.74E-14 | 1.82E-13 | 20.91837612 |
| MACROD2 | -1.115054783 | 1.273294905 | -7.753619163 | 3.89E-14 | 1.89E-13 | 20.88082502 |
| CXCL3 | -1.095822583 | 1.7523543 | -7.75268941 | 3.91E-14 | 1.90E-13 | 20.87427965 |
| IGLV1-40 | 2.067001407 | 7.088169935 | 7.745281036 | 4.12E-14 | 2.00E-13 | 20.82214834 |
| MZB1 | 1.604982188 | 3.508160386 | 7.733571803 | 4.48E-14 | 2.18E-13 | 20.73983573 |
| CGREF1 | 1.007958212 | 1.047152812 | 7.732176349 | 4.53E-14 | 2.20E-13 | 20.73003287 |
| JSRP1 | 1.100420371 | 1.400203791 | 7.703147644 | 5.57E-14 | 2.68E-13 | 20.52643843 |
| RP11-284F21.7 | 1.161272873 | 1.294730863 | 7.703101559 | 5.57E-14 | 2.68E-13 | 20.52611571 |
| IGHJ3P | 1.84964227 | 2.929435524 | 7.70275816 | 5.59E-14 | 2.69E-13 | 20.52371101 |
| S100P | 2.924585383 | 4.990872844 | 7.699283261 | 5.73E-14 | 2.75E-13 | 20.49938261 |
| IGKV1-5 | 2.093744685 | 7.286003689 | 7.690178037 | 6.11E-14 | 2.93E-13 | 20.4356779 |
| CAPN5 | 1.176949968 | 2.756254137 | 7.683990002 | 6.38E-14 | 3.06E-13 | 20.39241854 |
| IGHV5-78 | 1.095713274 | 1.542745194 | 7.681176892 | 6.51E-14 | 3.12E-13 | 20.37276206 |
| ATP6V1C2 | 1.082060202 | 1.464356681 | 7.679668856 | 6.58E-14 | 3.15E-13 | 20.36222716 |
| SYT12 | 1.271976676 | 1.219888745 | 7.67918048 | 6.60E-14 | 3.16E-13 | 20.35881581 |
| SPATA18 | -1.111494393 | 1.562189846 | -7.675090761 | 6.80E-14 | 3.24E-13 | 20.33025569 |
| GPR115 | 1.033277276 | 1.040869977 | 7.668107621 | 7.14E-14 | 3.40E-13 | 20.28151849 |
| IGHJ3 | 1.945742541 | 4.500014885 | 7.657968801 | 7.68E-14 | 3.65E-13 | 20.21082173 |
| HSF4 | 1.051975342 | 1.950343002 | 7.654591941 | 7.86E-14 | 3.73E-13 | 20.18729233 |
| AGR2 | 1.799712557 | 6.743693366 | 7.644174486 | 8.47E-14 | 4.01E-13 | 20.11475888 |
| TGFB2 | -1.002913445 | 1.765665187 | -7.638951619 | 8.78E-14 | 4.15E-13 | 20.07842422 |
| IFT57 | -1.053399349 | 4.433073517 | -7.631118745 | 9.28E-14 | 4.38E-13 | 20.02397039 |
| IGHV5-51 | 2.14671425 | 6.704151438 | 7.611512193 | 1.07E-13 | 5.01E-13 | 19.88786771 |
| HIST1H4H | 1.281098899 | 2.504633926 | 7.60984518 | 1.08E-13 | 5.07E-13 | 19.87630909 |
| SLC7A11 | 1.323213452 | 1.729115332 | 7.603118805 | 1.13E-13 | 5.30E-13 | 19.82969138 |
| IGLV3-9 | 2.071320489 | 3.896739278 | 7.596015503 | 1.19E-13 | 5.57E-13 | 19.78049817 |
| SOCS3 | -1.072502736 | 5.709781779 | -7.59097305 | 1.23E-13 | 5.77E-13 | 19.74560012 |
| APOBEC3B | 1.141208494 | 1.920941314 | 7.586554035 | 1.27E-13 | 5.94E-13 | 19.71503248 |
| PLA1A | -1.068231367 | 2.007418576 | -7.581859997 | 1.31E-13 | 6.13E-13 | 19.68257848 |
| AOC1 | 1.90087875 | 2.112681053 | 7.576641916 | 1.36E-13 | 6.35E-13 | 19.64652073 |
| MT1A | -1.378384768 | 1.606984906 | -7.573575851 | 1.39E-13 | 6.48E-13 | 19.62534328 |
| LOXL2 | 1.161382321 | 2.909268329 | 7.563297197 | 1.50E-13 | 6.94E-13 | 19.55439969 |
| ALDH3B2 | 1.192822281 | 1.671402684 | 7.557980265 | 1.55E-13 | 7.20E-13 | 19.51773322 |
| IGHV4-55 | 1.446544571 | 2.565828901 | 7.554814984 | 1.59E-13 | 7.35E-13 | 19.49591501 |
| RAB15 | 1.039104822 | 3.457264523 | 7.553581972 | 1.60E-13 | 7.41E-13 | 19.48741792 |
| C2 | -1.166047409 | 3.708233654 | -7.549709643 | 1.65E-13 | 7.61E-13 | 19.46073988 |
| LYPD3 | 1.445625901 | 2.029478563 | 7.548829157 | 1.66E-13 | 7.66E-13 | 19.45467544 |
| DMBT1 | -2.144882202 | 3.200874294 | -7.533818995 | 1.84E-13 | 8.46E-13 | 19.351381 |
| RP11-284F21.10 | 1.377144232 | 1.474312782 | 7.528144983 | 1.91E-13 | 8.79E-13 | 19.31237873 |
| CTSH | -1.343264552 | 6.096979498 | -7.526924631 | 1.93E-13 | 8.86E-13 | 19.30399339 |
| MYEOV | 1.543004524 | 1.486294568 | 7.518984247 | 2.04E-13 | 9.35E-13 | 19.2494605 |
| MAP7D2 | 1.077260023 | 1.250065531 | 7.518483303 | 2.05E-13 | 9.38E-13 | 19.24602172 |
| RARRES2 | -1.139214323 | 5.108664177 | -7.518039348 | 2.05E-13 | 9.41E-13 | 19.2429743 |
| HLA-DPA1 | -1.271340369 | 6.29905517 | -7.510031712 | 2.17E-13 | 9.94E-13 | 19.18803341 |
| VCAN | 1.269659208 | 3.637925028 | 7.506111742 | 2.23E-13 | 1.02E-12 | 19.1611559 |
| LAMA3 | -1.350095622 | 2.504600971 | -7.491327814 | 2.48E-13 | 1.13E-12 | 19.05989341 |
| HLA-DRA | -1.270528332 | 9.948871048 | -7.489077141 | 2.51E-13 | 1.14E-12 | 19.04449192 |
| SCD | -1.056575173 | 6.14675229 | -7.485696889 | 2.57E-13 | 1.17E-12 | 19.02136784 |
| IGHV1-18 | 2.084645506 | 6.476730051 | 7.481875317 | 2.64E-13 | 1.20E-12 | 18.99523512 |
| IGHV3-43 | 1.807365793 | 3.20600347 | 7.477030795 | 2.73E-13 | 1.24E-12 | 18.96212314 |
| FNDC4 | 1.12350361 | 2.373471359 | 7.470306675 | 2.87E-13 | 1.30E-12 | 18.91619367 |
| CLDN10-AS1 | 1.081686841 | 1.000862533 | 7.464610021 | 2.98E-13 | 1.34E-12 | 18.87730914 |
| IGHV4-39 | 2.113372554 | 6.377078133 | 7.458323504 | 3.11E-13 | 1.40E-12 | 18.83442682 |
| UNC13D | -1.015694959 | 3.077268717 | -7.458102238 | 3.12E-13 | 1.41E-12 | 18.83291804 |
| B3GNT6 | 1.680721031 | 1.653174594 | 7.441576701 | 3.50E-13 | 1.57E-12 | 18.72033777 |
| IGHV1-69-2 | 2.13720709 | 5.20144014 | 7.437259451 | 3.60E-13 | 1.61E-12 | 18.6909606 |
| VMO1 | -1.049416435 | 2.537965043 | -7.435926476 | 3.64E-13 | 1.63E-12 | 18.6818931 |
| EGFL7 | -1.047113871 | 4.079742768 | -7.432773466 | 3.72E-13 | 1.66E-12 | 18.66045026 |
| RP11-356K23.1 | -1.080004136 | 0.862571832 | -7.423089048 | 3.97E-13 | 1.78E-12 | 18.59463612 |
| SHE | -1.074456336 | 1.940617756 | -7.418545388 | 4.10E-13 | 1.83E-12 | 18.56378251 |
| TMPRSS11E | 1.659629962 | 1.576430082 | 7.417460186 | 4.13E-13 | 1.84E-12 | 18.5564158 |
| SNORD14E | 1.040471554 | 1.583688536 | 7.391651264 | 4.94E-13 | 2.19E-12 | 18.38148005 |
| IGHV3-53 | 1.708232262 | 3.647171888 | 7.391484907 | 4.94E-13 | 2.19E-12 | 18.38035411 |
| RGS16 | -1.001161183 | 3.683610708 | -7.386798415 | 5.11E-13 | 2.26E-12 | 18.34864353 |
| PTK6 | 1.118269346 | 2.99799816 | 7.386512892 | 5.12E-13 | 2.27E-12 | 18.34671211 |
| IGLC3 | 1.951553848 | 7.905716768 | 7.385447193 | 5.15E-13 | 2.28E-12 | 18.33950375 |
| IGKV3D-15 | 1.601130028 | 2.466579502 | 7.36976646 | 5.74E-13 | 2.53E-12 | 18.23353977 |
| S100A2 | 1.722144973 | 3.201203609 | 7.368908565 | 5.77E-13 | 2.54E-12 | 18.22774787 |
| IGHV3-15 | 1.955609698 | 5.931517324 | 7.362262485 | 6.04E-13 | 2.66E-12 | 18.1828973 |
| IGHV4-28 | 1.601758705 | 3.226876574 | 7.354179848 | 6.39E-13 | 2.81E-12 | 18.12839766 |
| IGLV6-57 | 2.057493315 | 5.316911404 | 7.352152722 | 6.48E-13 | 2.84E-12 | 18.11473696 |
| PMAIP1 | 1.136368979 | 2.889911307 | 7.35166706 | 6.50E-13 | 2.85E-12 | 18.11146458 |
| CD79A | 1.554486842 | 3.597971433 | 7.350438893 | 6.55E-13 | 2.87E-12 | 18.10319001 |
| HILPDA | 1.109072982 | 3.281520926 | 7.345455394 | 6.78E-13 | 2.97E-12 | 18.06962634 |
| RP11-44F21.5 | 1.044575333 | 1.596804303 | 7.34507686 | 6.80E-13 | 2.97E-12 | 18.06707771 |
| RP11-510N19.5 | 1.059570724 | 1.740583904 | 7.335260539 | 7.27E-13 | 3.17E-12 | 18.00102353 |
| IGLV3-1 | 2.005217138 | 5.864988132 | 7.330311694 | 7.52E-13 | 3.28E-12 | 17.9677506 |
| COL17A1 | 1.729561356 | 2.04560833 | 7.321077374 | 8.01E-13 | 3.48E-12 | 17.90571492 |
| FNDC1 | 1.231865226 | 2.087008857 | 7.319054024 | 8.13E-13 | 3.52E-12 | 17.89213087 |
| CLDN3 | 1.547633416 | 5.486849984 | 7.312269319 | 8.51E-13 | 3.69E-12 | 17.84660367 |
| PTN | -1.222668618 | 2.199253048 | -7.308516995 | 8.73E-13 | 3.78E-12 | 17.8214397 |
| MIF | 1.05335692 | 5.070691033 | 7.307955331 | 8.77E-13 | 3.79E-12 | 17.81767399 |
| HIST1H2BC | 1.244890128 | 2.640098365 | 7.301853968 | 9.14E-13 | 3.94E-12 | 17.77678246 |
| IGHV3-21 | 1.982627608 | 5.784469073 | 7.30169262 | 9.15E-13 | 3.95E-12 | 17.7757015 |
| GALNT6 | 1.175117565 | 2.94099332 | 7.299658903 | 9.28E-13 | 4.00E-12 | 17.76207805 |
| MS4A8 | -1.450558474 | 1.370701149 | -7.295307731 | 9.56E-13 | 4.11E-12 | 17.73294112 |
| CTD-2619J13.13 | 1.13700758 | 1.499334711 | 7.294071781 | 9.64E-13 | 4.14E-12 | 17.72466742 |
| FOXA1 | 1.093881408 | 3.981159946 | 7.290420106 | 9.88E-13 | 4.24E-12 | 17.70022923 |
| IGHJ2 | 1.808517992 | 3.49279632 | 7.289337639 | 9.95E-13 | 4.27E-12 | 17.69298697 |
| IGLV3-19 | 2.112426475 | 6.800138078 | 7.286354165 | 1.02E-12 | 4.36E-12 | 17.67303066 |
| NKG7 | -1.216797221 | 3.359488112 | -7.269594427 | 1.14E-12 | 4.87E-12 | 17.56105258 |
| IGLV2-14 | 1.968080448 | 7.163465082 | 7.261488628 | 1.20E-12 | 5.13E-12 | 17.50697211 |
| IGKV3-7 | 1.445241898 | 2.380136356 | 7.251111832 | 1.29E-12 | 5.49E-12 | 17.43781366 |
| IRX2 | -1.608814031 | 2.742621437 | -7.238391214 | 1.41E-12 | 5.96E-12 | 17.3531473 |
| SEZ6L2 | 1.262257669 | 4.102196604 | 7.236342879 | 1.43E-12 | 6.04E-12 | 17.33952556 |
| IGKV1-9 | 1.949831643 | 5.392896879 | 7.233964441 | 1.45E-12 | 6.14E-12 | 17.32371263 |
| LINC00261 | -1.168842342 | 1.578824945 | -7.232941658 | 1.46E-12 | 6.18E-12 | 17.31691405 |
| HIST1H2BK | 1.047405474 | 5.861863883 | 7.225569113 | 1.53E-12 | 6.48E-12 | 17.26793156 |
| HLA-DRB5 | -1.552262297 | 7.444428044 | -7.224457466 | 1.55E-12 | 6.53E-12 | 17.26054951 |
| CDHR3 | -1.0984104 | 1.12748901 | -7.221592222 | 1.58E-12 | 6.65E-12 | 17.24152686 |
| AHNAK2 | 1.20586379 | 2.18765236 | 7.220969732 | 1.58E-12 | 6.67E-12 | 17.23739492 |
| AIM2 | 1.277038231 | 1.884553026 | 7.215628831 | 1.64E-12 | 6.90E-12 | 17.20195558 |
| HLA-DRB1 | -1.280672684 | 9.059201506 | -7.214231664 | 1.66E-12 | 6.97E-12 | 17.19268836 |
| COL1A2 | 1.288069356 | 6.81561828 | 7.213383049 | 1.67E-12 | 7.00E-12 | 17.18706035 |
| MUC3A | 1.454186331 | 1.601417992 | 7.194695328 | 1.89E-12 | 7.91E-12 | 17.06326428 |
| MXRA5 | 1.188296642 | 3.330507594 | 7.190805821 | 1.94E-12 | 8.11E-12 | 17.0375323 |
| SNORD104 | 1.092451932 | 3.58924211 | 7.18739521 | 1.99E-12 | 8.29E-12 | 17.01497818 |
| IGHV3-30 | 1.886057099 | 5.79187032 | 7.185628531 | 2.01E-12 | 8.39E-12 | 17.00329881 |
| ALDH3B1 | -1.106212055 | 4.019994345 | -7.184001367 | 2.03E-12 | 8.47E-12 | 16.99254388 |
| HLA-DPB1 | -1.170372926 | 7.025453073 | -7.183227875 | 2.04E-12 | 8.51E-12 | 16.98743211 |
[truncated: 221,960 more chars]
